# Expression of *HMA4* cDNAs of the zinc hyperaccumulator *Noccaea caerulescens* from endogenous *NcHMA4* promoters does not complement the zinc-deficiency phenotype of the *Arabidopsis thaliana hma2hma4* double mutant

**DOI:** 10.3389/fpls.2013.00404

**Published:** 2013-10-16

**Authors:** Mazhar Iqbal, Ismat Nawaz, Zeshan Hassan, Henk W. J. Hakvoort, Mattijs Bliek, Mark G.M. Aarts, Henk Schat

**Affiliations:** ^1^Department of Genetics, Faculty of Earth and Life Sciences, Vrije UniversiteitAmsterdam, Netherlands; ^2^Laboratory of Genetics, Wageningen UniversityWageningen, Netherlands

**Keywords:** *Noccaea caerulescens*, *HMA4*, promoter activity, Zn deficiency, Zn translocation, gene expression

## Abstract

*Noccaea caerulescens* (*Nc*) exhibits a very high constitutive expression of the heavy metal transporting ATPase, *HMA4*, as compared to the non-hyperaccumulator *Arabidopsis thaliana* (*At*), due to copy number expansion and altered *cis*-regulation. We screened a BAC library for *HMA4* and found that *HMA4* is triplicated in the genome of a *N. caerulescens* accession from a former Zn mine near La Calamine (LC), Belgium. We amplified multiple *HMA4* promoter sequences from three calamine *N. caerulescens* accessions, and expressed *AtHMA4* and different *NcHMA4* cDNAs under *At* and *Nc* *HMA4* promoters in the *A. thaliana* (Col) *hma2hma4* double mutant. Transgenic lines expressing *HMA4* under the *At* promoter were always fully complemented for root-to-shoot Zn translocation and developed normally at a 2-μM Zn supply, whereas the lines expressing *HMA4* under *Nc* promoters usually showed only slightly enhanced root to shoot Zn translocation rates in comparison with the double mutant, probably owing to ectopic expression in the roots, respectively. When expression of the Zn deficiency responsive marker gene *ZIP4* was tested, the transgenic lines expressing *AtHMA4* under an *NcHMA4-1-LC* promoter showed on average a 7-fold higher expression in the leaves, in comparison with the double *hma2hma4* mutant, showing that this construct aggravated, rather than alleviated the severity of foliar Zn deficiency in the mutant, possible owing to expression in the leaf mesophyll.

## INTRODUCTION

Zinc (Zn) is an essential element for all organisms. However, it is toxic when taken up in excess (Marschner, 1995; Kamal et al., 2004). Therefore, all organisms tightly regulate their cellular Zn status (Clemens, 2001). The network underlying Zn homeostasis in plants is incompletely known, but a number of Zn transporters of the ZIP/IRT, MTP, and HMA families have been shown to play essential roles in the acquisition, plant-internal transport and sequestration of Zn (Guerinot, 2000; Hussain et al., 2004; Papoyan and Kochian, 2004; van de Mortel et al., 2006; Gustin et al., 2009; Wong and Cobbett, 2009; Shahzad et al., 2010). Heavy metal transporting ATPases (HMAs) constitute the P_1__b_ subfamily of cation transporter ATPases. In *Arabidopsis thaliana* this subfamily is represented by eight members, of which HMA1 – HMA4 and HMA5 – HMA8 are transporting divalent and univalent heavy metal cations, respectively. All HMAs are effluxing heavy metal ions from the cytosol, either into the apoplast, the vacuole, or other organelles (Hussain et al., 2004; Andrés-Colás et al., 2006; Kim et al., 2009; Ueno et al., 2011). In *A. thaliana*, HMA2 and HMA4 are plasma membrane-localized, particularly expressed in the xylem parenchyma of the roots, and supposed to be involved in the loading of Zn and Cd into the xylem (Hussain et al., 2004; Wong and Cobbett, 2009). They seem to be partly redundant, since the single knock-out mutants, *hma4* and *hma2*, have only a modest or no phenotype for Zn root-to-shoot transport respectively, whereas the double mutant has a very strong phenotype under normal Zn supply, including stunted growth, chlorosis and infertility (Hussain et al., 2004).

A minority of plant species, called metallophytes, are capable to grow and reproduce on strongly heavy-metal enriched “metalliferous” soils. These plants, or at least their metallicolous populations, exhibit extraordinary high levels of tolerance, also called hypertolerance (Clemens, 2006), to particular heavy metals (Antonovics et al., 1971; Ernst, 1974; Macnair, 1993). In so-called facultative metallophytes or pseudometallophytes, i.e., species occurring on non-metalliferous as well metalliferous soils, hypertolerance is largely metal-specific and confined to the metal or metals present at toxic concentrations in the soil at the site of population origin (Schat et al., 1996; Schat and Vooijs, 1997). A small fraction of metallophytes, about 450 species worldwide, are classified as metal hyperaccumulator plants, accumulating particular heavy metals at extremely high concentrations in their foliage (Baker et al., 2000; van der Ent et al., 2013). Most of them hyperaccumulate nickel (Ni), but some of them mostly Zn, and/or cadmium (Cd), such as *Noccaea caerulescens* (formerly known as *Thlaspi caerulescens*) and *Arabidopsis halleri* (Cosio et al., 2004).

The mechanisms of hypertolerance and hyperaccumulation in metallophytes are far from completely understood. However, Cd and Zn hyperaccumulation and hypertolerance in *Arabidopsis halleri* have been shown to depend on a strongly enhanced expression of *HMA4*, which is in this species effected by tandem triplication and altered *cis*-regulation in comparison to *A. thaliana* (Courbot et al., 2007; Willems et al., 2007; Hanikenne et al., 2008). Recently, enhanced *HMA4* expression, due to tandem quadruplication and altered *cis*-regulation, has also been demonstrated in *Noccaea caerulescens*, which is a remarkable case of parallel molecular evolution, since the hyperaccumulation trait must have been independently evolved in *Noccaea* and *Arabidopsis* (Ó Lochlainn et al., 2011).

Although enhanced *HMA4* expression is doubtlessly essential for the hypertolerance and foliar allocation of Zn and Cd in hyperaccumulators, as convincingly demonstrated by RNAi-mediated silencing in *A. halleri* (Hanikenne et al., 2008), it does not seem to be sufficient to confer significant levels of Cd or Zn hypertolerance or hyperaccumulator-like foliar accumulation rates in a non-hyperaccumulator/non-metallophyte genetic background. Heterologous expression of *AhHMA4*, under the *AhHMA4* promoter, yielded enhanced Zn or Cd sensitivity, manifested as reduced shoot growth and chlorosis, but without considerably enhanced foliar metal accumulation in *A. thaliana* (Hanikenne et al., 2008) and tomato (Barabasz et al., 2012). The reason for this is still elusive, and further characterization of the functioning of hyperaccumulator *HMA4* genes in a non-hyperaccumulator genetic background is therefore required. Moreover, although the three *AhHMA4* copies seem to show very similar expression patterns (Hanikenne et al., 2008), it cannot be excluded that there is some degree of functional differentiation among them. Therefore, in the present study we made an attempt to more precisely characterize the *HMA4* cDNAs and *HMA4* promoters from *Noccaea caerulescens*, through expression in the *A. thaliana hma2hma4* double mutant. We were particularly interested in the potential of *NcHMA4* to revert the foliar Zn deficiency phenotype of the double mutant. We also phenotyped the transgenic lines for Cd tolerance and translocation. To better understand the function of HMA4 in hyperaccumulators and non-hyperaccumulators, we expressed the *NcHMA4* cDNAs under the *AtHMA4* promoter and *AtHMA4 *under *NcHMA4 *promoters, and compared the Zn and Cd translocation phenotypes of the transgenic lines with those of lines expressing *AtHMA4* under the native *AtHMA4* promoter. To detect potential differences in tissue or cell type specificity, we made the promoter::GUS constructs for the *N. caerulescens* *HMA4 *promoters and the *A. thaliana* one and compared their activities.

## MATERIALS AND METHODS

### PLANT MATERIALS AND EXPERIMENTAL CONDITIONS

Seeds of *A.* *thaliana* (Col) wild-type, the *hma2hma4* double mutant and transgenic lines were sterilized in 96% ethanol, then 10% bleach, washed three times with sterilized water, suspended in 0.1% agarose and sown on 0.8% (w/v) gelrite plates (Duchefa, G1101.0250) containing half-strength Murashige and Skoog (MS) medium at pH 5.7–5.9 with 25 μg/ml hygromycin for the transgenic lines and no antibiotics for wild-type on square petri plates that were vertically placed. Seeds were germinated at 22°C under a 10 h day^-^^1^ photoperiod. After 2 weeks seedlings were transferred to hydroponics culture in 1-L polyethylene pots (three plants per pot, each plant of a different genotype) containing a modified half-strength Hoagland’s solution (Schat and Ten Bookum, 1992). Plants were grown in a climate room at 20/15°C day/night, light intensity 220 μmol m^-^^2^ s^-^^1^ at plant level, 10 h day^-^^1^, 75% RH. Nutrient solutions were renewed weekly. After 2 weeks in hydroponics, plants were exposed to five different concentrations of Cd (0.5, 12, 25 and 50 μM) and two concentrations of Zn (2 and 10 μM), supplied as CdSO_4_ or ZnSO_4_, ten plants per treatment. Before exposure, roots were stained with active carbon powder (to facilitate the measurement of root length increment) and washed with demineralised water (Schat and Ten Bookum, 1992). After five days of exposure, root growth, i.e., the length of the longest unstained root segment was measured.

Seeds of *N. caerulescens*, collected from the populations near La Calamine (LC), Belgium, and Saint Laurent de Miniers (this population is also known as Ganges, Ga) and Col du Mas de l’Aire (CMA), South-France, were sown on garden soil (Jongkind B.V., number 6, Aalsmeer, The Netherlands). Site and accession characteristics are given in Assunção et al. (2003; LC and Ga). CMA is another lead mine from the region around the village of Ga. Some accumulation and tolerance characteristics of the accessions are given in Peer et al. (2003) and Mohtadi et al. (2012). Two-weeks-old seedlings were transferred to hydroponics in 1-L polyethylene pots containing a modified half-strength Hoagland’s solution (Schat and Ten Bookum, 1992). After 2 weeks leaves and roots were harvested, snap-frozen in liquid nitrogen and stored at -80°C until RNA extraction.

### DETERMINATION OF Cd AND Zn CONCENTRATIONS

Cd and Zn concentrations were determined in roots and shoots (10 plants per population per concentration, pooled two by two, to make five samples) of wild-type, mutant and transgenic lines. Roots were carefully rinsed with ice-cold PbNO_3_ (5 mM) for 30 min and blotted with tissue paper. Cd and Zn were determined by digesting 50–100 mg of oven-dried plant material in 2 ml of a 1:4 (v/v) mixture of 37% (v/v) HCl and 65% (v/v) HNO_3_ in Teflon bombs for 7 h at 140°C, after which the volume was adjusted to 10 ml with demineralised water. Cd and Zn were determined on a flame atomic absorption spectrophotometer (Perkin Elmer AAS100).

### RNA AND DNA EXTRACTION AND 1st STRAND cDNA SYNTHESIS

RNA was extracted from frozen root and shoot tissues using Trizol^TM^ (Invitrogen), following the manufacturer’s instructions and as described in Jack et al.**(2007). Single-stranded cDNA was synthesized from total RNA (2.5 μg, boiled for 1 min) using 100 Units M-MLV Reverse Transcriptase (Invitrogen), 2 mM dNTPs, 100 mM DTT, 10× RT buffer and 10 μM oligo dT primer at 42°C for 1 h. DNA was isolated according to Rivera et al. (1999).

### EXPRESSION ANALYSIS

cDNA was synthesized following the manufacturer’s protocol. Based on the sequence from the National Center for Biotechnology Information (NCBI) database^1^, intron spanning specific primers matching all of the HMA4 cDNAs were designed for real-time quantitative reverse transcriptase (RT) PCR (qRT-PCR; Table S1, Supplementary Material). ACT2 was used as a positive internal control. The position of the intron was predicted by aligning coding sequences of NcHMA4 with the AtHMA4. Quantitative RT-PCR was performed using SensiMix^TM^ SYBR No-ROX kit (Bioline) using the Bio-Rad MJ Research Opticon^TM^ Real Time PCR detection system (Life Technologies; Invitrogen). SensiMix^TM^ SYBR No-ROX kit includes the SYBR^®^ Green I dye, dNTPs, stabilizers and enhancers. A dilution range in water of the cDNA samples was tested to identify the cDNA concentration that produced a C_T_ between 15 and 30 cycles. The final reaction conditions were, 10 μl SensiMix^TM^ SYBR No-ROX master mix, 0.75 μl forward primer (final concentration of 250 nM), 0.75 μl of reverse primer (final concentration of 250 nM) and cDNA in a total reaction volume of 20 μl. An initial step of 95°C for 10 min was used to activate the polymerase. Cycling conditions were: melting step at 95°C for 10 s and annealing-extension at 60°C for 20 s, with 40 cycles, at the end melting curve from 60 to 90°C, read every 0.5°C, hold 10s. All qPCR reactions were performed in triplicate, and a maximum difference of one cycle between the C_T_ of the triplicate samples was considered acceptable. Negative controls were included for each primer pair to check for significant levels of any contaminants. Expression values were calculated using the 2^-^^Δ^^Δ^^CT^ method (Livak and Schmittgen, 2001).

### DNA BLOT HYBRIDISATION

We screened a BAC library of LC for *HMA4*. BAC clone DNA was extracted from 5 ml of overnight culture grown in LB medium containing 12.5 μg/ml chloramphenicol, using the Miniprep Plasmid DNA Purification kit (QIAGEN) but skipping the column purification step. For Southern analyses, 2 μg of BAC clone DNA from two clones was digested with restriction enzymes (EcoRV, XbaI, BamHI, HindIII, EcoRI and PstI) at 37°C for 6–7 h and separated on a 0.8% (w/v) agarose gel in full-strength TEA buffer (20 mM Tris-acetate; 1 mM EDTA). Then, the agarose gel was submerged into 0.25 N HCl for 15 min and rinsed with water 2–3 times for 15 min. DNA fragments were transferred and cross-linked onto a positively charged nylon membrane (Hybond-N+, Amersham Biosciences) by capillary blotting using 0.4 N NaOH overnight (±16 h). For DNA blot analysis of BAC clones, an *NcHMA4* probe was obtained from a PCR fragment of 521 bp produced from LC genomic DNA using forward (5′-ACAGGAAGAAAGTTGAAGGCGG-3′) and reverse (5′-CCTCACTAGCAA-GCAACAAACG-3′) primers designed on the last exon and purified using PCR purification kit (QIAGEN). Fifty ng of purified PCR product was radioactively labeled with α[^32^P] dCTP using the Klenow fragment of DNA polymerase with random hexamer primers. Prehybridization was carried out in Gilbert and Clark buffer (0.5 M phosphate buffer, pH 7.2; 7% SDS; 1% BSA) for 2 h. Hybridization was carried out in the same buffer overnight at 65°C. Then the blot was washed at 55°C with 2×SSC and 0.1% SDS. The final and more stringent wash was 0.5×SSC, 0.1% (w/v) SDS, again at 55°C (Sambrook et al., 1989). Images were made using a Storm 820 Phospho-imager.

### GENERATION AND CHARACTERIZATION OF TRANSFORMED LINES

Several chromosome walks were performed using DNA of N. caerulescens, accessions LC, Ga and CMA, to pick up the promoters of HMA4 using primers listed in Table S2 (Supplementary Material). NcHMA4-Ga promoter 1 and 2 were amplified using forward primers designed on the promoter sequence of Saint Laurent Le Minier HMA4 (Ó Lochlainn et al., 2011). AtHMA4 cDNA was amplified from a cDNA clone obtained from Riken, Japan (Resource number pda 08214, cDNA clone RAFL 09-32-D05). The sequence from the start codon of AtHMA4 until the upstream gene was taken as the AtHMA4 promoter (8274880-8278980 and 3 nucleotides of 5′ UTR just before ATG in TAIR^2^. Amplicons with the Noccaea promoters fused with the AtHMA4 and NcHMA4 cDNAs, the AtHMA4 promoter with the NcHMA4 and AtHMA4 cDNAs, and HMA4 promoters were prepared using attB1 and attB2 sites flanking the forward and reverse primers. In case of promoter::cDNA fused products, promoters and cDNAs were amplified separately with the overlapping parts at the fusion side and then fused in a single PCR (10 cycles without primers [as the overlapping parts will elongate to complete the fragment], then 25 cycles with atB1 forward and attB2 reverse primer to amplify the whole fragment). PCR reactions were performed using the “Phusion^®^ High Fidelity DNA Polymerase” (Finnzymes). The primers used to amplify these products are given in the Supplementary Material under “Supplementary methods.” All DNA recombinant techniques were performed according to the Invitrogen GATEWAY Cloning System (Karimi et al., 2002). We used pHGWFS7 as a destination vector for promoter analysis, and pH7WG2 without p35S for all other constructs (Karimi et al., 2002). All the fragments were confirmed by sequencing. Transformation with these binary vectors confers resistance to hygromycin in transformed plant cells. These binary vectors were introduced into the Agrobacterium tumefaciens strain C58 (pMP90) by electroporation.

Seeds of the**homozygous *A. thaliana hma2hma4* double mutant (Col; Hussain et al., 2004) were kindly provided by Prof. Chris Cobbett, University of Melbourne. *A.thaliana *(wt) and *Athma2hma4 *double mutants were transformed by *A. tumefaciens* containing the promoter and all other constructs, respectively, given in **Table 1**, using the flower dip technique (Clough and Bent, 1998). T_0_ seeds (three lanes with ± 1000 seeds each) were surface-sterilized and sown on 0.8% (w/v) gelrite plates containing half-strength MS medium at pH 5.7–5.9 with 50 μg/ml hygromycin for screening. The plates were kept vertically to record the root growth. After 2 weeks, there was a clear difference in root growth between the transgenic and untransformed plants. The transgenic plants were transferred to a nutrient solution containing a modified half-strength Hoagland’s nutrient solution (see above). After 2 weeks in hydroponics, samples were taken from roots and leaves to extract RNA. RNA was isolated using Trizol. cDNA was synthesized using M-MLV from Invitrogen. Then the relative transcript levels were measured by RT-qPCR, using actin-2 as a positive internal control (see above). The primers are given in Table S1, Supplementary Material.

**Table 1:** Constructs used for *A. thaliana* transformation and phenotyping experiments.

*pAtHMA4::GUS*	*pAtHMA4::NcHMA4-2-LC*
*pNcHMA4-1-LC::GUS*	*pNcHMA4-1-LC::NcHMA4-1-LC*
*pNcHMA4-2-LC::GUS*	*pNcHMA4-1-LC::NcHMA4-2-LC*
*pNcHMA4-3-LC::GUS*	*pNcHMA4-1-LC::AtHMA4*
*pNcHMA4-1-Ga::GUS*	*pNcHMA4-2-LC::AtHMA4*
*pNcHMA4-2-Ga::GUS*	*pNcHMA4-3-LC::AtHMA4*
*pNcHMA4-3-Ga::GUS*	*pNcHMA4-1-Ga::AtHMA4*
*pNcHMA4-1-CMA::GUS*	*pNcHMA4-2-Ga::AtHMA4*
*pNcHMA4-2-CMA::GUS*	*pNcHMA4-3-Ga::AtHMA4*
*pAtHMA4::AtHMA4*	*pNcHMA4-1-CMA::AtHMA4*
*pAtHMA4::NcHMA4-1-LC*	*pNcHMA4-2-CMA::AtHMA4*

*pNcHMA4-1-LC* and *pNcHMA4-2-LC* were used in a LR reaction with the pKGWGG-RR vector (Limpens et al., 2004) through Gateway^®^ LR Clonase^TM^ II Enzyme Mix (Invitrogen^TM^) to create an expression clone. Sequence reactions were done using the big dye terminator protocol. These expression clones were inserted separately in *Agrobacterium rhizogenes* by electroporation, and then used for hairy root transformation of *N. caerulescens*.

### HAIRY ROOT TRANSFORMATIONS OF *N. CAERULESCENS*

Roots of *N. caerulescens *were transformed with *pNcHMA4-LC* constructs via *Agrobacterium rhizogenes *mediated transformation. Single colonies of transformed *A. rhizogenes *(MSU 440) were cultured in 3 ml LB media with 100 μg/ml spectinomycin at 28°C overnight. Two hundred μl from the liquid culture were plated on LB-agar plates containing the same antibiotic and grew for 2 days at 28°C. Seeds of *N. caerulescens *accession LC were sown in four agar plates containing half-strength MS (pH 5.8), 25 seeds per plate. Seven-days-old plants had their roots removed just below the hypocotyl. One dot of aggregated *A. rhizogenes *bacteria was put at the tip of each hypocotyl. For co-culturing, plates were incubated in a climate chamber, set at 250 μmol m^-^^2^ s^-^^1^ light at plant level, during a 16-h day period, 24°C, and 70% relative humidity. After 5 days, seedlings were transferred to new ½ MS-agar plates supplemented with 100 μg/ml tricarcillin to kill the bacteria. Roots were checked every three days using a stereomicroscope under UV, for expression of the DsRED protein indicating transformation. Non-transformed roots were removed. After 8 weeks, the plants showing fully transformed root systems were analyzed for GUS reporter activity.

### GUS ASSAY

Seeds of *A. thaliana* primary transformants containing *AtHMA4* or *NcHMA4* promoter – GUS fusions were sown on 0.8% (w/v) gelrite plates containing half-strength MS medium at pH 5.7–5.9 with 25 mg l^-^^1^ hygromycin. GUS activity was determined in 2-week-old *A. thaliana *seedlings growing on MS gelrite plates, and in transformed *N. caerulescens* roots by staining overnight in a solution containing 1 mM X-Gluc in 50 mM sodium phosphate at pH 7.5. After the incubation, 70% ethanol was used to remove chlorophyll. Staining patterns were observed and photographed using a binocular microscope (bright light).****

### SEQUENCE AND PHYLOGENETIC ANALYSES

Complete coding sequences of *HMA4* were amplified using primers designed on the basis of the sequence available in the database (Bernard et al., 2004). Data base searches were conducted with BLAST^1^ and TAIR^2^. The nucleotide sequences were aligned using ClustalW2^3^. A phylogenetic tree was constructed using MEGA4 (Kumar et al., 2008) with Neighbor-joining and 1000 bootstrap replicates.

### STATISTICS

Statistical analysis was performed using one-way and two-way ANOVA. The MSR statistic was used for *a posteriori* comparisons of individual means (Sokal and Rohlf, 1981). To obtain homogeneity of variances, all the data were subjected to logarithmic transformation prior to analysis, except for the root elongation data.

## RESULTS

### DNA BLOT ANALYSIS

**FIGURE 1:**
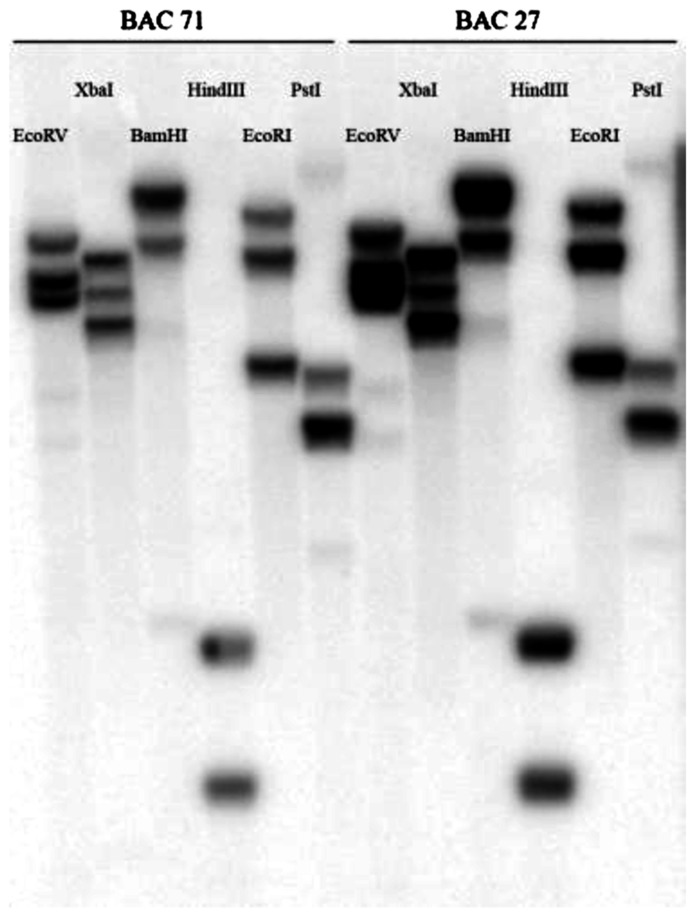
**Southern blot of two BAC clones of* N. caerulescens*, accession La Calamine.** Each clone was restricted by six different restriction enzymes (EcoRV, XbaI, BamHI, HindIII, EcoRI and PstI). Hybridization was done with a 521 bp probe from the last exon of *HMA4*.

A genomic BAC library of *N. Caerulescens*, LC, with an average insert size of 135 Kb was constructed by EPICENTRE Biotechnologies. Eight 384 well plates were screened for *HMA4*. We found two clones containing *HMA4*. DNA blot analysis showed that most of the lanes contained three bands, suggesting that *HMA4* is at least triplicated in LC (**Figure 1**), because we used restriction enzymes that do not cut within the probed exon.

### *N. CAERULESCENS HMA4* CODING AND PROMOTER SEQUENCES

We constructed phylogenetic trees for *NcHMA4s* and other known *HMA4* coding sequences and promoters (Figure S1 and S2, Supplementary Material). *A. halleri* *HMA4 *cDNAs are about 88% identical with the *A. thaliana HMA4* cDNA (Hanikenne et al., 2008). *N. caerulescens* *HMA4 *cDNAs are less similar to the *AtHMA4* cDNAs, sharing 80 to 85% identity. All the *NcHMA4*s share about 98% identity. On an amino acid basis AhHMA4s and NcHMA4s are around 86%, or 71 to 76% identical with AtHMA4, respectively and NcHMA4s are 94 to 99% identical among each other (Table S3-A, Supplementary Material).

When comparing promoter sequences, the *NcHMA4* promoters are 36 to 39% identical with the *AtHMA4* promoter, and 38 to 43% identical with the *AhHMA4* promoters. When compared among each other, the *NcHMA4* promoters share 36 to 96% identity (Table S3-B and Alignment S3, Supplementary Material).

### EXPRESSION OF *HMA4* IN *A. THALIANA * AND *N. CAERULESCENS*

**FIGURE 2:**
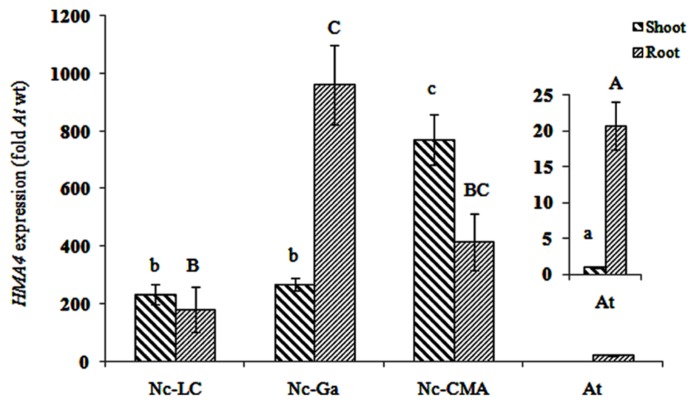
*** HMA4* expression in three *Noccaea caerulescens* accessions (fold *A. thaliana* in shoot)**La Calamine**(LC), Ganges (Ga) and Col du Mas de l’Aire (CMA)**. Significant differences (*P* < 0.05) between means are indicated, separately for root and shoot, by different superscripted letters.

Transcript levels of *HMA4* were determined by qRT-PCR in *A. thaliana* and *N. caerulescens* shoots and roots. In *A. thaliana* *HMA4* was mainly expressed in the root, i.e., 20 times higher than in the shoot. In *N. caerulescens *LC and CMA *HMA4* was higher expressed in the shoot than in the root, whereas in Ga it was higher expressed in the root (**Figure 2**). Assuming equal expression of *Act2*, the shoot and root *HMA4* transcript concentrations were about 400-fold and 25-fold higher, respectively, in *N. caerulescens* than they were in *A. thaliana*.

### TRANSGENIC *HMA4* EXPRESSION USING *N. CAERULESCENS* AND *A. THALIANA HMA4* PROMOTERS

**FIGURE 3:**
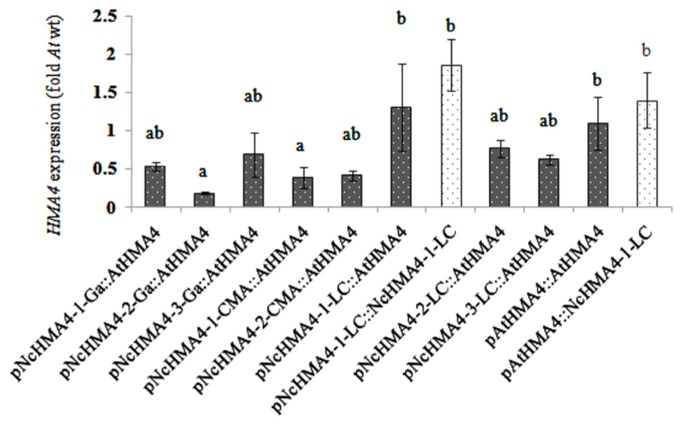
***AtHMA4* (solid bars) and *NcHMA4-1-LC* (open bars) expression under *A. thaliana* and *N. caerulescens* promoters in the root of transgenic *A. thalianahma2hma4 *double mutants, relative to the average expression in the root of *A. thaliana* wild-type which is considered as 1 (means of 5 plants ± SE).** Significant differences (*P* < 0.05) between means are indicated by different superscripted letters.

**FIGURE 4:**
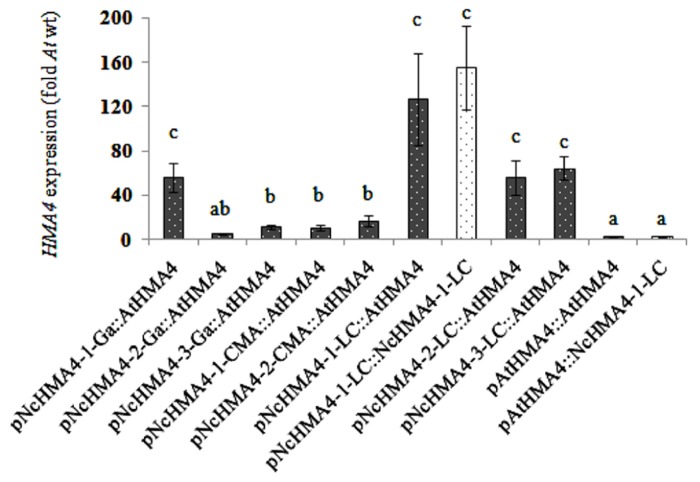
***AtHMA4* (solid bars) and *NcHMA4-1-LC* (open bars) expression under *A. thaliana* and *N. caerulescens* promoters in the shoot of transgenic *A. thaliana* relative to the expression in the shoot of *A. thaliana* wild-type which is considered as 1 (means of 5 plants ± SE).** Significant differences (*P* < 0.05) between means are indicated by different superscripted letters.

**FIGURE 5:**
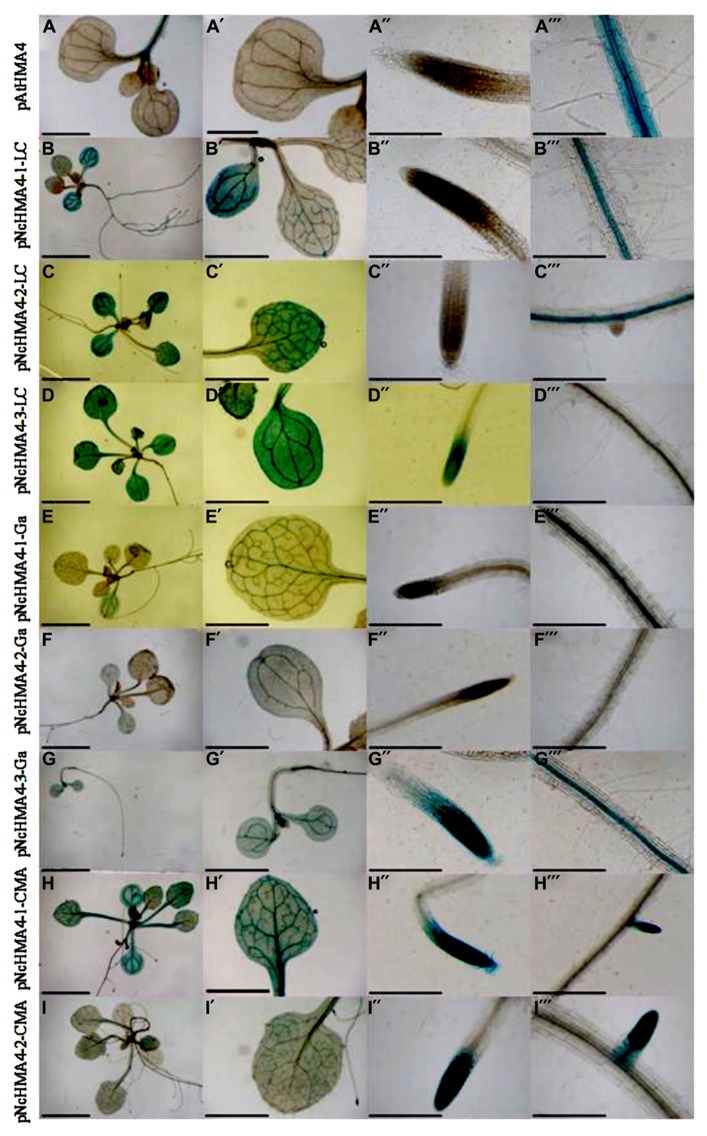
***Arabidopsis* (wt) plants expressing GUS under *AtHMA4* and *NcHMA4* promoters from the accessions LC, Ga and CMA.** [Scale bar = 5 mm in **(A)** to **(I)**, 2 mm in **(A′)** to **(I′)** and 30 μm in **(A′′**) to **(I′′′**)].

We expressed *NcHMA4-1-LC* and *AtHMA4* cDNAs under different *NcHMA4* promoters and the *AtHMA4* promoter in the *Athma2hma4* double mutant. There was no significant effect of the cDNA source on the expression levels in 4-weeks old primary transgenic plants (**Figures 3** and **4**). When expressed under *Nc* promoters, the average transgene expression levels in the roots were, surprisingly, lower than those under the endogenous *AtHMA4* promoter, except for the *pNcHMA4-1-LC*. The latter and the *At* promoter both yielded expression levels close to the wild-type level in untransformed *A. thaliana* (**Figure 3**). In the shoot, however, all the *Nc* promoters tested were significantly (4- to 60-fold) more active than the *At* one (**Figure 4**). To compare the patterns of tissue-specificity, we also expressed, under the same promoters, *GUS* in wild-type *A. thaliana*. When expressed under the *AtHMA4* promoter, GUS activity was consistently high in the root stele, but negligible in the root tip. When expressed under *NcHMA4* promoters, however, GUS activity was often low or negligible in the root stele, but (extremely) high all over the apical 2-mm root segment (**Figure 5**). Moreover, the *NcHMA4* promoters often produced considerable GUS activity in the leaves, occasionally all over the leaf blade, rather than confined to the veins, whereas the *AtHMA4* promoter was barely active in the leaves (**Figure 5**).

**FIGURE 6:**
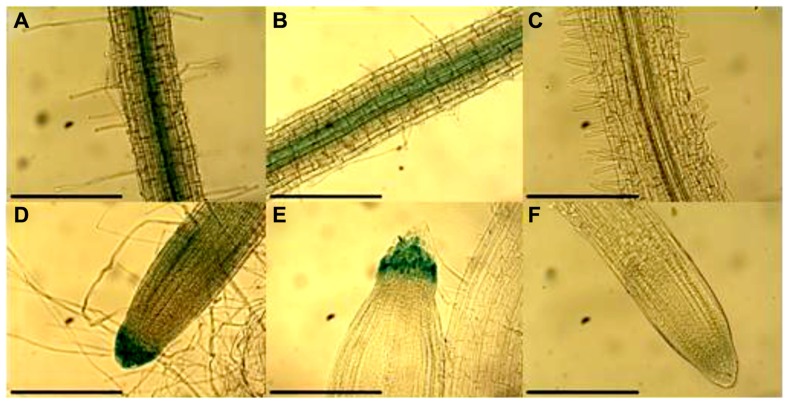
***GUS* expression under the (A) and (D) *pNcHMA4-1-LC*, (B) and (E) *pNcHMA4-2-LC* in *N. caerulescens *roots.** **(C)** and **(F)** are control roots [Scale bar = 30 μm].

*GUS* was also expressed under the *pNcHMA4-1-LC* and *pNcHMA4-2-LC* in *N. caerulescens*, using *A. rhizogenes*-mediated root transformation. Staining was observed mainly in the stele, like *pAtHMA4*, and the root tip, though exclusively in the root cap (**Figure 6**).

### MUTANT COMPLEMENTATION WITH *Nc* AND *At* HMA4 PROMOTER-DRIVEN *HMA4 *cDNAs

**FIGURE 7:**
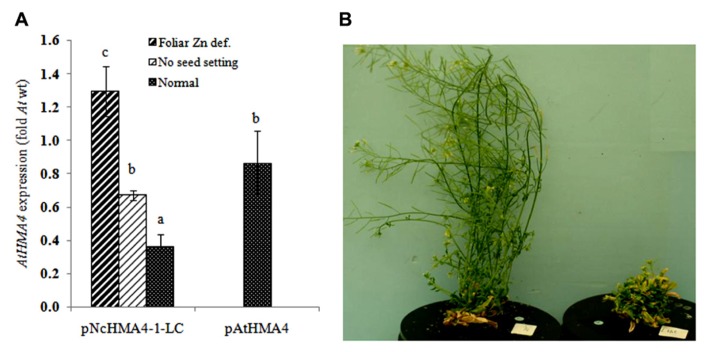
**(A)*** HMA4* expression in the root of transgenic *Arabidopsis hma2hma4* double mutant plants (fold expression relative to *At* wild-type). **(B)** *Arabidopsis hma2hma4* double mutant transformed by *pNcHMA4-1-LC::AtHMA4*, on the left a plant expressing *AtHMA4* at a low level and on the right one with high *AtHMA4* expression.

All primary transgenic *hma2hma4* double mutant plants expressing *NcHMA4* or *AtHMA4* cDNAs under the *AtHMA4* promoter developed normally in a control nutrient solution (2 μM Zn). On the other hand, with few exceptions, the majority of the T_0_ plants expressing *NcHMA4* or *AtHMA4* under any of the *NcHMA4* promoters developed the stunted, curly and chlorotic rosette leaves typical of the *Athma2hma4 *double mutant. These plants were all infertile. In many of them the inflorescences already died before anthesis, which was also observed in case of the double mutant, and in the rest, the siliques remained very small (<4 mm) and completely devoid of seeds. Some plants developed more or less normal leaves and normally sized inflorescences, but with small and seedless siliques. All of these plants, including the untransformed double mutants, were rescued by increasing the Zn concentration in the nutrient solution from 2 to 10 μM. Only two transgenic plants, both transformed with the *pNcHMA4-1-LC::AtHMA4 *construct, developed normally and set seeds at 2 μM Zn. To compare expression levels and phenotypes, we classified all *pNcHMA4-1-LC::AtHMA4* plants, according to their Zn deficiency phenotype, into three categories, i.e., (1) no apparent Zn deficiency symptoms at 2 μM Zn (“normal”), (2) green rosette, but infertile at 2 μM Zn, and (3) stunted and chlorotic rosette and infertile at 2 μM Zn (**Figures 7A,B**). The severity of the Zn deficiency phenotype appeared to be inversely related to the level of *HMA4* expression in the root. The “normal” plants had expression levels of less than half of those of plants expressing *AtHMA4* under the *At* promoter, on average. The green, but infertile, and the chlorotic infertile plants had expression levels close to, and higher than the mean for *pAtHMA4::AtHMA4*-transformed plants, respectively (**Figure 7A**).

### ZN ROOT-TO-SHOOT TRANSLOCATION AND MUTANT COMPLEMENTATION

**FIGURE 8:**
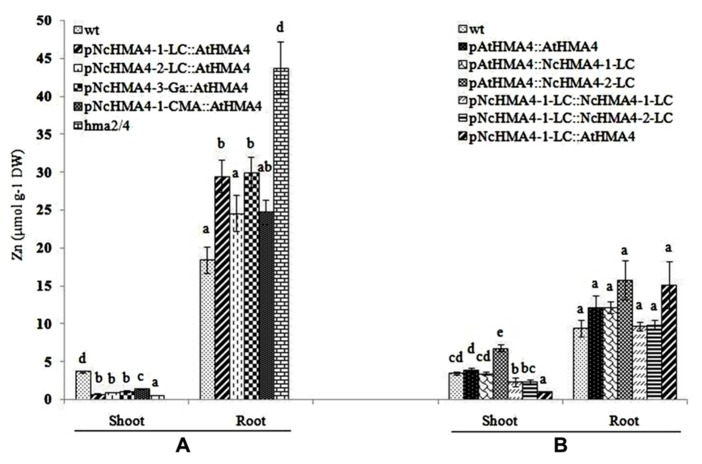
**Shoot Zn concentration (μmol g^-1^ DW) at 2 μM ZnSO_4_ in the nutrient solution in two successive experiments performed with *Arabidopsis* wt, untransformed *hma2hma4* double mutant, and five transgenic lines expressing *AtHMA4* under different *NcHMA4* promoters (A), or six lines expressing three different *HMA4* cDNAs, each under the *AtHMA4* and the *NcHMA4-1-LC* promoter (B).** Significant differences (*P* < 0.05) between means are indicated, separately for both shoots and roots, by different superscripted letters.

We compared Zn root-to-shoot translocation among wild-type and *hma2hma4* double mutant and transgenic plants. In a first experiment we used selected T_1_ transgenic plants (10 plants per concentration) derived from T_0_ plants expressing *AtHMA4* approximately at wild-type levels in the roots, under the *pNcHMA4-1-LC*, *pNcHMA4-2-LC*, *pNcHMA4-3-Ga* and *pNcHMA4-1-CMA*, respectively. After 3 weeks of growth in hydroponics at 2 μM Zn, all of the transgenic lines had foliar Zn concentrations that were slightly, but significantly higher than that of the double mutant, but much lower than that of wild-type plants (**Figure 8A**). The same pattern was observed at 10 μM Zn (data not shown). The root Zn concentrations were highest in the double mutant and lowest in the wild-type, except for the *pNcHMA4-1-CMA::AtHMA4* line at 10 μM Zn (data not shown). All the transgenic lines and the double mutant showed symptoms of foliar Zn deficiency.

In a second experiment we compared selected T_1_ derived from T_0_ plants transformed with *pAtHMA4::AtHMA4*, *pAtHMA4::NcHMA4-1-LC*, *pAtHMA4::NcHMA4-2-LC*, *pNcHM*A4-1-LC::NcHMA4-1-LC, *pNcHMA4-1-LC::NcHMA4-2-LC*, and *pNcHMA4-1-LC::AtHMA4*, using the wild-type as a reference. For this experiment we chose the T_1_ progenies derived from T_0_ plants expressing *NcHMA4-LC* 5- to 10-fold higher than wild-type in the roots. The two *AtHMA4* expressing lines were derived from T_0_ plants with approximately wild-type expression levels in their roots. The lines with the constructs containing the *AtHMA4* promoter had wild-type foliar Zn concentrations, or even higher in the case of the *pAtHMA4::NcHMA4-2-LC* line, which was derived from a T_0_ plant with a particularly high root expression level (six times *At* wild-type level, in comparison with 1.5 and 4 times for the T_0_ parents of the other lines; **Figure 8B**). The lines with the constructs containing the *pNcHMA4-1-LC* exhibited foliar Zn concentrations that were significantly lower than *At* wild-type level. The two lines derived from the T_0_ plants with the highest root expression levels (9 and 10 times *At* wild-type level for *pNcHMA4-1-LC::NcHMA4-1-LC*, *pNcHMA4-1-LC::NcHMA4-2-LC*) showed much higher foliar Zn concentrations, i.e., more than half of the wild-type level, than the one derived from the T_0_ with the lowest root expression level (1.5 times for* pNcHMA4-1-LC::AtHMA4*), i.e., about 25% of wild-type, comparable to the former experiment (**Figure 8A**). As expected, in the* pNcHMA4-1-LC::AtHMA4 *line the root Zn concentration was higher than in *At* wild-type, but this was also the case for the *pAtHMA4::NcHMA4-2-LC* line (**Figure 8B**). All the lines transformed with the* pNcHMA4-1-LC*, as well as the double mutant, showed symptoms of foliar Zn deficiency, whereas all the lines transformed with the *At* promoter did not.

**FIGURE 9:**
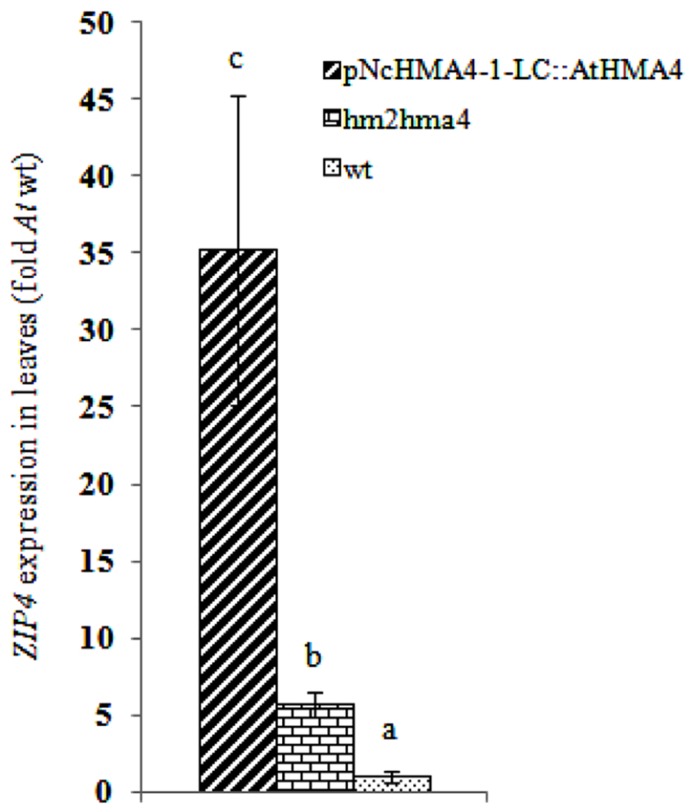
**Average *ZIP4* expression in leaves in transgenic lines expressing *AtHMA4* under the* pNcHMA4-1-LC*, the *hma2hma4 *double**mutant, and wild-type (fold expression relative to *Arabidopsis* wt)**. Significant differences (*P* < 0.05) between means are indicated by different superscripted letters.

To estimate the degree of foliar Zn deficiency in the lines transformed with the* pNcHMA4-1-LC::HMA4* construct, we measured the foliar expression level of a Zn deficiency sensitive marker gene, *ZIP4* (Assunçã et al; 2010), in an* pNcHMA4-1-LC::AtHMA4* T_1 _line, the *hma2hma4* double mutant, and the wild-type. *ZIP4* was five fold higher expressed in the double mutant, and even 35-fold higher in the transgenic line, in comparison with wild-type, showing that expression of *HMA4* under the *pNcHMA4-1-LC* aggravates, rather than alleviates the foliar Zn deficiency of the double mutant (**Figure 9**).

### Cd TOLERANCE AND ACCUMULATION

**FIGURE 10:**
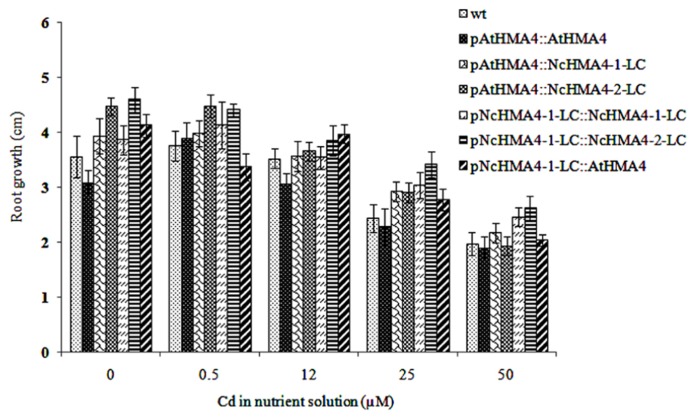
**Effect of Cd on root growth in *Arabidopsis* wt and transgenic lines**. Root length increments were measured after 4 days.

**FIGURE 11:**
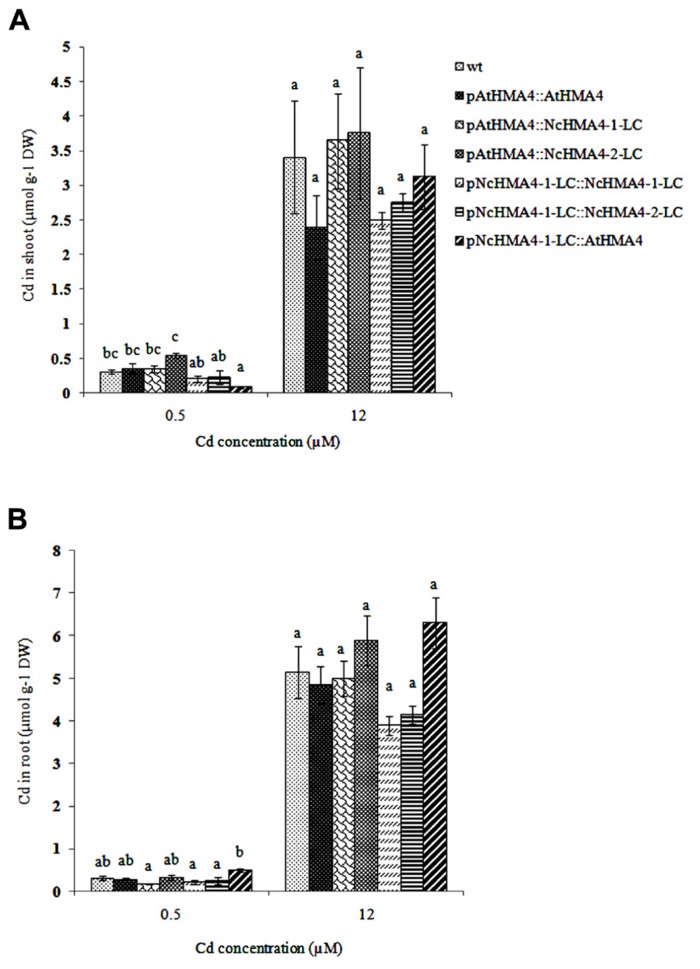
**Cd concentration in *Arabidopsis* wt and transgenic lines (μmol g^**-1**^ DW) exposed to 0. 5 and 12 μM Cd.** **(A)** shoot, **(B)** root. Significant differences (*P* < 0.05) between means are indicated, separately for both treatments, by different superscripted letters.

Cd tolerance, estimated from the root growth response, was tested in the same set of T_1_ lines that was used in the second Zn translocation experiment. Although there was significant variation in root length increments among the lines, the lines by Cd interaction was insignificant, showing that the lines did not significantly differ in their response to Cd (**Figure 10**). The Cd concentrations in shoot and root at the 0.5- and 12-μM Cd exposure levels are given in **Figures 11A,B**. At the 0.5-μM exposure level the lines transformed with the *pAtHMA4* exhibited wild-type-like or higher foliar Cd concentrations, whereas the lines transformed with *pNcHMA4-*1-LC exhibited significantly lower concentrations, in comparison with wild-type. In the roots the Cd concentrations were higher in wild-type than in the *pNcHMA4-1-LC::NcHMA4-1-LC* and *pNcHMA4-1-LC::NcHMA4-2-LC* lines. Overall, the patterns of variation in the Cd concentrations found at the 0.5-μM exposure level were essentially the same as found for Zn among the same set of lines (see above). At the 12-μM exposure level, however, there were no considerable differences in the root and shoot Cd concentrations between the lines and wild-type, or among the lines (**Figures 11A,B**).

## DISCUSSION

Our results clearly confirm that *HMA4* is strongly over-expressed, both in roots and in shoots, in the Zn/Cd/Ni hyperaccumulator *N. caerulescens*, in comparison with the non-hyperaccumulator *A. thaliana. *Like in the other Zn/Cd hyperaccumulator model, *A. halleri*, this over-expression must be due to a combination of copy number expansion and altered *cis*-regulation of the individual copies (Hanikenne et al., 2008). The copy number seems to vary between accessions in *N. caerulescens*, ranging from 2 to 4 (Ó Lochlainn et al., 2011; Craciun et al., 2012). The three copies that we found in the LC library matches with the three copies established in the nearby accession from Prayon (Craciun et al., 2012).

The most striking result that we obtained is that expressing either *AtHMA4* or *NcHMA4* cDNAs under any *N. caerulescens* *HMA4* promoter did not fully complement, except for two T_1_ plants, the Zn-translocation-deficient *A. thaliana hma2hma4* double mutant, whereas expression under the endogenous *AtHMA4* promoter always did. This is not simply a matter of the degree of expression of the transgene in the root. Admittedly, the *HMA4* expression levels produced by *Nc* promoters in the double mutant background were surprisingly low, in some cases even significantly lower than the wild-type expression level in *A. thaliana*. However, when compared in lines derived from plants with equal transgene expression levels in the roots, *pAtHMA4::HMA4* constructs completely restored the Zn root-to-shoot translocation to wild-type level or higher, whereas *pNcHMA4::HMA4* constructs only slightly enhanced the foliar Zn concentration. Even in *pNcHMA4::HMA4* lines expressing the transgene about 10 times higher than wild-type in the root, the foliar Zn concentrations were not higher than 60% of the wild-type level, whereas a strongly expressing *pAtHMA4::HMA4* line showed enhanced Zn translocation, in comparison with wild-type (**Figure 8B**). Admittedly, there was considerable variation regarding the root and shoot Zn concentrations between the two successive experiments that we performed (**Figure 8**). We cannot explain this variation, however, it is remarkable that the Zn shoot concentrations and translocation rates of the lines with the *Nc* promoters are consistently intermediate between those in wild-type and in the *hma2hma4* double mutant. The reasons for this are not entirely clear, but ectopic expression under the *Nc* promoters, in terms of tissue-specificity, seems to be the most plausible explanation. As indicated by the GUS assays, the *Nc* promoters are often mainly active in all the cells of the 2-mm apical root segment, rather than in the xylem parenchyma, whereas the *At* promoter is exclusively active in the xylem parenchyma (**Figure 5**), as is the *Nc* promoter tested in the *A. rhizogenes-*transformed *N. caerulescens* roots. One might argue that this could be owing to the short length of the *Nc* promoter sequences that we isolated, i.e., 2100–2600 bp, in comparison with the *At* promoter (4103 bp), which covered the whole upstream intergenic region. However, as shown by the GUS assay with Rhizogenes-transformed *N. caerulescens* roots, the 2135 bp of the *pNcHMA4-1-LC* does produce expression predominantly in the xylem parenchyma in *N. caerulescens* itself (**Figure 6**), suggesting that *A. thaliana* and *N. caerulescens* may have different transcriptional regulators for *HMA4* expression, at least in part.

There seemed to be a degree of positive correlation between the transgene expression levels in the root and the foliar Zn concentrations among the lines transformed with the *Nc* promoters (**Figure 8A**). However, in spite of this, the transgene expression levels in the roots were also positively correlated with the severity of foliar Zn deficiency symptoms, at least among the plants transformed with the *pNcHMA4-1-LC::AtHMA4* construct (**Figure 7**). The most likely explanation for this phenomenon is that the transgene expression levels in roots are more or less correlated with those in shoots (**Figures 3** and **4**). It is remarkable that expression of *AtHMA4* under the *pNcHMA4-1-LC* improved, albeit slightly, the root-to-shoot Zn translocation (**Figure 8B**), but aggravated rather than alleviated the foliar Zn deficiency symptoms in the *hma2hma4* double mutant, as demonstrated by the strongly enhanced *ZIP4* expression level (**Figure 9**). The latter might be attributable to the relatively high level of *HMA4* expression in the leaf mesophyll (**Figure 4**), which would be expected to enhance the efflux of Zn from photosynthetically active cells. This might in turn cause or enhance Zn deficiency in the absence of hyperaccumulator-like uptake and root-to-shoot translocation rates. The high expression of *HMA4* outside the veins in the leaves of mutants transformed with *pNcHMA4::HMA4* constructs might well reflect the natural expression pattern in *N. caerulescens* itself. As demonstrated by Klein et al. (2008), *N. caerulescens* cells in suspension culture accumulate less Zn or Cd than *A. thaliana* cells, presumably due to higher metal efflux rates. It is therefore conceivable that a high-level of *HMA4* expression in the leaf mesophyll in hyperaccumulators could serve to promote the cell-to-cell transport of metals from the vasculature to their final destiny, the large epidermal cells (Küpper and Kochian, 2010).

In the Cd tolerance test, we used only wild-type as a control, since we had insufficient seeds of the *hma2hma4 *double mutant. However, it can be safely assumed that the double mutant, at least when in the wild-type background, does not have a detectable phenotype for Cd tolerance (Hussain et al., 2004; Wong and Cobbett, 2009). In any case, our results clearly show that *HMA4* expression, regardless of the promoter and cDNA source, does not confer Cd tolerance, in comparison to wild-type, to the *hma2hma4* double mutant. This is not surprising, because at the higher toxic Cd exposure levels there were no considerable effects of the transgenes on the root or shoot Cd concentrations (**Figure 11**). At the non-toxic 0.5-μM Cd exposure level, however, there were pronounced and significant effects of the transgenes on the root and shoot Cd concentrations. Overall, these effects were about the same as those on the root and shoot Zn concentrations (**Figure 8**). This, together with the extremely low foliar Cd concentration in the mutant at the 0.5 μM exposure level, confirms that HMA4 can translocate both Zn and Cd in *A. thaliana* (Hussain et al., 2004; Wong and Cobbett, 2009). However, at higher exposure levels, the translocation of Cd, in contrast with that of Zn, is apparently no longer dependent on HMA4, but mediated by other systems with lower affinity.

Comparing our results with those obtained with *HMA4* genes and promoters from the other model Zn/Cd hyperaccumulator, *A. halleri*, reveals several differences. First, the *AhHMA4* promoters produced a much higher GUS expression in *A. thaliana* roots than did the *NcHMA4* promoters in the present study. Second, GUS expression in transgenic *A. thaliana* under the different *AhHMA4* promoters exhibited the correct tissue-specificity pattern, i.e., exclusively in the xylem parenchyma and the root cap (Hanikenne et al., 2008). The reason for the low and different *HMA4* expression under *NcHMA4* promoters that we observed in our study is elusive, but it is well conceivable that it has something to do with the bigger phylogenetic distance between *N. caerulescens* and *A. thaliana*, in comparison with that between the congeneric *A. thaliana* and *A. halleri*.

Finally, the question of whether correct over-expression of *HMA4* can confer hyperaccumulator-like metal translocation rates to non-hyperaccumulator host species remains unanswered. *HMA4* over-expression in *A. thaliana* has been reported to enhance Cd or Zn translocation to the shoot, albeit only to a marginal degree (Verret et al., 2004; Hanikenne et al., 2008). However, in the latter studies *HMA4* has been expressed under the 35S CaMV promoter, which is bound to yield ectopic expression. Barabasz et al. (2012) expressed a *HMA4* copy from *A. halleri* under the endogenous *Ah* promoter in tobacco, but also found no more than a marginal effect on Zn translocation. It is unknown, however, whether the transgene was correctly over-expressed in the latter study. Unfortunately, the *NcHMA4* promoters that we used in the present study did not produce the correct expression pattern in *A. thaliana* roots, which probably explains the inconsiderable effects of the *HMA4* transgenes on Zn and Cd translocation. It is reasonable to expect that this may not apply to the *A. halleri* *HMA4* promoters (see above). However, even in cases where hyperaccumulator promoters would yield a correct hyperaccumulator-like *HMA4* expression pattern, it is doubtful whether this would lead to more than marginal increments of root-to-shoot metal translocation rates. There are good reasons to assume that hyperaccumulator-like translocation rates are not only dependent on HMA4-mediated xylem loading, but also on “upstream mechanisms,” which prevent the retention of metals in root cell vacuoles (Richau et al., 2009; Deinlein et al., 2012).

## Conflict of Interest Statement

The authors declare that the research was conducted in the absence of any commercial or financial relationships that could be construed as a potential conflict of interest.

## Supplementary Material

The Supplementary Material for this article can be found online at http://www.frontiersin.org/Journal/10.3389/fpls.2013.00404/abstract

## Footnotes

1 http://www.ncbi.nlm.nih.gov

2 http://www.arabidopsis.org

3 http://www.ebi.ac.uk/Tools/msa/clustalw2/
