# Supplementary material for: Expression of HMA4 cDNAs of the zinc hyperaccumulator Noccaea caerulescens from endogenous NcHMA4 promoters does not complement the zinc-deficiency phenotype of the Arabidopsis thaliana hma2hma4 double mutant
Source: Front Plant Sci. 2013 Oct 16;4:404. doi: 10.3389/fpls.2013.00404 (PMC3807671; doi:10.3389/fpls.2013.00404)
Supplement: Supplementary file 1 [file DataSheet1.ZIP › 54667_Iqbal_DataSheet1.DOCX]

**Supplementary Information**

**This file includes**

Supplementary Tables S1 to S4

Supplementary Figure S1 to S2

Supplementary Methods

Supplementary Alignments S1 to S3

Supplementary Sequences S1 to S8

**SUPPLEMENTARY TABLES**

| Primer Name | Sequence |
| --- | --- |
| QsiNcAct2F | CGTCGCCATCCAAGCTGTTC |
| QsiNcAct2R | CACCATCACCAGAGTCCAGC |
| QsiNcHMA4F | CGAGAACCGTGATCGTTGTC |
| QsiNcHMA4R | CGTTTGCTTCTAACCTCGCTTG |
| QsiAtAct2fw | ATGTCGCCATCCAAGCTGTTC |
| QsiAtAct2rev | CACCATCACCAGAATCCAGCA |
| qAtHM4fwd | CGCCGTAGTTTCCGGCTTAC |
| qAtHM4rev | AATCGGATAGATACCGGCGG |
| qAtZIP4F | GATCTTCGTCGATGTTCTTTGG |
| qAtZIP4R | TGAGAGGTATGGCTACACCAGCAGC |

**Supplementary Table S1.** Primers used for RT-qPCR

| Genome walk | Primer Name | Sequence |
| --- | --- | --- |
| Nc 1st walk | NcHMA4rev1 | CGTCGAAGTAACTCTTCTGCC |
|  | NcHMA4rev2 | CTCTTCTGCCACTTCTTCTTTGTC |
| LC 2nd walk | LCprom.Rev1 | GCAAAACTTTTATGCTGGGATGC |
|  | LCprom.Rev2 | CCGGATAAGCAGGGCCGGGTC |
| LC 3rd walk | LCprom.Rev3 | GGCTGGAGGAAATTGCATCGCATGAG |
|  | LCprom.Rev4 | GGTGTTTTGATAGGGTTAACATGG |
| LC 4th walk | LCprom.Rev5 | GCATACACAAACACTTATTCAAGG |
|  | LCprom.Rev6 | GTGGAGATGAATAAAGTGGAGAAAG |
| Ga 2nd walk | Gaprom.Rev1 | CCAAATAAGCAGGGCCGGGTC |
|  | Gaprom.Rev2 | GTGGCAATACCGAAAATATATGTG |
| Ga 3rd walk | Gaprom.Rev3 | CCTCCACAGTAATCCTCACGC |
|  | Gaprom.Rev4 | CAGTATTCGATAGAGAGAGAAGGC |
| Ga 4th walk | Gaprom.Rev5 | GGTGTTTTGATAGGGTTAACATGG |
|  | Gaprom.Rev6 | CTATCCAAGATATTTTGTTCACAAAC |
| CMA 2nd walk | CMAprom.Rev1 | CCAAATAAGCAGGGCCGGGTC |
|  | CMAprom.Rev2 | GTGGCAATACCGAAAATATATGTG |
| CMA 3rd walk | CMAprom.Rev3 | CAGTATTCGATAGAGAGAGAAGGC |
|  | CMAprom.Rev4 | GGCTGGAGGAAATTGCATCGCATGAG |
| CMA 4th walk | CMAprom.Rev5 | GGTGTTTTGATAGGGTTAACATGG |
|  | CMAprom.Rev6 | CTATCCAAGATATTTTGTTCACAAAC |

**Supplementary Table S2.** Primers used for genome walking

| **Sequence Name** | **GenBank accession number** | **Sequence Name** | **GenBank accession number** |
| --- | --- | --- | --- |
| *NcACT2-LC* | JQ435780 | *NcHMA4-1-Ga* | JQ904706 |
| *NcACT2-Ga* | JQ435781 | *NcHMA4-2-Ga* | JQ904707 |
| *NcACT2-CMA* | JQ435782 | *NcHMA4-3-Ga* | JQ904708 |
| *NcHMA4-1-LC* | JQ904704 | *NcHMA4-1-CMA* | JQ904709 |
| *NcHMA4-2-LC* | JQ904705 | *NcHMA4-2-CMA* | JQ904710 |

**Supplementary Table S3.**Sequences submitted to GeneBank with their accession numbers

| (A) | *AtHMA4* | *NcHMA4-1-LC* | *NcHMA4-2-LC* | *NcHMA4-1-Ga* | *NcHMA4-2-Ga* | *NcHMA4-3-Ga* | *NcHMA4-1-CMA* | *NcHMA4-2-CMA* | *NcHMA4-1-SLM* | *NcHMA4-2-SLM* | *NcHMA4-3-SLM* | *NcHMA4-4-SLM* | *AhHMA4-1* | *AhHMA4-2* | *AhHMA4-3* |
| --- | --- | --- | --- | --- | --- | --- | --- | --- | --- | --- | --- | --- | --- | --- | --- |
|  |  | Amino acid | | | | | | | | | | | | |  |
| *AtHMA4* |  | 71 | 72 | 71 | 71 | 72 | 72 | 76 | 71 | 72 | 76 | 76 | 86 | 86 | 85 |
| *NcHMA4-1-LC* | 80 |  | 98 | 94 | 94 | 97 | 97 | 94 | 95 | 97 | 94 | 94 | 75 | 75 | 75 |
| *NcHMA4-2-LC* | 80 | 99 |  | 94 | 94 | 98 | 97 | 94 | 95 | 97 | 94 | 94 | 75 | 74 | 74 |
| *NcHMA4-1-Ga* | 80 | 97 | 97 |  | 98 | 94 | 94 | 97 | 97 | 94 | 95 | 96 | 73 | 73 | 73 |
| *NcHMA4-2-Ga* | 81 | 97 | 97 | 99 |  | 95 | 96 | 97 | 97 | 95 | 96 | 96 | 74 | 74 | 74 |
| *NcHMA4-3-Ga* | 81 | 98 | 98 | 97 | 97 |  | 99 | 94 | 95 | 99 | 94 | 94 | 75 | 74 | 74 |
| *NcHMA4-1-CMA* | 81 | 98 | 98 | 97 | 98 | 99 |  | 94 | 95 | 99 | 95 | 94 | 75 | 74 | 74 |
| *NcHMA4-2-CMA* | 85 | 97 | 97 | 98 | 98 | 96 | 96 |  | 99 | 94 | 98 | 98 | 79 | 78 | 79 |
| *NcHMA4-1-SLM* | 81 | 97 | 97 | 99 | 98 | 97 | 97 | 99 |  | 95 | 97 | 98 | 74 | 74 | 74 |
| *NcHMA4-2-SLM* | 81 | 98 | 98 | 97 | 97 | 99 | 99 | 96 | 97 |  | 94 | 94 | 75 | 74 | 75 |
| *NcHMA4-3-SLM* | 85 | 97 | 96 | 98 | 97 | 96 | 97 | 98 | 98 | 96 |  | 99 | 78 | 78 | 78 |
| *NcHMA4-4-SLM* | 85 | 97 | 97 | 98 | 98 | 96 | 96 | 99 | 99 | 96 | 99 |  | 78 | 78 | 78 |
| *AhHMA4-1* | 88 | 82 | 82 | 82 | 81 | 81 | 81 | 85 | 82 | 81 | 85 | 85 |  | 98 | 98 |
| *AhHMA4-2* | 88 | 81 | 82 | 82 | 81 | 81 | 81 | 85 | 82 | 81 | 85 | 85 | 99 |  | 98 |
| *AhHMA4-3* | 88 | 82 | 82 | 82 | 81 | 81 | 81 | 85 | 82 | 81 | 85 | 85 | 99 | 99 |  |
|  |  | Coding sequence | | | | | | | | | | | | |  |

| (B) | *NcHMA4-1-LC* | *NcHMA4-2-LC* | *NcHMA4-3-LC* | *NcHMA4-1-Ga* | *NcHMA4-2-Ga* | *NcHMA4-3-Ga* | *NcHMA4-1-CMA* | *NcHMA4-2-CMA* | *NcHMA4-1-SLM* | *NcHMA4-2-SLM* | *NcHMA4-3-SLM* | *NcHMA4-4-SLM* | *AhHMA4-1* | *AhHMA4-2* | *AhHMA4-3* |
| --- | --- | --- | --- | --- | --- | --- | --- | --- | --- | --- | --- | --- | --- | --- | --- |
|  | Promoter sequences | | | | | | | | | | | | | |  |
| *AtHMA4* | 36 | 36 | 38 | 39 | 38 | 36 | 36 | 37 | 37 | 37 | 39 | 36 | 52 | 37 | 38 |
| *NcHMA4-1-LC* |  | 85 | 78 | 61 | 61 | 69 | 65 | 58 | 70 | 86 | 56 | 69 | 39 | 42 | 41 |
| *NcHMA4-2-LC* |  |  | 92 | 51 | 51 | 69 | 65 | 58 | 60 | 75 | 52 | 60 | 39 | 40 | 40 |
| *NcHMA4-3-LC* |  |  |  | 45 | 45 | 72 | 71 | 64 | 61 | 69 | 58 | 61 | 41 | 40 | 41 |
| *NcHMA4-1-Ga* |  |  |  |  | 96 | 47 | 43 | 36 | 52 | 73 | 41 | 52 | 38 | 38 | 39 |
| *NcHMA4-2-Ga* |  |  |  |  |  | 47 | 43 | 36 | 52 | 73 | 42 | 52 | 38 | 38 | 38 |
| *NcHMA4-3-Ga* |  |  |  |  |  |  | 94 | 86 | 82 | 67 | 58 | 82 | 41 | 43 | 40 |
| *NcHMA4-1-CMA* |  |  |  |  |  |  |  | 88 | 78 | 63 | 57 | 77 | 42 | 40 | 40 |
| *NcHMA4-2-CMA* |  |  |  |  |  |  |  |  | 70 | 56 | 54 | 70 | 41 | 38 | 38 |
| *NcHMA4-1-SLM* |  |  |  |  |  |  |  |  |  | 70 | 59 | 98 | 40 | 40 | 40 |
| *NcHMA4-2-SLM* |  |  |  |  |  |  |  |  |  |  | 51 | 69 | 40 | 41 | 40 |
| *NcHMA4-3-SLM* |  |  |  |  |  |  |  |  |  |  |  | 59 | 41 | 41 | 42 |
| *NcHMA4-4-SLM* |  |  |  |  |  |  |  |  |  |  |  |  | 40 | 40 | 39 |
| *AhHMA4-1* |  |  |  |  |  |  |  |  |  |  |  |  |  | 48 | 47 |
| *AhHMA4-2* |  |  |  |  |  |  |  |  |  |  |  |  |  |  | 81 |

**Supplementary Table S4.** The percentage identity of the *AtHMA4*, *AhHMA4* and *NcHMA4*; A) amino acid and nucleotide basis and B) promoters sequences at the nucleotide level. Comparisons of promoter sequences are based on the first 2,000 bp upstream of the translational start codon of *HMA4*.

**SUPPLEMENTARY FIGURES**


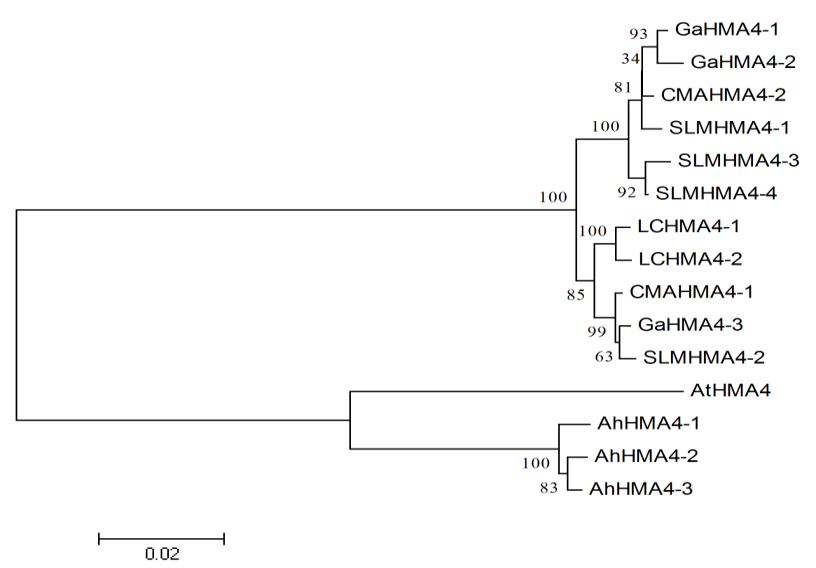


**Supplementary Fig. S1.**The evolutionary history was inferred using the Neighbor-Joining method. The optimal tree with the sum of branch length = 0.29161241 is shown. The percentage of replicate trees in which the associated taxa clustered together in the bootstrap test (1000 replicates) are shown next to the branches. The tree is drawn to scale, with branch lengths in the same units as those of the evolutionary distances used to infer the phylogenetic tree. The evolutionary distances were computed using the Maximum Composite Likelihood method and are in the units of the number of base substitutions per site. All positions containing gaps and missing data were eliminated from the dataset (Complete deletion option). There were a total of 3018 positions in the final dataset. Phylogenetic analyses were conducted in MEGA4. LC = *La Calamine*, SLM = *Saint Laurent Le Minier*, Ga = *Ganges*, Ah = *Arabidopsis halleri*, CMA = *Col du Mas de l’Ayre*, At = *Arabidopsis thaliana*


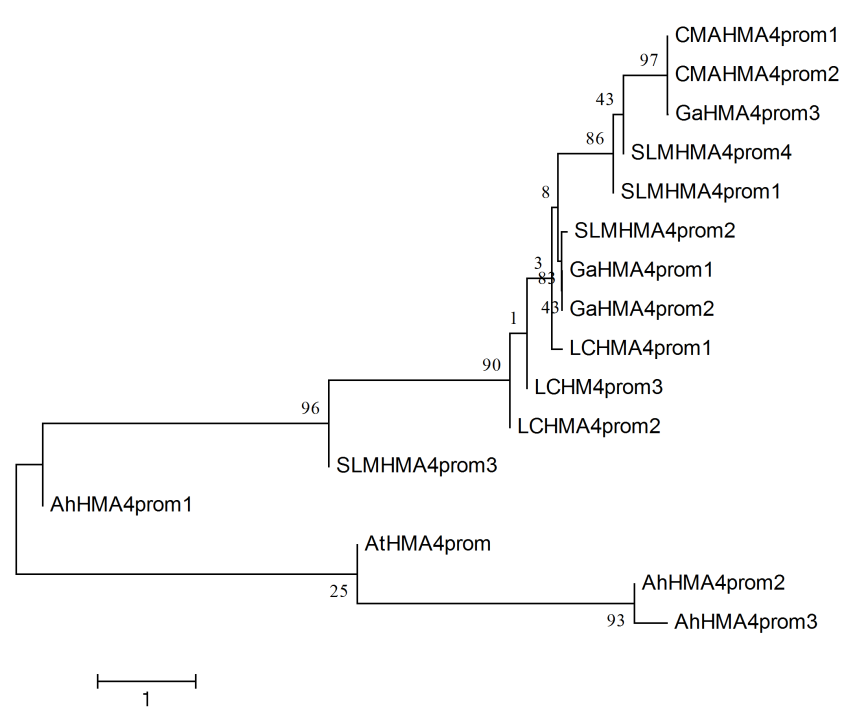


**Supplementary Fig. S2.** The evolutionary history was inferred using the Neighbor-Joining method. The optimal tree with the sum of branch length = 6.70605672 is shown. The percentage of replicate trees in which the associated taxa clustered together in the bootstrap test (1000 replicates) are shown next to the branches. The tree is drawn to scale, with branch lengths in the same units as those of the evolutionary distances used to infer the phylogenetic tree. The evolutionary distances were computed using the Maximum Composite Likelihood method and are in the units of the number of base substitutions per site. All positions containing gaps and missing data were eliminated from the dataset (Complete deletion option). There were a total of 706 positions in the final dataset. Phylogenetic analyses were conducted in MEGA4. This tree was made using 2,000 bp upstream of the translational start codon of HMA4. The sequence accession numbers are AhHMA4p1 and p2: EU382073; AhHMA4p3: EU382072.

**SUPPLEMENTARY METHODS**

**Making Constructs**

To make the constructs for plant transformation, the following primers were used;

***AtHMA4* promoter**, 5’-CACCCAAAACCACTATAGCCTCTTCCTC- 3’ and 5’-TAACGCCATTTTCGGA-GAAGAGAGGAGAGC-3’ for Topo cloning;

***NcHMA4-LC* promoters**, 5’-GGGGACAAGTTTGTACAAAAAAGCAGGCTGCAAGAAGG ACATTGTAA-CTA- 3’ and 5’-GGGGACCACTTTGTACAAGAAAGCTGGGT AACGTCGAAGTAACTCTTCTG- 3’;

***NcHMA4-Ga promoter 3,*** 5’ -GGGGACAAGTTTGTACAAAAAAGCAGGCT TGCCTTGAATAAGTGTTT-GTG- 3’ and 5’-GGGGACCACTTTGTACAAGAAAGCTGGGT AACGTCGAAGTAACTCTTCTG- 3’;

***NcHMA4-Ga promoter 1* and *2,*** 5’-GGGGACAAGTTTGTACAAAAAAGCAGGCT GCGAGGATCATGTGTC-TAAAC-3’ and 5’-GGGGACCACTTTGTACAAGAAAGCTGGGT AACGTCGAAGTAACTCTTCTG- 3’;

***NcHMA4-CMA promoter 1* and *2****,* 5’-GGGGACAAGTTTGTACAAAAAAGCAGGCT CTCCCTCAACAAAGT-GATATC-3’ and 5’-GGGGACCACTTTGTACAAGAAAGCTGGGT AACGTCGAAGTAACTCTTCTG- 3’;

The constructs below have been generated in two steps; (i) The promoter and *HMA4* cDNA were amplified separately. Amplified products were loaded on the gel, then fragments of the right length were cut and purified from the gel. (ii) The purified gel fragments were mixed in optimum concentration and PCR was done using everything except primers. After 10 cycles primers were added to get the fused (promoter + cDNA) products. All the PCR were done using Phusion^®^ High Fidelity DNA Polymerase (Finnzymes) and attB sites of the primers are underlined.

***pAtHMA4::NcHMA4-LC* cDNAs**, 5’ -GGGGACAAGTTTGTACAAAAAAGCAGGCT CACCGCCGGCTCCA-TACTTGC- 3’ and 5’ -TCTGTAACGCCATTTTCGGAGAAGAGAGGAG- 3’ to amplify *pAtHMA4* with overhang at 3’ corresponding to *NcHMA4-LC* cDNAs and 5’- CTCTTCTCCGAAAATGGCGTTACAGAAGGAG- 3’ and 5’-GGGGACCACTTTGTACAAGAAAGCTGGGT TTGAAGACGGAAAGGTTATGC- 3’ to amplify *NcHMA4-LC* with overhang at 5’ corresponding to *pAtHMA4*;

***pNcHMA4-LC::AtHMA4* cDNA**, 5’ -GGGGACAAGTTTGTACAAAAAAGCAGGCT GCAAGAAGGACATTG-TAACTA- 3’ and 5’ -TTTGTTTTGTAACGCCATTTCTGTATC- 3’ to amplify *pNcHMA4-LC* with overhang at 3’ corresponding to *AtHMA4* cDNA, 5’ -CTTTGATACAGAAATGGCGTTACAAAAC- 3’ and 5’ -GGGGACCACTTTGTACAAGAAAGCTGGGT CGGCATTCACGGAATGAGAC- 3’ to amplify *AtHMA4* cDNA with overhang at 5’ corresponding to *pNcHMA4-LC*.

***pNcHMA4-1-LC::NcHMA4-LC* cDNA**, 5’ -GGGGACAAGTTTGTACAAAAAAGCAGGCT GCAAGAAGGAC-ATTGTAACTA- 3’ and 5’-CTCTTCTGCCACTTCTTCTTTGTC- 3’ to amplify *pNcHMA4-1-LC* with overhang at 3’ corresponding to *NcHMA4* cDNA, 5’ -ATGGCGTTACAGAAGGAGATCAAG- 3’ and 5’ -GGGGACCACTTTGTACAAGAAAGCTGGGT TTGAAGACGGAAAGGTTATGC- 3’ to amplify *NcHMA4* cDNA with overhang at 5’ corresponding to *pNcHMA4-1-LC*.

***pNcHMA4-1&2-Ga::AtHMA4* cDNA**, 5’ -GGGGACAAGTTTGTACAAAAAAGCAGGCT GCGAGGATCATG-TGTCTAAAC- 3’ and 5’-TTTGTTTTGTAACGCCATTTCTGTATC- 3’ to amplify *pNcHMA4-1&2-Ga* with overhang at 3’ corresponding to *AtHMA4* cDNA, 5’ -CTTTGATACAGAAATGGCGTTACAAAAC- 3’ and 5’ -GGGGACCACTTTGTACAAGAAAGCTGGGT CGGCATTCACGGAATGAGAC- 3’ to amplify *AtHMA4* cDNA with overhang at 5’ corresponding to *pNcHMA4-1&2-Ga*.

***pNcHMA4-3-Ga::AtHMA4* cDNA**, 5’ -GGGGACAAGTTTGTACAAAAAAGCAGGCT TGCCTTGAATAAGT-GTTTGTG- 3’ and 5’ -TTTGTTTTGTAACGCCATTTCTGTATC- 3’ to amplify *pNcHMA4-3-Ga* with overhang at 3’ corresponding to *AtHMA4* cDNA, 5’ -ATTTTGATACAGAAATGGCGTTACAAAAC- 3’ and 5’ -GGGGACCACTTTGTACAAGAAAGCTGGGT CGGCATTCACGGAATGAGAC- 3’ to amplify *AtHMA4* cDNA with overhang at 5’ corresponding to *pNcHMA4-3-Ga*.

***pNcHMA4-1&2-CMA::AtHMA4* cDNA**, 5’ -GGGGACAAGTTTGTACAAAAAAGCAGGCT CTCCCTCAACA-AAGTGATATC-3’ and 5’ -TTTGTTTTGTAACGCCATTTCTGTATC- 3’ to amplify *pNcHMA4-1-CMA* with overhang at 3’ corresponding to *AtHMA4* cDNA, 5’ -ATTTTGATACAGAAATGGCGTTACAAAAC- 3’ and 5’ -GGGGACCACTTTGTACAAGAAAGCTGGGT CGGCATTCACGGAATGAGAC- 3’ to amplify *AtHMA4* cDNAwith overhang at 5’ corresponding to *pNcHMA4-1-CMA*.

**SUPPLEMENTARY ALIGNMENTS**

AtHMA4 ATGGCGTTACAAAA------------CAAAGAAGAAGAGAAAAAGAAAGTGAAGAAGTTG

NcHMA4-1-LC ATGGCGTTACAGAAGGAGATCAAGAACAAAGAAGAAGATAAAAAGACAAAGAAGAAGTGG

NcHMA4-2-LC ATGGCGTTACAGAAGGAGATCAAGAACAAAGAAGAAGATAAAAAGACAAAGAAGAAGTGG

NcHMA4-1-Ga ATGGCGTTACAGAAGGAGATCAAGAACAAAGAAGAAAATAAAATGACAAAGAAGAAGTGG

NcHMA4-2-Ga ATGGCGTTACAGAAGGAGGACAAGAACAAAGAAGAAAATAAAATGACAAAGAAGACGTGG

NcHMA4-3-Ga ATGGCGTCACAGAAGGAGATCAAGAACAAAGAAGAAGATAAAAAGACAAAGAAGAAGTGG

NcHMA4-1-CMA ATGGCGTCACAGAAGGAGATCAAGAACAAAGAAGAAGATAAAAAGACAAAGAAGAAGTGG

NcHMA4-2-CMA ATGGCGACACAGAAGGAGGACAAGAACAAAGAAGAAAATAAAATGACAAAGAAGAAGTGG

NcHMA4-1-SLM ATGGCGTTACAGAAGGAGGACAAGAACAAAGAAGAAAATAAAATGACAAAGAAGAAGTGG

NcHMA4-2-SLM ATGGCGTTACAGAAGGAGATCAAGAACAAAGAAGAAGATAAAAAGACAAAGAAGAAGTGG

NcHMA4-3-SLM ATGGCGTTACAGAAGGAGGACAAGAACAAAGAAGAAAATAAAATGACAAAGAAGAAGTGG

NcHMA4-4-SLM ATGGCGTTACAGAAGGAGGACAAGAACAAAGAAGAAAATAAAATGACAAAGAAGAAGTGG

AhHMA4-1 ATGGCGTCACAAAA------------CAAAGAAGAAGAGAAAAAGAAAGTGAAGAAGTTG

AhHMA4-2 ATGGCGTCACAAAA------------CAAAGAAGAAGAGAAAAAGAAAGTGAAGAAGTTG

AhHMA4-3 ATGGCGTCACAAAA------------CAAAGAAGAAGAGAAAAAGAAAGTGAAGAAGTTG

AtHMA4 CAAAAGAGTTACTTCGATGTTCTCGGAATCTGTTGTACATCGGAAGTTCCTATAATCGAG

NcHMA4-1-LC CAGAAGAGTTACTTCGACGTTTTGGGAATCTGTTGTACATCGGAGATTCCTCTGATCGAG

NcHMA4-2-LC CAGAAGAGTTACTTCGACGTTTTGGGAATCTGTTGTACATCGGAGATTCCTCTGATCGAG

NcHMA4-1-Ga CAGAAGAGTTACTTCGACGTTTTGGGACTTTGTTGTACATCGGAGATTCCTCTGATCGAG

NcHMA4-2-Ga CAGAAGAGTTACTTCGACGTTTTGGGACTTTGTTGTACATCGGAGATTCCTCTGATCGAG

NcHMA4-3-Ga CAGAAGAGTTACTTCGACGTTTTGGGAATCTGTTGTACATCGGAGATTCCTGTGATCGAG

NcHMA4-1-CMA CAGAAGAGTTACTTCGACGTTTTGGGAATCTGTTGTACATCGGAGATTCCTGTGATCGAG

NcHMA4-2-CMA CAGAAGAGTTACTTCGACGTTTTAGGAATCTGTTGTACATCGGAGATTCCTCTGATCGAG

NcHMA4-1-SLM CAGAAGAGTTACTTCGACGTTTTAGGAATCTGTTGTACATCGGAGATTCCTCTGATCGAG

NcHMA4-2-SLM CAGAAGAGTTACTTCGACGTTTTGGGAATCTGTTGTACATCGGAGATTCCTGTGATCGAG

NcHMA4-3-SLM CAGAAGAGTTACTTCGACGTTTTAGGAATCTGTTGTACATCGGAGATTCCTCTGATCGAG

NcHMA4-4-SLM CAGAAGAGTTACTTCGACGTTTTAGGAATCTGTTGTACATCGGAGATTCCTCTGATCGAG

AhHMA4-1 CAAAAGAGTTACTTCGATGTTCTCGGAATCTGTTGTACATCGGAAGTTCCTATCATCGAG

AhHMA4-2 CAAAAGAGTTACTTCGATGTTCTCGGAATCTGTTGTACATCGGAAGTTCCTATCATCGAG

AhHMA4-3 CAAAAGAGTTACTTCGATGTTCTCGGAATCTGTTGTACATCGGAAGTTCCTATCATCGAG

AtHMA4 AATATTCTCAAGTCACTTGACGGCGTTAAAGAATATTCCGTCATCGTTCCCTCGAGAACC

NcHMA4-1-LC AATATTCTCAAGTCTCTCGACGGCGTTAAGGAATATACCGTCATCGTTCCGTCGAGAACC

NcHMA4-2-LC AATATTCTCAAGTCTCTCGACGGCGTTAAGGAATATACCGTCATCGTTCCGTCGAGAACC

NcHMA4-1-Ga AATATTCTCAAGTCTCTCGACGGCATTAAGGACTATACCATCATCGTTCCGTCGAGAACC

NcHMA4-2-Ga AATATTCTCAAGTCTCTCGACGGCATTAAGGACTATACCATCATCGTTCCGTCGAGAACC

NcHMA4-3-Ga AATATTCTCAAGTCTCTCGACGGCGTTAAGGAATATACCGTCATCGTTCCGTCGAGAACC

NcHMA4-1-CMA AATATTCTCAAGTCTCTCGACGGCGTTAAGGAATATACCGTCATCGTTCCGTCGAGAACC

NcHMA4-2-CMA AATATTCTCAAGTCTCTCGACGGCATTAAGGACTATACCATCATCGTTCCGTCGAGAACC

NcHMA4-1-SLM AATATTCTCAAGTCTCTCGACGGCATTAAGGACTATACCATCATCGTTCCGTCGAGAACC

NcHMA4-2-SLM AATATTCTCAAGTCTCTCGACGGCGTTAAGGAATATACCGTCATCGTTCCGTCGAGAACC

NcHMA4-3-SLM AATATTCTCAAGTCTCTCGACGGCATTAAGGACTATACCATCATCGTTCCGTCGAGAACC

NcHMA4-4-SLM AATATTCTCAAGTCTCTCGACGGCATTAAGGACTATACCATCATCGTTCCGTCGAGAACC

AhHMA4-1 AATATTCTCAAGTCACTTGACGGCGTTAAAGAATATTCCGTCATCGTTCCCTCGAGAACC

AhHMA4-2 AATATTCTCAAGTCACTTGACGGCGTTAAAGAATATTCCGTCATCGTTCCCTCGAGAACC

AhHMA4-3 AATATTCTCAAGTCACTTGACGGCGTTAAAGAATATTCCGTCATCGTTCCCTCGAGAACC

AtHMA4 GTGATTGTTGTTCACGACAGTCTCCTCATCTCTCCCTTCCAAATTGCTAAGGCACTAAAC

NcHMA4-1-LC GTGATCGTTGTCCACGACAGTCTCCTCATCTCCCCGTTCCAAATTGCTAAGGCACTGAAC

NcHMA4-2-LC GTGATCGTTGTCCACGACAGTCTCCTCATCTCCCCGTTCCAAATTGCTAAGGCACTGAAC

NcHMA4-1-Ga GTGATCGTTGTCCACGACAGTCTCCTCATCTCCCCGTTCCAAATTGCTAAGGCACTGAAC

NcHMA4-2-Ga GTGATCGTTGTCCACGACAGTCTCCTCATCTCCCCGTTCCAAATTGCTAAGGCACTGAAC

NcHMA4-3-Ga GTGATCGTTGTCCACGACAGTCTCCTCATCTCCCCGTTCCAAATTGCTAAGGCACTGAAC

NcHMA4-1-CMA GTGATCGTTGTCCACGACAGTCTCCTCATCTCCCCGTTCCAAATTGCTAAGGCACTGAAC

NcHMA4-2-CMA GTGATCGTTGTCCACGACAGTCTCCTCATCTCCCCGTTCCAAATTGCTAAGGCACTGAAC

NcHMA4-1-SLM GTGATCGTTGTCCACGACAGTCTCCTCATCTCCCCGTTCCAAATTGCTAAGGCACTGAAC

NcHMA4-2-SLM GTGATCGTTGTCCACGACAGTCTCCTCATCTCCCCGTTCCAAATTGCCAAGGCACTGAAC

NcHMA4-3-SLM GTGATCGTTGTCCACGACAGTCTCCTCATCTCCCCGTTCCAAATTGCTAAGGCACTGAAC

NcHMA4-4-SLM GTGATCGTTGTCCACGACAGTCTCCTCATCTCCCCGTTCCAAATTGCTAAGGCACTGAAC

AhHMA4-1 GTGATCGTTGTTCACGACAGTCTCCTCATCTCTCCCTTCCAGATTGCTAAGGCATTGAAC

AhHMA4-2 GTGATCGTTGTTCACGACAGCCTCCTCATCTCTCCCTTCCAAATTGCTAAGGCATTGAAC

AhHMA4-3 GTGATCGTTGTTCACGACAGCCTCCTCATCTCTCCCTTCCAAATTGCTAAGGCATTGAAC

AtHMA4 GAAGCTAGGTTAGAAGCAAACGTGAGGGTAAACGGAGAAACTAGCTTCAAGAACAAATGG

NcHMA4-1-LC CAAGCGAGGTTAGAAGCAAACGTGAAAGTAAACGGAGAAACCAGCTTCAAGAATAAATGG

NcHMA4-2-LC CAAGCGAGGTTAGAAGCAAACGTGAAAGTAAACGGAGAAACCAGCTTCAAGAATAAATGG

NcHMA4-1-Ga CAAGCGAGGTTAGAAGCAAACGTGAAAGTAGACGGAGAAACCAGCTTCAAGAATAAATTG

NcHMA4-2-Ga CAAGCGAGGTTAGAAGCAAACGTGAAAGTAGACGGAGAAACCAGCTTCAAGAATAAATTG

NcHMA4-3-Ga CAAGCGAGGTTAGAAGCAAACGTGAAAGTAAACGGAGAAACCAGCTTCAAGAATAAATGG

NcHMA4-1-CMA CAAGCGAGGTTAGAAGCAAACGTGAAAGTAAACGGAGAAACCAGCTTCAAGAATAAATGG

NcHMA4-2-CMA CAAGCGAGGTTAGAAGCAAACGTGAAAGTAGACGGAGAAACCAGCTTCAAGAATAAATTG

NcHMA4-1-SLM CAAGCGAGGTTAGAAGCAAACGTGAAAGTAGACGGAGAAACCAGCTTCAAGAATAAATTG

NcHMA4-2-SLM CAAGCGAGGTTAGAAGCAAACGTGAAAGTAAACGGAGAAACCAGCTTCAAGAATAAATGG

NcHMA4-3-SLM CAAGCGAGGTTAGAAGCAAACGTGAAAGTAGACGGAGAAACCAGCTTCAAGAATAAATTG

NcHMA4-4-SLM CAAGCGAGGTTAGAAGCAAACGTGAAAGTAGACGGAGAAACCAGCTTCAAGAATAAATTG

AhHMA4-1 CAAGCTAGGTTAGAAGCAAACGTGAGAGTAAACGGAGAAACCAACTTCAAGAACAAATGG

AhHMA4-2 CAAGCTAGGTTAGAAGCAAACGTGAGAGTAAACGGAGAAACCAACTTCAAGAACAAATGG

AhHMA4-3 CAAGCTAGGTTAGAAGCAAACGTGAGAGTAAACGGAGAAACAAACTTCAAGAACAAATGG

AtHMA4 CCGAGCCCTTTCGCCGTAGTTTCCGGCTTACTTCTCCTCCTATCCTTCCTAAAGTTTGTC

NcHMA4-1-LC CCGAGCCCTTTCGCGGTGGTTTCCGGCATATTCCTCCTCCTCTCCTTCTTAAAATTTGTA

NcHMA4-2-LC CCGAGCCCTTTCGCGGTGGTTTCCGGCATATTCCTCCTCCTCTCCTTCTTAAAATTTGTA

NcHMA4-1-Ga CCAAGCCCTTTCGCGGTGTTTTCCGGCATATTCCTCCTCCTCTCCTTCTTAAAATTTGTA

NcHMA4-2-Ga CCAAGCCCTTTCGCGGTGTTTTCCGGCATATTCCTCCTCCTCTCCTTCTTAAAATTTGTA

NcHMA4-3-Ga CCAAGCCCTTTCGCGGTGGTTTCCGGCATATTCCTCCTCCTCTCCTTCTTAAAATTTGTA

NcHMA4-1-CMA CCAAGCCCTTTCGCGGTGGTTTCCGGCATATTCCTCCTCCTCTCCTTCTTAAAATTTGTA

NcHMA4-2-CMA CCAAGCCCTTTCGCGGTGTTTTCCGGCATATTCCTCCTCCTCTCCTTCTTAAAATTTGTA

NcHMA4-1-SLM CCAAGCCCTTTCGCGGTGTTTTCCGGCATATTCCTCCTCCTCTCCTTCTTAAAATTTGTA

NcHMA4-2-SLM CCAAGCCCTTTCGCGGTGGTTTCCGGCATATTCCTCCTCCTCTCCTTCTTAAAATTTGTA

NcHMA4-3-SLM CCAAGCCCTTTCGCGGTGTTTTCCGGCATATTCCTCCTCCTCTCCTTCTTAAAATTTGTA

NcHMA4-4-SLM CCAAGCCCTTTCGCGGTGTTTTCCGGCATATTCCTCCTCCTCTCCTTCTTAAAATTTGTA

AhHMA4-1 CCAAGCCCTTTCGCTGTGGTTTCTGGCTTACTTCTCCTCCTCTCCTTCTTAAAGTTTGTC

AhHMA4-2 CCAAGCCCTTTCGCGGTGGTTTCCGGCATACTTCTCCTCCTCTCCTTCTTAAAGTTTGTC

AhHMA4-3 CCAAGCCCTTTCGCTGTGGTTTCCGGCATACTTCTCCTCCTCTCCTTCTTAAAGTTTGTC

AtHMA4 TACTCGCCTTTACGTTGGCTCGCCGTGGCAGCAGTTGCCGCCGGTATCTATCCGATTCTT

NcHMA4-1-LC TACCCACCTCTTCGATGGCTAGCTGTCGTGGGCGTCGCTGCTGGTATTTATCCGATTCTT

NcHMA4-2-LC TACCCACCTCTTCGATGGCTAGCTGTCGTGGGCGTCGCTGCTGGTATTTATCCGATTCTT

NcHMA4-1-Ga TACCCACCTCTTCGATGGCTAGCTGTCGTGGGCGTCGCTACTGGTATTTATCCGATTCTT

NcHMA4-2-Ga TACCCACCTCTTCGATGGCTAGCTGTCGTGGGCGTCGCTACTGGTATTTATCCGATTCTT

NcHMA4-3-Ga TACCCACCTCTTCGATGGCTAGCTGTCGTGGGCGTCGCTGCTGGTATTTATCCGATTCTT

NcHMA4-1-CMA TACCCACCTCTTCGATGGCTAGCTGTCGTGGGCGTCGCTGCTGGTATTTATCCGATTCTT

NcHMA4-2-CMA TACCCACCTCTTCGATGGCTAGCTGTCGTGGGCGTCGCTACTGGTATTTATCCGATTCTT

NcHMA4-1-SLM TACCCACCTCTTCGATGGCTAGCTGTCGTGGGCGTCGCTACTGGTATTTATCCGATTCTT

NcHMA4-2-SLM TACCCACCTCTTCGATGGCTAGCTGTCGTGGGCGTCGCTGCTGGTATTTATCCGATTCTT

NcHMA4-3-SLM TACCCACCTCTTCGATGGCTAGCTGTCGTGGGCGTCGCTACTGGTATTTATCCGATTCTT

NcHMA4-4-SLM TACCCACCTCTTCGATGGCTAGCTGTCGTGGGCGTCGCTACTGGTATTTATCCGATTCTT

AhHMA4-1 TACTCGCCTTTACGTTGGCTCGCGGTCGCAGCAGTTGCCGCCGGTATATATCCGATACTT

AhHMA4-2 TACTCGCCTTTACGCTGGGTTGCAGTCGCAGCAGTTGCCGCCGGTATATATCCGATACTT

AhHMA4-3 TACCCGCCTTTACGCTGGCTTGCAGTCGTAGCAGTTGCCGCCGGTATATATCCGATACTT

AtHMA4 GCCAAAGCCTTTGCTTCCATTAAAAGGCCTAGGATCGACATCAACATATTGGTCATAATA

NcHMA4-1-LC GCAAAAGCCGTCGCTTCTATAAGAAGGCTTAGGGTCGACATCAACATCCTAATCATTATC

NcHMA4-2-LC GCAAAAGCCGTCGCTTCTATAAGAAGGCTTAGGGTCGACATCAACATCCTAATCATTATC

NcHMA4-1-Ga GCAAAATCCGTCGCTTCTATAAGAAGGCTTAGGGTCGACATCAACATCCTAGTCATTATC

NcHMA4-2-Ga GCAAAATCCGTCGCTTCTATAAGAAGGCTTAGGGTCGACATCAACATCCTAGTCATTATC

NcHMA4-3-Ga GCAAAATCCGTCGCTTCTATAAGAAGGCTTAGGGTCGACATCAACATCCTAATCATTATC

NcHMA4-1-CMA GCAAAATCCGTCGCTTCTATAAGAAGGCTTAGGGTCGACATCAACATCCTAATCATTATC

NcHMA4-2-CMA GCAAAATCCGTCGCTTCTATAAGAAGGCTTAGGGTCGACATCAACATCCTAGTCATTATC

NcHMA4-1-SLM GCAAAATCCGTCGCTTCTATAAGAAGGCTTAGGGTCGACATCAACATCCTAGTCATTATC

NcHMA4-2-SLM GCAAAATCCGTCGCTTCTATAAGAAGGCTTAGGGTCGACATCAACATCCTAATCATTATC

NcHMA4-3-SLM GCAAAATCCGTCGCTTCTATAAGAAGGCTTAGGGTCGACATCAACATCCTAGTCATTATC

NcHMA4-4-SLM GCAAAATCCGTCGCTTCTATAAGAAGGCTTAGGGTCGACATCAACATCCTAGTCATTATC

AhHMA4-1 GCCAAAGCCTTTGCTTCCATTAGAAGGCCTAGGATCGACATCAACATATTGGTCATTATA

AhHMA4-2 GCCAAAGCCTTTGCTTCCATTAGAAGGCTTAGGCTCGACATCAACATATTGGTCATTATA

AhHMA4-3 GCCAAAGCCTTTGCTTCCATTAGAAGGCTTAGGCTCGACATCAACATATTGGTCATTATA

AtHMA4 ACCGTGATTGCAACACTTGCAATGCAAGATTTCATGGAGGCAGCAGCAGTTGTGTTCCTA

NcHMA4-1-LC ACAGTGGCTGCAACACTTGCAATGCAAGATTACATGGAGGCTGCAGCAGTTGTCTTCTTA

NcHMA4-2-LC ACAGTGGCTGCAACACTTGCAATGCAAGATTACATGGAGGCTGCAGCAGTTGTCTTCTTA

NcHMA4-1-Ga ACAGTGGCTGCAACACTTGCAATGCAAGATTACATGGAGGCTGCAGCAGTTGTCTTCTTA

NcHMA4-2-Ga ACAGTGGCTGCAACACTTGCAATGCAAGATTACATGGAGGCTGCAGCAGTTGTCTTCTTA

NcHMA4-3-Ga ACAGTGGCTGCAACACTTGCAATGCAAGATTACATGGAGGCTGCAGCAGTTGTCTTCTTA

NcHMA4-1-CMA ACAGTGGCTGCAACACTTGCAATGCAAGATTACATGGAGGCTGCAGCAGTTGTCTTCTTA

NcHMA4-2-CMA ACAGTGGCTGCAACACTTGCAATGCAAGATTACATGGAGGCTGCAGCAGTTGTCTTCTTA

NcHMA4-1-SLM ACAGTGGCTGCAACACTTGCAATGCAAGATTACATGGAGGCTGCAGCAGTTGTCTTCTTA

NcHMA4-2-SLM ACAGTGGCTGCAACACTTGCAATGCAAGATTACATGGAGGCTGCAGCAGTTGTCTTCTTA

NcHMA4-3-SLM ACAGTGGCTGCAACACTTGCAATGCAAGATTACATGGAGGCTGCAGCAGTTGTCTTCTTA

NcHMA4-4-SLM ACAGTGGCTGCAACACTTGCAATGCAAGATTACATGGAGGCTGCAGCAGTTGTCTTCTTA

AhHMA4-1 ACCGTGATAGCAACACTTGCAATGCAAGATTTCATGGAGGCTGCAGCAGTTGTGTTCTTG

AhHMA4-2 ACCGTGATAGCAACACTTGCAATGCAAGATTTCATGGAGGCTGCAGCAGTTGTGTTCTTG

AhHMA4-3 ACCGTGATAGCAACACTTGCAATGCAAGATTTCATGGAGGCTGCAGCAGTTGTGTTCTTG

AtHMA4 TTCACCATATCCGACTGGCTCGAAACAAGAGCTAGCTACAAGGCGACCTCGGTAATGCAG

NcHMA4-1-LC TTCACCATAGCTGACTGGCTGGAAACAAGAGCTAGCTACAAGGCCAACTCGGTGATGCAG

NcHMA4-2-LC TTCACCATAGCTGACTGGCTGGAAACAAGAGCTAGCTACAAGGCCAACTCGGTGATGCAG

NcHMA4-1-Ga TTCACCATAGCTGACTGGCTGGAAACAAGAGCTAGCTACAAGGCGAGCTCGGTGATGCAG

NcHMA4-2-Ga TTCACCATAGCTGACTGGCTGGAAACAAGAGCTAGCTACAAGGCGAGCTCGGTGATGCAG

NcHMA4-3-Ga TTCACCATAGCTGACTGGCTGGAAACAAGAGCTAGCTACAAGGCCAACTCGGTGATGCAG

NcHMA4-1-CMA TTCACCATAGCTGACTGGCTGGAAACAAGAGCTAGCTACAAGGCCAACTCGGTGATGCAG

NcHMA4-2-CMA TTCACCATAGCTGACTGGCTGGAAACAAGAGCTAGCTACAAGGCGAGCTCGGTGATGCAG

NcHMA4-1-SLM TTCACCATAGCTGACTGGCTGGAAACAAGAGCTAGCTACAAGGCGAGCTCGGTGATGCAG

NcHMA4-2-SLM TTCACCATCGCTGACTGGCTGGAAACAAGAGCTAGCTACAAGGCCAACTCGGTGATGCAG

NcHMA4-3-SLM TTCACCATAGCTGACTGGCTGGAAACAAGAGCTAGCTACAAGGCGAGCTCGGTGATGCAG

NcHMA4-4-SLM TTCACCATAGCTGACTGGCTGGAAACAAGAGCTAGCTACAAGGCGAGCTCGGTGATGCAG

AhHMA4-1 TTCACCATAGCCGACTGGCTTGAAACAAGAGCTAGCTACAGGGCGACAGCAGTAATGCAG

AhHMA4-2 TTCACCATAGCCGACTGGCTTGAAACAAGAGCTAGCTACAGGGCGACAGCAGTAATGCAG

AhHMA4-3 TTCACCATAGCCGACTGGCTTGAAACAAGAGCTAGCTACAGGGCGACAGCAGTAATGCAG

AtHMA4 TCTCTGATGAGCTTAGCTCCACAAAAGGCTATAATAGCAGAGACTGGTGAAGAAGTTGAA

NcHMA4-1-LC TCTCTGATGAGCTTAGCTCCACAAAAGGCAGTCATAGCAGAGACTGGAGAAGAAGTTGAA

NcHMA4-2-LC TCTCTGATGAGCTTAGCTCCACAAAAGGCAGTCATAGCAGAGACTGGAGAAGAAGTTGAA

NcHMA4-1-Ga TCTCTGATGAGCTTAGCTCCACAAAAGGCAGTCATAGCAGAGACTGGAGAAGAAGTTGAA

NcHMA4-2-Ga TCTCTGATGAGCTTAGCTCCACAAAAGGCAGTCATAGCAGAGACTGGAGAAGAAGTTGAA

NcHMA4-3-Ga TCTCTGATGAGCTTAGCTCCACAAAAGGCAGTCATAGCAGAGACTGGAGAAGAAGTTGAA

NcHMA4-1-CMA TCTCTGATGAGCTTAGCTCCACAAAAGGCAGTCATAGCAGAGACTGGAGAAGAAGTTGAA

NcHMA4-2-CMA TCTCTGATGAGCTTAGCTCCACAAAAGGCAGTCATAGCAGAGACTGGAGAAGAAGTTGAA

NcHMA4-1-SLM TCTCTGATGAGCTTAGCTCCACAAAAGGCAGTCATAGCAGAGACTGGAGAAGAAGTTGAA

NcHMA4-2-SLM TCTCTGATGAGCTTAGCTCCACAAAAGGCAGTCATAGCAGAGACTGGAGAAGAAGTTGAA

NcHMA4-3-SLM TCTCTGATGAGCTTAGCTCCACAAAAGGCAGTCATAGCAGAGACTGGAGAAGAAGTTGAA

NcHMA4-4-SLM TCTCTGATGAGCTTAGCTCCACAAAAGGCAGTCATAGCAGAGACTGGAGAAGAAGTTGAA

AhHMA4-1 TCTCTGATGAGCTTAGCTCCACAGAAGGCAATAATAGCAGAGACTGGTGAAGAAGTTGAA

AhHMA4-2 TCTCTGATGAGCTTAGCTCCACAGAAGGCAATAATAGCAGAGACTGGTGAAGAAGTTGAA

AhHMA4-3 TCTCTGATGAGCTTAGCTCCACAGAAGGCAATAATAGCAGAGACTGGTGAAGAAGTTGAA

AtHMA4 GTAGATGAGGTTAAGGTTGATACAGTTGTAGCAGTTAAAGCTGGTGAAACCATACCAATT

NcHMA4-1-LC GTAGATGAGGTTCAGCTCAACACAATCATAGCAGTTAAAGCCGGTGAAACCATACCTATT

NcHMA4-2-LC GTAGATGAGGTTCAGCTCAACACAATCATAGCAGTTAAAGCCGGTGAAACCATACCTATT

NcHMA4-1-Ga GTAGATGAGGTTGAGCTCAACACAATCATAGCAGTTAAAGCCGGTGAAACCATACCTATT

NcHMA4-2-Ga GTAGATGAGGTTGAGCTCAACACAATCATAGCAGTTAAAGCCGGTGAAACCATACCTATT

NcHMA4-3-Ga GTAGATGAGGTTCAGCTCAACACAATCATAGCAGTTAAAGCCGGTGAAACCATACCTATT

NcHMA4-1-CMA GTAGATGAGGTTCAGCTCAACACAATCATAGCAGTTAAAGCCGGTGAAACCATACCTATT

NcHMA4-2-CMA GTAGATGAGGTTGAGCTCAACACAATCATAGCAGTTAAAGCCGGTGAAACCATACCTATT

NcHMA4-1-SLM GTAGATGAGGTTGAGCTCAACACAATCATAGCAGTTAAAGCCGGTGAAACCATACCTATT

NcHMA4-2-SLM GTAGATGAGGTTCAGCTCAACACAATCATAGCAGTTAAAGCCGGTGAAACCATACCTATT

NcHMA4-3-SLM GTAGATGAGGTTGAGCTCAACACAATCATAGCAGTTAAAGCCGGTGAAACCATACCTATT

NcHMA4-4-SLM GTAGATGAGGTTGAGCTCAACACAATCATAGCAGTTAAAGCCGGTGAAACCATACCTATT

AhHMA4-1 GTAGATGAGGTTAAGGTTAGCACAGTTGTAGCGGTTAAAGCTGGTGAAACCATTCCAATT

AhHMA4-2 GTAGATGAGGTTAAGGTTAGCACAGTTGTAGCGGTTAAAGCTGGTGAAACCATTCCAATT

AhHMA4-3 GTAGATGAGGTTAAGGTTAGCACAGTTGTAGCGGTTAAAGCTGGTGAAACCATTCCAATT

AtHMA4 GATGGAATTGTGGTGGATGGAAACTGTGAGGTAGACGAGAAAACCTTAACGGGCGAAGCA

NcHMA4-1-LC GATGGAATTGTGGTCGATGGAAACTGTGAAGTAGACGAGAAAACCTTAACCGGTGAAGCA

NcHMA4-2-LC GATGGAATTGTGGTCGATGGAAACTGTGAAGTAGACGAGAAAACCTTAACCGGTGAAGCA

NcHMA4-1-Ga GATGGAATTGTAGTCGATGGGAACTGTGAAGTAGACGAGAAAACCTTAACTGGTGAAGCA

NcHMA4-2-Ga GATGGAATTGTAGTCGATGGAAACTGTGAAGTAGACGAGAAAACCTTAACTGGTGAAGCA

NcHMA4-3-Ga GATGGAATTGTAGTCGATGGAAACTGTGAAGTAGACGAGAAAACCTTAACCGGTGAAGCA

NcHMA4-1-CMA GATGGAATTGTAGTCGATGGGAACTGTGAAGTAGACGAGAAAACCTTAACCGGTGAAGCA

NcHMA4-2-CMA GATGGAATTGTAGTCGATGGGAACTGTGAAGTAGACGAGAAAACCTTAACTGGTGAAGCA

NcHMA4-1-SLM GATGGAATTGTAGTCGATGGAAACTGTGAAGTAGACGAGAAAACCTTAACTGGTGAAGCA

NcHMA4-2-SLM GATGGAATTGTAGTCGATGGAAACTGTGAAGTAGACGAGAAAACCTTAACCGGTGAAGCA

NcHMA4-3-SLM GATGGAATTGTAGTCGATGGAAACTGTGAAGTAGACGAGAAAACCTTAACTGGTGAAGCA

NcHMA4-4-SLM GATGGAATTGTAGTCGATGGAAACTGTGAAGTAGACGAGAAAACCTTAACTGGTGAAGCA

AhHMA4-1 GATGGAATTGTGGTGGATGGTAACTGTGAAGTAGACGAGAAAACCTTAACGGGCGAAGCA

AhHMA4-2 GATGGAATTGTGGTGGATGGTAACTGTGAAGTAGACGAGAAAACCTTAACGGGCGAAGCA

AhHMA4-3 GATGGAATTGTGGTGGATGGTAACTGTGAAGTAGACGAGAAAACCTTAACGGGCGAAGCA

AtHMA4 TTTCCTGTGCCTAAACAGAGAGATTCTACCGTTTGGGCTGGAACCATTAATCTAAATGGT

NcHMA4-1-LC TTTCCTGTGCCTAAACAGAGAGATTCTACGGTTTTGGCTGGAACTATGAATCTAAATGGT

NcHMA4-2-LC TTTCCTGTGCCTAAACAGAGAGATTCTACGGTTTTGGCTGGAACTATGAATCTAAATGGT

NcHMA4-1-Ga TTTCCTGTGCCTAAACAGAGAGATTCTACGGTTTGGGCTGGAACTATTAATCTAAATGGT

NcHMA4-2-Ga TTTCCTGTGCCTAAACAGAGAGATTCTACGGTTTGGGCTGGAACTATTAATCTAAATGGT

NcHMA4-3-Ga TTTCCTGTGCCTAAACAGAGAGATTCTACGGTTTTGGCTGGAACTATGAATCTAAATGGT

NcHMA4-1-CMA TTTCCTGTGCCTAAACAGAGAGATTCTACGGTTTTGGCTGGAACTATGAATCTAAATGGT

NcHMA4-2-CMA TTTCCTGTGCCTAAACAGAGAGATTCTACGGTTTGGGCTGGAACTATTAATCTAAATGGT

NcHMA4-1-SLM TTTCCTGTGCCTAAACAGAGAGATTCTACGGTTTGGGCTGGAACTATTAATCTAAATGGT

NcHMA4-2-SLM TTTCCTGTGCCTAAACAGAGAGATTCTACGGTTTTGGCTGGAACTATTAATCTAAATGGT

NcHMA4-3-SLM TTTCCTGTGCCTAAACAGAGAGATTCTACGGTTTGGGCTGGAACTATTAATCTAAATGGT

NcHMA4-4-SLM TTTCCTGTGCCTAAACAGAGAGATTCTACGGTTTGGGCTGGAACTATTAATCTAAATGGT

AhHMA4-1 TTTCCTGTGCCGAAACAGAAAGATTCTTCGGTTTGGGCTGGAACCATCAATCTAAATGGT

AhHMA4-2 TTTCCTGTGCCGAAACAGAAAGATTCTTCGGTTTGGGCTGGAACCATCAACCTAAATGGT

AhHMA4-3 TTTCCTGTGCCGAAACAGAAAGATTCTTCGGTTTGGGCTGGAACCATCAATCTAAATGGT

AtHMA4 TACATATGTGTGAAAACAACTTCTTTAGCGGGTGATTGTGTGGTTGCGAAAATGGCTAAG

NcHMA4-1-LC TATATAAGTGTGAACACAACTGCTTTAGCTAGTGATTGCGTGGTTGCAAAGATGGCTAAG

NcHMA4-2-LC TATATAAGTGTGAACACAACTGCTTTAGCTAGTGATTGCGTGGTTGCAAAGATGGCTAAG

NcHMA4-1-Ga TATATAAGTGTGAACACAACTGCTTTAGCTAGTGATTGTGTGGTTGCAAAGATGGCTAAG

NcHMA4-2-Ga TATATAAGTGTGAACACAACTGCTTTAGCTAGTGATTGTGTGGTTGCAAAGATGGCTAAG

NcHMA4-3-Ga TATATAAGTGTGAACACAACTGCTTTAGCTAGTGATTGTGTGGTTGCAAAGATGGCTAAG

NcHMA4-1-CMA TATATAAGTGTGAACACAACTGCTTTAGCTAGTGATTGTGTGGTTGCAAAGATGGCTAAG

NcHMA4-2-CMA TATATAAGTGTGAACACAACTGCTTTAGCTAGTGATTGTGTGGTTGCAAAGATGGCTAAG

NcHMA4-1-SLM TATATAAGTGTGAACACAACTGCTTTAGCTAGTGATTGTGTGGTTGCAAAGATGGCTAAG

NcHMA4-2-SLM TATATAAGTGTGAACACAACTGCTTTAGCTAGTGATTGTGTGGTTGCAAAGATGGCTAAG

NcHMA4-3-SLM TATATAAGTGTGAACACAACTGCTTTAGCTAGTGATTGTGTGGTTGCAAAGATGGCTAAG

NcHMA4-4-SLM TATATAAGTGTGAACACAACTGCTTTAGCTAGTGATTGTGTGGTTGCAAAGATGGCTAAG

AhHMA4-1 TACATAAGTGTGAAAACAACTTCTTTAGCGGGTGATTGCGTGGTTGCGAAGATGGCTAAG

AhHMA4-2 TACATAAGTGTGAAAACAACTTCTTTAGCGGGTGATTGCGTGGTTGCGAAGATGGCTAAG

AhHMA4-3 TACATAAGTGTGAAAACAACTTCTTTAGCGGGTGATTGCGTGGTTGCGAAGATGGCTAAG

AtHMA4 CTAGTAGAAGAAGCTCAGAGCAGTAAAACCAAATCTCAGAGACTAATAGACAAATGTTCT

NcHMA4-1-LC CTCGTAGAAGAAGCTCAGGGCAGTAAAACCAAATCTCAGAGACTAATAGACAAATGTTCT

NcHMA4-2-LC CTCGTAGAAGAAGCTCAGGGCAGTAAAACCAAATCTCAGAGACTAATAGACAAATGTTCT

NcHMA4-1-Ga CTCGTAGAAGAAGCTCAGAGCAGTAAAACCAAATCTCAGAGACTAATAGACAAATATTCT

NcHMA4-2-Ga CTCGTAGAAGAAGCTCAGAGCAGTAAAACCAAATCTCAGAGACTAATAGACAAATATTCT

NcHMA4-3-Ga CTCGTAGAAGAAGCTCAGAGCAGTAAAACCAAATCTCAGAGACTAATAGACAAATGTTCT

NcHMA4-1-CMA CTCGTAGAAGAAGCTCAGAGCAGTAAAACCAAATCTCAGAGACTAATAGACAAATGTTCT

NcHMA4-2-CMA CTCGTAGAAGAAGCTCAGAGCAGTAAAACCAAATCTCAGAGACTAATAGACAAATATTCT

NcHMA4-1-SLM CTCGTAGAAGAAGCTCAGAGCAGTAAAACCAAATCTCAGAGACTAATAGACAAATATTCT

NcHMA4-2-SLM CTCGTAGAAGAAGCTCAGAGCAGTAAAACCAAATCTCAGAGACTAATAGACAAATGTTCT

NcHMA4-3-SLM CTCGTAGAAGAAGCTCAGAGCAGTAAAACCAAATCTCAGAGACTAATAGACAAATATTCT

NcHMA4-4-SLM CTCGTAGAAGAAGCTCAGAGCAGTAAAACCAAATCTCAGAGACTAATAGACAAATATTCT

AhHMA4-1 CTAGTAGAAGAAGCTCAGAGCAGTAAAACCAAATCCCAAAGACTAATAGACAAATGTTCT

AhHMA4-2 CTAGTAGAAGAAGCTCAGAGCAGTAAAACCAAATCCCAAAGACTAATAGACAAATGTTCT

AhHMA4-3 CTAGTAGAAGAAGCTCAGAGCAGTAAAACCAAATCCCAAAGACTAATAGACAAATGTTCT

AtHMA4 CAGTACTATACTCCAGCAATCATCTTAGTATCAGCTTGCGTTGCCATTGTCCCGGTTATA

NcHMA4-1-LC CAGTACTATACTCCAGCAATCATCATAATATCGGCTGGCTTTGCGATTGTCCCGGCTATA

NcHMA4-2-LC CAGTACTATACTCCAGCAATCATCATAATATCGGCTGGCTTTGCGATTGTCCCGGCTATA

NcHMA4-1-Ga CAGTACTATACTCCAGCAATCATCATAATATCGGCTGGCTTTGCGATTGTCCCGCTTATA

NcHMA4-2-Ga CAGTACTATACTCCAGCAATCATCATAATATCGGCTGGCTTTGCGATTGTCCCGCTTATA

NcHMA4-3-Ga CAGTACTATACTCCAGCAATCATCATAATATCGGCTGGCTTTGCGATTGTCCCGGCTATA

NcHMA4-1-CMA CAGTACTATACTCCAGCAATCATCATAATATCGGCTGGCTTTGCGATTGTCCCGGCTATA

NcHMA4-2-CMA CAGTACTATACTCCAGCAATCATCATAATATCGGCTGGCTTTGCGATTGTCCCGCTTATA

NcHMA4-1-SLM CAGTACTATACTCCAGCAATCATCATAATATCGGCTGGCTTTGCAATTGTCCCGGCTATA

NcHMA4-2-SLM CAGTACTATACTCCAGCAATCATCATAATATCGGCTGGCTTTGCGATTGTCCCGGCTATA

NcHMA4-3-SLM CAGTACTATACTCCAGCAATCATCATAATATCGGCTGGCTTTGCAATTGTCCCGGCTATA

NcHMA4-4-SLM CAGTACTATACTCCAGCAATCATCATAATATCGGCTGGCTTTGCAATTGTCCCGGCTATA

AhHMA4-1 CAGTACTATACTCCAGCGATCATCGTAGTATCAGCTTGCGTCGCCATTGTCCCGGTTATT

AhHMA4-2 CAGTACTATACTCCAGCGATCATCGTAGTATCAGCTTGCGTCGCCATTGTCCCGGTTATT

AhHMA4-3 CAGTACTATACTCCAGCGATCATCGTAGTATCAGCTTGCGTCGCCATTGTCCCGGTTATT

AtHMA4 ATGAAGGTCCACAACCTTAAACATTGGTTCCACCTAGCATTAGTTGTGTTAGTCAGTGGT

NcHMA4-1-LC ATGAAAGTTCACAACCTCAACCATTGGTTTCATTTAGCACTGGTTGTGTTAGTCAGTGCT

NcHMA4-2-LC ATGAAAGTTCACAACCTCAACCATTGGTTTCATTTAGCACTGGTTGTGTTAGTCAGTGCT

NcHMA4-1-Ga ATGAAAGTTCGCAACCTCAACCATTGGTTTCATTTAGCACTGGTTGTGTTAGTCAGTGCT

NcHMA4-2-Ga ATGAAAGTTCGCAACCTCAACCATTGGTTTCATTTAGCACTGGTTGTGTTAGTCAGTGCT

NcHMA4-3-Ga ATGAAAGTTCGCAACCTCAACCATTGGTTTCATTTAGCACTGGTTGTGTTAGTCAGTGCT

NcHMA4-1-CMA ATGAAAGTTCGCAACCTCAACCATTGGTTTCATTTAGCACTGGTTGTGTTAGTCAGTGCT

NcHMA4-2-CMA ATGAAAGTTCGCAACCTCAACCATTGGTTTCATTTAGCACTGGTTGTGTTAGTCAGTGCT

NcHMA4-1-SLM ATGAAAGTTCGCAACCTCAACCATTGGTTTCATTTAGCACTGGTTGTGTTAGTCAGTGCT

NcHMA4-2-SLM ATGAAAGTTCGCAACCTCAACCATTGGTTTCATTTAGCACTGGTTGTGTTAGTCAGTGCT

NcHMA4-3-SLM ATGAAAGTTCGCAACCTCAACCATTGGTTTCATTTAGCACTGGTTGTGTTAGTCAGTGCT

NcHMA4-4-SLM ATGAAAGTTCGCAACCTCAACCATTGGTTTCATTTAGCACTGGTTGTGTTAGTCAGTGCT

AhHMA4-1 ATGAAGGTCCACAACCTTAAACATTGGTTCCACCTAGCATTAGTTGTGTTAGTCAGTGGC

AhHMA4-2 ATGAAGGTCCACAACCTTAAACATTGGTTCCACCTAGCATTAGTTGTGTTAGTCAGTGGC

AhHMA4-3 ATGAAGGTCCACAACCTTAAACATTGGTTCCACCTAGCATTAGTTGTGTTAGTCAGTGGC

AtHMA4 TGTCCCTGTGGTCTTATCCTCTCTACACCAGTTGCTACTTTCTGTGCACTTACTAAAGCG

NcHMA4-1-LC TGTCCCTGTGGTCTTATCCTCTCTACACCAGTAGCTACATTCTGTGCACTTACTAAAGCG

NcHMA4-2-LC TGTCCCTGTGGTCTTATCCTCTCTACACCAGTAGCTACATTCTGTGCACTTACTAAAGCG

NcHMA4-1-Ga TGTCCCTGTGGTCTTATCCTCTCTACACCAGTAGCTACATTCTGTGCACTTACTAAAGCG

NcHMA4-2-Ga TGTCCCTGTGGTCTTATCCTCTCTACACCAGTAGCTACATTCTGTGCACTTACTAAAGCG

NcHMA4-3-Ga TGTCCCTGTGGTCTTATCCTCTCTACACCAGTAGCTACATTCTGTGCACTTACTAAAGCG

NcHMA4-1-CMA TGTCCCTGTGGTCTTATCCTCTCTACACCAGTAGCTACATTCTGTGCACTTACTAAAGCG

NcHMA4-2-CMA TGTCCCTGTGGTCTTATCCTCTCTACACCAGTAGCTACATTCTGTGCACTTACTAAAGCG

NcHMA4-1-SLM TGTCCCTGTGGTCTTATCCTCTCTACACCAGTAGCTACATTCTGTGCACTTACTAAAGCG

NcHMA4-2-SLM TGTCCCTGTGGTCTTATCCTCTCTACACCAGTAGCTACATTCTGTGCACTTACTAAAGCG

NcHMA4-3-SLM TGTCCCTGTGGTCTTATCCTCTCTACACCAGTAGCTACATTCTGTGCACTTACTAAAGCG

NcHMA4-4-SLM TGTCCCTGTGGTCTTATCCTCTCTACACCAGTAGCTACATTCTGTGCACTTACTAAAGCG

AhHMA4-1 TGTCCCTGTGGTCTTATCCTCTCTACACCAGTTGCTACTTTCTGTGCACTTACTAAAGCG

AhHMA4-2 TGTCCCTGTGGTCTTATCCTCTCTACACCAGTTGCTACTTTCTGTGCACTTACTAAAGCG

AhHMA4-3 TGTCCCTGTGGTCTTATCCTCTCTACACCAGTTGCTACTTTCTGTGCACTTACTAAAGCG

AtHMA4 GCAACTTCAGGGCTTCTGATCAAAAGTGCTGATTATCTTGACACACTCTCAAAGATCAAG

NcHMA4-1-LC GCAACTTCAGGGCTTCTGATCAAAAGTGCTGATTATCTTGACACTCTTTCAAAGATCAAA

NcHMA4-2-LC GCAACTTCAGGGCTTCTGATCAAAAGTGCTGATTATCTTGACACTCTTTCAAAGATCAAA

NcHMA4-1-Ga GCAACTTCAGGGCTTCTGATCAAAAGTGCTGATTATCTTGACACTCTTTCAAAGATCAAG

NcHMA4-2-Ga GCAACTTCAGGGCTTCTGATCAAAAGTGCTGATTATCTTGACACTCTTTCAAAGATCAAG

NcHMA4-3-Ga GCAACTTCAGGGCTTCTGATCAAAAGTGCTGACTATCTTGACACTCTTTCAAAGATCAAG

NcHMA4-1-CMA GCAACTTCAGGGCTTCTGATCAAAAGTGCTGATTATCTTGACACTCTTTCAAAGATCAAG

NcHMA4-2-CMA GCAACTTCAGGGCTTCTGATCAAAAGTGCTGATTATCTTGACACTCTTTCAAAGATCAAG

NcHMA4-1-SLM GCAACTTCAGGGCTTCTGATCAAAAGTGCTGATTATCTTGACACTCTTTCAAAGATCAAG

NcHMA4-2-SLM GCAACTTCAGGGCTTCTGATCAAAAGTGCTGATTATCTTGACACTCTTTCAAAGATCAAG

NcHMA4-3-SLM GCAACTTCAGGGCTTCTGATCAAAAGTGCTGATTATCTTGACACTCTTTCAAAGATCAAG

NcHMA4-4-SLM GCAACTTCAGGGCTTCTGATCAAAAGTGCTGATTATCTTGACACTCTTTCAAAGATCAAG

AhHMA4-1 GCTACTTCAGGGCTTCTGATCAAAAGTGCTGATTATCTTGACACTCTCTCAAAGATCAAA

AhHMA4-2 GCTACTTCAGGGCTTCTGATCAAAAGTGCTGATTATCTTGACACTCTCTCAAAGATCAAA

AhHMA4-3 GCTACTTCAGGGCTTCTGATCAAAAGTGCTGATTATCTTGACACTCTCTCAAAGATCAAA

AtHMA4 ATTGTTGCTTTCGATAAAACTGGGACTATTACAAGAGGAGAGTTCATTGTCATAGATTTC

NcHMA4-1-LC ATCGCTGCTTTTGACAAAACCGGAACTATCACTAGAGGAGAGTTCATTGTCATAGAATTC

NcHMA4-2-LC ATCGCTGCTTTTGACAAAACCGGAACTATCACTAGAGGAGAGTTCATTGTCATAGAATTC

NcHMA4-1-Ga ATCGCTGCTTTTGACAAAACCGGAACTATCACTAGAGGAGAGTTCATTGTCATAGAATTC

NcHMA4-2-Ga ATCGCTGCTTTTGACAAAACCGGAACTATCACTAGAGGAGAGTTCATTGTCATAGAATTC

NcHMA4-3-Ga ATCGCTGCTTTTGACAAAACCGGAACTATCACTAGAGGAGAGTTCATTGTCATAGAATTC

NcHMA4-1-CMA ATCGCTGCTTTTGACAAAACCGGAACTATCACTAGAGGAGAGTTCATTGTCATAGAATTC

NcHMA4-2-CMA ATCGCTGCTTTTGACAAAACCGGAACTATCACTAGAGGAGAGTTCATTGTCATAGAATTC

NcHMA4-1-SLM ATCGCTGCTTTTGACAAAACCGGAACTATCACTAGAGGAGAGTTCATTGTCATAGAATTC

NcHMA4-2-SLM ATCGCTGCTTTTGACAAAACCGGAACTATCACTAGAGGAGAGTTCATTGTCATAGAATTC

NcHMA4-3-SLM ATCGCTGCTTTTGACAAAACCGGAACTATCACTAGAGGAGAGTTCATTGTCATAGAATTC

NcHMA4-4-SLM ATCGCTGCTTTTGACAAAACCGGAACTATCACTAGAGGAGAGTTCATTGTCATAGAATTC

AhHMA4-1 ATCGCTGCTTTCGACAAAACCGGGACTATTACCAGAGGAGAGTTCATTGTCATAGATTTC

AhHMA4-2 ATCGCTGCTTTCGACAAAACCGGGACTATTACCAGAGGAGAGTTCATTGTCATAGATTTC

AhHMA4-3 ATCGCTGCTTTCGACAAAACCGGGACTATTACCAGAGGAGAGTTCATTGTCATAGATTTC

AtHMA4 AAGTCACTCTCTAGAGATATAAACCTACGCAGCTTGCTTTACTGGGTATCAAGTGTTGAA

NcHMA4-1-LC AAGTCACTCTCTAGAGACATAAGCCTACGCAGCTTGCTTTACTGGGTGTCAAGTGTTGAA

NcHMA4-2-LC AAGTCACTCTCTAGAGACATAAGCCTACGCAGCTTGCTTTACTGGGTGTCAAGTGTTGAA

NcHMA4-1-Ga AAGTCACTCTCTAGAGACATAAGCCTAAGCAGCTTGCTTTACTGGGTATCAAGTGTTGAA

NcHMA4-2-Ga AAGTCACTCTCTAGAGACATAAGCCTAAGCAGCTTGCTTTACTGGGTATCAAGTGTTGAA

NcHMA4-3-Ga AAGTCACTCTCTAGAGACATAAGCCTACGCAGCTTGCTTTACTGGGTGTCAAGTGTTGAA

NcHMA4-1-CMA AAGTCACTCTCTAGAGACATAAGCCTACGCAGCTTGCTTTACTGGGTGTCAAGTGTTGAA

NcHMA4-2-CMA AAGTCACTCTCTAGAGACATAAGCCTAAGCAGCTTGCTTTACTGGGTATCAAGTGTTGAA

NcHMA4-1-SLM AAGTCACTCTCTAGAGACATAAGCCTAAGCAGCTTGCTTTACTGGGTATCAAGTGTTGAA

NcHMA4-2-SLM AAGTCACTCTCTAGAGACATAAGCCTACGCAGCTTGCTTTACTGGGTATCAAGTGTTGAA

NcHMA4-3-SLM AAGTCACTCTCTAGAGACATTAGCCTACGCAGCTTGCTTTACTGGGTATCAAGTGTTGAA

NcHMA4-4-SLM AAGTCACTCTCTAGAGACATAAGCCTAAGCAGCTTGCTTTACTGG-TATCAAGTGTTGAA

AhHMA4-1 AAGTCACTCTCTAGAGATATAAGCCTACGTAGCTTGCTTTACTGGGTATCAAGTGTCGAA

AhHMA4-2 AAGTCACTCTCTAGAGATATAAGCCTACGTAGCTTGCTTTACTGGGTATCAAGTGTTGAA

AhHMA4-3 AAGTCACTCTCTAGAGATATAACCCTACGTAGCTTGCTTTACTGGGTATCAAGTGTTGAA

AtHMA4 AGCAAATCAAGTCATCCAATGGCAGCAACAATCGTGGATTATGCAAAATCTGTTTCTGTT

NcHMA4-1-LC AGCAAATCAAGTCATCCAATGGCAGCAACGATTGTGGACTATGCTAAATCTGTTTCTGTT

NcHMA4-2-LC AGCAAATCAAGTCATCCAATGGCAGCAACGATTGTGGACTATGCTAAATCTGTTTCTGTT

NcHMA4-1-Ga AGCAAATCAAGTCATCCAATGGCAGCAACGATTGTGGACTATGCTAAATCTGTTTCTGTT

NcHMA4-2-Ga AGCAAATCAAGTCATCCAATGGCAGCAACGATTGTGGACTATGCTAAATCTGTTTCTGTT

NcHMA4-3-Ga AGCAAATCAAGTCATCCAATGGCAGCAACGATTGTGGACTATGCTAAATCTGTTTCTGTT

NcHMA4-1-CMA AGCAAATCAAGTCATCCAATGGCAGCAACGATTGTGGACTATGCTAAATCTGTTTCTGTT

NcHMA4-2-CMA AGCAAATCAAGTCATCCAATGGCAGCAACGATTGTGGACTATGCTAAATCTGTTTCTGTT

NcHMA4-1-SLM AGCAAATCAAGTCATCCAATGGCAGCAACGATTGTGGACTATGCTAAATCTGTTTCTGTT

NcHMA4-2-SLM AGCAAATCAAGTCATCCAATGGCAGCAACGATTGTGGACTATGCTAAATCTGTTTCTGTT

NcHMA4-3-SLM AGCAAATCAAGTCATCCAATGGCAACAACGATCGTGGACTATGCTAAATCTGTTTCTGTT

NcHMA4-4-SLM AGCAAATCAAGTCATCCAATGGCAGCAACGATTGTGGACTATGCTAAATCTGTTTCTGTT

AhHMA4-1 AGCAAATCAAGTCATCCAATGGCTGCAACAATCGTGGACTACGCGAAATCCGTTTCTGTT

AhHMA4-2 AGCAAATCAAGTCATCCAATGGCTGCAACAATCGTGGACTACGCGAAATCCGTTTCTGTT

AhHMA4-3 AGCAAATCAAGTCATCCAATGGCTGCAACAATCGTGGACTACGCGAAATCCGTTTCTGTT

AtHMA4 GAGCCTAGGCCTGAAGAGGTTGAGGATTACCAGAACTTTCCAGGTGAAGGAATCTACGGG

NcHMA4-1-LC GAGCCTAGGAGTGAAGAGGTTGAGGATTATCAGAACTTTCCTGGTGAAGGAATCTATGGG

NcHMA4-2-LC GAGCCTAGGAGTGAAGAGGTTGAGGATTATCAGAACTTTCCTGGTGAAGGAATCTATGGG

NcHMA4-1-Ga GAGCCTAGGAGTGAAGAGGTTGAGGATTATCAGAACTTTCCTGGTGAAGGAATCTATGGG

NcHMA4-2-Ga GAGCCTAGGAGTGAAGAGGTTGAGGATTATCAGAACTTTCCTGGTGAAGGAATCTATGGG

NcHMA4-3-Ga GAGCCTAGGAGTGAAGAGGTTGAGGATTATCAGAACTTTCCTGGTGAAGGAATCTATGGG

NcHMA4-1-CMA GAGCCTAGGAGTGAAGAGGTTGAGGATTATCAGAACTTTCCTGGTGAAGGAATCTATGGG

NcHMA4-2-CMA GAGCCTAGGAGTGAAGAGGTTGAGGATTATCAGAACTTTCCTGGTGAAGGAATCTATGGG

NcHMA4-1-SLM GAGCCTAGGAGTGAAGAGGTTGAGGATTATCAGAACTTTCCTGGTGAAGGAATCTATGGG

NcHMA4-2-SLM GAGCCTAGGAGTGAAGAGGTTGAGGATTATCAAAACTTTCCAGGTGAAGGAATCTATGGG

NcHMA4-3-SLM GAGCCTAGGAGTGAAGAGGTTGAGGATTATCATAACTTTCCAGGTGAAGGAATCTATGGG

NcHMA4-4-SLM GAGCCTAGGAGTGAAGAGGTTGAGGATTATCAGAACTTTCCTGGTGAAGGAATCTATGGG

AhHMA4-1 GAGCCTAGGCCTGAAGAGGTAGAGGACTACCAGAATTTTCCAGGTGAAGGAATCTACGGG

AhHMA4-2 GAGCCTAGGCCTGAAGAGGTAGAGGACTACCAGAATTTTCCAGGTGAAGGAATCTACGGG

AhHMA4-3 GAGCCTAGGCCTGAAGAGGTTGAGGATTACCAGAATTTTCCAGGTGAAGGAATCTACGGG

AtHMA4 AAGATTGATGGTAACGATATCTTCATTGGGAACAAAAAGATAGCTTCTCGAGCTGGTTGT

NcHMA4-1-LC AAGATTGATGGGAACAATGTTTACATTGGGAACAAAAGGATTGCTTCACGAGCTGGTTGT

NcHMA4-2-LC AAGATTGATGGGAACAATGTTTACATTGGGAACAAAAGGATTGCTTCACGAGCTGGTTGT

NcHMA4-1-Ga AAGATTGATGGGAACAATGTTTACATTGGGAACAAAAGGATTGCTTCACGAGCTGGTTGT

NcHMA4-2-Ga AAGATTGATGGGAACAATGTTTACATTGGGAACAAAAGGATTGCTTCACGAGCTGGTTGT

NcHMA4-3-Ga AAGATTGATGGGAACAATGTTTACATTGGGAACAAAAGGATTGCTTCACGAGCTGGTTGT

NcHMA4-1-CMA AAGATTGATGGGAACAATGTTTACATTGGGAACAAAAGGATTGCTTCACGAGCTGGTTGT

NcHMA4-2-CMA AAGATTGATGGGAACAATGTTTACATTGGGAACAAAAGGATTGCTTCACGAGCTGGTTGT

NcHMA4-1-SLM AAGATTGATGGGAACAATGTTTACATTGGGAACAAAAGGATTGCTTCACGAGCTGGTTGT

NcHMA4-2-SLM AAGATTGATGGGAACAATGTTTACATTGGGAACAAAAGGATTGCTTCACGAGCTGGTTGT

NcHMA4-3-SLM AAGATTGATGGGAACAATGTTTACATTGGGAACAAAAGGATTGCTTCACGAGCTGGTTGT

NcHMA4-4-SLM AAGATTGATGGGAACAATGTTTACATTGGGAACAAAAGGATTGCTTCACGAGCTGGTTGT

AhHMA4-1 AAGATTGATGGGAACGATATCTACATCGGGAACAAAAGGATTGCTTCTCGAGCTGGTTGT

AhHMA4-2 AAGATTGATGGGAACGATATCTACATCGGGAACAAAAGGATTGCTTCTCGAGCTGGTTGT

AhHMA4-3 AAGATTGATGGGAACGATATCTACATCGGGAACAAAAGGATTGCTTCTCGAGCTGGTTGT

AtHMA4 TCAACAGTTCCAGAGATTGAAGTTGATACCAAAGGCGGGAAGACTGTTGGATACGTCTAT

NcHMA4-1-LC TCAACAGTTCCAGAGATTGAGGTTGATACCAAAAAAGGAAAGACTGTCGGATACGTCTAT

NcHMA4-2-LC TCAACAGTTCCAGAGATTGAGGTTGATACCAAAAAAGGAAAGACTGTCGGATACGTCTAT

NcHMA4-1-Ga TCAACAGTTCCAGAGATTGATGTTGATACCAAAAAAGGAAAGACTGTCGGATACGTCTAT

NcHMA4-2-Ga TCAACAGTTCCAGAGATTGATGTTGATACCAAAAAAGGAAAGACTGTCGGATACGTCTAT

NcHMA4-3-Ga TCAACAGTTCCAGAGATTGATGTTGATACCAAAAAAGGAAAGACTGTCGGATACGTCTAT

NcHMA4-1-CMA TCAACAGTTCCAGAGATTGATGTTGATACCAAAAAAGGAAAGACTGTCGGATACGTCTAT

NcHMA4-2-CMA TCAACAGTTCCAGAGATTGATGTTGATACCAAAAAAGGAAAGACTGTCGGATACGTCTAT

NcHMA4-1-SLM TCAACAGTTCCAGAGATTGATGTTGATACCAAAAAAGGAAAGACTGTCGGATACGTCTAT

NcHMA4-2-SLM TCAACAGTTCCAGAGATTGATGTTGATACCAAAAAAGGAAAGACTGTCGGATACGTCTAT

NcHMA4-3-SLM TCAACAGTTCCAGAGATTGATGTTGATACCAAAGAAGGAAAGACTGTCGGATACGTCTAT

NcHMA4-4-SLM TCAACAGTTCCAGAGATTGATGTTGATACCAAAGAAGGAAAGACTGTCGGATACGTCTAT

AhHMA4-1 TCAACAGTTCCAGAGATCGAAGTTGATACAAAAGGTGGAAAGACTGTTGGATACGTCTAT

AhHMA4-2 TCAACAGTTCCAGAGACTGAAATTGATACCAAAGGTGGGAAGACTGTTGGATACGTCTAT

AhHMA4-3 TCAACAGTTCCAGAGACTGAAATTGATACCAAAGGTGGGAAGACTGTTGGATACGTCTAT

AtHMA4 GTAGGTGAAAGACTAGCTGGATTTTTCAATCTTTCTGATGCTTGTAGATCTGGTGTTTCT

NcHMA4-1-LC GTAGGTGAAAGATTAGCTGGAGTTTTCAATCTTTCCGATGCTTGTAGATCCGGAGTAGCT

NcHMA4-2-LC GTAGGTGAAAGATTAGCTGGAGTTTTCAATCTTTCCGATGCTTGTAGATCCGGAGTAGCT

NcHMA4-1-Ga GTAGGTGAAAGATTAGCTGGAGTTTTCAATCTTTCCGATGCTTGTAGATCCGGAGTAGCT

NcHMA4-2-Ga GTAGGTGAAAGATTAGCTGGAGTTTTCAATCTTTCCGATGCTTGTAGATCCGGAGTAGCT

NcHMA4-3-Ga GTAGGTGAAAGATTAGCTGGAGTTTTCAATCTTTCCGATGCTTGTAGATCCGGAGTAGCT

NcHMA4-1-CMA GTAGGTGAAAGATTAGCTGGAGTTTTCAATCTTTCCGATGCTTGTAGATCCGGAGTAGCT

NcHMA4-2-CMA GTAGGTGAAAGATTAGCTGGAGTTTTCAATCTTTCCGATGCTTGTAGATCCGGAGTAGCT

NcHMA4-1-SLM GTAGGTGAAAGATTAGCTGGAGTTTTCAATCTTTCCGATGCTTGTAGATCCGGAGTAGCT

NcHMA4-2-SLM GTAGGTGAAAGATTAGCTGGAGTTTTCAATCTTTCCGATGCTTGTAGATCCGGAGTAGCT

NcHMA4-3-SLM GTAGATGAAAGATTAGCTGGAGTTTTCAATCTTTCTGATGCTTGTAGATCCGGAGTAGCT

NcHMA4-4-SLM GTAGATGAAAGATTAGCTGGAGTTTTCAATCTTTCTGATGCTTGTAGATCCGGAGTAGCT

AhHMA4-1 GTAGGTGAAAGACTAGCTGGAGTTTTCAATCTTTCTGATGCTTGTAGATCAGGTGTATCT

AhHMA4-2 GTAGGTGAAAGACTAGCTGGAGTTTTCAATCTTTCTGATGCTTGTAGATCAGGTGTATCT

AhHMA4-3 GTAGGTGAAAGACTAGCTGGAGTTTTCAATCTTTCTGATGCTTGTAGATCAGGTGTATCT

AtHMA4 CAAGCAATGGCAGAACTGAAATCTCTAGGAATCAAAACCGCAATGCTAACGGGAGATAAT

NcHMA4-1-LC CAAGCAATGAAGGAACTCAAAGATCTTGGAATCAAAACCGCAATGCTAACAGGAGATAAT

NcHMA4-2-LC CAAGCAATGAAGGAACTCAAAGATCTTGGAATCAAAACCGCAATGCTAACAGGAGATAAT

NcHMA4-1-Ga CAAGCAATGAAGGAACTCAAAGATCTTGGAATCAAAACCGCAATGCTAACAGGAGATAAT

NcHMA4-2-Ga CAAGCAATGAAGGAACTCAAAGATCTTGGAATCAAAACCGCAATGCTAACAGGAGATAAT

NcHMA4-3-Ga CAAGCAATGAAGGAACTCAAAGATCTTGGAATCAAAACCGCAATGCTAACAGGAGATAAT

NcHMA4-1-CMA CAAGCAATGAAGGAACTCAAAGATCTTGGAATCAAAACCGCAATGCTAACAGGAGATAAT

NcHMA4-2-CMA CAAGCAATGAAGGAACTCAAAGATCTTGGAATCAAAACCGCAATGCTAACAGGAGATAAT

NcHMA4-1-SLM CAAGCAATGAAGGAACTCAAAGATCTTGGAATCAAAACCGCAATGCTAACAGGAGATAAT

NcHMA4-2-SLM CAAGCAATGAAGGAACTCAAAGATCTTGGAATCAAAACCGCAATGCTAACAGGAGATAAT

NcHMA4-3-SLM CAAGCAATGAAGGAACTCAAAGATCTTGGAATCAAAACCGCAATGCTAACAGGAGATAAT

NcHMA4-4-SLM CAAGCAATGAAGGAACTCAAAGATCTTGGAATCAAAACCGCAATGCTAACAGGAGATAAT

AhHMA4-1 CAAGCAATGAAAGAACTAAAATCTCTAGGAATCAAAACCGCAATGCTAACGGGAGATAGT

AhHMA4-2 CAAGCAATGAAAGAACTAAAATCTCTAGGAATCAAAACCGCAATGCTAACGGGAGATAGT

AhHMA4-3 CAAGCAATGAAAGAACTAAAATCTCTAGGAATCAAAACCGCAATGCTAACGGGAGATAGT

AtHMA4 CAAGCCGCGGCAATGCATGCTCAAGAACAGCTAGGGAATGTTTTAGATGTTGTACATGGA

NcHMA4-1-LC CAAGATTCAGCAATGCAAGCTCAAGAACAGCTAGGGAATGCTTTGGATGTTGTTCATGGA

NcHMA4-2-LC CAAGATTCAGCAATGCAAGCTCAAGAACAGCTAGGGAATGCTTTGGATGTTGTTCATGGA

NcHMA4-1-Ga CAAGATTCAGCAATGCAAGCTCAAGAACAGCTAGGGAATGCTTTGGATGTTGTTCATGGA

NcHMA4-2-Ga CAAGATTCAGCAATGCAAGCTCAAGAACAGCTAGGGAATGCTTTGGATGTTGTTCATGGA

NcHMA4-3-Ga CAAGATTCAGCAATGCAAGCTCAAGAACAGCTAGGGAATGCTTTGGATGTTGTTCATGGA

NcHMA4-1-CMA CAAGATTCAGCAATGCAAGCTCAAGAACAGCTAGGGAATGCTTTGGATGTTGTTCATGGA

NcHMA4-2-CMA CAAGATTCAGCAATGCATGCTCAAGAACAGCTAGGGAATGCTTTGGATGTTGTTCATGGA

NcHMA4-1-SLM AAAGATTCAGCAATGCATGCTCAAGAACAGCTAGGGAATGCTTTGGATGTTGTTCATGGA

NcHMA4-2-SLM CAAGATTCAGCAATGCAAGCTCAAGAACAGCTAGGGAATGCTTTGGATGTTGTTCATGGA

NcHMA4-3-SLM AAAGATTCAGCAATGCATGCTCAAGAACAGCTAGGGAATGCTTTGGATGTTGTTCATGGA

NcHMA4-4-SLM AAAGATTCAGCAATGCATGCTCAAGAACAGCTAGGGAATGCTTTGGATGTTGTTCATGGA

AhHMA4-1 CAAGCTGCGGCAATGCATGCTCAAGAACAGCTAGGGAATGCTTTGGATGTTGTGCATGGA

AhHMA4-2 CAAGCTGCGGCAATGCATGCTCAAGAACAGCTAGGGAATGTTTTGGATGTTGTGCATGGA

AhHMA4-3 CAAGCTGCGGCAATGCATGCTCAAGAACAGCTAGGGAATGCTTTGGATGTTGTGCATGGA

AtHMA4 GATCTTCTTCCAGAAGATAAGTCCAGAATCATACAAGAGTTTAAGAAAGAGGG-ACCAAC

NcHMA4-1-LC GAGCTTCTTCCAGAAGACAAATCCAAAATCATACAAGAGTTTAAGAAAGAAGG-ACCAAC

NcHMA4-2-LC GAGCTTCTTCCAGAAGACAAATCCAAAATCATACAAGAGTTTAAGAAAGAAGG-ACCAAC

NcHMA4-1-Ga GAGCTTCTTCCAGAAGACAAATCCAAA-TCATACAAGAGTTTAAGAAAGAAGGGACCAAC

NcHMA4-2-Ga GAGCTTCTTCCAGAAGACAAATCCAAAATCATACAAGAGTTTAAGAAAGAAGG-ACCAAC

NcHMA4-3-Ga GAGCTTCTTCCAGAAGACAAATCCAAAATCATACAAGAGTTTAAGAAAGAAGG-ACCAAC

NcHMA4-1-CMA GAGCTTCTTCCAGAAGACAAATCCAAAATCATACAAGAGTTTAAGAAAGAAGG-ACCAAC

NcHMA4-2-CMA GAGCTTCTTCCTGAAGACAAATCCAAAATCATACAAGAGTTTAAGAAAGAAGG-ACCAAC

NcHMA4-1-SLM GAGCTTCTTCCTGAAGACAAATCCAAAATCATACAAGAGTTTAAGAAAGAAGG-ACCAAC

NcHMA4-2-SLM GAGCTTCTTCCAGAAGACAAATCCAAAATCATACAAGAGTTTAAGAAAGAAGG-ACCAAC

NcHMA4-3-SLM GAGCTTCTTCCAGAAGACAAATCCAAAATCATACAAGAGTTTAAGAAAGAAGG-ACCAAC

NcHMA4-4-SLM GAGCTTCTTCCAGAAGACAAATCCAAAATCATACAAGAGTTTAAGAAAGAAGG-ACCAAC

AhHMA4-1 GAACTTCTTCCAGAAGATAAATCTAAAATCATACAAGAGTTCAAGAAAGAAGG-ACCAAC

AhHMA4-2 GAACTTCTTCCAGAAGATAAATCTAAAATCATACAAGAGTTCAAGAAAGAAGG-ACCAAC

AhHMA4-3 GAACTTCTTCCAGAAGATAAATCTAAAATCATACAAGAGTTCAAGAAAGAAGG-ACCAAC

AtHMA4 CGCAATGGTAGGGGACGGTGTGAATGATGCACCAGCTTTAGCTACAGCTGATATTGGTAT

NcHMA4-1-LC TTGTATGGTAGGAGATGGTGTGAATGATGCACCAGCTTTAGCTAATGCTGATATTGGTAT

NcHMA4-2-LC TTGTATGGTAGGAGATGGTGTGAATGATGCACCAGCTTTAGCTAATGCTGATATTGGTAT

NcHMA4-1-Ga TTGTATGGTAGGAGATGGTGTGAATGATGCACCAGCTTTAGCTAATGCTGATATTGGTAT

NcHMA4-2-Ga TTGTATGGTAGGAGATGGTGTGAATGATGCACCAGCTTTAGCTAATGCTGATATTGGTAT

NcHMA4-3-Ga TTGTATGGTAGGAGATGGTGTGAATGATGCACCAGCTTTAGCTAATGCTGATATTGGTAT

NcHMA4-1-CMA TTGTATGGTAGGAGATGGTGTGAATGATGCACCAGCTTTAGCTAATGCTGATATTGGTAT

NcHMA4-2-CMA TTGTATGGTAGGAGATGGTGTGAATGATGCACCAGCTTTAGCTAATGCTGATATTGGTAT

NcHMA4-1-SLM TTGTATGGTAGGAGATGGTGTGAATGATGCACCAGCTTTAGCTAATGCTGATATTGGTAT

NcHMA4-2-SLM TTGTATGGTAGGAGATGGTGTGAATGATGCACCAGCTTTAGCTAATGCTGATATTGGTAT

NcHMA4-3-SLM TTGTATGGTAGGAGATGGTGTGAATGATGCACCAGCTTTAGCTAATGCTGATATTGGTAT

NcHMA4-4-SLM TTGTATGGTAGGAGATGGTGTGAATGATGCACCAGCTTTAGCTAATGCTGATATTGGTAT

AhHMA4-1 CGCAATGGTAGGGGACGGTGTGAACGATGCACCAGCTTTAGCTACAGCTGATATTGGTAT

AhHMA4-2 CGCAATGGTAGGGGACGGTGTGAATGATGCACCAGCTTTAGCTACAGCTGATATTGGTAT

AhHMA4-3 CGCAATGGTAGGGGACGGTGTGAATGATGCACCAGCTTTAGCTACAGCTGATATTGGTAT

AtHMA4 CTCCATGGGAATTTCTGGCTCTGCTCTTGCAACACAAACTGGTAATATTATTCTGATGTC

NcHMA4-1-LC CTCCATGGGGATTTCTGGCTCTGCGCTCGCGACGCAGACTGGTCATATCATTCTCATGTC

NcHMA4-2-LC CTCCATGGGGATTTCTGGCTCTGCGCTCGCGACGCAGACTGGTCATATCATTCTCATGTC

NcHMA4-1-Ga CTCCATGGGGATTTCTGGCTCTGCGCTCGCGACGCAGTCTGGTCATATCATTCTCATGTC

NcHMA4-2-Ga CTCCATGGGGATTTCTGGCTCTGCGCTCGCGACGCAGTCTGGTCATATCATTCTCATGTC

NcHMA4-3-Ga CTCCATGGGGATTTCTGGCTCTGCGCTCGCGACGCAGACTGGTCATATCATTCTCATGTC

NcHMA4-1-CMA CTCCATGGGGATTTCTGGCTCTGCGCTCGCGACGCAGACTGGTCATATCATTCTCATGTC

NcHMA4-2-CMA CTCCATGGGGATTTCTGGCTCTGCGCTCGCGACGCAGTCTGGTCATATCATTCTCATGTC

NcHMA4-1-SLM CTCCATGGGGATTTCTGGCTCTGCGCTCGCGACGCAGTCTGGTCATATCATTCTCATGTC

NcHMA4-2-SLM CTCCATGGGGATTTCTGGCTCTGCGCTCGCGACGCAGACTGGTCATATCATTCTTATGTC

NcHMA4-3-SLM CTCCATGGGGATTTCTGGCTCTGCGCTCGCGACGCAGTCTGGTCATATCATTCTCATGTC

NcHMA4-4-SLM CTCCATGGG-ATTTCTGGCTCTGCGCTCGCGACGCAGTCTGGTCATATCATTCTCATGTC

AhHMA4-1 CTCCATGGGGATTTCTGGCTCTGCTCTTGCAACACAGACTGGTCATATTATTCTGATGTC

AhHMA4-2 CTCCATGGGGATTTCTGGCTCTGCTCTTGCAACACAGACTGGTCATATTATTCTGATGTC

AhHMA4-3 CTCCATGGGGATTTCTGGCTCTGCTCTTGCAACACAGACTGGTCATATTATTCTGATGTC

AtHMA4 TAATGATATAAGAAGGATACCACAAGCGGTGAAGCTAGCGAGAAGAGCACGACGCAAAGT

NcHMA4-1-LC AAATGATATCAGAAGGATACCACAAGCGATAAAGCTAGCAAGAAGAGCTCAGCGGAAAGT

NcHMA4-2-LC AAATGATATCAGAAGGATACCACAAGCGATAAAGCTAGCAAGAAGAGCTCAGCGGAAAGT

NcHMA4-1-Ga AAATGATATCAGAAGGATACCAAAAGCGATAAAGCTAGCAAGAAGAGCTCAGCGGAAAGT

NcHMA4-2-Ga AAATGATATCAGAAGGATACCAAAAGCGATAAAGCTAGCAAGAAGAGCTCAGCGGAAAGT

NcHMA4-3-Ga AAATGATATCAGAAGGATACCACAAGCGATAAAGCTAGCAAGAAGAGCTCAGCGGAAAGT

NcHMA4-1-CMA AAATGATATCAGAAGGATACCACAAGCGATAAAGCTAGCAAGAAGAGCTCAGCGGAAAGT

NcHMA4-2-CMA AAATGATATCAGAAGGATACCAAAAGCGATAAAGCTAGCAAGAAGAGCTCAGCGGAAAGT

NcHMA4-1-SLM AAATGATATCAGAAGGATACCAAAAGCGATAAAGCTAGCAAGAAGAGCTCAGCGGAAAGT

NcHMA4-2-SLM TAATGATATCAGAAGGATACCACAAGCGATAAAGCTAGCAAGAAGAGCTCAGCGGAAAGT

NcHMA4-3-SLM AAATGATATCAGAAGGATACCAAAAGCGATAAAGCTAGCAAGAAGAGCTCAGCGGAAAGT

NcHMA4-4-SLM AAATGATATCAGAAGGATACCA-AAGCGATAAAGCTAGCAAGAAGAGCTCAGCGGAAAGT

AhHMA4-1 TAATGACATAAGAAGGATACCACAAGCGGTGAAGCTAGCGAGAAGAGCTCGGCGCAAAGT

AhHMA4-2 TAATGACATAAGAAGGATACCACAAGCGGTGAAGCTAGCGAGAAGAGCTCGGCGCAAAGT

AhHMA4-3 TAATGACATAAGAAGGATACCACAAGCGGTGAAGCTAGCGAGAAGAGCTCGGCGCAAAGT

AtHMA4 TGTTGAAAACGTGTGTCTATCGATCATTTTAAAAGCAGGAATACTCGCTTTGGCATTTGC

NcHMA4-1-LC TCTTCAAAACGTGTTCATCTCCATCACTTTGAAAGTAGGGATACTGGTTTTAGCATTTGC

NcHMA4-2-LC TCTTCAAAACGTGTTCATCTCCATCACTTTGAAAGTAGGGATACTGGTTTTAGCATTTGC

NcHMA4-1-Ga TCTTGAAAACGTGTTCATCTCCATCACTTTGAAAGTAGGGATACTGGTTTTAGCATTTGC

NcHMA4-2-Ga TCTTGAAAACGTGTTCATCTCCATCACTTTGAAAGTAGGGATACTGGTTTTAGCATTTGC

NcHMA4-3-Ga TCTTCAAAACGTGATCATCTCCATCACTTTGAAAGTAGGGATACTGGTTTTAGCATTTGC

NcHMA4-1-CMA TCTTCAAAACGTGTTCATCTCCATCACTTTGAAAGTAGGGATACTGGTTTTAGCATTTGC

NcHMA4-2-CMA TCTTGAAAACGTGTTCATCTCCATCACTTTGAAAGTAGGGATACTGGTTTTAGCATTTGC

NcHMA4-1-SLM TCTTGAAAACGTGTTCATCTCCATCACTTTGAAAGTAGGGATACTGGTTTTAGCATTTGC

NcHMA4-2-SLM TCTTCAAAACGTGATCATCTCCATCACTTTGAAAGTAGGGATACTGGTTTTAGCATTTGC

NcHMA4-3-SLM TCTTGAAAACGTGTTCATCTCCATCACTTTGAAAGTAGGGATACTGGTTTTAGCATTTGC

NcHMA4-4-SLM TCTTGAAAACGTGTTCATCTCCATCACTTTGAAAGTAGGGATACTGGTTTTAGCATTTGC

AhHMA4-1 TATTGAAAACGTGTGTCTTTCCATCATTTTAAAAGCAGGAATACTGGCTTTGGCATTTGC

AhHMA4-2 TATTGAAAACGTGTCTCTTTCCATCATTTTAAAAGCAGGAATACTGGCTTTGGCATTTGC

AhHMA4-3 TATTGAAAACGTGTGTCTTTCCATCATTTTAAAAGCAGGAATACTGGCTTTGGCATTTGC

AtHMA4 TGGTCATCCTTTGATTTGGGCTGCGGTTCTTGTTGATGTAGGGACTTGTCTGCTTGTGAT

NcHMA4-1-LC TGGTCATCCTTTGATTTGGGCTGCGGTGCTTACTGATGTAGGGACTTGCCTGATTGTGAT

NcHMA4-2-LC TGGTCATCCTTTGATTTGGGCTGCGGTGCTTACTGATGTAGGGACTTGCCTGATTGTGAT

NcHMA4-1-Ga TGGTCATCCTTTGATTTGGGCTGCGGTGCTTACTGATGTAGGGACTTGCCTGATTGTGAT

NcHMA4-2-Ga TGGTCATCCTTTGATTTGGGCTGCGGTGCTTACTGATGTAGGGACTTGCCTGATTGTGAT

NcHMA4-3-Ga TGGTCATCCTTTGATTTGGGCTGCGGTGCTTACTGATGTAGGGACTTGCCTGATTGTGAT

NcHMA4-1-CMA TGGTCATCCTTTGATTTGGGCTGCGGTGCTTACTGATGTAGGGACTTGCCTGATTGTGAT

NcHMA4-2-CMA TGGTCATCCTTTGATTTGGGCTGCGGTGCTTACTGATGTAGGGACTTGCCTGATTGTGAT

NcHMA4-1-SLM TGGTCATCCTTTGATTTGGGCTGCGGTGCTTACTGATGTAGGGACTTGCCTGATTGTGAT

NcHMA4-2-SLM TGGTCATCCTTTGATTTGGGCTGCGGTGCTTACTGATGTAGGGACTTGCCTGATTGTGAT

NcHMA4-3-SLM TGGTCATCCTTTGATTTGGGCTGCGGTGCTTACTGATGTAGGGACTTGCCTGATTGTGAT

NcHMA4-4-SLM TGGTCATCCTTTGATTTGGGCTGCGGTGCTTACTGATGTAGGGACTTGCCTGATTGTGAT

AhHMA4-1 TGGTCATCCTTTGATTTGGGCAGCGGTTCTTGTTGACGTAGGAACTTGTTTGCTTGTGAT

AhHMA4-2 TGGTCATCCTTTGATTTGGGCAGCGGTTCTTGTTGACGTAGGAACTTGTTTGCTTGTGAT

AhHMA4-3 TGGTCATCCTTTGATTTGGGCAGCGGTTCTTGTTGACGTAGGAACTTGTTTGCTTGTGAT

AtHMA4 TTTCAATAGTATGTTGCTGCTGCGAGAGAAGAAAAAGATTGGGAACAAAAAGTGTTACAG

NcHMA4-1-LC TCTCAACAGTATGTTGCTTCTGCGAGAGAAGGATAAATCTAAGATCAAGAAGTGTTACAG

NcHMA4-2-LC TCTCAACAGTATGTTGCTTCTGCGAGAGAAGGATAAATCTAAGATCAAGAAGTGTTACAG

NcHMA4-1-Ga TTTTAACAGTATGTTGCTTCTGCGAGAGAAGGATAAATCTAAGAACAAGAAGTGTTACAG

NcHMA4-2-Ga TTTTAACAGTATGTTGCTTCTGCGAGAGAAGGATAAATCTAAGAACAAGAAGTGTTACAG

NcHMA4-3-Ga TCTCAACAGTATGTTGCTTCTGCGAGAGAAGGATAAATCTAAGATCAAGAAGTGTTACAG

NcHMA4-1-CMA TCTCAACAGTATGTTGCTTCTGCGAGAGAAGGATAAATCTAAGATCAAGAAGTGTTACAG

NcHMA4-2-CMA TTTTAACAGTATGTTGCTTCTGCGAGAGAAGGATAAATCTAAGAACAAGAAGTGTTACAG

NcHMA4-1-SLM TTTTAACAGTATGTTGCTTCTGCGAGAGAAGGATAAATCTAAGAACAAGAAGTGTTACAG

NcHMA4-2-SLM TCTCAACAGTATGTTGCTTCTGCGAGAGAAGGATAAATCTAAGATCAAGAAGTGTTACAG

NcHMA4-3-SLM TTTTAACAGTATGTTGCTTCTGCGAGAGAAGGATAAATCTAAGAACAAGAATTGTTACAG

NcHMA4-4-SLM TTTTAACAGTATGTTGCTTCTGCGAGAGAAGGATAAATCTAAGAACAAGAATTGTTACAG

AhHMA4-1 TCTCAATAGTATGTTGCTGCTGCGAGAGAAGAAAAAGATTGGGAACAAAAAGTGTTACAG

AhHMA4-2 TTTCAATAGTATGTTGCTGCTGCGAGAGAAGAAAAAGATTGGGAACAAAAAGTGTTACAG

AhHMA4-3 TCTCAATAGTATGTTGCTGCTGCGAGAGAAGAAAAAGATTGGGAACAAAAAGTGTTACAG

AtHMA4 GGCTTCTACATCTAAGTTGAATGGTAGGAAACTTGAAGGCGATGATGATTATGTTGTGGA

NcHMA4-1-LC G------------------------AAGAAAGTTGAAGGCGGCGATGACCAAGGCCTTGA

NcHMA4-2-LC G------------------------AAGAAACTTGAAGGCGTCGATGACCAAGGCCTTGA

NcHMA4-1-Ga GGCTTCTACATCTGTGTTGAATGGTAAGAAACTTGAAGGCGATGATGAAGAAGGTCTTGA

NcHMA4-2-Ga GGCTTCTACATCTGTGTTGAATGGTAAGAAACTTGAAGGCGATGATGAAGAAGGTCTTGA

NcHMA4-3-Ga G------------------------AAGAAACTTGAAGGCGTCGATGACCAAGGCCTTGA

NcHMA4-1-CMA G------------------------AAGAAACTTGAAGGCGGCGATGACCAAGGCCTTGA

NcHMA4-2-CMA GGCTTCTACATCTGTGTTGAATGGTAAGAAACTTGAAGGCGATGATGAAGAAGGTCTTGA

NcHMA4-1-SLM GGCTTCTACATCTGTGTTGAATGGTAAGAAACTTGAAGGCGATGATGAAGAAGGTCTTGA

NcHMA4-2-SLM G------------------------AAGAAACTTGAAGGCGTCGATGACCAAGGCCTTGA

NcHMA4-3-SLM GGCTTCTACATCTGTGTTGAATGGTAAGAAACTTGAAGGCGGCGATGACCAAGGCCTTGA

NcHMA4-4-SLM GGCTTCTACATCTGTGTTGAATGGTAAGAAACTTGAAGGCGATGATGAAGAAGGTCTTGA

AhHMA4-1 GGCTTCTACATCTATGTTGAATGGTAGGAAACTCGAAGGCGATGATGATGATGCTGTGGA

AhHMA4-2 GGCTTCTACATCTATGTTGAATGGTAGGAAACTCGAAGGCGATGATGATGATGCTGTGGA

AhHMA4-3 GGCTTCTACATCTATGTTGAATGGTAGGAAACTCGAAGGCGATGATGATGATGCTGTGGA

AtHMA4 CTTAGAAGCAGGCTTGTTAACAAAGAGCGGGAATGGTCAATGCAAATCAAGCTGTTGTGG

NcHMA4-1-LC CTTAGAAGCAGGGTTGTTGTCAAAGAG---------TCAATGCAACTCAGGATGTTGTGG

NcHMA4-2-LC CTTAGAAGCAGGGTTGTTATCAAAGAG---------TCAATGCAACTCAGGATGTTGTGG

NcHMA4-1-Ga CTTAGAAGCAGGGTTGGTATCAAAGAG---------TCAATGCAACTCAGGATGTTGTGG

NcHMA4-2-Ga CTTAGAAGCAGGGTTGGTATCAAAGAG---------TCAATGCAACTCAGGATGTTGTGG

NcHMA4-3-Ga CTTAGAAGCAGGGTTGTTATCAAAGAG---------TCAATGCAACTCAGGATGTTGTGG

NcHMA4-1-CMA CTTAGAAGCAGGGTTGTTCTCAAAGAG---------TCAATGCAACTCAGGATGTTGTGG

NcHMA4-2-CMA CTTAGAAGCAGGGTTGGTATCAAAGAG---------TCAATGCAACTCAGGATGTTGTGG

NcHMA4-1-SLM CTTAGAAGCAGGGTTGGTATCAAAGAG---------TCAATGCAACTCAGGATGTTGTGG

NcHMA4-2-SLM CTTAGAAGCAGGGTTGTTATCAAAGAG---------TCAATGCAACTCAGGATGTTGTGG

NcHMA4-3-SLM CTTAGAAGCAGGGTTGTTCTCAAAGAG---------TCAATGCAACTCAGGATGTTGTGG

NcHMA4-4-SLM CTTAGAAGCAGGGTTGGTATCAAAGAG---------TCAATGCAACTCAGGATGTTGTGG

AhHMA4-1 CTTAGAAGCAGGCTTGTTAACAAAAAGCGGGAATGGTCAATGTAAATCAAGCTGTTGTGG

AhHMA4-2 CTTAGAAGCAGGCTTGTTAACAAAAAGCGGGAATGGTCAATGTAAATCAAGCTGTTGTGG

AhHMA4-3 CTTAGAAGCAGGCTTGTTAACAAAAAGCGGGAATGGTCAATGTAAATCAAGCTGTTGTGG

AtHMA4 AGATAAGAAAAATCAAGAGAATGTTGTGATGATGAAACCAAGTAGTAAAACCAGTTCTGA

NcHMA4-1-LC TGATAAGAAAAGCCAAGAGAAGGTGATGTTGATGAGACCAGCTAGTAAAACCAGTTCTGA

NcHMA4-2-LC TGATAAGAAAAGCCAAGAGAAGGTGATGTTGATGAGACCAGCTAGTAAAACCAGTTCTGA

NcHMA4-1-Ga TGATAAGAATAGCCAAGGGAAGGTGATGTTGATGAGACCAGCTAGTAAAACCAGTACTGA

NcHMA4-2-Ga TGATAAGAATAGCCAAGGGAAGGTGATGTTGATGAGACCAGCTAGTAAAACCAGTACTGA

NcHMA4-3-Ga TGATAAGAAAAGCCAAGAGAAGGTGATGTTGATGAGACCAGCTAGTAAAACCAGTTCTGA

NcHMA4-1-CMA TGATAAGAAAAGCCAAGAGAAGGTGATGTTGATGAGACCAGCTAGTAAAACCAGTACTGA

NcHMA4-2-CMA TGATAAGAAAAGCCAAGAGAAGGTGATGTTGATGAGACCAGCTAGTAAAACCAGTACTGA

NcHMA4-1-SLM TGATAAGAAAAGCCAAGAGAAGGTGATGTTGATGAGACCAGCTAGTAAAACCAGTACTGA

NcHMA4-2-SLM TGATAAGAAAAGCCAAGAGAAGGTGATGTTGATGAGACCAGCTAGTAAAACCAGTTCTGA

NcHMA4-3-SLM TGATAAGAAAAGCCAAGAGAAGGTGATGTTGATGAGACCAGCTAGTAAAACCAGTACTGA

NcHMA4-4-SLM TGATAAGAAAAGCCAAGAGAAGGTGATGTTGATGAGACCAGCTAGTAAAACCAGTACTGA

AhHMA4-1 AGATAAGAAAAATCAAGAGAAGGTTGTGATGATGAAACCAAGTAGTAAAACCAGTTCTGA

AhHMA4-2 AGATAAGAAAAATCAAGAGAAGGTTGTGATGATGAAACCAAGTAGTAAAACCAGTTCTGA

AhHMA4-3 AGATAAGAAAAATCAAGAGAAGGTTGTGATGATGAAACCAAGTAGTAAAACCAGTTCTGA

AtHMA4 TCATTCTCACCCTGGTTGTTGTGGCGATAAGAAGGAAGAAAAAGTGAAGCCGCTTGTGAA

NcHMA4-1-LC CCATCTTCACTCTGGTTGTTGTGGTGAAAAGAAGCAAGAGAGTGTAAAGC---TTGTGAA

NcHMA4-2-LC CCATCTTCACTCTGGTTGTTGCGGTGAAAAGAAGCAAGAGAGTGTAAAGC---TTGTGAA

NcHMA4-1-Ga CCATCTTCACTCTGGTTGTTGTGGTGAAAAGAAGCAAGAGAGTGTAAAGC---TTGTGAA

NcHMA4-2-Ga CCATCTTCACTCTGGTTGTTGTGGTGAAAAGAAGCAAGAGAGTGTAAAGC---TTGTGAA

NcHMA4-3-Ga CCATCTTCACTCTGGTTGTTGTGGTGAAAAGAAGCAAGAGAGTGTAAAGC---TTGTGAA

NcHMA4-1-CMA CCATCTTCACTCTGGTTGTTGTGGTGAAAAGAAGCAAGAGAGTGTAAAGC---TTGTGAA

NcHMA4-2-CMA CCATCTTCACTCTGGTTGTTGTGGTGAAAAGAAGCAAGAGAGTGTAAAGC---TTGTGAA

NcHMA4-1-SLM CCATCTTCACTCTGGTTGTTGTGGTGAAAAGAAGCAAGAGAGTGTAAAGC---TTGTGAA

NcHMA4-2-SLM CCATCTTCACTCTGGTTGTTGTGGTGAAAAGAAGCAAGAGAGTGTAAAGC---TTGTGAA

NcHMA4-3-SLM CCATCTTCACTCTGGTTGTTGTGGTGAAAAGAATCAAGAGAGTGTAAAGC---TTGTGAA

NcHMA4-4-SLM CCATCTTCACTCTGGTTGTTGTGGTGAAAAGA-TCAAGAGAGTGTAAAGC---TTGTGAA

AhHMA4-1 TCATTCTCACCCTGGTTGTTGTGGCGATAAGAAGCAAGGCAATGTGAAGCCGCTTGTGAG

AhHMA4-2 TCATTCTCACCCTGGTTGTTGTGGCGATAAGAAGCAAGGCAATGTGAAGCCGCTTGTGAG

AhHMA4-3 TCATTCTCACCCTGGTTGTTGTGGCGATAAGAAGCAAGGCAATGTGAAGCCGCTTGTGAG

AtHMA4 AGATGGCTGTTGCAGTGAGAAAACTAGGAAATCAGAGGGAGATATGGTTTCATTGAGCTC

NcHMA4-1-LC AGATAGCTGTTGCGGTGAGAAAAGTAGGAAACCAGAGGGAGATATGGCTTCACTGAGCTC

NcHMA4-2-LC AGATAGCTGTTGCGGTGAGAAAAGTAGGAAACCAGAGGGAGATATGGCTTCACTGAGCTC

NcHMA4-1-Ga AGATAGCTGTTGCGGTGAGAAAAGTAGGAAACCAGAGGGAGACATGGCTTCACTGAGCTC

NcHMA4-2-Ga AGATAGCTGTTGCGGTGAGAAAAGTAGGAAACCAGAGGGAGACATGGCTTCACTGAGCTC

NcHMA4-3-Ga AGATAGCTGTTGCGGTGAGAAAAGTAGGAAACCAGAGGGAGACATGGCTTCACTGAGCTC

NcHMA4-1-CMA AGATAGCTGTTGCGGTGAGAAAAGTAGGAAACCAGAGGGAGATATGGCTTCACTGAGCTC

NcHMA4-2-CMA AGATAGCTGTTGCGGTGAGAAAAGTAGGAAACCAGAGGGAGACATGGCTTCACTGAGCTC

NcHMA4-1-SLM AGATAGCTGTTGCGGTGAGAAAAGTAGGAAACAAGAGGGAGATATGGCTTCACTGAGCTC

NcHMA4-2-SLM AGATAGCTGTTGCGGTGAGAAAAGTAGGAAACCAGTGGGAGACATGGCTTCACTGAGCTC

NcHMA4-3-SLM AGATAGCTGTTGCGGTGAGAAAAGTAAGAAACCAGAGGGAGATATGGCTTCACTGAGCTC

NcHMA4-4-SLM AGATAGCTGTTGCGGTGAGAAAAGTAAGAAACCAGAGGGAGATATGGCTTCACTGAGCTC

AhHMA4-1 AGATGGCGGTTGCAGTGAGGAAACTAGGAAAGCAGTGGGAGACATGGTTTCATTGAGCTC

AhHMA4-2 AGATGGCGGTTGCAGTGAGGAAACTAGGAAAGCAGTGGGAGACATGGTTTCATTGAGCTC

AhHMA4-3 AGATGGCGGTTGCAGTGAGGAAACTAGGAAAGCAGTGGGAGACATGGTTTCATTGAGCTC

AtHMA4 ATGTAAGAAGTCTAGTCATGTCAAACATGACCTGAAAATGAAAGGTGGTTCAGGTTGTTG

NcHMA4-1-LC ATGCAAGAAGTCTAA-CA--------ATGACCTGAAAATGAAAGGTGGTTCAAGTTGTTG

NcHMA4-2-LC ATGCAAGAAGTCTAA-CA--------ATGACCTGAAAATGAAAGGTGGTTCAAGTTGTTG

NcHMA4-1-Ga ATGCAAGAAGTCTAA-CA--------ATGACCTGAAAATGAAAGGTGGTTCAAGTTGTTG

NcHMA4-2-Ga ATGCAAGAAGTCTAA-CA--------ATGACCTGAAAATGAAAGGTGGTTCAAGTTGTTG

NcHMA4-3-Ga ATGCAAGAAGTCTAA-CA--------ATGACATAAAAATGAAAGGTGGTTCAAGTTGTTG

NcHMA4-1-CMA ATGCAAGAAGTCTAA-CA--------ATGACCTGAAAATGAAAGGTGGTTCAAGTTGTTG

NcHMA4-2-CMA ATGCAAGAAGTCTAA-CA--------ATGACCTGAAAATGAAAGGTGGTTCAAGTTGTTG

NcHMA4-1-SLM ATGCAAGAAGTCTGA-CA--------ATGACCTGAAAATGAAAGGTGGTTCAAGTTGTTG

NcHMA4-2-SLM ATGCAAGAAGTCTAA-CA--------ATGACCTGAAAATGAAAGGTGGTTCAAGTTGTTG

NcHMA4-3-SLM ATGCAAGAACTCTAA-CA--------ATGACCTGAAAATGAAAGGTGGTTCAAGTTGTTG

NcHMA4-4-SLM ATGCAAGAACTCTAA-CA--------ATGACCTGAAAATGAAAGGTGGTTCAAGTTGTTG

AhHMA4-1 ATGTAAGAAGTCTAGTCATGTCAAACATGACCTGAAAATGAAAGGTGGTTCAGGTTGTTG

AhHMA4-2 ATGTAAGAAGTCTAGTCATGTTAAACATGACCTGAAAATGAAAGGTGGTTCAGGTTGTTG

AhHMA4-3 ATGTAAGAAGTCTAGTCATGTCAAACATGACCTGAAAATGAAAGGTGGTTCAGGTTGTTG

AtHMA4 TGCTAGCAAAAATGAGAAAGGGAAGGAAGTAGTGGCAAAGAGCTGTTGTGAGAAACCCAA

NcHMA4-1-LC TGCTAGTAAAAATGAGAAGCTGAAGGAAGCAGTAGTA-----------------------

NcHMA4-2-LC TGCTAGTAAAAATGAGAAGCTGAAGGAAGTAGTAGTA-----------------------

NcHMA4-1-Ga TGCTAGTAAAAATGAGAAGCTGAAGGAAGTAGTAGTA-----------------------

NcHMA4-2-Ga TGCTAGTAAAAATGAGAAGCTGAAGGAAGTAGTAGTA-----------------------

NcHMA4-3-Ga TGCTAGTAAAAATGAGAAGCTGAAGGAAGTAGTAGTA-----------------------

NcHMA4-1-CMA TGCTAGTAAAAATGAGAAGCTGAAGGAAGTAGTAGTA-----------------------

NcHMA4-2-CMA TGCTAGTAAAAATGAGAAGCTGAAGGAAGTAGTAGTA-----------------------

NcHMA4-1-SLM TGCTAGTAAAAATGAGAAGCTGAAGGAAGTAGCAGTA-----------------------

NcHMA4-2-SLM TGCTAGTAAAAATGAGAAGCTGAAGGAAGTAGTAGTA-----------------------

NcHMA4-3-SLM TGCTAGTAAAAATGAGAAGCTGAAGGAAGTAGTAGTA-----------------------

NcHMA4-4-SLM TGCTAGTAAAAATGAGAAGCTGAAGGAAGTAGTAGTA-----------------------

AhHMA4-1 TGCTAACAAAAGTGAGAAGGTAGAGGAAGTAGTGGCAAAGAGCTGTTGTGAGAAACCAAA

AhHMA4-2 TGCTAACAAAAGTGAGAAGGTAGAGGAAGTAGTGGCAAAGAGCTGTTGTGAGAAACCAAA

AhHMA4-3 TGCTAACAAAAGTGAGAAGGTAGAGGAAGTAGTGGCAAAGAGCTGTTGTGAGAAACCAAA

AtHMA4 ACAGCAGGTGGAGAGTGTTGGAGACTGCAAGTCTGGTCATTGCGAGAAGAAGAAGCAAGC

NcHMA4-1-LC ---GCAA----AGAG---------CTGC------------TGTGAAGACAAGGAGAAAAC

NcHMA4-2-LC ---GCAA----AGAG---------CTGC------------TGTGAAGACAAGGAGAAAGC

NcHMA4-1-Ga ---GCAA----AGAG---------CTGC------------TGTGAAGACAAGGAGAAAGC

NcHMA4-2-Ga ---GCAA----AGAG---------CTGC------------TGTGAAGACAAGGAGAAAGC

NcHMA4-3-Ga ---GCAA----AGAG---------CTGC------------TGTGAAGAGAAGGAGAAAGC

NcHMA4-1-CMA ---GCAA----AGAG---------CTGC------------TGTGGAGAGAAGGAGAAAGC

NcHMA4-2-CMA ---GCAA----AGAG---------CTGC------------TGTGAAGACAAGGAGAAAGC

NcHMA4-1-SLM ---GCAA----AGAC---------CTGC------------TGTGAAGACAAGGAGAAAGC

NcHMA4-2-SLM ---GCAA----AGAG---------CTGC------------TGTGAAGAGAAGGAGAAAGC

NcHMA4-3-SLM ---GCAA----AGAG---------CTGC------------TGTGGAGAGAAGGAGAAAGC

NcHMA4-4-SLM ---GCAA----AGAG---------CTGC------------TGTGGAGAGAAGGAGAAAGC

AhHMA4-1 ACAGCAAATGGAGAGTGCTGGAGACTGCAAATCTAGCCATTGCGAGGAGAAGAAGCATGC

AhHMA4-2 ACAGCAAATGGAGAGTGCTGGAGACTGCAAATCTAGCCATTGCGAGGAGAAGAAGCATGC

AhHMA4-3 ACAGCAAATGGAGAGTGCTGGAGACTGCAAATCTAGCCATTGCGAGGAGAAGAAGCATGC

AtHMA4 TGAAGACATTGTTGTCCCGGTGCAGATTATTGGTCATGCATTAACGCATGTGGAGATCGA

NcHMA4-1-LC AGAGGGAAATGTTGA---GATGCAGATT------------CTAA---ATTTGGAGAAAGG

NcHMA4-2-LC AGAGGGAAATGTTGA---GATGCAGATT------------CTAA---ATTTGGAGAAAGG

NcHMA4-1-Ga AGAGGGAAATGTTGA---GATGCAGATT------------CTTG---ATTTGGAGAAAGG

NcHMA4-2-Ga AGAGGGAAATGTTGA---GATGCAGATT------------CTTG---ATTTGGAGAAAGG

NcHMA4-3-Ga AGAGGGAAATGTTGA---GATGCAGATT------------CTAA---ATTTGGAGAAAGG

NcHMA4-1-CMA AGAGGGAAATGTTGA---GATGCAGATT------------CTAA---ATTTGGAGAAAGG

NcHMA4-2-CMA AGAGGGAAATGTTGA---GATGCAGATT------------CTTG---ATTTGGAGAAAGG

NcHMA4-1-SLM AGAGGGAAATGTTGA---GATGCAGATT------------CTTG---ATTTGGAGAAAGG

NcHMA4-2-SLM AGAGGGAAATGTTGA---GATGCAGATT------------CTAA---ATTTGGAGAAAGG

NcHMA4-3-SLM AGAGGGAAATGTTGA---GATGCAGATT------------CTAA---ATTTGGAGAAAGG

NcHMA4-4-SLM AGAGGGAAATGTTGA---GATGCAGATT------------CTAA---ATTTGGAGAAAGG

AhHMA4-1 TGAGGAAATTGTTCTCCCGGTGCAGATGATTGGTCAGGCATTAACTGGTTTGGAAATAGA

AhHMA4-2 TGAGGAAATTGTTCTCCCGGTGCAGATGATTGGTCAGGCATTAACTGGTTTGGAAATAGA

AhHMA4-3 TGAGGAAATTGTTCTCCCGGTGCAGATGATTGGTCAGGCATTAACTGGTTTGGAAATAGA

AtHMA4 GTTGCAGACAAAG------GAAACCTGCAAAACAAGCTGTTGTGACAGTAAAGAAAAGGT

NcHMA4-1-LC GTCGCAGAAAAAGGTTGGTGAAACCTGCAAATCAAGCTGTTGTGGAGATAAAGAGAAGGC

NcHMA4-2-LC GTCGCAGAAAAAGGTTGGTGAAACCTGCAAATCAAGCTGTTGTGGAGATAAAGAGAAGGC

NcHMA4-1-Ga GTCGCAGAAAAAGGTTGGTGAAACCTGCAAATCAAGCTGTTGTGGAGATAAAGAGAAGGC

NcHMA4-2-Ga GTCGCAGAAAAAGGTTGGTGAAACCTGCAAATCAAGCTGTTGTGGAGATAAAGAGAAGGC

NcHMA4-3-Ga GTCGCAGAAAAAGGTTGGTGAAACCTGCAAATCAAGCTGTTGTGGAGATAAAGAGAAGGC

NcHMA4-1-CMA GTCGCAGAAAAAGGTTGGTGAAACCTGCAAATCAAGCTGTTGTGGAGATAAAGAGAAGGC

NcHMA4-2-CMA GTCGCAGAAAACGGTTGGTGAAACCTGCAAATCAAGCTGTTGTGGAGATAAAGAGAAGGC

NcHMA4-1-SLM GTCGCAGAAAAAGGTTGGTGAAACCTGCAAATCAAGCTGTTGTGGAGATAAAGAGAAGGC

NcHMA4-2-SLM GTCGCAGAAAAAGGTTGGTGAAACCTGCAAATCAAGCTGTTGTGGAGATAAAGAGAAGGC

NcHMA4-3-SLM GTCGCAGAAAAAGGTTGGTGAAACCTGCAAATCAAGCTGTTGTGGAGATAAAGAGAAGGC

NcHMA4-4-SLM GTCGCAGAAAAAGGTTGGTGAAACCTGCAAATCAAGCTGTTGTGGAGATAAAGAGAAGGC

AhHMA4-1 GTTGCAGACAAAG------GAAACTTGCAAAACAAGATGTTGTGACAATAAAGAGAAGGC

AhHMA4-2 GTTGCAGACAAAG------GAAACTTGCAAAACAAGATGTTGTGACAATAAAGAGAAGGC

AhHMA4-3 GTTGCAGACAAAG------GAAACTTGCAAAACAAGATGTTGTGACAATAAAGAGAAGGC

AtHMA4 TAAGGAGACAGGTTTGCTGCTTTCTAGTGAGAACACACCTTACCTGGAGAAAGGAGTGCT

NcHMA4-1-LC TAAGGAAACACGTTTGTTGCTTGCTAGTGAGGATCCATCTTATCTGGAGAAGGAAG----

NcHMA4-2-LC TAAGGAAACACGTTTGTTGCTTGCTAGTGAGGATCCATCTTATCTGGAGAAGGAAG----

NcHMA4-1-Ga TAAGGAAACACGTTTGTTGCTGGCTAGTGAAGATCCATCTTATCTGGAGAAGGAAG----

NcHMA4-2-Ga TAAGGAAACACGTTTGTTGCTGGCTAGTGAAGATCCATCTTATCTGGAGAAGGAAG----

NcHMA4-3-Ga TAAGGAAACACGTTTGTTGCTTGCTAGTGAGGATCCATCTTATCTGGAGAAGGAAG----

NcHMA4-1-CMA TAAGGAAACACGTTTGTTGCTTGCTAGTGAGGATCCATCTTATCTGGAGAAGGAAG----

NcHMA4-2-CMA TAAGGAAACACGTTTGTTGCTTGCTAGTGAGGATCCATCTTATCTGGAGAAGGAG-----

NcHMA4-1-SLM TAAGGAAACACGTTTGTTGCTGGCTAGTGAAGATCCATCTTATCTGGAGAAGGAAG----

NcHMA4-2-SLM TAAGGAAACACGTTTGGTGCTTGCTAGTGAGGATCCATCTTATCTGGAGAAGGAAG----

NcHMA4-3-SLM TAAGGAAACACGTTTGTTGCTTGCTAGTGAGGATCCATCTTATCTGGAGAAGGAG-----

NcHMA4-4-SLM TAAGGAAACACGTTTGTTGCTTGCTAGTGAGGATCCATCTTATCTGGAGAAGGAG-----

AhHMA4-1 TAAGAAAAAAGGTTTGTTGCTTTCTAGTGAGGACACATCTTACCTGGAGAAGGGAGTGCT

AhHMA4-2 TAAGAAAAAAGGTTTGTTGCTTTCTAGTGAGGACACATCTTACCTGGAGAAGGGAGTGCT

AhHMA4-3 TAAGAAAAAAGGTTTGTTGCTTTCTAGTGAGGACACATCTTACCTGGAGAAGGGAGTGCT

AtHMA4 GATTAAAGATGAAGGAAACTGCAAGTCTGGCAGCGAGAACATGGGGACAGTGAAACAAAG

NcHMA4-1-LC ----AAAGGCAAACTA--CTG-AAGCT----------AACATTGTGACAGTGAAACAGAG

NcHMA4-2-LC ----AAAGGCAAACTA--CTG-AAGCT----------AACATTGTGACAGTGAAACAGAG

NcHMA4-1-Ga ----AAAGGCAAACTA--CTG-AAGCT----------AACATTGTGACAGTGAAACAGAG

NcHMA4-2-Ga ----AAAGGCAAACTA--CTG-AAGCT----------AACATTGTGACAGTGAAACAGAG

NcHMA4-3-Ga ----AAAGGCAAACTA--CTG-AAGCT----------AACATTGTGACAGTGAAACAGAG

NcHMA4-1-CMA ----AAAGGCAAACTA--CTG-AAGCT----------AACATTGTGACAGTGAAACAGAG

NcHMA4-2-CMA ------------------------------------------------------------

NcHMA4-1-SLM ----AAAGGCAAACTA--CTG-AAGCT----------AACATTGTGACAGTGAAACAGAG

NcHMA4-2-SLM ----AAAGGCAAACTA--CTG-AAGCT----------AACATTGTGACAGTGAAACAGAG

NcHMA4-3-SLM ------------------------------------------------------------

NcHMA4-4-SLM ------------------------------------------------------------

AhHMA4-1 GATTAAAGATGAAGGAAACTGCAAGTCTGCCTGCCAGAAAACGGGGACAGTGAAAGAAAG

AhHMA4-2 GATTAAAGATGAAGGAAACTGCAAGTCTGCCTGCCAGAAAACGGGGACAGTGAAACAAAG

AhHMA4-3 GATTAAAGATGAAGGAAACTGCAAGTCTGCCTGCCAGAAAACGGGGACAGTGAAACAAAG

AtHMA4 CTGCCATGAGAAGG--GCTGCAGCGATGAAAAACAAA----CCGGGGAAAT--AACTCTT

NcHMA4-1-LC CTGCCATGAGAAGGCAAGTCTGGACATTGAAACTGGAGTTACTTGTGATCTCAAGTTGGT

NcHMA4-2-LC CTGCCATGAGAAGGCAAGTCTGGACATTGAAACTGGAGTTACTTGTGATCTCAAGTTGGT

NcHMA4-1-Ga CTGCCATGAGAAGGCAAGTCTGGACATTGAAACTGGAGTTACTTGTGATCTCAAGTTGGT

NcHMA4-2-Ga CTGCCATGAGAAGGCAAGTCTGGACATTGAAACTGGAGTTACTTGTGATCTCAAGTTGGT

NcHMA4-3-Ga CTGCCATGAGAAGGCAAGTCTGGACATTGAAACTGGAGTTACTTGTGATCTCAAGTTGGT

NcHMA4-1-CMA CTGCCATGAGAAGGCAAGTCTGGACATTGAAACTGGAGTTACTTGTGATCTCAAGTTGGT

NcHMA4-2-CMA ------------------------------------------------------------

NcHMA4-1-SLM CTGCCATGAGAAGGCAAGTCTGGACATTGAAACTGGAGTTACTTGTGATCTCAAGTTGGT

NcHMA4-2-SLM CTGCCATGAGAAGGCAAGTCTGGACATTGAAAATGGAGTTACTTGTGATCTCAAGTTGGT

NcHMA4-3-SLM ------------------------------------------------------------

NcHMA4-4-SLM ------------------------------------------------------------

AhHMA4-1 CTGCCATGAGAAGGCACCTCTTGATATAGAAACCAAG----TTGGT----T--AGTTGTG

AhHMA4-2 CTGCCATGAGAAGGCACCTCTTGATATAGAAACCAAG----TTGGT----T--AGTTGTG

AhHMA4-3 CTGCCATGAGAAGGCACCTCTTGATATAGAAACCAAG----TTGGT----T--AGTTGTG

AtHMA4 G--CTTCGGAGGAA--GAGACAGATGATCAAGATTGCTCC-----------TCGGGATGT

NcHMA4-1-LC CTGCTGCGGAAACATAGAAGTGGGAGAGCAA-TCTGATCTTGAGAAAGGCATGAAGTTAA

NcHMA4-2-LC CTGCTGCGGAAACATAGAAGTGGGAGAGCAA-TCTGATCTTGAGAAAGGCATGAAGTTAA

NcHMA4-1-Ga CTGCTGTGGAAACATAGAAGTGGGAGAGCAA-TCTGATCTTGAGAAAGGCATGAAGTTAA

NcHMA4-2-Ga CTGCTGTGGAAACATAGAAGTGGGAGAGCAA-TCTGATCTTGAGAAAGGCATGAAGTTAA

NcHMA4-3-Ga CTGCTGTGGAAACATAGAAGTGGGAGAGCAA-TCTGATCTTGAGAAAGGCATGAAGTTAA

NcHMA4-1-CMA CTGCTGTGGAAACATAGAAGTGGGAGAGCAA-TCTGATCTTGAGAAAGGCATGAAGTTAA

NcHMA4-2-CMA ------------------------------------------------------------

NcHMA4-1-SLM CTGCTGTGGAGACATAGAAGTGGGAGAGCAA-TCTGATCTTGAGAAAGGCATGAAGTTAA

NcHMA4-2-SLM CTGCTGTGGAAACATAGAAGTGGGAGAGCAA-TCTGATCTTGAGAAAGGCATGAAGTTAA

NcHMA4-3-SLM ------------------------------------------------------------

NcHMA4-4-SLM ------------------------------------------------------------

AhHMA4-1 GAAACACAGAGGGG--GAAGTGGGAGAACAA-ACTGATC------------TGGAGATAA

AhHMA4-2 GAAACACAGAGGGG--GAAGTGGGAGAACAA-ACTGATC------------TGGAGATAA

AhHMA4-3 GAAACACAGAGGGG--GAAGTGGGAGAACAA-ACTGATC------------TGGAGATAA

AtHMA4 TGTGTGAACGAGGGAACAGTGAAACAAA--GCTTCGATGAGAAGA---------------

NcHMA4-1-LC AGGGTGAAGGAC---AATGCAAGTCTGACTGCTGCGGTGATGAAATACCTCTAGCTTCTG

NcHMA4-2-LC AGGGTGAAGGAC---AATGCAAGTCTGACTGCTGCGGTGATGAAATACCTCTAGCTTCTG

NcHMA4-1-Ga AGGGTGAAGGAC---AATGTAAGTCTGACTGCTGCGGTGATGAAATACCTCTAGCTTCTG

NcHMA4-2-Ga AGGGTGAAGGAC---AATGTAAGTCTGACTGCTGCGGTGATGAAATACCTCTAGCTTCTG

NcHMA4-3-Ga AGGGTGAAGGAC---AATGCAAGTCTGACTGCTGCGGTGATGAAATACCTCTAGCTTCTG

NcHMA4-1-CMA AGGGTGAAGGAC---AATGCAAGTCTGACTGCTGCGGTGATGAAATACCTCTAGCTTCTG

NcHMA4-2-CMA ------------------------------------------------------------

NcHMA4-1-SLM AGGGTGAAGGAC---AATGCAAGTCTGACTGCTGCGGTGATGAAATACCTCTAACTTCTG

NcHMA4-2-SLM AGGGTGAAGGAC---AATGCAAGTCTGACTGCTGCGGTGATGAAATACCTCTAGCTTCTG

NcHMA4-3-SLM ------------------------------------------------------------

NcHMA4-4-SLM ------------------------------------------------------------

AhHMA4-1 AGATTGAAGGAG---ACTGCAAGTCTGGTTGCTGCAGCGATGAAA---------------

AhHMA4-2 AGATTGAAGGAG---ACTGCAAGTCTGGTTGCTGCAGCGATGAAA---------------

AhHMA4-3 AGATTGAAGGAG---ACTGCAAGTCTGGTTGCTGCAGCGATGAAA---------------

AtHMA4 -----AGCATTCTGTGTTG----------------GTGGAGAAGGAAGG-TT--------

NcHMA4-1-LC AGGAAGACAGTGTGGATTGCTCCTCCGGATGCTGCGGAAACAAGGAGGAATTGACACAAA

NcHMA4-2-LC AGGAAGACAGTGTGGATTGCTCCTCCGGATGCTGCGGAAACAAGGAGGAATTGACACAAA

NcHMA4-1-Ga AGGAAGACAGTGTGGATTGCTCCTCCGGATGCTGCGGAAACAGGGAGGAATTAACACAAA

NcHMA4-2-Ga AGGAAGACAGTGTGGATTGCTCCTCCGGATGCTGCGGAAACAAGGAGGAATTGACACAAA

NcHMA4-3-Ga AGGAAGACAGTGTGGATTGCTCCTCCGGATGCTGCGGAAACAAGGAGGAATTGACACAAA

NcHMA4-1-CMA AGGAAGACAGTGTGGATTGCTCCTCCGGATGCTGCGGAAACAAGGAGGAATTGACACAAA

NcHMA4-2-CMA ------------------------------------------------------------

NcHMA4-1-SLM AGGAAGACAGTGTGGATTGCTCCTCCGGATGCTGCGGAAACAAGGAGGAATTAACACAAA

NcHMA4-2-SLM AGGAAGACAGTGTGGATTGCTCCTCCGGATGCTGCGGAAACAAGGAGGAATTGACACAAA

NcHMA4-3-SLM ------------------------------------------------------------

NcHMA4-4-SLM ------------------------------------------------------------

AhHMA4-1 -----AACAAACTG----G----------------GGAAATAACTCTGGCTT--------

AhHMA4-2 -----AACAAACTG----G----------------GGAAATAACTCTGGCTT--------

AhHMA4-3 -----AACAAACTG----G----------------GGAAATAACTCTGGCTT--------

AtHMA4 -TGG--ACATGGAAACT------GGTTTC---TGTTGTGATGCCAAGCTGGTTTGTTGTG

NcHMA4-1-LC TCTGTCATGAGAAGACATGTCTGGACATTGTAAGTTGTGATTCCAAGTTGGTTTGCTGTG

NcHMA4-2-LC TCTGTCATGAGAAGACATGTCTGGACATTGTAAGTTGTGATTCCAAGTTGGTTTGCTGTG

NcHMA4-1-Ga TCTGTCATGAGAAGACATGTCTGGACATTGTAAGTTGTGATTCCAAGTTGGTTTGCTGTG

NcHMA4-2-Ga TCTGTCATGAGAAGGCATGTCTGGACATTGTAAGTTGTGATTCCAAGTTGGTTTGTTGTG

NcHMA4-3-Ga TCTGTCATGAGAAGGCATGTCTGGACATTGTAAGTTGTGATTCCAAGTTGGTTTGTTGTG

NcHMA4-1-CMA TCTGTCATGAGAAGGCATGTCTGGACATTGTAAGTTGTGATTCCAAGTTGGTTTGTTGTG

NcHMA4-2-CMA ------------------------------------------------------------

NcHMA4-1-SLM TCTGTCATGAGAAGGCATGTCTGGACATTGTAAGTTGTGATTCCAAGTTGGTTTGTTGTG

NcHMA4-2-SLM TCTGTCATGAGAAGGCATGTCTGGACATTGTAAGTTGTGATTCCAAGTTGGTTTGTTGTG

NcHMA4-3-SLM ------------------------------------------------------------

NcHMA4-4-SLM ------------------------------------------------------------

AhHMA4-1 -CTG--AGGAAGAGACA------GACAGC---ACGGATTGTTCC---TCGGGATGTTGTA

AhHMA4-2 -CTG--AGGAAGAGACA------GACAGC---ACGGATTGTTCC---TCGGGATGTTGTA

AhHMA4-3 -CTG--AGGAAGAGGCA------GACAGC---ACGGATTGTTCC---TCGGGATGTTGTA

AtHMA4 GAAACACAGAAGGTGAAGTGAAGGAGCAATGTC---------------------------

NcHMA4-1-LC GAGAAACAGAAGTGGAAGTGAGAGAGCAATGTGATCTCAAGAAGGGTCTGCAGATAAAGA

NcHMA4-2-LC GAGAAACAGAAGTGGAAGTGAGAGAGCAATGTGATCTCAAGAAGGGTCTGCAGATAAAGA

NcHMA4-1-Ga GAGAAACAGAAGTGGAAGTGAGAGAGCAATGTGATCTCAAGAAGGGTCTGCAGATAAAGA

NcHMA4-2-Ga GAGAAACAGAAGTGGAAGTGAGAGAGCAATGTGATCTCAAGAAGGGTCTGCAGATAAAGA

NcHMA4-3-Ga GAGAAACAGAAGTGGAAGTGAGAGAGCAATGTGATCTCAAGAAGGGTCTGCAGATAAAGA

NcHMA4-1-CMA GAGAAACAGAAGTGGAAGTGAGAGAGCAATGTGATCTCAAGAAGGGTCTGCAGATAAAGA

NcHMA4-2-CMA ------------------------------------------------------------

NcHMA4-1-SLM GAGAAACAGAAGTGGAAGTGAGAGAGCAATGTGATCTCAAGAAGGGTCTGCAGATAAAGA

NcHMA4-2-SLM GAGAAACAGAAGTGGAAGTGAGAGAGCAATGTGATCTCAAGAAGGGTCTGCAGATAAAGA

NcHMA4-3-SLM ------------------------------------------------------------

NcHMA4-4-SLM ------------------------------------------------------------

AhHMA4-1 TGGACAAAGAAG---AAGTGACACAAATCTGCG---------------------------

AhHMA4-2 TGGACAAAGAAG---AAGTGACACAAATCTGCG---------------------------

AhHMA4-3 TGGACAAAGAAG---AAGTGACACAAATCTGCG---------------------------

AtHMA4 ------------------------------------------------------------

NcHMA4-1-LC ATGAAGGACAATGCAAGTCTGTTTGTTGCGGTGATGAAAAGAAAACAGAGGAGATAACT-

NcHMA4-2-LC ATGAAGGACAATGCAAGTCTGTTCGTTGCGGTGATGAAAAGAAAACAGAGGAGATAACT-

NcHMA4-1-Ga ATGAAGGACAATGCAAGTCTGTTTGTTGCGGTGATGAAAAGAAAACAGAGGAGATAACT-

NcHMA4-2-Ga ATGAAGGACAATGCGAGTCTGTTTGTTGTGGTGATGAAAAGAAAACAGAGGAGATAACTC

NcHMA4-3-Ga ATGAAGGACAATGCGAGTCTGTTTGTTGTGGTGATGAAAAGAAAACAGAGGAGATAACTC

NcHMA4-1-CMA ATGAAGGACAATGCGAGTCTGTTTGTTGTGGTGATGAAAAGAAAACAGAGGAGATAACTC

NcHMA4-2-CMA ------------------------------------------------------------

NcHMA4-1-SLM ATGAAGGACAATGCAAGTCTGTTTGTTGCGGTGATGAAAAGAAAACAGAGGAGATAACT-

NcHMA4-2-SLM ATGAAGGACAATGCGAGTCTGTTTGTTGTGGTGATGAAAAGAAAACAGAGGAGATAACTC

NcHMA4-3-SLM ------------------------------------------------------------

NcHMA4-4-SLM ------------------------------------------------------------

AhHMA4-1 ------------------------------------------------------------

AhHMA4-2 ------------------------------------------------------------

AhHMA4-3 ------------------------------------------------------------

AtHMA4 ------------------------GTCTGGAGATAAAGAAAGAAGAACATTGCAAGTCTG

NcHMA4-1-LC -----------GAAGAGACGGACAATCTGAAAAGTGAAAGTGGTGATGATTGCAAATCTC

NcHMA4-2-LC -----------GAAGAGACGGACAATCTGAAAAGTGAAAGTGGTGATGATTGCAAATCTC

NcHMA4-1-Ga -----------GAAGAGACGGACAATCTGAAAAGTGAAAGTGGTGATGATTGCAAATCTC

NcHMA4-2-Ga TGGTTTCTGATGAAGAGACGGACAATCTGAAAAGTGAAAGTGGTGGCGATAGCAAAGCTC

NcHMA4-3-Ga TGGTTTCTGATGAAGAGACGGACAATCTGAAAAGTGAAAGTGGTGGCGATAGCAAAGCTC

NcHMA4-1-CMA TGGTTTCTGATGAAGAGACGGACAATCTGAAAAGTGAAAGTGGTGGCGATAGCAAAGCTC

NcHMA4-2-CMA -----------------------AATCTGAAAAGTGAAAGTGGTGATGATTGCAAATCTC

NcHMA4-1-SLM -----------GAAGAGACGGACAATCTGAAAAGTGAAAGTGGTGATGATTGCAAATCTC

NcHMA4-2-SLM TGGTTTCTGATGAAGAGACGGACAATCTGAAAAGTGAAAGTGGTGGCGATAGCAAAGCTC

NcHMA4-3-SLM -----------------------AATCTGAAAAGTGAAAGTGGTGATGATTGCAAATCTC

NcHMA4-4-SLM -----------------------AATCTGAAAAGTGAAAGTGGTGATGATTGCAAATCTC

AhHMA4-1 ------------------------GCTTGGAAACTGAAGGTGGTGGTGATTGCAAATCAC

AhHMA4-2 ------------------------GCTTGGAAACTGAAGGTGGTGGTGATTGCAAATCAC

AhHMA4-3 ------------------------GCTTGGAAACTGAAGGTGGTGGTGATTGCAAATCAC

AtHMA4 GTTGCTGCGG---CGAGGAAATACAAACCGGAGAAATCACTCTGGTTTCAGAGGAAGAGA

NcHMA4-1-LC TTTGTTGTGGAACTGGTTTGAAGCAA----GAAGGGTCTTC-TAGTTT----GGTCAATG

NcHMA4-2-LC TTTGTTGTGGAACTGGTTTGAAGCAA----GAAGGGTCTTC-TAGTTT----GGTCAATG

NcHMA4-1-Ga TTTGTTGTGGAACTGGTTTGAAGCAC----GAAGGGTCTTC-TAGTTT----GGTCAATG

NcHMA4-2-Ga TTTGTTGTGGAACTGGTTTGAAGCAA----GAAGGGTCTTC-TAGTTT----GGTCAATG

NcHMA4-3-Ga TTTGTTGTGGAACTGGTTTGAAGCAA----GAAGGGTCTTC-TAGTTT----GGTCAATG

NcHMA4-1-CMA TTTGTTGTGGAACTGGTTTGAAGCAA----GAAGGGTCTTC-TAGTTT----GGTCAATG

NcHMA4-2-CMA TTTGTTGTGGAACTGGTTTGAAGCAA----GAAGGGTCTTC-TAGTTT----GGTCAATG

NcHMA4-1-SLM TTTGTTGTGGAACTGGTTTGAAGCAA----GAAGGGTCTTC-TAGTTT----GGTCAATG

NcHMA4-2-SLM TTTGTTGTGGAACTGGTTTGAAGCAA----GAAGGGTCTTC-TAGTTT----GGTCAATG

NcHMA4-3-SLM TTTGTTGTGGAACTGGTTTGAAGCAA----GAAGGGTCTTC-TAGTTT----GGTCAATG

NcHMA4-4-SLM TTTGTTGTGGAACTGGTTTGAAGCAA----GAAGGGTCTTC-TAGTTT----GGTCAATG

AhHMA4-1 ATTGTTGTGGAACTGGGTTGACACAA----GAAGGGTCTTCGAAGTT-----GGGCAATG

AhHMA4-2 ATTGTTGTGGAACTGGGTTGACACAA----GAAGGGTCTTCAAAGTT-----GGGCAATG

AhHMA4-3 ATTGTTGTGGAACTGGGTTGACACAA----GAAGGGTCTTCGAAGTT-----GGGCAATG

AtHMA4 CAG----AGAGCACGAATTGTTCCACG---GGTTGTTGTGT-----------GGACAAAG

NcHMA4-1-LC TTGTGGTGGAGAGCGG-TGAATCCGGGTCAAGCTGTTGCAGCAAGGAGGGAGAGATAGTG

NcHMA4-2-LC TTGTGGTGGAGAGTGG-TGAATCCGGGTCAAGCTGTTGCAGCAAGGAGGGAGAGATAGTG

NcHMA4-1-Ga TTGTGGTGGAGAGTGG-TGAATCCGGGTCAAGCTGTTGCAGCAAGGAGGGAGAGATAGTG

NcHMA4-2-Ga TGGTGGTGGAGAGCGG-TGAATCCGGGTCAAGCTGTTGCAGCAAGGAGGGAGAGATAGTG

NcHMA4-3-Ga TGGTGGTGGAGAGCGG-TGAATCCGGGTCAAGCTGTTGCAGCAAGGAGGGAGAGATAGTG

NcHMA4-1-CMA TGGTGGTGGAGAGCGG-TGAATCCGGGTCAAGCTGTTGCAGCAAGGAGGGAGAGATAGTG

NcHMA4-2-CMA TTGTGGTGGAGAGTGG-TGAATCCGGGTCAAGCTGTTGCAGCAAGGAGGGAGAGATAGTG

NcHMA4-1-SLM TTGTGGTGGAGAGTGG-TGAATCCGGGTCAAGCTGTTGCAGCAAGGAGGGAGAGATAGTG

NcHMA4-2-SLM TGGTGGTGGAGAGCGG-TGAATCCGGGTCAAGCTGTTGCAGCAAGGAGGGAGAGATAGTG

NcHMA4-3-SLM TTGTGGTGGAGAGTGG-TGAATCCGGGTCAAGCTGTTGCAGCAAGGAGGGAGAGATAGTG

NcHMA4-4-SLM TTGTGGTGGAGAGTGG-TGAATCCGGGTCAAGCTGTTGCAGCAAGGAGGGAGAGATAGTG

AhHMA4-1 TGG----AGAGTGC---TCAATCCGGA---GGCTGTGG---------------AACAGTG

AhHMA4-2 TGG----AGAGTGC---TCAATTCGGA---GGCTGTGG---------------AACAGTG

AhHMA4-3 TGG----AGACTGC---TCAATCCGGA---GGCTGTGG---------------AACAGTG

AtHMA4 AAGAAGTG-ACACAAACCTGTCATGAGAAGCCTGCTAGCTTGGTGGTATCAGGCTTGGAA

NcHMA4-1-LC AAAGTCTCTAGCCAAAGCTGTTGCGCAAGTCCAAGTGATGTGGTGTTATCTGACTTTCAA

NcHMA4-2-LC AAAGTCTCTAGCCAAAGCTGTTGCGCAAGTCCAAGTGATGTGGTGTTATCTGACTTGGAA

NcHMA4-1-Ga AAAGTCTCTAGCCAAAGCTGTTGCACAAGTCCAAGTGATGTGGTGTTATCTGACTTGCAA

NcHMA4-2-Ga AAAGTCTCTAGCCAAAGCCGTTGCACAAGTCCAAGTGATGTGGTGTTATCTGACTTGCAA

NcHMA4-3-Ga AAAGTCTCTAGCCAAAGCCGTTGCACAAGTCCAAGTGATGTGGTGTTATCTGACTTGCAA

NcHMA4-1-CMA AAAGTCTCTAGCCAAAGCCGTTGCACAAGTCCAAGTGATGTGGTGTTATCTGACTTGCAA

NcHMA4-2-CMA AAAGTCTCTAGCCAAAGCTGTTGCACAAGTCCAAGTGATGTGGTGTTATCTGACTTTCAA

NcHMA4-1-SLM AAAGTCTCTAGCCAAAGCTGTTGCACAAGTCCAAGTGATGTGGTGTTATCTGACTTTCAA

NcHMA4-2-SLM AAAGTCTCTAGCCAAAGCCGTTGCACAAGTCCAAGTGATGTGGTGTTATCTGACTTGCAA

NcHMA4-3-SLM AAAGTCTCTAGCCAAAGCTGTTGCACAAGTCCAAGTGATGTGGTGTTATCTGACTTTCAA

NcHMA4-4-SLM AAAGTCTCTAGCCAAAGCTGTTGCACAAGTCCAAGTGATGTGGTGTTATCTGACTTTCAA

AhHMA4-1 AAAGTCTCTAGTCAAAGCTGTTGCACTAGTTCTACTGATCTGGTGCTATCTGACTTGCAA

AhHMA4-2 AAAGTCTCTAGTCAAAGCTGTTGCACTAGTTCTACTGATCTGGTGCTATCTGACTTGCAA

AhHMA4-3 AAAGTCTCTAGTCAAAGCTGTTGCACTAGTTCTACTGATCTGGTGCTATCTGACTTGCAA

AtHMA4 GTGAAGAAGGATGAGCATTGTGAGAGCTCACACAGAGCCGTCAAGGTAGAGACCTGTTGC

NcHMA4-1-LC GCTAAGAA---------------------AC---------------TAGAGATTTGTTGC

NcHMA4-2-LC GTCAAGAA---------------------AC---------------TAGAGATTTGTTGC

NcHMA4-1-Ga GCTAAGAA---------------------AC---------------TAGAGATTTGTTGC

NcHMA4-2-Ga GCTAAGAA---------------------AC---------------TAGAGATTTGTTGC

NcHMA4-3-Ga GCTAAGAA---------------------AC---------------TAGAGATTTGTTGC

NcHMA4-1-CMA GCTAAGAA---------------------AC---------------TAGAGATTTGTTGC

NcHMA4-2-CMA GCTAAGAA---------------------AC---------------TAGAGATTTGTTGC

NcHMA4-1-SLM GCTAAGAA---------------------AC---------------TAGAGATTTGTTGC

NcHMA4-2-SLM GCTAAGAA---------------------AC---------------TAGAGATTTGTTGC

NcHMA4-3-SLM GCTAAGAA---------------------AC---------------TAGAGATTTGTTGC

NcHMA4-4-SLM GCTAAGAA---------------------AC---------------TAGAGATTTGTTGC

AhHMA4-1 GTGAAGAAGGATGAGCACTGTAAGAGCTCACACGGAGCCGTCAAGGTAGAGACCTGTTGC

AhHMA4-2 GTGAAGAAGGATGAGCATTGTGAGAGCTCACACGGAGCCGTCAAGGTAGAGACCTGTTGC

AhHMA4-3 GTGACGAAGGATGAGCATTGTGAGAGCTCACACGGAGCCGTCAAGGTAGAGACCTGTTGC

AtHMA4 AAAGTGAAGA---TTCC---AGAGGCTTGCGCATCAAAATGTAGGGACAGAGCGAAGCGT

NcHMA4-1-LC GAAGTGAAGAAGACTCCAGAGGAGGTTTGTGGATCTAAATGTAAGGAAACAGAGAAGCGT

NcHMA4-2-LC AAAGCGAAGAAGACTCCAGAGGAGGTTCGTGGATCTAAATGTAAGGAAACAGAGAAGCGT

NcHMA4-1-Ga AAAGTGAAGAAGACTCCAGAGGAGGTTTGTGGATCTAAATGTAAGGCAACAGAGAAGCCT

NcHMA4-2-Ga AAAGTGAAGAAGACTCTTGAGGAGGTTCGTGGATCTAAATGTAAGGAAACAGAGAAGCCT

NcHMA4-3-Ga AAAGTGAAGAAGACTCTTGAGGAGGTTCGTGGATCTAAATGTAAGGAAACAGAGAAGCCT

NcHMA4-1-CMA AAAGTGAAGAAGACTCTTGAGGAGGTTCGTGGATCTAAATGTAAGGAAACAGAGAAGCCT

NcHMA4-2-CMA GAAGTGAAGAAGACTCCAGAGGAGGTTTGTGGATCTAAATGTAAGGAAACAGAGAAGCCT

NcHMA4-1-SLM AAAGTGAAGAAGACTCCAGAGGAGGTTTGTGGATCTAAATGTAAGGCAACAGAGAAGCCT

NcHMA4-2-SLM AAAGTGAAGAAGACTCTTGAGGAGGTTCGTGGATCTAAATGTAAGGAAACAGAGAAGCCT

NcHMA4-3-SLM GAAGTGAAGAAGACTCCAGAGGAGGTTTGTGGATCTAAATGTAAGGAAACAGAGAAGCCT

NcHMA4-4-SLM GAAGTGAAGAAGACTCCAGAGGAGGTTTGTGGATCTAAATGTAAGGAAACAGAGAAGCCT

AhHMA4-1 AAAGTGAAGA---TTCC---AGAGGCTTGTGCATCGGAATGTAAGGAAAAAGAGAAGCGT

AhHMA4-2 AAAGTGAAGA---TTCC---AGAGGCTTGTGCACCGGAATGTAAGGAAAAAGAGAAGCGT

AhHMA4-3 AAAGTGAAGA---TTCC---AGAGGCTTGTGCACCGGAATGTAAGGAAAAAGAGAAGCGT

AtHMA4 CAC---AGTGGTAAAAGCTGTTGCAGGAGTTATGCAAAAGAGTTATGCAGCCACCGCCAT

NcHMA4-1-LC CACCACGTTGGTAAAAGCTGTTGCAGGAGTTATGCAAAAGAGCATTGCAGCCACAGGCAT

NcHMA4-2-LC CACCACGTTGGTAAAAGCTGTTGCAGGAGTTATGCAAAAGAGTATTGCAGCCACAGGCAT

NcHMA4-1-Ga CACCACGTCGGTAAAAGCTGTTGCAGGAGTTATGCAAAAGAGTATTGCAGCCACAGGCAT

NcHMA4-2-Ga CACCACGTTGGTAAAAGCTGTTGCAGGAGTTATGCAAAAGAGTATTGCAGCCACAGGCAT

NcHMA4-3-Ga CACCACGTTGGTAAAAGCTGTTGCAGGAGTTATGCAAAAGAGTATTGCAGCCACAGGCAT

NcHMA4-1-CMA CACCACGTTGGTAAAAGCTGTTGCAGGAGTTATGCAAAAGAGTATTGCAGCCACAGGCAT

NcHMA4-2-CMA CACCACGTCGGTAAAAGCTGTTGCAGGAGTTATGCAAAAGAGTATTGCAGCCACAGGCAT

NcHMA4-1-SLM CACCACGTCGGTAAAAGCTGTTGCAGGAGTTATGCAAAAGAGTATTGCAGCCACAGGCAT

NcHMA4-2-SLM CACCACGTTGGTAAAAGCTGTTGCAGGAGTTATGCAAAAGAGTATTGCAGCCACAGGCAT

NcHMA4-3-SLM CACCACGTTGGTAAAAGCTGTTGCAGGAGTTATGCAAAAGAGTATTGCAGCCACAGGCAT

NcHMA4-4-SLM CACCACGTTGGTAAAAGCTGTTGCAGGAGTTATGCAAAAGAGTATTGCAGCCACAGGCAT

AhHMA4-1 CAC---AGTGGTAAAAGCTGTTGCAGGAGTTATGCAAAAGAGTTTTGCAGCCACCGCCAC

AhHMA4-2 CAC---AGTGGTAAAAGCTGTTGCAGGAGTTATGCAAAAGAGTTTTGCAGCCACCGCCAC

AhHMA4-3 CAC---AGTGGTAAAAGCTGTTGCAGGAGTTATGCAAAAGAGTTTTGCAGCCACCGCCAC

AtHMA4 CATCATCACCACCACCACCACCATCACCATGTGAGTGCTTGA

NcHMA4-1-LC CACCACCACCACCACCACCACCACCATGTTGGGGCTGCTTGA

NcHMA4-2-LC CACCACCACCACCACCACCAC---CATGTTGGGGCTGCTTGA

NcHMA4-1-Ga CACGACAACCA---------------TGTTGGGGCTGCTTGA

NcHMA4-2-Ga CACGACAACCA---------------TGTTGGGGCTGCTTGA

NcHMA4-3-Ga CACCACCACCACCACCACCAC---CATGTCGGGGCTGCTTGA

NcHMA4-1-CMA CACCACCACCACCACCACCAC---CATGTCGGGGCTGCTTGA

NcHMA4-2-CMA CACGACAACCATCATCACCAC---CATGTTGGGGCTGCTTGA

NcHMA4-1-SLM CACGACAACCATCATCACCAC---CATGTTGGGGCTGCTTGA

NcHMA4-2-SLM CACCACCACCACCACCACCAC---CATGTCGGGGCTGCTTGA

NcHMA4-3-SLM CACGACAACCATCATCACCAC---CATGTTGGGGCTGCTTGA

NcHMA4-4-SLM CACGACAACCATCATCACCAC---CATGTTGGGGCTGCTTGA

AhHMA4-1 CACCA---CCACCACCA------TCACCATGTGAGTGCTTGA

AhHMA4-2 CACCA---CCACCACCA------TCACCATGTGAGTGCTTGA

AhHMA4-3 CACCA---CCACCACCACCACCATCACCATGTGAGTGCTTGA

**Supplementary Alignment S1.** Coding sequence alignment of *HMA4*s from *A. thaliana*, *N. caerulescens* accessions (La Calamine, Ganges, Col du Mas de l’Aire and Saint Laurent Le Minier) and *A. halleri*

AtHMA4 MA----LQNKEEEKKKVKKLQKSYFDVLGICCTSEVPIIENILKSLDGVKEYSVIVPSRT

NcHMA4-1-LC MALQKEIKNKEEDKKTKKKWQKSYFDVLGICCTSEIPLIENILKSLDGVKEYTVIVPSRT

NcHMA4-2-LC MALQKEIKNKEEDKKTKKKWQKSYFDVLGICCTSEIPLIENILKSLDGVKEYTVIVPSRT

NcHMA4-1-Ga MALQKEIKNKEENKMTKKKWQKSYFDVLGLCCTSEIPLIENILKSLDGIKDYTIIVPSRT

NcHMA4-2-Ga MALQKEDKNKEENKMTKKTWQKSYFDVLGLCCTSEIPLIENILKSLDGIKDYTIIVPSRT

NcHMA4-3-Ga MASQKEIKNKEEDKKTKKKWQKSYFDVLGICCTSEIPVIENILKSLDGVKEYTVIVPSRT

NcHMA4-1-CMA MASQKEIKNKEEDKKTKKKWQKSYFDVLGICCTSEIPVIENILKSLDGVKEYTVIVPSRT

NcHMA4-2-CMA MATQKEDKNKEENKMTKKKWQKSYFDVLGICCTSEIPLIENILKSLDGIKDYTIIVPSRT

NcHMA4-1-SLM MALQKEDKNKEENKMTKKKWQKSYFDVLGICCTSEIPLIENILKSLDGIKDYTIIVPSRT

NcHMA4-2-SLM MALQKEIKNKEEDKKTKKKWQKSYFDVLGICCTSEIPVIENILKSLDGVKEYTVIVPSRT

NcHMA4-3-SLM MALQKEDKNKEENKMTKKKWQKSYFDVLGICCTSEIPLIENILKSLDGIKDYTIIVPSRT

NcHMA4-4-SLM MALQKEDKNKEENKMTKKKWQKSYFDVLGICCTSEIPLIENILKSLDGIKDYTIIVPSRT

AhHMA4-1 MA----SQNKEEEKKKVKKLQKSYFDVLGICCTSEVPIIENILKSLDGVKEYSVIVPSRT

AhHMA4-2 MA----SQNKEEEKKKVKKLQKSYFDVLGICCTSEVPIIENILKSLDGVKEYSVIVPSRT

AhHMA4-3 MA----SQNKEEEKKKVKKLQKSYFDVLGICCTSEVPIIENILKSLDGVKEYSVIVPSRT

AtHMA4 VIVVHDSLLISPFQIAKALNEARLEANVRVNGETSFKNKWPSPFAVVSGLLLLLSFLKFV

NcHMA4-1-LC VIVVHDSLLISPFQIAKALNQARLEANVKVNGETSFKNKWPSPFAVVSGIFLLLSFLKFV

NcHMA4-2-LC VIVVHDSLLISPFQIAKALNQARLEANVKVNGETSFKNKWPSPFAVVSGIFLLLSFLKFV

NcHMA4-1-Ga VIVVHDSLLISPFQIAKALNQARLEANVKVDGETSFKNKLPSPFAVFSGIFLLLSFLKFV

NcHMA4-2-Ga VIVVHDSLLISPFQIAKALNQARLEANVKVDGETSFKNKLPSPFAVFSGIFLLLSFLKFV

NcHMA4-3-Ga VIVVHDSLLISPFQIAKALNQARLEANVKVNGETSFKNKWPSPFAVVSGIFLLLSFLKFV

NcHMA4-1-CMA VIVVHDSLLISPFQIAKALNQARLEANVKVNGETSFKNKWPSPFAVVSGIFLLLSFLKFV

NcHMA4-2-CMA VIVVHDSLLISPFQIAKALNQARLEANVKVDGETSFKNKLPSPFAVFSGIFLLLSFLKFV

NcHMA4-1-SLM VIVVHDSLLISPFQIAKALNQARLEANVKVDGETSFKNKLPSPFAVFSGIFLLLSFLKFV

NcHMA4-2-SLM VIVVHDSLLISPFQIAKALNQARLEANVKVNGETSFKNKWPSPFAVVSGIFLLLSFLKFV

NcHMA4-3-SLM VIVVHDSLLISPFQIAKALNQARLEANVKVDGETSFKNKLPSPFAVFSGIFLLLSFLKFV

NcHMA4-4-SLM VIVVHDSLLISPFQIAKALNQARLEANVKVDGETSFKNKLPSPFAVFSGIFLLLSFLKFV

AhHMA4-1 VIVVHDSLLISPFQIAKALNQARLEANVRVNGETNFKNKWPSPFAVVSGLLLLLSFLKFV

AhHMA4-2 VIVVHDSLLISPFQIAKALNQARLEANVRVNGETNFKNKWPSPFAVVSGILLLLSFLKFV

AhHMA4-3 VIVVHDSLLISPFQIAKALNQARLEANVRVNGETNFKNKWPSPFAVVSGILLLLSFLKFV

AtHMA4 YSPLRWLAVAAVAAGIYPILAKAFASIKRPRIDINILVIITVIATLAMQDFMEAAAVVFL

NcHMA4-1-LC YPPLRWLAVVGVAAGIYPILAKAVASIRRLRVDINILIIITVAATLAMQDYMEAAAVVFL

NcHMA4-2-LC YPPLRWLAVVGVAAGIYPILAKAVASIRRLRVDINILIIITVAATLAMQDYMEAAAVVFL

NcHMA4-1-Ga YPPLRWLAVVGVATGIYPILAKSVASIRRLRVDINILVIITVAATLAMQDYMEAAAVVFL

NcHMA4-2-Ga YPPLRWLAVVGVATGIYPILAKSVASIRRLRVDINILVIITVAATLAMQDYMEAAAVVFL

NcHMA4-3-Ga YPPLRWLAVVGVAAGIYPILAKSVASIRRLRVDINILIIITVAATLAMQDYMEAAAVVFL

NcHMA4-1-CMA YPPLRWLAVVGVAAGIYPILAKSVASIRRLRVDINILIIITVAATLAMQDYMEAAAVVFL

NcHMA4-2-CMA YPPLRWLAVVGVATGIYPILAKSVASIRRLRVDINILVIITVAATLAMQDYMEAAAVVFL

NcHMA4-1-SLM YPPLRWLAVVGVATGIYPILAKSVASIRRLRVDINILVIITVAATLAMQDYMEAAAVVFL

NcHMA4-2-SLM YPPLRWLAVVGVAAGIYPILAKSVASIRRLRVDINILIIITVAATLAMQDYMEAAAVVFL

NcHMA4-3-SLM YPPLRWLAVVGVATGIYPILAKSVASIRRLRVDINILVIITVAATLAMQDYMEAAAVVFL

NcHMA4-4-SLM YPPLRWLAVVGVATGIYPILAKSVASIRRLRVDINILVIITVAATLAMQDYMEAAAVVFL

AhHMA4-1 YSPLRWLAVAAVAAGIYPILAKAFASIRRPRIDINILVIITVIATLAMQDFMEAAAVVFL

AhHMA4-2 YSPLRWVAVAAVAAGIYPILAKAFASIRRLRLDINILVIITVIATLAMQDFMEAAAVVFL

AhHMA4-3 YPPLRWLAVVAVAAGIYPILAKAFASIRRLRLDINILVIITVIATLAMQDFMEAAAVVFL

AtHMA4 FTISDWLETRASYKATSVMQSLMSLAPQKAIIAETGEEVEVDEVKVDTVVAVKAGETIPI

NcHMA4-1-LC FTIADWLETRASYKANSVMQSLMSLAPQKAVIAETGEEVEVDEVQLNTIIAVKAGETIPI

NcHMA4-2-LC FTIADWLETRASYKANSVMQSLMSLAPQKAVIAETGEEVEVDEVQLNTIIAVKAGETIPI

NcHMA4-1-Ga FTIADWLETRASYKASSVMQSLMSLAPQKAVIAETGEEVEVDEVELNTIIAVKAGETIPI

NcHMA4-2-Ga FTIADWLETRASYKASSVMQSLMSLAPQKAVIAETGEEVEVDEVELNTIIAVKAGETIPI

NcHMA4-3-Ga FTIADWLETRASYKANSVMQSLMSLAPQKAVIAETGEEVEVDEVQLNTIIAVKAGETIPI

NcHMA4-1-CMA FTIADWLETRASYKANSVMQSLMSLAPQKAVIAETGEEVEVDEVQLNTIIAVKAGETIPI

NcHMA4-2-CMA FTIADWLETRASYKASSVMQSLMSLAPQKAVIAETGEEVEVDEVELNTIIAVKAGETIPI

NcHMA4-1-SLM FTIADWLETRASYKASSVMQSLMSLAPQKAVIAETGEEVEVDEVELNTIIAVKAGETIPI

NcHMA4-2-SLM FTIADWLETRASYKANSVMQSLMSLAPQKAVIAETGEEVEVDEVQLNTIIAVKAGETIPI

NcHMA4-3-SLM FTIADWLETRASYKASSVMQSLMSLAPQKAVIAETGEEVEVDEVELNTIIAVKAGETIPI

NcHMA4-4-SLM FTIADWLETRASYKASSVMQSLMSLAPQKAVIAETGEEVEVDEVELNTIIAVKAGETIPI

AhHMA4-1 FTIADWLETRASYRATAVMQSLMSLAPQKAIIAETGEEVEVDEVKVSTVVAVKAGETIPI

AhHMA4-2 FTIADWLETRASYRATAVMQSLMSLAPQKAIIAETGEEVEVDEVKVSTVVAVKAGETIPI

AhHMA4-3 FTIADWLETRASYRATAVMQSLMSLAPQKAIIAETGEEVEVDEVKVSTVVAVKAGETIPI

AtHMA4 DGIVVDGNCEVDEKTLTGEAFPVPKQRDSTVWAGTINLNGYICVKTTSLAGDCVVAKMAK

NcHMA4-1-LC DGIVVDGNCEVDEKTLTGEAFPVPKQRDSTVLAGTMNLNGYISVNTTALASDCVVAKMAK

NcHMA4-2-LC DGIVVDGNCEVDEKTLTGEAFPVPKQRDSTVLAGTMNLNGYISVNTTALASDCVVAKMAK

NcHMA4-1-Ga DGIVVDGNCEVDEKTLTGEAFPVPKQRDSTVWAGTINLNGYISVNTTALASDCVVAKMAK

NcHMA4-2-Ga DGIVVDGNCEVDEKTLTGEAFPVPKQRDSTVWAGTINLNGYISVNTTALASDCVVAKMAK

NcHMA4-3-Ga DGIVVDGNCEVDEKTLTGEAFPVPKQRDSTVLAGTMNLNGYISVNTTALASDCVVAKMAK

NcHMA4-1-CMA DGIVVDGNCEVDEKTLTGEAFPVPKQRDSTVLAGTMNLNGYISVNTTALASDCVVAKMAK

NcHMA4-2-CMA DGIVVDGNCEVDEKTLTGEAFPVPKQRDSTVWAGTINLNGYISVNTTALASDCVVAKMAK

NcHMA4-1-SLM DGIVVDGNCEVDEKTLTGEAFPVPKQRDSTVWAGTINLNGYISVNTTALASDCVVAKMAK

NcHMA4-2-SLM DGIVVDGNCEVDEKTLTGEAFPVPKQRDSTVLAGTINLNGYISVNTTALASDCVVAKMAK

NcHMA4-3-SLM DGIVVDGNCEVDEKTLTGEAFPVPKQRDSTVWAGTINLNGYISVNTTALASDCVVAKMAK

NcHMA4-4-SLM DGIVVDGNCEVDEKTLTGEAFPVPKQRDSTVWAGTINLNGYISVNTTALASDCVVAKMAK

AhHMA4-1 DGIVVDGNCEVDEKTLTGEAFPVPKQKDSSVWAGTINLNGYISVKTTSLAGDCVVAKMAK

AhHMA4-2 DGIVVDGNCEVDEKTLTGEAFPVPKQKDSSVWAGTINLNGYISVKTTSLAGDCVVAKMAK

AhHMA4-3 DGIVVDGNCEVDEKTLTGEAFPVPKQKDSSVWAGTINLNGYISVKTTSLAGDCVVAKMAK

AtHMA4 LVEEAQSSKTKSQRLIDKCSQYYTPAIILVSACVAIVPVIMKVHNLKHWFHLALVVLVSG

NcHMA4-1-LC LVEEAQGSKTKSQRLIDKCSQYYTPAIIIISAGFAIVPAIMKVHNLNHWFHLALVVLVSA

NcHMA4-2-LC LVEEAQGSKTKSQRLIDKCSQYYTPAIIIISAGFAIVPAIMKVHNLNHWFHLALVVLVSA

NcHMA4-1-Ga LVEEAQSSKTKSQRLIDKYSQYYTPAIIIISAGFAIVPLIMKVRNLNHWFHLALVVLVSA

NcHMA4-2-Ga LVEEAQSSKTKSQRLIDKYSQYYTPAIIIISAGFAIVPLIMKVRNLNHWFHLALVVLVSA

NcHMA4-3-Ga LVEEAQSSKTKSQRLIDXCSQYYTPAIIIISAGFAIVPAIMKVRNLNHWFHLALVVLVSA

NcHMA4-1-CMA LVEEAQSSKTKSQRLIDKCSQYYTPAIIIISAGFAIVPAIMKVRNLNHWFHLALVVLVSA

NcHMA4-2-CMA LVEEAQSSKTKSQRLIDKYSQYYTPAIIIISAGFAIVPLIMKVRNLNHWFHLALVVLVSA

NcHMA4-1-SLM LVEEAQSSKTKSQRLIDKYSQYYTPAIIIISAGFAIVPAIMKVRNLNHWFHLALVVLVSA

NcHMA4-2-SLM LVEEAQSSKTKSQRLIDKCSQYYTPAIIIISAGFAIVPAIMKVRNLNHWFHLALVVLVSA

NcHMA4-3-SLM LVEEAQSSKTKSQRLIDKYSQYYTPAIIIISAGFAIVPAIMKVRNLNHWFHLALVVLVSA

NcHMA4-4-SLM LVEEAQSSKTKSQRLIDKYSQYYTPAIIIISAGFAIVPAIMKVRNLNHWFHLALVVLVSA

AhHMA4-1 LVEEAQSSKTKSQRLIDKCSQYYTPAIIVVSACVAIVPVIMKVHNLKHWFHLALVVLVSG

AhHMA4-2 LVEEAQSSKTKSQRLIDKCSQYYTPAIIVVSACVAIVPVIMKVHNLKHWFHLALVVLVSG

AhHMA4-3 LVEEAQSSKTKSQRLIDKCSQYYTPAIIVVSACVAIVPVIMKVHNLKHWFHLALVVLVSG

AtHMA4 CPCGLILSTPVATFCALTKAATSGLLIKSADYLDTLSKIKIVAFDKTGTITRGEFIVIDF

NcHMA4-1-LC CPCGLILSTPVATFCALTKAATSGLLIKSADYLDTLSKIKIAAFDKTGTITRGEFIVIEF

NcHMA4-2-LC CPCGLILSTPVATFCALTKAATSGLLIKSADYLDTLSKIKIAAFDKTGTITRGEFIVIEF

NcHMA4-1-Ga CPCGLILSTPVATFCALTKAATSGLLIKSADYLDTLSKIKIAAFDKTGTITRGEFIVIEF

NcHMA4-2-Ga CPCGLILSTPVATFCALTKAATSGLLIKSADYLDTLSKIKIAAFDKTGTITRGEFIVIEF

NcHMA4-3-Ga CPCGLILSTPVATFCALTKAATSGLLIKSADYLDTLSKIKIAAFDKTGTITRGEFIVIEF

NcHMA4-1-CMA CPCGLILSTPVATFCALTKAATSGLLIKSADYLDTLSKIKIAAFDKTGTITRGEFIVIEF

NcHMA4-2-CMA CPCGLILSTPVATFCALTKAATSGLLIKSADYLDTLSKIKIAAFDKTGTITRGEFIVIEF

NcHMA4-1-SLM CPCGLILSTPVATFCALTKAATSGLLIKSADYLDTLSKIKIAAFDKTGTITRGEFIVIEF

NcHMA4-2-SLM CPCGLILSTPVATFCALTKAATSGLLIKSADYLDTLSKIKIAAFDKTGTITRGEFIVIEF

NcHMA4-3-SLM CPCGLILSTPVATFCALTKAATSGLLIKSADYLDTLSKIKIAAFDKTGTITRGEFIVIEF

NcHMA4-4-SLM CPCGLILSTPVATFCALTKAATSGLLIKSADYLDTLSKIKIAAFDKTGTITRGEFIVIEF

AhHMA4-1 CPCGLILSTPVATFCALTKAATSGLLIKSADYLDTLSKIKIAAFDKTGTITRGEFIVIDF

AhHMA4-2 CPCGLILSTPVATFCALTKAATSGLLIKSADYLDTLSKIKIAAFDKTGTITRGEFIVIDF

AhHMA4-3 CPCGLILSTPVATFCALTKAATSGLLIKSADYLDTLSKIKIAAFDKTGTITRGEFIVIDF

AtHMA4 KSLSRDINLRSLLYWVSSVESKSSHPMAATIVDYAKSVSVEPRPEEVEDYQNFPGEGIYG

NcHMA4-1-LC KSLSRDISLRSLLYWVSSVESKSSHPMAATIVDYAKSVSVEPRSEEVEDYQNFPGEGIYG

NcHMA4-2-LC KSLSRDISLRSLLYWVSSVESKSSHPMAATIVDYAKSVSVEPRSEEVEDYQNFPGEGIYG

NcHMA4-1-Ga KSLSRDISLSSLLYWVSSVESKSSHPMAATIVDYAKSVSVEPRSEEVEDYQNFPGEGIYG

NcHMA4-2-Ga KSLSRDISLSSLLYWVSSVESKSSHPMAATIVDYAKSVSVEPRSEEVEDYQNFPGEGIYG

NcHMA4-3-Ga KSLSRDISLRSLLYWVSSVESKSSHPMAATIVDYAKSVSVEPRSEEVEDYQNFPGEGIYG

NcHMA4-1-CMA KSLSRDISLRSLLYWVSSVESKSSHPMAATIVDYAKSVSVEPRSEEVEDYQNFPGEGIYG

NcHMA4-2-CMA KSLSRDISLSSLLYWVSSVESKSSHPMAATIVDYAKSVSVEPRSEEVEDYQNFPGEGIYG

NcHMA4-1-SLM KSLSRDISLSSLLYWVSSVESKSSHPMAATIVDYAKSVSVEPRSEEVEDYQNFPGEGIYG

NcHMA4-2-SLM KSLSRDISLRSLLYWVSSVESKSSHPMAATIVDYAKSVSVEPRSEEVEDYQNFPGEGIYG

NcHMA4-3-SLM KSLSRDISLRSLLYWVSSVESKSSHPMATTIVDYAKSVSVEPRSEEVEDYHNFPGEGIYG

NcHMA4-4-SLM KSLSRDISLSSLLYWVSSVESKSSHPMAATIVDYAKSVSVEPRSEEVEDYQNFPGEGIYG

AhHMA4-1 KSLSRDISLRSLLYWVSSVESKSSHPMAATIVDYAKSVSVEPRPEEVEDYQNFPGEGIYG

AhHMA4-2 KSLSRDISLRSLLYWVSSVESKSSHPMAATIVDYAKSVSVEPRPEEVEDYQNFPGEGIYG

AhHMA4-3 KSLSRDITLRSLLYWVSSVESKSSHPMAATIVDYAKSVSVEPRPEEVEDYQNFPGEGIYG

AtHMA4 KIDGNDIFIGNKKIASRAGCSTVPEIEVDTKGGKTVGYVYVGERLAGFFNLSDACRSGVS

NcHMA4-1-LC KIDGNNVYIGNKRIASRAGCSTVPEIEVDTKKGKTVGYVYVGERLAGVFNLSDACRSGVA

NcHMA4-2-LC KIDGNNVYIGNKRIASRAGCSTVPEIEVDTKKGKTVGYVYVGERLAGVFNLSDACRSGVA

NcHMA4-1-Ga KIDGNNVYIGNKRIASRAGCSTVPEIDVDTKKGKTVGYVYVGERLAGVFNLSDACRSGVA

NcHMA4-2-Ga KIDGNNVYIGNKRIASRAGCSTVPEIDVDTKKGKTVGYVYVGERLAGVFNLSDACRSGVA

NcHMA4-3-Ga KIDGNNVYIGNKRIASRAGCSTVPEIDVDTKKGKTVGYVYVGERLAGVFNLSDACRSGVA

NcHMA4-1-CMA KIDGNNVYIGNKRIASRAGCSTVPEIDVDTKKGKTVGYVYVGERLAGVFNLSDACRSGVA

NcHMA4-2-CMA KIDGNNVYIGNKRIASRAGCSTVPEIDVDTKKGKTVGYVYVGERLAGVFNLSDACRSGVA

NcHMA4-1-SLM KIDGNNVYIGNKRIASRAGCSTVPEIDVDTKKGKTVGYVYVGERLAGVFNLSDACRSGVA

NcHMA4-2-SLM KIDGNNVYIGNKRIASRAGCSTVPEIDVDTKKGKTVGYVYVGERLAGVFNLSDACRSGVA

NcHMA4-3-SLM KIDGNNVYIGNKRIASRAGCSTVPEIDVDTKEGKTVGYVYVDERLAGVFNLSDACRSGVA

NcHMA4-4-SLM KIDGNNVYIGNKRIASRAGCSTVPEIDVDTKEGKTVGYVYVDERLAGVFNLSDACRSGVA

AhHMA4-1 KIDGNDIYIGNKRIASRAGCSTVPEIEVDTKGGKTVGYVYVGERLAGVFNLSDACRSGVS

AhHMA4-2 KIDGNDIYIGNKRIASRAGCSTVPETEIDTKGGKTVGYVYVGERLAGVFNLSDACRSGVS

AhHMA4-3 KIDGNDIYIGNKRIASRAGCSTVPETEIDTKGGKTVGYVYVGERLAGVFNLSDACRSGVS

AtHMA4 QAMAELKSLGIKTAMLTGDNQAAAMHAQEQLGNVLDVVHGDLLPEDKSRIIQEFKKEGPT

NcHMA4-1-LC QAMKELKDLGIKTAMLTGDNQDSAMQAQEQLGNALDVVHGELLPEDKSKIIQEFKKEGPT

NcHMA4-2-LC QAMKELKDLGIKTAMLTGDNQDSAMQAQEQLGNALDVVHGELLPEDKSKIIQEFKKEGPT

NcHMA4-1-Ga QAMKELKDLGIKTAMLTGDNQDSAMQAQEQLGNALDVVHGELLPEDKSKSYKSLRKKGPT

NcHMA4-2-Ga QAMKELKDLGIKTAMLTGDNQDSAMQAQEQLGNALDVVHGELLPEDKSKIIQEFKKEGPT

NcHMA4-3-Ga QAMKELKDLGIKTAMLTGDNQDSAMQAQEQLGNALDVVHGELLPEDKSKIIQEFKKEGPT

NcHMA4-1-CMA QAMKELKDLGIKTAMLTGDNQDSAMQAQEQLGNALDVVHGELLPEDKSKIIQEFKKEGPT

NcHMA4-2-CMA QAMKELKDLGIKTAMLTGDNQDSAMHAQEQLGNALDVVHGELLPEDKSKIIQEFKKEGPT

NcHMA4-1-SLM QAMKELKDLGIKTAMLTGDNKDSAMHAQEQLGNALDVVHGELLPEDKSKIIQEFKKEGPT

NcHMA4-2-SLM QAMKELKDLGIKTAMLTGDNQDSAMQAQEQLGNALDVVHGELLPEDKSKIIQEFKKEGPT

NcHMA4-3-SLM QAMKELKDLGIKTAMLTGDNKDSAMHAQEQLGNALDVVHGELLPEDKSKIIQEFKKEGPT

NcHMA4-4-SLM QAMKELKDLGIKTAMLTGDNKDSAMHAQEQLGNALDVVHGELLPEDKSKIIQEFKKEGPT

AhHMA4-1 QAMKELKSLGIKTAMLTGDSQAAAMHAQEQLGNALDVVHGELLPEDKSKIIQEFKKEGPT

AhHMA4-2 QAMKELKSLGIKTAMLTGDSQAAAMHAQEQLGNVLDVVHGELLPEDKSKIIQEFKKEGPT

AhHMA4-3 QAMKELKSLGIKTAMLTGDSQAAAMHAQEQLGNALDVVHGELLPEDKSKIIQEFKKEGPT

AtHMA4 AMVGDGVNDAPALATADIGISMGISGSALATQTGNIILMSNDIRRIPQAVKLARRARRKV

NcHMA4-1-LC CMVGDGVNDAPALANADIGISMGISGSALATQTGHIILMSNDIRRIPQAIKLARRAQRKV

NcHMA4-2-LC CMVGDGVNDAPALANADIGISMGISGSALATQTGHIILMSNDIRRIPQAIKLARRAQRKV

NcHMA4-1-Ga CMVGDGVNDAPALANADIGISMGISGSALATQSGHIILMSNDIRRIPKAIKLARRAQRKV

NcHMA4-2-Ga CMVGDGVNDAPALANADIGISMGISGSALATQSGHIILMSNDIRRIPKAIKLARRAQRKV

NcHMA4-3-Ga CMVGDGVNDAPALANADIGISMGISGSALATQTGHIILMSNDIRRIPQAIKLARRAQRKV

NcHMA4-1-CMA CMVGDGVNDAPALANADIGISMGISGSALATQTGHIILMSNDIRRIPQAIKLARRAQRKV

NcHMA4-2-CMA CMVGDGVNDAPALANADIGISMGISGSALATQSGHIILMSNDIRRIPKAIKLARRAQRKV

NcHMA4-1-SLM CMVGDGVNDAPALANADIGISMGISGSALATQSGHIILMSNDIRRIPKAIKLARRAQRKV

NcHMA4-2-SLM CMVGDGVNDAPALANADIGISMGISGSALATQTGHIILMSNDIRRIPQAIKLARRAQRKV

NcHMA4-3-SLM CMVGDGVNDAPALANADIGISMGISGSALATQSGHIILMSNDIRRIPKAIKLARRAQRKV

NcHMA4-4-SLM CMVGDGVNDAPALANADIGISMGISGSALATQSGHIILMSNDIRRIPKAIKLARRAQRKV

AhHMA4-1 AMVGDGVNDAPALATADIGISMGISGSALATQTGHIILMSNDIRRIPQAVKLARRARRKV

AhHMA4-2 AMVGDGVNDAPALATADIGISMGISGSALATQTGHIILMSNDIRRIPQAVKLARRARRKV

AhHMA4-3 AMVGDGVNDAPALATADIGISMGISGSALATQTGHIILMSNDIRRIPQAVKLARRARRKV

AtHMA4 VENVCLSIILKAGILALAFAGHPLIWAAVLVDVGTCLLVIFNSMLLLREKKKIGNKKCYR

NcHMA4-1-LC LQNVFISITLKVGILVLAFAGHPLIWAAVLTDVGTCLIVILNSMLLLREKDKSKIKKCYR

NcHMA4-2-LC LQNVFISITLKVGILVLAFAGHPLIWAAVLTDVGTCLIVILNSMLLLREKDKSKIKKCYR

NcHMA4-1-Ga LENVFISITLKVGILVLAFAGHPLIWAAVLTDVGTCLIVIFNSMLLLREKDKSKNKKCYR

NcHMA4-2-Ga LENVFISITLKVGILVLAFAGHPLIWAAVLTDVGTCLIVIFNSMLLLREKDKSKNKKCYR

NcHMA4-3-Ga LQNVIISITLKVGILVLAFAGHPLIWAAVLTDVGTCLIVILNSMLLLREKDKSKIKKCYR

NcHMA4-1-CMA LQNVFISITLKVGILVLAFAGHPLIWAAVLTDVGTCLIVILNSMLLLREKDKSKIKKCYR

NcHMA4-2-CMA LENVFISITLKVGILVLAFAGHPLIWAAVLTDVGTCLIVIFNSMLLLREKDKSKNKKCYR

NcHMA4-1-SLM LENVFISITLKVGILVLAFAGHPLIWAAVLTDVGTCLIVIFNSMLLLREKDKSKNKKCYR

NcHMA4-2-SLM LQNVIISITLKVGILVLAFAGHPLIWAAVLTDVGTCLIVILNSMLLLREKDKSKIKKCYR

NcHMA4-3-SLM LENVFISITLKVGILVLAFAGHPLIWAAVLTDVGTCLIVIFNSMLLLREKDKSKNKNCYR

NcHMA4-4-SLM LENVFISITLKVGILVLAFAGHPLIWAAVLTDVGTCLIVIFNSMLLLREKDKSKNKNCYR

AhHMA4-1 IENVCLSIILKAGILALAFAGHPLIWAAVLVDVGTCLLVILNSMLLLREKKKIGNKKCYR

AhHMA4-2 IENVSLSIILKAGILALAFAGHPLIWAAVLVDVGTCLLVIFNSMLLLREKKKIGNKKCYR

AhHMA4-3 IENVCLSIILKAGILALAFAGHPLIWAAVLVDVGTCLLVILNSMLLLREKKKIGNKKCYR

AtHMA4 ASTSKLNGRKLEGDDDYVVDLEAGLLTKSGNGQCKSSCCGDKKNQENVVMMKPSSKTSSD

NcHMA4-1-LC --KKVEGGDDQ------GLDLEAGL---LSKSQCNSGCCGDKKSQEKVMLMRPASKTSSD

NcHMA4-2-LC --KKLEGVDDQ------GLDLEAGL---LSKSQCNSGCCGDKKSQEKVMLMRPASKTSSD

NcHMA4-1-Ga ASTSVLNGKKLEGDDEEGLDLEAGL---VSKSQCNSGCCGDKNSQGKVMLMRPASKTSTD

NcHMA4-2-Ga ASTSVLNGKKLEGDDEEGLDLEAGL---VSKSQCNSGCCGDKNSQGKVMLMRPASKTSTD

NcHMA4-3-Ga --KKLEGVDDQ------GLDLEAGL---LSKSQCNSGCCGDKKSQEKVMLMRPASKTSSD

NcHMA4-1-CMA --KKLEGGDDQ------GLDLEAGL---FSKSQCNSGCCGDKKSQEKVMLMRPASKTSTD

NcHMA4-2-CMA ASTSVLNGKKLEGDDEEGLDLEAGL---VSKSQCNSGCCGDKKSQEKVMLMRPASKTSTD

NcHMA4-1-SLM ASTSVLNGKKLEGDDEEGLDLEAGL---VSKSQCNSGCCGDKKSQEKVMLMRPASKTSTD

NcHMA4-2-SLM --KKLEGVDDQ------GLDLEAGL---LSKSQCNSGCCGDKKSQEKVMLMRPASKTSSD

NcHMA4-3-SLM ASTSVLNGKKLEGGDDQGLDLEAGL---FSKSQCNSGCCGDKKSQEKVMLMRPASKTSTD

NcHMA4-4-SLM ASTSVLNGKKLEGDDEEGLDLEAGL---VSKSQCNSGCCGDKKSQEKVMLMRPASKTSTD

AhHMA4-1 ASTSMLNGRKLEGDDDDAVDLEAGLLTKSGNGQCKSSCCGDKKNQEKVVMMKPSSKTSSD

AhHMA4-2 ASTSMLNGRKLEGDDDDAVDLEAGLLTKSGNGQCKSSCCGDKKNQEKVVMMKPSSKTSSD

AhHMA4-3 ASTSMLNGRKLEGDDDDAVDLEAGLLTKSGNGQCKSSCCGDKKNQEKVVMMKPSSKTSSD

AtHMA4 HSHPGCCGDKKEEKVKPLVKDGCCSEKTRKSEGDMVSLSSCKKSSHVKHDLKMKGGSGCC

NcHMA4-1-LC HLHSGCCGEKKQESVKL-VKDSCCGEKSRKPEGDMASLSSCKKSNNDLKM---KGGSSCC

NcHMA4-2-LC HLHSGCCGEKKQESVKL-VKDSCCGEKSRKPEGDMASLSSCKKSNNDLKM---KGGSSCC

NcHMA4-1-Ga HLHSGCCGEKKQESVKL-VKDSCCGEKSRKPEGDMASLSSCKKSNNDLKM---KGGSSCC

NcHMA4-2-Ga HLHSGCCGEKKQESVKL-VKDSCCGEKSRKPEGDMASLSSCKKSNNDLKM---KGGSSCC

NcHMA4-3-Ga HLHSGCCGEKKQESVKL-VKDSCCGEKSRKPEGDMASLSSCKKSNNDIKM---KGGSSCC

NcHMA4-1-CMA HLHSGCCGEKKQESVKL-VKDSCCGEKSRKPEGDMASLSSCKKSNNDLKM---KGGSSCC

NcHMA4-2-CMA HLHSGCCGEKKQESVKL-VKDSCCGEKSRKPEGDMASLSSCKKSNNDLKM---KGGSSCC

NcHMA4-1-SLM HLHSGCCGEKKQESVKL-VKDSCCGEKSRKQEGDMASLSSCKKSDNDLKM---KGGSSCC

NcHMA4-2-SLM HLHSGCCGEKKQESVKL-VKDSCCGEKSRKPVGDMASLSSCKKSNNDLKM---KGGSSCC

NcHMA4-3-SLM HLHSGCCGEKNQESVKL-VKDSCCGEKSKKPEGDMASLSSCKNSNNDLKM---KGGSSCC

NcHMA4-4-SLM HLHSGCCGEKNQESVKL-VKDSCCGEKSKKPEGDMASLSSCKNSNNDLKM---KGGSSCC

AhHMA4-1 HSHPGCCGDKKQGNVKPLVRDGGCSEETRKAVGDMVSLSSCKKSSHVKHDLKMKGGSGCC

AhHMA4-2 HSHPGCCGDKKQGNVKPLVRDGGCSEETRKAVGDMVSLSSCKKSSHVKHDLKMKGGSGCC

AhHMA4-3 HSHPGCCGDKKQGNVKPLVRDGGCSEETRKAVGDMVSLSSCKKSSHVKHDLKMKGGSGCC

AtHMA4 ASKNEK-GKEVVAKSCCEKPKQQVESVGDCKSGHCEKKKQAEDIVVPVQIIGHALTHVEI

NcHMA4-1-LC ASKNEKLKEAVVAKSCCED------KEKTEGN------VEMQILNLEKGSQKKV------

NcHMA4-2-LC ASKNEKLKEVVVAKSCCED------KEKAEGN------VEMQILNLEKGSQKKV------

NcHMA4-1-Ga ASKNEKLKEVVVAKSCCED------KEKAEGN------VEMQILDLEKGSQKKV------

NcHMA4-2-Ga ASKNEKLKEVVVAKSCCED------KEKAEGN------VEMQILDLEKGSQKKV------

NcHMA4-3-Ga ASKNEKLKEVVVAKSCCEE------KEKAEGN------VEMQILNLEKGSQKKV------

NcHMA4-1-CMA ASKNEKLKEVVVAKSCCGE------KEKAEGN------VEMQILNLEKGSQKKV------

NcHMA4-2-CMA ASKNEKLKEVVVAKSCCED------KEKAEGN------VEMQILDLEKGSQKTV------

NcHMA4-1-SLM ASKNEKLKEVAVAKTCCED------KEKAEGN------VEMQILDLEKGSQKKV------

NcHMA4-2-SLM ASKNEKLKEVVVAKSCCEE------KEKAEGN------VEMQILNLEKGSQKKV------

NcHMA4-3-SLM ASKNEKLKEVVVAKSCCGE------KEKAEGN------VEMQILNLEKGSQKKV------

NcHMA4-4-SLM ASKNEKLKEVVVAKSCCGE------KEKAEGN------VEMQILNLEKGSQKKV------

AhHMA4-1 ANKSEK-VEEVVAKSCCEKPKQQMESAGDCKSSHCEEKKHAEEIVLPVQMIGQALTGLEI

AhHMA4-2 ANKSEK-VEEVVAKSCCEKPKQQMESAGDCKSSHCEEKKHAEEIVLPVQMIGQALTGLEI

AhHMA4-3 ANKSEK-VEEVVAKSCCEKPKQQMESAGDCKSSHCEEKKHAEEIVLPVQMIGQALTGLEI

AtHMA4 ELQTKETCKTSCCDSKEKVKETGLLLSSENTPYLEK--------GVLIKDEGNCKSGSEN

NcHMA4-1-LC ----GETCKSSCCGDKEKAKETRLLLASEDPSYLEKEERQTTEANIVTVKQSCHEKASLD

NcHMA4-2-LC ----GETCKSSCCGDKEKAKETRLLLASEDPSYLEKEERQTTEANIVTVKQSCHEKASLD

NcHMA4-1-Ga ----GETCKSSCCGDKEKAKETRLLLASEDPSYLEKEERQTTEANIVTVKQSCHEKASLD

NcHMA4-2-Ga ----GETCKSSCCGDKEKAKETRLLLASEDPSYLEKEERQTTEANIVTVKQSCHEKASLD

NcHMA4-3-Ga ----GETCKSSCCGDKEKAKETRLLLASEDPSYLEKEERQTTEANIVTVKQSCHEKASLD

NcHMA4-1-CMA ----GETCKSSCCGDKEKAKETRLLLASEDPSYLEKEERQTTEANIVTVKQSCHEKASLD

NcHMA4-2-CMA ----GETCKSSCCGDKEKAKETRLLLASEDPSYLEKE-----------------------

NcHMA4-1-SLM ----GETCKSSCCGDKEKAKETRLLLASEDPSYLEKEERQTTEANIVTVKQSCHEKASLD

NcHMA4-2-SLM ----GETCKSSCCGDKEKAKETRLVLASEDPSYLEKEERQTTEANIVTVKQSCHEKASLD

NcHMA4-3-SLM ----GETCKSSCCGDKEKAKETRLLLASEDPSYLEKE-----------------------

NcHMA4-4-SLM ----GETCKSSCCGDKEKAKETRLLLASEDPSYLEKE-----------------------

AhHMA4-1 ELQTKETCKTRCCDNKEKAKKKGLLLSSEDTSYLEK--------GVLIKDEGNCKSACQK

AhHMA4-2 ELQTKETCKTRCCDNKEKAKKKGLLLSSEDTSYLEK--------GVLIKDEGNCKSACQK

AhHMA4-3 ELQTKETCKTRCCDNKEKAKKKGLLLSSEDTSYLEK--------GVLIKDEGNCKSACQK

AtHMA4 MGTVKQS----CHEK---------GCSDEKQTGEITLASEEETD---DQDCSSGCCVNEG

NcHMA4-1-LC IETGVTCDLKLVCCGNIEVGEQSDLEKGMKLKGEGQCKSDCCGDEIPLASEEDSVDCSSG

NcHMA4-2-LC IETGVTCDLKLVCCGNIEVGEQSDLEKGMKLKGEGQCKSDCCGDEIPLASEEDSVDCSSG

NcHMA4-1-Ga IETGVTCDLKLVCCGNIEVGEQSDLEKGMKLKGEGQCKSDCCGDEIPLASEEDSVDCSSG

NcHMA4-2-Ga IETGVTCDLKLVCCGNIEVGEQSDLEKGMKLKGEGQCKSDCCGDEIPLASEEDSVDCSSG

NcHMA4-3-Ga IETGVTCDLKLVCCGNIEVGEQSDLEKGMKLKGEGQCKSDCCGDEIPLASEEDSVDCSSG

NcHMA4-1-CMA IETGVTCDLKLVCCGNIEVGEQSDLEKGMKLKGEGQCKSDCCGDEIPLASEEDSVDCSSG

NcHMA4-2-CMA ------------------------------------------------------------

NcHMA4-1-SLM IETGVTCDLKLVCCGDIEVGEQSDLEKGMKLKGEGQCKSDCCGDEIPLTSEEDSVDCSSG

NcHMA4-2-SLM IENGVTCDLKLVCCGNIEVGEQSDLEKGMKLKGEGQCKSDCCGDEIPLASEEDSVDCSSG

NcHMA4-3-SLM ------------------------------------------------------------

NcHMA4-4-SLM ------------------------------------------------------------

AhHMA4-1 TGTVKES----CHEKAPLDIETKLVSCGNTEGEVGEQTDLEIKI---EGDCKSGCCSDEK

AhHMA4-2 TGTVKQS----CHEKAPLDIETKLVSCGNTEGEVGEQTDLEIKI---EGDCKSGCCSDEK

AhHMA4-3 TGTVKQS----CHEKAPLDIETKLVSCGNTEGEVGEQTDLEIKI---EGDCKSGCCSDEK

AtHMA4 TVKQSFDEKKHSVLVEKEGLDMETGFCCDAKLVCCGNTEGEVKEQCRLEIK----KEEHC

NcHMA4-1-LC CCGNKEELTQICHEK-----TCLDIVSCDSKLVCCGETEVEVREQCDLKKGLQIKNEGQC

NcHMA4-2-LC CCGNKEELTQICHEK-----TCLDIVSCDSKLVCCGETEVEVREQCDLKKGLQIKNEGQC

NcHMA4-1-Ga CCGNREELTQICHEK-----TCLDIVSCDSKLVCCGETEVEVREQCDLKKGLQIKNEGQC

NcHMA4-2-Ga CCGNKEELTQICHEK-----ACLDIVSCDSKLVCCGETEVEVREQCDLKKGLQIKNEGQC

NcHMA4-3-Ga CCGNKEELTQICHEK-----ACLDIVSCDSKLVCCGETEVEVREQCDLKKGLQIKNEGQC

NcHMA4-1-CMA CCGNKEELTQICHEK-----ACLDIVSCDSKLVCCGETEVEVREQCDLKKGLQIKNEGQC

NcHMA4-2-CMA ------------------------------------------------------------

NcHMA4-1-SLM CCGNKEELTQICHEK-----ACLDIVSCDSKLVCCGETEVEVREQCDLKKGLQIKNEGQC

NcHMA4-2-SLM CCGNKEELTQICHEK-----ACLDIVSCDSKLVCCGETEVEVREQCDLKKGLQIKNEGQC

NcHMA4-3-SLM ------------------------------------------------------------

NcHMA4-4-SLM ------------------------------------------------------------

AhHMA4-1 QTGEI---TLASEEE-----TDSTDCSSG---CCMDKEEV--TQICGLETE----GGGDC

AhHMA4-2 QTGEI---TLASEEE-----TDSTDCSSG---CCMDKEEV--TQICGLETE----GGGDC

AhHMA4-3 QTGEI---TLASEEE-----ADSTDCSSG---CCMDKEEV--TQICGLETE----GGGDC

AtHMA4 KSGCCGEE---------------IQTGEITLVSEEETESTNC-----ST-GCCVD-KEEV

NcHMA4-1-LC KSVCCGDEKKTEEITEE----TDNLKSESGDDCKSLCCGTGLKQEGSSSLVNVVVESGES

NcHMA4-2-LC KSVRCGDEKKTEEITEE----TDNLKSESGDDCKSLCCGTGLKQEGSSSLVNVVVESGES

NcHMA4-1-Ga KSVCCGDEKKTEEITEE----TDNLKSESGDDCKSLCCGTGLKHEGSSSLVNVVVESGES

NcHMA4-2-Ga ESVCCGDEKKTEEITLVSDEETDNLKSESGGDSKALCCGTGLKQEGSSSLVNVVVESGES

NcHMA4-3-Ga ESVCCGDEKKTEEITLVSDEETDNLKSESGGDSKALCCGTGLKQEGSSSLVNVVVESGES

NcHMA4-1-CMA ESVCCGDEKKTEEITLVSDEETDNLKSESGGDSKALCCGTGLKQEGSSSLVNVVVESGES

NcHMA4-2-CMA -----------------------NLKSESGDDCKSLCCGTGLKQEGSSSLVNVVVESGES

NcHMA4-1-SLM KSVCCGDEKKTEEITEE----TDNLKSESGDDCKSLCCGTGLKQEGSSSLVNVVVESGES

NcHMA4-2-SLM ESVCCGDEKKTEEITLVSDEETDNLKSESGGDSKALCCGTGLKQEGSSSLVNVVVESGES

NcHMA4-3-SLM -----------------------NLKSESGDDCKSLCCGTGLKQEGSSSLVNVVVESGES

NcHMA4-4-SLM -----------------------NLKSESGDDCKSLCCGTGLKQEGSSSLVNVVVESGES

AhHMA4-1 KSHCCG-------------------TGLTQEGSS--KLGNVESAQSGGC-GTVKV-SSQS

AhHMA4-2 KSHCCG-------------------TGLTQEGSS--KLGNVESAQFGGC-GTVKV-SSQS

AhHMA4-3 KSHCCG-------------------TGLTQEGSS--KLGNVETAQSGGC-GTVKV-SSQS

AtHMA4 TQTCHEKPASLVVSGLEVKKDEHCESSHR-----AVKVETCCKVKIPEACASKCRDRAK-

NcHMA4-1-LC GSSCCSKEGEIVKVS--SQSCCASPSDVVLSDFQAKKLEICCEVKKTPEEVCGSKCKETE

NcHMA4-2-LC GSSCCSKEGEIVKVS--SQSCCASPSDVVLSDLEVKKLEICCKAKKTPEEVRGSKCKETE

NcHMA4-1-Ga GSSCCSKEGEIVKVS--SQSCCTSPSDVVLSDLQAKKLEICCKVKKTPEEVCGSKCKATE

NcHMA4-2-Ga GSSCCSKEGEIVKVS--SQSRCTSPSDVVLSDLQAKKLEICCKVKKTLEEVRGSKCKETE

NcHMA4-3-Ga GSSCCSKEGEIVKVS--SQSRCTSPSDVVLSDLQAKKLEICCKVKKTLEEVRGSKCKETE

NcHMA4-1-CMA GSSCCSKEGEIVKVS--SQSRCTSPSDVVLSDLQAKKLEICCKVKKTLEEVRGSKCKETE

NcHMA4-2-CMA GSSCCSKEGEIVKVS--SQSCCTSPSDVVLSDFQAKKLEICCEVKKTPEEVCGSKCKETE

NcHMA4-1-SLM GSSCCSKEGEIVKVS--SQSCCTSPSDVVLSDFQAKKLEICCKVKKTPEEVCGSKCKATE

NcHMA4-2-SLM GSSCCSKEGEIVKVS--SQSRCTSPSDVVLSDLQAKKLEICCKVKKTLEEVRGSKCKETE

NcHMA4-3-SLM GSSCCSKEGEIVKVS--SQSCCTSPSDVVLSDFQAKKLEICCEVKKTPEEVCGSKCKETE

NcHMA4-4-SLM GSSCCSKEGEIVKVS--SQSCCTSPSDVVLSDFQAKKLEICCEVKKTPEEVCGSKCKETE

AhHMA4-1 CCTSSTDLVL---SDLQVKKDEHCKSSHG-----AVKVETCCKVKIPEACASECKEKEK-

AhHMA4-2 CCTSSTDLVL---SDLQVKKDEHCESSHG-----AVKVETCCKVKIPEACAPECKEKEK-

AhHMA4-3 CCTSSTDLVL---SDLQVTKDEHCESSHG-----AVKVETCCKVKIPEACAPECKEKEK-

AtHMA4 --RHSGKSCCRSYAKELCSHRHHHHHHHHHHHVSA

NcHMA4-1-LC KRHHVGKSCCRSYAKEHCSHRHHHHHHHHHHVGAA

NcHMA4-2-LC KRHHVGKSCCRSYAKEYCSHRHHHHHHHHHVGAA-

NcHMA4-1-Ga KPHHVGKSCCRSYAKEYCSHRHHDNHVGAA-----

NcHMA4-2-Ga KPHHVGKSCCRSYAKEYCSHRHHDNHVGAA-----

NcHMA4-3-Ga KPHHVGKSCCRSYAKEYCSHRHHHHHHHHHVGAA-

NcHMA4-1-CMA KPHHVGKSCCRSYAKEYCSHRHHHHHHHHHVGAA-

NcHMA4-2-CMA KPHHVGKSCCRSYAKEYCSHRHHDNHHHHHVGAA-

NcHMA4-1-SLM KPHHVGKSCCRSYAKEYCSHRHHDNHHHHHVGAA-

NcHMA4-2-SLM KPHHVGKSCCRSYAKEYCSHRHHHHHHHHHVGAA-

NcHMA4-3-SLM KPHHVGKSCCRSYAKEYCSHRHHDNHHHHHVGAA-

NcHMA4-4-SLM KPHHVGKSCCRSYAKEYCSHRHHDNHHHHHVGAA-

AhHMA4-1 --RHSGKSCCRSYAKEFCSHRHHHHHHHHVSA---

AhHMA4-2 --RHSGKSCCRSYAKEFCSHRHHHHHHHHVSA---

AhHMA4-3 --RHSGKSCCRSYAKEFCSHRHHHHHHHHHHVSA-

**Supplementary Alignment S2.** Amino acid sequence alignment of *HMA4*s from *A. thaliana*, *N. caerulescens* accessions (La Calamine, Ganges, Col du Mas de l’Aire and Saint Laurent Le Minier) and *A. halleri*

pAtHMA4 ------------------------------------------------------------

pNcHMA4-1-LC -----CC-----------TTCTCTCTCTTC---------TTTTCTTTCTCCACTTTATTC

pNcHMA4-2-LC ------------------------------------------------------------

pNcHMA4-3-LC ------------------------------------------------------------

pNcHMA4-1-Ga ------------------------------------------------------------

pNcHMA4-2-Ga ------------------------------------------------------------

pNcHMA4-3-Ga ------------------------------------------------------------

pNcHMA4-1-CMA ------------------------------------------------------------

pNcHMA4-2-CMA ------------------------------------------------------------

pNcHMA4-1-SLM ------------------------------------------------------------

pNcHMA4-2-SLM TTTTACCAGTTACACAATTTATCTCTCTCCACGATAAATCCTTCTCTCTCTTCTTT-TCT

pNcHMA4-3-SLM ------------------------------------------------------------

pNcHMA4-4-SLM ------------------------------------------------------------

pAhHMA4-1 ------------------------------------------------------------

pAhHMA4-2 ------------------------------------------------------------

pAhHMA4-3 ------------------------------------------------------------

pAtHMA4 ------------------------------------------------------------

pNcHMA4-1-LC ATCTCCACTTTATTCATCTCCACTTTCCTTCTCTCTTTGCTTCCTACAAATTCTCTGTCT

pNcHMA4-2-LC ------------------------------------------------------------

pNcHMA4-3-LC ------------------------------------------------------------

pNcHMA4-1-Ga ------------------------------------------------------------

pNcHMA4-2-Ga ------------------------------------------------------------

pNcHMA4-3-Ga ------------------------------------------------------------

pNcHMA4-1-CMA ------------------------------------------------------------

pNcHMA4-2-CMA ------------------------------------------------------------

pNcHMA4-1-SLM ------------------------------------------------------------

pNcHMA4-2-SLM TTCTCCACTTTATTCATCTCCACTTTCCTTATCTCTTTGCTTCCTACAAATTCTCTGTCT pNcHMA4-3-SLM ------------------------------------------------------------

pNcHMA4-4-SLM ------------------------------------------------------------

pAhHMA4-1 ------------------------------------------------------------

pAhHMA4-2 ------------------------------------------------------------

pAhHMA4-3 -----------------------------------------------------TCTTTGT

pAtHMA4 ------------------------------------------------------------

pNcHMA4-1-LC TTCTCATTTTCCACCAGGTTCTTCTTCCACCAATAAAGATTGGGAGGGACAATGGTTGCG

pNcHMA4-2-LC ------------------------------------------------------------

pNcHMA4-3-LC ------------------------------------------------------------

pNcHMA4-1-Ga ------------------------------------------------------------

pNcHMA4-2-Ga ------------------------------------------------------------

pNcHMA4-3-Ga ------------------------------------------------------------

pNcHMA4-1-CMA ------------------------------------------------------------

pNcHMA4-2-CMA ------------------------------------------------------------

pNcHMA4-1-SLM ------------------------------------------------------------

pNcHMA4-2-SLM TTCTCATTTTCCACTAGGTTCTTCTTCCACCAATAAAGATTGGGA----CAAGGGTTGCG

pNcHMA4-3-SLM ------------------------------------------------------------

pNcHMA4-4-SLM ------------------------------------------------------------

pAhHMA4-1 ------------------------------------------------------------

pAhHMA4-2 ------------------------------------------------------------

pAhHMA4-3 TCGGCTTCCCCCATAGGGAGTTTGTTTGCCTGTAACTTATTATAATGGAGAGTTTGTTGA

pAtHMA4 ------------------------------------------------------------

pNcHMA4-1-LC TAAAGACATTAT---GCCTTGAATAAGTGTTTGTGTATGC----CCTTTTT-CAAAAAAA

pNcHMA4-2-LC ------------------------------------------------------------

pNcHMA4-3-LC ------------------------------------------------------------

pNcHMA4-1-Ga ------------------------------------------------------------

pNcHMA4-2-Ga ------------------------------------------------------------

pNcHMA4-3-Ga ------------------------------------------------------------

pNcHMA4-1-CMA ------------------------------------------------------------

pNcHMA4-2-CMA ------------------------------------------------------------

pNcHMA4-1-SLM ------------------------------------------------------------

pNcHMA4-2-SLM TAAAGACATTATTATGCCTTGAATAAGTGTTTGTGTATGCATGCCCTTTTTACAAAAAAA

pNcHMA4-3-SLM ------------------------------------------------------------

pNcHMA4-4-SLM ------------------------------------------------------------

pAhHMA4-1 ------------------------------------------------------------

pAhHMA4-2 ------------------------------------------------------------

pAhHMA4-3 CAAAAAAAAAATTGTCGATTCAAATTTTAAGAAATTTCGTATAAATTAAGTCAGTAAATC

pAtHMA4 ------------------------------------------------------------

pNcHMA4-1-LC GAAAAAAGAATAAGTGTTTGTGAACAAAATATCTTGGACAGAATTTTAGAGTAT------

pNcHMA4-2-LC ---------------GTATGTGAACAAAATATCTTGGACAGAATTTTAGAGTAT------

pNcHMA4-3-LC ------------------------------------------------------------

pNcHMA4-1-Ga ---------------AGGAACTAGGTTGAAAATTCGGAAGGAATATAGGAATTCG-----

pNcHMA4-2-Ga ------------------------------------------------------------

pNcHMA4-3-Ga ------------------------------------------------------------

pNcHMA4-1-CMA ------------------------------------------------------------

pNcHMA4-2-CMA ------------------------------------------------------------

pNcHMA4-1-SLM ------------------------------------------------------------

pNcHMA4-2-SLM AAAAAAAGAATAAGTGTTTGTGAACAAAATATCTTGGATAGAATTTTAGAATATGATTGA

pNcHMA4-3-SLM ------------------------------------------------------------

pNcHMA4-4-SLM ------------------------------------------------------------

pAhHMA4-1 ------------------------------------------------------------

pAhHMA4-2 ------------------------------------------------------------

pAhHMA4-3 ATCAAAAAAATTTAAAGGAGTTAGACTAGTCTATAATCCAATATTGAGGATAATC-----

pAtHMA4 --------------------------------------------------------TCTA

pNcHMA4-1-LC ---------------------------ATCACGTAGATCGTGGACAATATGCCATGGACA

pNcHMA4-2-LC ---------------------------ATCACGTAGATCGTGGACAATATGCCATGGACA

pNcHMA4-3-LC ------------------------------------------------------------

pNcHMA4-1-Ga ----------------------------------AAACAAAGATTAAAATATCCTACGAA

pNcHMA4-2-Ga --------------------------------------------------------CGAA

pNcHMA4-3-Ga ----------------------------------------TGGACAATAGGTGTGTGAAC

pNcHMA4-1-CMA ---------------------------------------------AATAGGTGTGTGAAC

pNcHMA4-2-CMA ------------------------------------------------------------

pNcHMA4-1-SLM ------------------------------------------------------------

pNcHMA4-2-SLM CAAAAAAAAAAAAGAATTTTAGAATATATCACGTAGATCGTGGACAATATGCCATGGACA

pNcHMA4-3-SLM ------------------------------------------------------------

pNcHMA4-4-SLM -----------------------------------------------TATATTTTACTAT

pAhHMA4-1 --------------------------------------------------------GAAG

pAhHMA4-2 ------------------------------------------------------------

pAhHMA4-3 ---------------------------------CTACCAAAAAAAAATCTAATATTGAAG

pAtHMA4 ATAAATGATGCAATCAAAAT--------------CTTCCATG--CACAGAGAAGAGTTGG

pNcHMA4-1-LC AT--GGGTGTGTGAACATATATAT----TCCGACATATAAC----AATAAAATATGTACA

pNcHMA4-2-LC AT--GGGTGTGTGAACATATATAT----TCCGACATATAAC----AATAAAATATGTACA

pNcHMA4-3-LC ------------------------------------------------------------

pNcHMA4-1-Ga ATTAACATAGTAAAAAAAAAAA--------CTAAACCAAAT----AAAAAAAGTTTAACG

pNcHMA4-2-Ga ATTAACATGGTAAAAAAAA-----------CTAAACCAAAT----AAAAAAAGTTTAACG

pNcHMA4-3-Ga ATATATATATATATATATATAT------TCAGACATATAAC----AATAAAATATGTACA

pNcHMA4-1-CMA ATATATATATATATATATATATATATATTCAGACATATAAC----AATAAAATATGTACA

pNcHMA4-2-CMA ------------------------------------------------------------

pNcHMA4-1-SLM ----TCAATTTTTACTTTTTAT-----------CTCTCAAC----AAAGAGATATCTTC-

pNcHMA4-2-SLM ATATGGGTGTGTGAACATATATATA--TTCAGACATATAAC----AATAAAATATGTACA

pNcHMA4-3-SLM --CTACCTTTCGTAGTTTTCGT----------ATCCTACGT----ACTACAGTTCATTC-

pNcHMA4-4-SLM TTTTTCAATTTTTACTTTTTAT-----------CTCTCAAC----AAAGAGATATCTTC-

pAhHMA4-1 GTAGACGA--CCATTGGTGT--------------GTTGCGT---TAAAAAATTTTGTAGT

pAhHMA4-2 ----------------------A--------GGAACTG-------------------GCG

pAhHMA4-3 ATAAATGTTTATCATAAGATATA--------GGAACTGAATCTTGAACATAGGAACTGCG

pAtHMA4 TAA------------TTGTT-GCAAT--TGTAAA--AACAAAAACCCAT-----AAAATC

pNcHMA4-1-LC CAAGCTTCGA--TTTTCACCTCTGATA-CACAATTCATCCACATCTTT------ATTATG

pNcHMA4-2-LC CAAGCTTCGA--TTTTCACCTCTGATA-CACAATTCATCCACATCTTT------ATTATG

pNcHMA4-3-LC --AGCTTCGA--TTTTCACCTCTGATA-CACAATTCATCCACATCTTT------ATTATG

pNcHMA4-1-Ga TAAAAAGAAAAGTTTTAATTCAAA-GAATCCAAACTAACCACAACTTTTGAAACATAATC

pNcHMA4-2-Ga TAAAAAGAAAAGTTTTAATTCAAAAGAATCCAGACTAACCACAACTTTTGAAGCATAATC

pNcHMA4-3-Ga CAAGCTTCGA--TTTTCACCTCTGATACAACAATTCATCCACATCTTT------ATTATG

pNcHMA4-1-CMA CAAGCTTCGA--TTTTCACCTCTGATACAACAATTCATCCACATCTTT------ATTATG

pNcHMA4-2-CMA ------------------------------------------------------------

pNcHMA4-1-SLM -------------TCTCCCTCACGATA-AATCCTTCTCTCTCTTCTTT------TCTTTC

pNcHMA4-2-SLM CAAGCTTCGA--TTTTCACCTCTGATA-CACAATTCATCCACATCTTT------ATTATG

pNcHMA4-3-SLM -------------TACCTCCCGCAGAATTAGTGTTCTACTAAAAGCAA------GTTTTC

pNcHMA4-4-SLM -------------TCTCCCTCACGATA-AATCCTTCTCTCTCTTCTTT------TCTTTC

pAhHMA4-1 TAA------------TTATTTAGGAT--CATAAACTAAGAAAATCGTAC-----GAAAAA

pAhHMA4-2 TGAAAA--------TTAGTTGTTGAACACATGAACTGGCCTGGAAATTAGGAAGGGTATA

pAhHMA4-3 TGAAAA--------TCAGTTGTTGAACACAGGAACTGGCCTGGTAATTAGGAAGGATATA

pAtHMA4 TC---AAGAACCTCATGTCACACAGATAATG----CCACATTGC-TTACTTAAAAA----

pNcHMA4-1-LC TCCAAACAAAAGTAATCCACGGCAATTAATC-TACATAATTTGGCTCTCTCTTTGA----

pNcHMA4-2-LC TCCAAACAAAAGTAATCCACGGCAATTAATC-TACATAATTTGGCTCTCTCTTTGA----

pNcHMA4-3-LC TCCAAACAAAAGTAATCCACGGCAATTAATC-TACATAATTTGGCTCTCTCTTTGA----

pNcHMA4-1-Ga CATAAATGTTAG--GTTTAGAACGGCT--TT-TACAGATTTAGTAACTATTCTCAAA---

pNcHMA4-2-Ga CATAAATGTTAG--GTTTAGAACGACT--TT-TAAAGATTTAGTAACTATTTTCAAA---

pNcHMA4-3-Ga TCCAAACAAAAGTAATCCACGGCAATAAATC-TACATAATTTG--GCTCTCTTTGA----

pNcHMA4-1-CMA TCCAAACAAAAGTAATCCACGGCAATAAATC-TACATAATTTG--GCTCTCTTTGA----

pNcHMA4-2-CMA ------------------------------------------------------------

pNcHMA4-1-SLM TCCACTTTA----------------TTCATC-TCCACTTTCCT--TCTCTCTTTGC----

pNcHMA4-2-SLM TCCAAACAAAAGTAATCCACGGCAATTAATC-TACATAATTTGGCTTTCTCTTTGA----

pNcHMA4-3-SLM TAC------------------------TATC-CGTAGTATTCG--TGTTTGTACAAGATG

pNcHMA4-4-SLM TCCACTTTA----------------TTCATC-TCCACTTTCCT--TCTCTCTTTGC----

pAhHMA4-1 TA---ATAAATACAATA--AAGAAAACAATG----AGATGAGGAATCACACATGGA----

pAhHMA4-2 TA-ATTTGAAAACAAATAACTAAAAATAGTCATGAAAAAATACTTTAGGTTTAAGAAAGG

pAhHMA4-3 TA-ATTTGAAAACAAATAACTAAAAATAGTCATGAAAAAATACTTTAGGTTTAAGAAAGG

pAtHMA4 ---ATTTGCTTTCTTGTTTTTCTTTTTTTTT---T-------------------------

pNcHMA4-1-LC TTCTTCCACTCTTACTTTCATCTTTTTATTTCCGT-----------AATCA--CAAGCAA

pNcHMA4-2-LC TTCTTCCACTCTTACTTTCATCTTTTTATTTCCGT-----------AATCA--CAAGCAA

pNcHMA4-3-LC TTCTTCCACTCTTACTTTCATCTTTTTATTTCCGT-----------AATCA--CAAGCAA

pNcHMA4-1-Ga -TCATTTAGTACGTCTTTCTT---------------------------------------

pNcHMA4-2-Ga -TCATTTAGTACGTCTTTCTTTTTTTTTTTTGGCTCAAACATAACAGATTTCATTAGAAA

pNcHMA4-3-Ga TTCTTCCACTCTTAGTTTCATCTTTTTATTTCCGT-----------AATCA--CAAGCAA

pNcHMA4-1-CMA TTCTTCCACTCTTACTTTCATCTTTTTATTTCCGT-----------AATCA--CAAGCAA

pNcHMA4-2-CMA ------------------------------------------------------------

pNcHMA4-1-SLM TTCCTACAAATTCTGTCTCTCATTTTCCACTAGGT-----------TCTTCTTCCACCAA

pNcHMA4-2-SLM TTCTTCCACTCTTACTTTCATCTTTTTATTTCCGT-----------AATCA--CAAGCAA

pNcHMA4-3-SLM TTCTACTAAAATAAGTATCATTATACCTTTTGTAT-----------AAATCATCTTCTAC

pNcHMA4-4-SLM TTCCTACAAATTCTGTCTCTCATTTTCCACTAGGT-----------TCTTCTTCCACCAA

pAhHMA4-1 ---TTCCATTTTCGTGACATTCCACTTTTGGGGGT-------------------------

pAhHMA4-2 TTTTTCTAATATTTAGTAATAATTTTTAATCACTT--------------------GGTAC

pAhHMA4-3 TTTTTCTAAAATTTAGTAATAATTTTTAATCACTT--------------------GGTAC

pAtHMA4 -----------------TTTTGCCAAGA-------A-----------ACCAATGTTTTTC

pNcHMA4-1-LC CAAT-----------GTTTCATTTTCACTTCTCGT-----TATC---ATCATATATT--A

pNcHMA4-2-LC CAAT-----------GTTTCATTTTCACTTCTCGT-----TATC---ATCATATATT--A

pNcHMA4-3-LC CAAT-----------GTTTCATTTTCACTTCTCGT-----TATC---ATCATATATT--A

pNcHMA4-1-Ga ------------------------AAACTTCTAAGAACCAAATTTGTGGTATATATTTGA

pNcHMA4-2-Ga TGATTTCATTAGTACGTCTTTCTTAAACTTCTAAGAACCAAATTTGTGGTATATATTTGA

pNcHMA4-3-Ga CAAT-----------GTTTCATTTTCACTTCTCGT-----TATC---ATCATATATT--A

pNcHMA4-1-CMA CAAT-----------GTTTCATTTTCACTTCTCGT-----TATC---ATCATATATT--A

pNcHMA4-2-CMA ---------------------------CTTCTCGT-----TATC---ATCATATATT--A

pNcHMA4-1-SLM TAAA-----------GATTGGGACAAGGGTTGCGTAAAGACATT---ATTATGCCTTGAA

pNcHMA4-2-SLM CAAT-----------GTTTCATTTTCACTTCTCGT-----TATC---ATCATATATT--A

pNcHMA4-3-SLM CAAA-----------A--TCGAGAAAATATTTCA-------------ACTGAATATTTTT

pNcHMA4-4-SLM TAAA-----------GATTGGGACAAGGGTTGCGTAAAGACATT---ATTATGCCTTGAA

pAhHMA4-1 -----------------TTCCACTAAAATTTTGGTA-----------ACTTTTGATTTCG

pAhHMA4-2 C---------------CCTTCTTAAAAATTTTAATA-----------ACCATTTATTTAC

pAhHMA4-3 C---------------CCTTCTTAAAATTTTTAATA-----------ATCATTTATTTAC

pAtHMA4 T---TTTCTCTTTT-------TTTTTTTTTGAGCAA------AACCAAA-AAACC-----

pNcHMA4-1-LC C--AATTTT---CTA-------CCCATGTTAACCCTATC--------AAAACACC-----

pNcHMA4-2-LC C--AATTTT---CTA-------CCCATGTTAACCCTATC--------AAAACACC-----

pNcHMA4-3-LC C--AATTTT---CTA-------CCCATGTTAACCCTATC--------AAAACACC-----

pNcHMA4-1-Ga T--ATATTTGAGTTA-------TCTTTTTCGTTCGACTTT-------GATGTAAT-----

pNcHMA4-2-Ga T--ATATTTGAGTTA-------TCTTTTTCGTTCGACTTT-------GATGTAAT-----

pNcHMA4-3-Ga C--AATTTT---TTA-------CCCATGTTAACCCTATC--------AAAACACC-----

pNcHMA4-1-CMA C--AATTTT---TTA-------CCCATGTTAACCCTATC--------AAAACACC-----

pNcHMA4-2-CMA C--AATTTT---TTA-------CCCATGTTAACCCTATC--------AAAACACC-----

pNcHMA4-1-SLM T--AAGTGT---TTG-------TGTATGCATGCCCTTTTTACAAAAAAAAAAAAA-----

pNcHMA4-2-SLM C--AATTTT---CTA-------CCCATGTTAACCCTATT--------AAAACACC-----

pNcHMA4-3-SLM C--AAATCT---TTG-------TTTCTTTTTTTCCTCTAACTATCCGAAAATAAACCAAA

pNcHMA4-4-SLM T--AAGTGT---TTG-------TGTATGCATGCCCTTTTT--CAAAAAAAAAAAA-----

pAhHMA4-1 T--ATCTCTCCCTCACTAAAATCCCCCTTTGATCAATTTTTCGTCCAACTAAATT-----

pAhHMA4-2 CTAAAATTTG--TGG-------TTTTTTTTGATAATTTTC--------------------

pAhHMA4-3 CTAAAATTTG--TGGG------TTTTTTTTGATAATTTTC--------------------

pAtHMA4 -------AATGTATATTTGTACTTT----------ACCAATAATAAAAT-----CTCAAA

pNcHMA4-1-LC -------AATT-------GGATAC------------ATGAAAACAA----TTCAATCCCA

pNcHMA4-2-LC -------AATT-------GGATAC------------ATGAAAACAA----TTCAATCCCA

pNcHMA4-3-LC -------AATT-------GGATAC------------ATGAAAACAA----TTCAATCCCA

pNcHMA4-1-Ga -----TGAATTTTTTGGGGGATTTC-----------GGGAAAGCAAAATATACATTTCGG

pNcHMA4-2-Ga -----TGAATTTTTTGGGGGATTTC-----------GGGAAAGCAAAATATACATTTCGG

pNcHMA4-3-Ga -------AATT-------AGATAC------------ATGAAAACAA----TTCAATCCCA

pNcHMA4-1-CMA -------AATT-------AGATAC------------ATGAAAACAA----TTCAATCCCA

pNcHMA4-2-CMA -------AATT-------AGATAC------------ATGAAAACAA----TTCAATCCCA

pNcHMA4-1-SLM -------GAAT-------AAGTGT------------TTGTGAACAA----AATA-TCTTG

pNcHMA4-2-SLM -------AATT-------GGATAC------------ATGAAAACAA----TTCAATCCCA

pNcHMA4-3-SLM ATGGTTTAATT-------AATTTTTAATTTTTAATAATGAAAATAG----TTTAATTAAA

pNcHMA4-4-SLM -------GAAT-------AAGTGT------------TTGTGAACAA----AATA-TCTTG

pAhHMA4-1 -------AAAACCTAGCAAGAATT-----------ATTAATAATAATATGGTTATTCAAA

pAhHMA4-2 -------AATTTTTTGGTAAATTTT------------TGAAAACAAAAGATGAAATTTGG

pAhHMA4-3 -------AATTTTTTGGTAAATTTT------------TGAAAACAAAAGATGAAATTTGG

pAtHMA4 AAC--------TTGAAG-----ATAAGTTTGATCA---AGGCCG-----ACCCATGGGTA

pNcHMA4-1-LC CAC----CAAACC--AGAA---ATCGTTTTAATTT--CTAGCTTCA--GTTTCAGAAACC

pNcHMA4-2-LC CAC----CAAACC--AGAA---ATCGTTTTAATTT--CTAGCTTCA--GTTTCAGAAACC

pNcHMA4-3-LC CAC----CAAACC--AGAA---ATCGTTTTAATTT--CTAGCTTCA--GTTTCAGAAACC

pNcHMA4-1-Ga TACTGTTAGAACG--AGAAA--ATTACATCAATAA---GGACTTTT-TGACTTAT---CT

pNcHMA4-2-Ga TACTGTTAGAACG--AGAAA--ATTACATCAATAA---GGACTTTT-TGACTTAT---CT

pNcHMA4-3-Ga CAC----CAAACC--AGAA----TCGTTTTAATTT--ATAGCTTCA--GTTTCAGAAATT

pNcHMA4-1-CMA CAC----CAAACC--AGAA---ATCGTTTTAATTT--ATAGCTTCA--GTTTCAGAAATT

pNcHMA4-2-CMA CAC----CAAACC--AGAA---ATCGTTTTAATTT--ATAGCTTCA--GTTTCAGAAATT

pNcHMA4-1-SLM GAT----AGAATTTTAGAATATATCACGTAGATCG--TGGACAATA--GGTGTGTGAACA

pNcHMA4-2-SLM CAC----CAAACC--AGAA---ATCGTTTTAATTT--CTAGCTTCA--GTTTCAGAAACC

pNcHMA4-3-SLM AAT----AAAACCT-------TATCTAGTTAATTTGAAGGACAAAAATGGATTATGTGAT

pNcHMA4-4-SLM GAT----AGAATTTTAGAATATATCACGTAGATCG--TGGACAATA--GGTGTGTGAACA

pAhHMA4-1 TTC--------TT--AG-----ATTA---TGAT-----ATGCTATA--TATTAATGTGCC

pAhHMA4-2 AACT-------CT----------TCGAATTATTCAAGAAAACAATT----CTAATGAACT

pAhHMA4-3 AACT-------CT----------TCGAATTATTCAAGAAAACAATT----CTAATGAACT

pAtHMA4 AAAAGCC-CAACA-AGCGAAT---------------------------------------

pNcHMA4-1-LC AATTTACGCCAA-----AAAAAAAAAAAAAAAAAAAAAAAAAAAAAAAAAAATTTACGCC

pNcHMA4-2-LC AATTTATGCCAA-----AAA----------------------------------------

pNcHMA4-3-LC AATTTATGCCAA-----AAA----------------------------------------

pNcHMA4-1-Ga TAT-TACAGCAAG--AAGGA----------------------------------------

pNcHMA4-2-Ga TAT-TACAGCAAG--AAGGA----------------------------------------

pNcHMA4-3-Ga AATATACGCCAA-----AAA----------------------------------------

pNcHMA4-1-CMA AATATACGCCAA-----AAA----------------------------------------

pNcHMA4-2-CMA AATATACGCCAA-----AAA----------------------------------------

pNcHMA4-1-SLM TATATATATATAT--TCAGA----------------------------------------

pNcHMA4-2-SLM AATTTACGCCAA-----AAA----------------------------------------

pNcHMA4-3-SLM AAAAAAGGTGTAA--GGGGA----------------------------------------

pNcHMA4-4-SLM TATATATATATAT--TCAGA----------------------------------------

pAhHMA4-1 TAAACTCGTGACGTATCGGACCA-------------------------------------

pAhHMA4-2 CAT-TATACAAAT--TCTGA----------------------------------------

pAhHMA4-3 CAT-TATACAAAT--TCTGA----------------------------------------

pAtHMA4 ----GCTTTAG----GCAACAACGTTTATA-----AATGTTTTTAGCAGCCTCACTAACC

pNcHMA4-1-LC AAAAACGTTGG--------TTACTGTGAAAC--ACA----C---ACCAAACCAGA-AATC

pNcHMA4-2-LC -----CATTGG--------TTACTGTGAAAC--ACATGCAC---ACCAAACCAGA-AATC

pNcHMA4-3-LC -----CATTGG--------TTACTGTGAAAC--ACATGCAC---ACCAAACCAGA-AATC

pNcHMA4-1-Ga -----CAGTG---------TAACTATGGTGC--ACTTTGCCTTAACAAAAATACATTGTT

pNcHMA4-2-Ga -----CAGTG---------TAACTATGGTGC--ACTTTGCCTTAACAAAAATACATTGTT

pNcHMA4-3-Ga -----CGTTGGT---TTGGTTACTGTGAAAC--ACA----C---ACCAAACCAAA-AATC

pNcHMA4-1-CMA -----CGTTGG--------TTACTGTGAAAC--ACA----C---ACCAAACCAAA-AATC

pNcHMA4-2-CMA -----CGTTGG--------TTACTGTGAAAC--ACA----C---ACCAAACCAAA-AATC

pNcHMA4-1-SLM -----CAT----------ATAACAATAAAAT--ATG----T---ACACAAGCTTC-GATT

pNcHMA4-2-SLM -----CGTTGT--------TTACTGTGAAAC--ACA----C---ACCAAACCAAA-AATC

pNcHMA4-3-SLM -----CAAAGA-------GCAAATGTGAAAG--AGT----A---ATGGGACAAGATAGGT

pNcHMA4-4-SLM -----CAT----------ATAACAATAAAAT--ATG----T---ACACAAGCTTC-GATT

pAhHMA4-1 ----GTTTTAGTCTCGCCCTACTTTTCAAACTAATAATGTTTT--GTAACCTCAATACTT

pAhHMA4-2 -------------------TAGCTGCAAAAA--ATA----TTAAAGAAAACCAACCAATC

pAhHMA4-3 -------------------TAGCTGCAAAAA--ATA----TTAAAGAAAACCAACCAATC

pAtHMA4 ATC-------AAGAGT------TTTG-CTTTATTTG---TTATCATATTCTTTTTCGCAG

pNcHMA4-1-LC ACCAT-CAACAGAAAT----AGCCAA-ATTCATCCA---CAATTGTATAAGTAACC-G--

pNcHMA4-2-LC ACCAT-CAACAGAAAT----AGCCAA-ATTCATCCA---CAATTGTATAAGTAACC-GG-

pNcHMA4-3-LC ACCAT-CAACAGAAAT----AGCCAA-ATTCATCCA---CAATTGTATAAGTAACC-GG-

pNcHMA4-1-Ga GCTCT-TTTTATTTGTTT-TACC-----TTTACACAATTTAACTTTATATTTTACC-AGT

pNcHMA4-2-Ga GCTCT-TTTTATTTGTTT-TACC-----TTTACACAATTTAACTTTATATTTTACC-AGT

pNcHMA4-3-Ga ACCAT-CA-CAGAAAT----AGCCAA-ATTTATCCA---CAATTGTATAAGTAACC-G--

pNcHMA4-1-CMA ACCAT-CA-CAGAAAT----AGCCAA-ATTTATCCA---CAATTGTATAAGTAACC-G--

pNcHMA4-2-CMA ACCAT-CA-CAGAAAT----AGCCAA-ATTTATCCA---CAATTGTATAAGTAACC-G--

pNcHMA4-1-SLM TTCAC-CT-CTGATAC----AAC--A-ATTCATCCA---CATCTTTATTA-TGTCC-A--

pNcHMA4-2-SLM ACCAT-CA-CAAAAAT----AGCCAA-ATTCATCCA---TAATTGTATAAGTAACC-G--

pNcHMA4-3-SLM TTCTT-TT-AGGGTATTT-TGGC--A-ATTTTCTCA---TGATTATGTGA---ATT-A--

pNcHMA4-4-SLM TTCAC-CT-CTGATAC----AAC--A-ATTCATCCA---CATCTTTATTA-TGTCC-A--

pAhHMA4-1 TTCCT-TTGTAACCAT------TTTA-AATTAATTA---TTAATAAAAAACT----GCCG

pAhHMA4-2 ATCTTTCATCTAACATCCGTAGCTGGCGCTCGCGGA--TCTATGGTTGAAGTGATTTGAT

pAhHMA4-3 ATCTTTCATCTAACATCCGTAGCTGGCGCTCGCGGA--TCTGTGGTTGAAGTGATTTGAT

pAtHMA4 TATA----------CACTATAAAGA------TGCGGTCAT-ATTATCTATATTTTTTG--

pNcHMA4-1-LC TA------------CA-AACGTA--------TATAACAA--AGCGTACACATATT-----

pNcHMA4-2-LC TAAAGTAACCGGTACA-AACGTA--------TATAACAA--GGCGTACACATATT-----

pNcHMA4-3-LC TA------------CA-AACGTA--------TATAACAA--GGCGTACACATATT-----

pNcHMA4-1-Ga TA------------CACAATTTATCTCTCTCCACGATAA--ATCCTTCTCTCTCTTC---

pNcHMA4-2-Ga TA------------CACAATTTATCTCTCTCCACGATAA--ATCCTTCTCTCTCTTC---

pNcHMA4-3-Ga TA------------CA-AACGTA--------TATAACAA--AGCCTACACATATT-----

pNcHMA4-1-CMA TA------------CA-AACGTA--------TATAACAA--AGCCTACACATATT-----

pNcHMA4-2-CMA TA------------CA-AACGTA--------TATAACAA--AGCCTACACATATT-----

pNcHMA4-1-SLM AA------------CA-AAAGTAA-----TCCACGGCAATAAATCTACATAATTTGG---

pNcHMA4-2-SLM TA------------CA-AACGTA--------TATAACAA--AGCGTACACATGTT-----

pNcHMA4-3-SLM TA------------GA-AATTCA--------TGTGATTTT--GGTTAAAT-CATT-----

pNcHMA4-4-SLM AA------------CA-AAAGTAA-----TCCACGGCAATAAATCTACATAATTTGG---

pAhHMA4-1 TAT-----------CAAAATAAAT-------TAAGATCG--AGCATCTAC---CTCGA--

pAhHMA4-2 TGAGC---------CACTTCTCGC----TTCTACCGCCGTGAGTTTGCTTCCACCGGGAC

pAhHMA4-3 TGAGC---------CACTTCTCGC----TTCTACCGCCGTGAGTTTGCTTCCACCGGGAC

pAtHMA4 ---------TAACG-ATTTGGCTGGGACTG-------TTT--------------------

pNcHMA4-1-LC --------TTCAA----TTGTCCAAAAACA------CATT-------GTACTCC----AA

pNcHMA4-2-LC --------TTCAA----TTGTCCAAACACA------CATT-------GTACTCC----AA

pNcHMA4-3-LC --------TTCAA----TTGTCCAAACACA------CATT-------GTACTCC----AA

pNcHMA4-1-Ga --TTTTCTTTCTCCACTTTATTCATCTCCA------CTTT-----CCTTATCTCT-----

pNcHMA4-2-Ga --TTTTCTTTCTCCACTTTATTCATCTCCA------CTTT-----CCTTATCTCT-----

pNcHMA4-3-Ga --------TTGAA----TTGTCCAAAAACA------CATT-------TTACTCC----AA

pNcHMA4-1-CMA --------TTGAA----TTGTCCAAAAACA------CATT-------TTACTCC----AA

pNcHMA4-2-CMA --------TTGAA----TTGTCCAAAAACA------CATT-------TTACTCC----AA

pNcHMA4-1-SLM --CTCTCTTTGAT----TCTTCCACTCTTA------CTTTCATCTTTTTATTTCCG-TAA

pNcHMA4-2-SLM --------TTCAA----TTGTCCAAAAACA------CATT-------TTACTAC----AA

pNcHMA4-3-SLM --------CTAAAATC-TTATCTAAAACCAAGTGA-TTTTGAGTCTTTTATTTTTAACAA

pNcHMA4-4-SLM --CTCTCTTTGAT----TCTTCCACTCTTA------CTTTCATCTTTTTATTTCCG-TAA

pAhHMA4-1 ---------TAAT--ACTTGGTTTCAACT---------TT--------------------

pAhHMA4-2 GGTTGGATTTGAT--CGTTGCTCGGAGGTAGGAAAACGTC-------TTAACGGGAGGGG

pAhHMA4-3 GGTTGGATTTGAT--CGTTGCCCGGAGATAGAAAAACGTC-------TTAACAG-AGGGG

pAtHMA4 -----------GTGGACCA----------CGACGTC-----TATTTTTGGTTGA-TGGAT

pNcHMA4-1-LC TTA------AACGAAGCTA---------CCAGATCC----ATGTCCT-CTCCCAGAACAG

pNcHMA4-2-LC TTA------AACGAAGCTA---------CCAGATCC----ATGTCCT-CTCCCAGAACAG

pNcHMA4-3-LC TTA------AACGAAGCTA---------CCAGATCC----ATGTCCT-CTCCCAGAACAG

pNcHMA4-1-Ga TTGCTTC-CTACAAATTCTCTGTCTTTCTCATTTTCCACTAGGTTCTTCTTCCACCA---

pNcHMA4-2-Ga TTGCTTC-CTACAAATTCTCTGTCTTTCTCATTTTCCACTAGGTTCTTCTTCCACCA---

pNcHMA4-3-Ga TTA------AACGAAGCTA---------TCAGATCC----ATGTCCT-CTACCAAAACAG

pNcHMA4-1-CMA TTA------AACGAAGCTA---------TCAGATCC----ATGTCCT-CTACCAAAACAG

pNcHMA4-2-CMA TTA------AACGAAGCTA---------TCAGATCC----ATGTCCT-CTACCAAAACAG

pNcHMA4-1-SLM TCACAAG-CAACAATGTT----------TCATTTTC----ACTTCTCGTTATCATCATAT

pNcHMA4-2-SLM TTA------AACGAAGCTA---------TCAGATCC----ATGTCCT-CTCCCAGAAAAG

pNcHMA4-3-SLM GAAAATC-TCACAAAATCACT-------CTAAAATC----AAATCTAATTTTAAAAATCT

pNcHMA4-4-SLM TCACAAG-CAACAATGTT----------TCATTTTC----ACTTCTCGTTATCATCATAT

pAhHMA4-1 -----------CAAGACTAA--------TTAAGATC-----GAGTTTTTTTTAAATAAAG

pAhHMA4-2 TTGTGTTATAACATAGTGGGTTAGGTTGCTAGGCAC----GGAACCTAACTCTGGTAGAG

pAhHMA4-3 TTGTGTTATAACCCAGTGGGTTAGGGTGCTAGGCAC----GGAACCTAACTCTGGTAGAG

pAtHMA4 TTCTACA-ATATAC-------GATACTGAAAA-------GAG--------TAAAAATCAT

pNcHMA4-1-LC A---AAATAAACACTC---------GTCAAGA------TAA--------CCAAATATCAT

pNcHMA4-2-LC A---AAATAAACACTC---------GTCAAGA------TAA--------CCAAATATCAT

pNcHMA4-3-LC A---AAATAAACACTC---------GTCAAGA------TAA--------CCAAATATCAT

pNcHMA4-1-Ga ------ATAAAGATTG--------GGACAAGGGTTGCGTAA----------AGACATTAT

pNcHMA4-2-Ga ------ATAAAGATTG--------GGACAAGGGTTGCGTAA----------AGACATTAT

pNcHMA4-3-Ga A---AAATAAACACT---------AGTCAAGA------TAA--------CCAAATATCAT

pNcHMA4-1-CMA A---AAATAAACACT---------AGTCAAGA------TAA--------CCAAATATCAT

pNcHMA4-2-CMA A---AAATAAACACT---------AGTCAAGA------TAA--------CCAAATATCAT

pNcHMA4-1-SLM A---TTACAATTTTTT--------ACCCATGT------TAA--------CCC--TATCAA

pNcHMA4-2-SLM A---AAATAAACACTC---------GTCAAGA------TAA--------CCA-------T

pNcHMA4-3-SLM ACTTTTAAAAATATTTCTAATAACAGTCGATT------TGAAAGTGGATTTTAAAATCAC

pNcHMA4-4-SLM A---TTACAATTTTTT--------ACCCATGT------TAA--------CCC--TATCAA

pAhHMA4-1 TCATGCACAAATATTGATTTAAATATTTATACTT---ATGAG--------TTATGACAAT

pAhHMA4-2 CCGGTCATCGGTGTGTT-----AGAACCGGGAGCTCCGTAG---------CGGGTCAGGT

pAhHMA4-3 TCGGTCGTCGGTGTGTT-----AGAACCGGGAGCTCCGTAG---------CGGGTCAGGT

pAtHMA4 TTATTTTATTATA-------AAAATAA--------------TGTC----TCCAACTTTC-

pNcHMA4-1-LC ATTATTTATGA-A-------TAAGTAAAAGTGTAAAACTCATGCG---ATGCAATTTC--

pNcHMA4-2-LC ATTATTTATGA-A-------TAAGTAAAAGTGTAAAACTCATGCG---ATGCAATTTC--

pNcHMA4-3-LC ATTATTTATGA-A-------TAAGTAAAAGTGTAAAACTCATGCG---ATGCAATTTC--

pNcHMA4-1-Ga TATGCCT-TGA-A-------TAAGTG-----------TTTGTGTATGCATGCCCTTTT--

pNcHMA4-2-Ga TATGCCT-TGA-A-------TAAGTG-----------TTTGTGTATGCATGCCCTTTT--

pNcHMA4-3-Ga ATTATTTATGA-A-------TAAGTAAAA--------CTCATGCG---ATGCAATTTC--

pNcHMA4-1-CMA ATTATTTATGA-A-------TAAGTAAAA--------CTCATGCG---ATGCAATTTC--

pNcHMA4-2-CMA ATTATTTATGA-A-------TAAGTAAAA--------CTCATGCG---ATGCAATTTC--

pNcHMA4-1-SLM AACACCAATTAGA-------TACATGAAA-------------ACA---ATTCAATCCCA-

pNcHMA4-2-SLM ATTATTTATGA-A-------TAAGTAAAA--------TTCATGCG---ATGCAATTTC--

pNcHMA4-3-SLM AAATTCAATAACACTGAATTTTAATAGGG--------TTTTTAGA---ATTCAAGTTTGA

pNcHMA4-4-SLM AACACCAATTAGA-------TACATGAAA-------------ACA---ATTCAATCCCA-

pAhHMA4-1 TTGT---ATTAAA-------AAAAAAA--------------CTTAG---TTCAAATTT--

pAhHMA4-2 GAGCCTTAGGGGA-------GGAGGAGGAG----------GTGAG---ACTCAAACAC--

pAhHMA4-3 GAGTCTTAGGGGA-------GGAGGAAGAG----------GTGAG---ACTCAAACAC--

pAtHMA4 ---TCTTGTAATAGGTTATGGCTTGTATTCACT----------------GATTC------

pNcHMA4-1-LC ----CTCCAGCC--GTATCAGTCTCTAAGTGG----------------AAATTG------

pNcHMA4-2-LC ----CTCCAGCC--GTATCAGTCTCTAAGTGG----------------AAATTG------

pNcHMA4-3-LC ----CTCCAGCC--GTATCAGTCTCTAAGTGG----------------AAATTG------

pNcHMA4-1-Ga -----TCAAAAAAAAAAAAAAAGAATAAGTGT-------------------TTG------

pNcHMA4-2-Ga -----TCAAAAAAAAAAAAAAAGAATAAGTGT-------------------TTG------

pNcHMA4-3-Ga ----CTCCAGCC--GTATCAGTCTTTATGTGGT---------GCGGTCGAGTT-------

pNcHMA4-1-CMA ----CTCCAGCC--GTATCAGTCTCTATGTGGT---------GCGGTCGAGTT-------

pNcHMA4-2-CMA ----CTCCAGCC--GTATCAGTCTCTATGTGGT---------GCGGTCGAGTT-------

pNcHMA4-1-SLM ----CACCAAACCAGAAATCGTTTTAATTTATA---------GC--TTCAGTTTCAG---

pNcHMA4-2-SLM ----CTCCAGCC--GTATCAGTCTCTACGTGGTGCGGTCGCCGGAGTTAAATTAAAAGAC

pNcHMA4-3-SLM ATAACACTAAAT---TTGTTATTTTAATATAAATCA----CCGTAAATCACTTC------

pNcHMA4-4-SLM ----CACCAAACCAGAAATCGTTTTAATTTATA---------GC--TTCAGTTTCAG---

pAhHMA4-1 --------TAAGAAGTT---CCCTATACTGAAA----------------AAAT-------

pAhHMA4-2 ----TCCCGTCGACAACTCTACCCCTATAGGCA---------------------------

pAhHMA4-3 ----TCCCATCGACATCTCTACCCCTATACGCA---------------------------

pAtHMA4 ----------------TTAATGC-------------------------------------

pNcHMA4-1-LC ----------------TGA-----------------------------------------

pNcHMA4-2-LC ----------------TGA-----------------------------------------

pNcHMA4-3-LC ----------------TGA-----------------------------------------

pNcHMA4-1-Ga ----------------TGA-----------------------------------------

pNcHMA4-2-Ga ----------------TGA-----------------------------------------

pNcHMA4-3-Ga -----------AAAT-TAAAA---------------------------------------

pNcHMA4-1-CMA -----------AAAT-TAAATTC-------------------------------------

pNcHMA4-2-CMA -----------AAAT-TAAATTC-------------------------------------

pNcHMA4-1-SLM -----------AAAT-TAATAT--------------------------------------

pNcHMA4-2-SLM ACCAACCAAAATAATCTGATTTTTCACCCCTTTTTCATTAAAGGTAAAATGGAACTCTCA

pNcHMA4-3-SLM -----------AAATCTCAATTT-------------------------------------

pNcHMA4-4-SLM -----------AAAT-TAATAT--------------------------------------

pAhHMA4-1 ----------------TAAATG--------------------------------------

pAhHMA4-2 -----------------AA-----------------------------------------

pAhHMA4-3 -----------------AA-----------------------------------------

pAtHMA4 -------------------------------ATTCTAG----------------TATAAC

pNcHMA4-1-LC -------------------------AAGTCCACCA---------------CTTTCACAAT

pNcHMA4-2-LC -------------------------AAGTCCACCA---------------CTTTCACAAT

pNcHMA4-3-LC -------------------------AAGTCCACCA---------------CTTTCACAAT

pNcHMA4-1-Ga -------------------------------AC----------------------AAAAT

pNcHMA4-2-Ga -------------------------------AC----------------------AAAAT

pNcHMA4-3-Ga ----------------------------GACACCA-------------ACC----AAAAT

pNcHMA4-1-CMA -------------------------AATGACACCA-------------ACC----AAAAT

pNcHMA4-2-CMA -------------------------AATGACACCA-------------ACC----AAAAT

pNcHMA4-1-SLM -----------------------------ACGCCA-------------A------AAAAC

pNcHMA4-2-SLM ACACACTATAAGCAAAAAAGAAAAAAAGTCCACCA---------------CTTTCACAAT

pNcHMA4-3-SLM -------------------------AAATACACCATTTCCTTTCTTAAACTTCTAAGAAC

pNcHMA4-4-SLM -----------------------------ACGCCA-------------A------AAA-C

pAhHMA4-1 -------------------------------ATCAT--------------------CAAT

pAhHMA4-2 -------------------------------GCCG------------------TGGAGAT

pAhHMA4-3 -------------------------------GCCA------------------TGGAGAT

pAtHMA4 ----TTTATGTCATTTATTTATTTAATTTAGTGGT------------------TTATC-A

pNcHMA4-1-LC -----TTCC------CTCTTATAAAAGTCACTCTTACTGTAAATGAC-------TCCT-G

pNcHMA4-2-LC -----TTCC------CTCTTATAAAAGTCACTCTTACTGTAAATGAC-------TCCT-G

pNcHMA4-3-LC -----TTCC------CTCTTATAAAAGTCACTCTTACTGTAAATGAC-------TCCT-G

pNcHMA4-1-Ga ---------------ATCTTG---GATAGAATTTTAGAATA------------TGATT-G

pNcHMA4-2-Ga ---------------ATCTTG---GATAGAATTTTAGAATA------------TGATT-G

pNcHMA4-3-Ga --AATCTG------ATTTTT--------CACCTCT------------------TTTTC-A

pNcHMA4-1-CMA --AATCTG------ATTTTT--------CACCCCT------------------TTTTC-A

pNcHMA4-2-CMA --AATCTG------ATTTTT--------CACCCCT------------------TTTTC-A

pNcHMA4-1-SLM --G--TTG------GTTACTGTGAAACACACACCAAACCAAAAA---------TCACC-A

pNcHMA4-2-SLM -----TTCC------CTCTTATAAAAGTCACTCTTAGTGTAAATGACAAATGACTCCT-G

pNcHMA4-3-SLM CAAATTTGTGGTATATATTTG------ATATATTTAATCTATTT---------TTTTC-T

pNcHMA4-4-SLM --G--TTG------GTTACTGTGAAACACACACCAAACCAAAAA---------TCACC-A

pAhHMA4-1 ----TCT---TTTTTTTTTTTTTTAGACTAGTCCA------------------TAATCCA

pAhHMA4-2 CGTGTTTGCTG-GTGCTACTGTCTGATTCACTGCTTC----------------TGGTC-G

pAhHMA4-3 CGTGTTTGCTG-GTGCTACTGTCTGATTCACTGCTTC----------------TGGTC-G

pAtHMA4 TT-TAAGCCGCATACGTCTTTACTCTATCTCTTTCTCCTAGAGTATAACTATAAGTTATA pNcHMA4-1-LC TTAGAACTACCTAAAAGCAGGTTATGGAATTCG-TTGGTCAAATAAATT--TGAAAAAAA

pNcHMA4-2-LC TTAGAACTACCTAAAAGCAGGTTATGGAATTCG-TTGGTCAAATGAATT--TGAAAAAAA

pNcHMA4-3-LC TTAGAACTACCTAAAAGCAGGTTATGGAATTCG-TTGGTCAAATGAATT--TGAAAAAAA

pNcHMA4-1-Ga ACAAA----AAAAAAAAAGAATTTTAGAATATATCACGTAGATCGTGGA--CAATATGCC

pNcHMA4-2-Ga ACAAA----AAAAAAAAAGAATTTTAGAATATATCACGTAGATCGTGGA--CAATATGCC

pNcHMA4-3-Ga TTAAA----GGTAAAA--------TGGAACTC--TCAACACACTATAAG--CAAAAAAAA

pNcHMA4-1-CMA TTAAA----GGTAAAA--------TGGAACTC--TCAACACACTATAAG--CAAAAAAAA

pNcHMA4-2-CMA TTAAA----GGTAAAA--------TGGAACTC--TCAACACACTATAAG--CAAAAAAAA

pNcHMA4-1-SLM TCACA----GAAATAG--------CCAAATTTA-TCCACA-ATTGTA----TAAGAAAAA

pNcHMA4-2-SLM TTAGAACTACCTAAAAGCAGGTTATGGAATTCG-TTGGTCAAATGAATT--AAAAAAAAA

pNcHMA4-3-SLM TCCAACTTTGATCTAA--------TTGAATTTTGTTGGTG-ATTTTGGA--AAAACAAAA

pNcHMA4-4-SLM TCACA----GAAATAG--------CCAAATTTA-TCCACA-ATTGTA----TAAGAAAAA

pAhHMA4-1 TTATGGATTGAAAACGTTGTCAATATATATATATATAT----ATGTAAA-GTAAAATGTT

pAhHMA4-2 TCAGC--------AGG-------TTCCGATCTA-CTCCTCCGGTGAA-----GACACAGT

pAhHMA4-3 TCAGC--------AGG-------TTCCGATCTA-CTCCTCCGGTGAA-----GACACAGT

pAtHMA4 CTATGCAA---------ACATGTGAG---------------ATTTTAT-TAGAAAGTATA

pNcHMA4-1-LC AA--CAAA-----------TTATAAATT-------------GTT-----ATGAAAGAA-A

pNcHMA4-2-LC AA--CAAA-----------TTATAAATT-------------GTT-----ATGAAAGAA-A

pNcHMA4-3-LC AA--CAAA-----------TTATAAATT-------------GTT-----ATGAAAGAA-A

pNcHMA4-1-Ga ATGGACAA------------TATGGGT--------------GT------GTGAACATA-T

pNcHMA4-2-Ga ATGGACAA------------TATGGGT--------------GT------GTGAACATA-T

pNcHMA4-3-Ga AA--AAAA----AAAAAGTCTTTGCGT-------AAAGAGAGTT-----ATAAAAGTA-A

pNcHMA4-1-CMA AA--AAAA----AAAA--TCTTTGCGT-------AAAGAGAGTT-----ATAAAAGTA-A

pNcHMA4-2-CMA AA--AAAA----AAAA--TCTTTGCGT-------AAAGAGAGTT-----ATAAAAGTA-A

pNcHMA4-1-SLM AA--AAAA----AAAAAGTCTTTGCGT-------AAGGAGAGTT-----ATAAAAGTA-A

pNcHMA4-2-SLM AAAACAAA-----------TTATAAATT-------------GTT-----ATGAAAGAA-A

pNcHMA4-3-SLM TA--TAAATTTCGGCACTGTTATGGGTCAATCTCAAAAAAGGTTTTCCTACCAAACTC-A

pNcHMA4-4-SLM -----------------GTCTTTGCGT-------AAGGAGAGTT-----ATAAAAGTA-A

pAhHMA4-1 TCATA-AA---------ATATATGA----------------ACTGAATGTTGAAAATAGG

pAhHMA4-2 TTTTCCGA------------TTTCCATT--------------TT-----------GTA-A

pAhHMA4-3 TTTTCCGA------------TTTCCATT--------------TT-----------GTA-A

pAtHMA4 AAA-GATATAGG---TAGATATTAA---GAGA--TAAGCT-ACGAAAGTAGAC-------

pNcHMA4-1-LC ATATCTCGTACA---CAAGTGTCA--CCAAG---TG------------------------

pNcHMA4-2-LC ATATCTCGTACA---CAAGTGTCA--CCAAG---TG------------------------

pNcHMA4-3-LC ATATCTCGTACA---CAAGTGTCA--CCAAG---TG------------------------

pNcHMA4-1-Ga ATATATT-CAGA---CATATAACA--ATAAAATATGTAC-------ACAAGC--------

pNcHMA4-2-Ga ATATATT-CAGA---CATATAACA--ATAAAATATGTAC-------ACAAGC--------

pNcHMA4-3-Ga ATATCTCGTACA---CAAGTGTCA--CCAAG---TGAAC--ATAC-ATTAGTCAATGTTA

pNcHMA4-1-CMA ATATCTCGTACA---CAAGTGTCA--CCAAG---TGAAC--ATAC-ATTAGTCAATGTTA

pNcHMA4-2-CMA ATATCTCGTACA---CAAGTGTCA--CCAAG---TGAAC--ATAC-ATTAGTCAATGTTA

pNcHMA4-1-SLM ATTTCTCGTACA---CAAGTGTCA--CCAAG---TGAAC--ATAC-ATTAGTCAATGTTA

pNcHMA4-2-SLM ATATCTCGTACA---CAAGTGTCA--CCAAG---TA------------------------

pNcHMA4-3-SLM AACTGGAATAAA---CACCGGTTC--CCGGT---TAGACCGGTCCGACCAGCCGGTCCTA

pNcHMA4-4-SLM ATTTCTCGTACA---CAAGTGTCA--CCAAG---TGAAC--ATAC-ATTAGTCAATGTTA

pAhHMA4-1 AACTGGCGTGAGAATTAGTTCTTG----GAGA--TAGG-------AATTGGCCT------

pAhHMA4-2 TTGTCTC-TGCT---TTCATATTGGGCCAAAGCCCAGCC---------------------

pAhHMA4-3 TTGTCTC-TGCG---TTCATATTAGGTCAAGGCCCAACC---------------------

pAtHMA4 -----GACATTGGTGCGTTGCGTTAA-------------AAGAATTTGTAGTTAATTATT

pNcHMA4-1-LC ----------TAA--------------------------GGG-ACCCAAATCT--TTGTT

pNcHMA4-2-LC ----------TAA--------------------------GGG-ACCCAAATCT--TTGTT

pNcHMA4-3-LC ----------TAA--------------------------GGGGACCCAAATCT--TTGTT

pNcHMA4-1-Ga --------TTCGATTTTCACCTCTG---ATACACA----ATTCATCCACATCT--TTATT

pNcHMA4-2-Ga --------TTCGATTTTCACCTCTG---ATACACA----ATTCATCCACATCT--TTATT

pNcHMA4-3-Ga ----GTATTTTAATATTCTCTTTTAGCTATATATA----AGGGACCC--ATCT--TTGTT

pNcHMA4-1-CMA ----GTATTTTAATATTCTCTTTTAGCTATATATA----AGGGACCC--ATCT--TTGTT

pNcHMA4-2-CMA ----GTATTTTAATATTCTCTTTTAGCTATATATA----AGGGACCC--ATCT--TTGTT

pNcHMA4-1-SLM ----GTATTTTAATATTCTCTTTTAGCTATATATA----AGGGACCC--ATCT--TTGTT

pNcHMA4-2-SLM ----------TAA--------------------------GGG-ACCC--ATCT--TTGTT

pNcHMA4-3-SLM TCTGATTTTTTAATTATTGTTTTTAGCTATATATATATAAGGGACTC--ATTT--TTGTT

pNcHMA4-4-SLM ----GTATTTTAATATTCTCTTT-AGCTATATATA----AGG-ACCC--ATCT--TTGTT

pAhHMA4-1 ----GGAAATTAGGAAG----GATAT-------------AGGAATT-GAAAACAAATAAC

pAhHMA4-2 ------CTTTTGGGAGATCCAT------------------GTAAC-----TTT--TTATT

pAhHMA4-3 ------CTTTTGGGAGGTCCAT------------------GTAACCTTTTTTT--TTATT

pAtHMA4 T------------AGAATGATAAACTAAGA-AAATCGTAC--TAAAAACAATAA---TAA

pNcHMA4-1-LC -----G------AAAGAAGATGA--------AGTTAACAA---AAATACTTTTGCCTTCT

pNcHMA4-2-LC -----G------AAAGAAGATGA--------AGTTAACAA---AAATACTTTTGCCTTCT

pNcHMA4-3-LC -----G------AAAGA-GATGA--------AGTTAACAA---AAATACTTTTGCCTTCT

pNcHMA4-1-Ga -----ATGTCCAAACAAAAGTAATCCACGGCAATTAATCT---ACATAATTTGGCTTTCT

pNcHMA4-2-Ga -----ATGTCCAAACAAAAGTAATCCACGGCAATTAATCT---ACATAATTTGGCTTTCT

pNcHMA4-3-Ga -----G------AAAGAAGATGA--------AGTTCACAA---AAAAACTTTTGCCTTCT

pNcHMA4-1-CMA -----G------AAAGAAGATGA--------AGTTCACAA---AAAAACTTT-GCCTTCT

pNcHMA4-2-CMA -----G------AAAGAAGATGA--------AGTTCACAA---AAAAACTTTTGCCTTCT

pNcHMA4-1-SLM -----G------AAAGAAGATGA--------AGTTCACAA---AAAAACTTTTGCCTTCT

pNcHMA4-2-SLM -----G------AAAGAAGATGA--------AGTTAACAA---AAAAACTTTTGCCTTCT

pNcHMA4-3-SLM -----G------AAAGAAGATAA--------AGTTAACAA---AAAAACTTTTGCCTTCT

pNcHMA4-4-SLM -----G------AAAGAAGATGA--------AGTTCACAA---AAAAACTTTTGCCTTCT

pAhHMA4-1 T------------AAAA--ATAGCCTACGA-AATTTACATGGTAAAAA-AATAACTTTAA

pAhHMA4-2 TATTTATT-----AAAAAAAAAA---------GTTAA-AA---AAAATCTGTCGCGATAC

pAhHMA4-3 TATTTATTTATTAAAAAAAAAAA---------GTTAG-AA---AAAACCTGTCGCGATAC

pAtHMA4 AG-----AAAAC----AATGAGATGAGG----AATCACACATGGATTCC-----------

pNcHMA4-1-LC CTCT-------CTCTCCATCGAATACTA---TTATCCCACTTTGCTTCC-----C-----

pNcHMA4-2-LC CTCT-------CTCTCCATCGAATACTA---TTATCCCACTTTGCTTCC-----C-----

pNcHMA4-3-LC CTCT-------CTCTCCATCGAATACTA---TTATCCCACTTTGCTTCC-----C-----

pNcHMA4-1-Ga CTTTG---ATTCT-----TCCACTCTTACTTTCATCTT--TTTATTTCCGTAATCACAAG

pNcHMA4-2-Ga CTTTG---ATTCT-----TCCACTCTTACTTTCATCTT--TTTATTTCCGTAATCACAAG

pNcHMA4-3-Ga CTCT-------CT----ATCGAATACTG---TTATCTCACTTTCCTTCC-----CTTTTC

pNcHMA4-1-CMA CTCT-------CT----ATCGAATACTG---TTATCTCACTTTCCTTCC-----CTTTTC

pNcHMA4-2-CMA CTCT-------CT----ATCGAATACTG---TTATCTCACTTTCCTTCC-----CTTTTC

pNcHMA4-1-SLM CTCT-------CT----ATCGAATACTG---TTATCCCACTTTCCTTCC-----CTTTTC

pNcHMA4-2-SLM CTCT-------CTCTCCATCGAATACTACTATTATCCCACTTTCCTTCC-----C-----

pNcHMA4-3-SLM CTTT-------CG----ATCGAATACTA---TC-TCCCACTTTCCTTC------------

pNcHMA4-4-SLM CTCT-------CT----ATCGAATACTG---TTATCCCACTTTCCTTCC-----CTTTTC

pAhHMA4-1 ACCCCATAAAACCTTTAATAAAA-GAAA----AACAACATTTAAAATA------------

pAhHMA4-2 TTAC-------CA----AACAAATCTTT---TGATATGAATATTTAATA-----------

pAhHMA4-3 TTAC-------CA----AACAAATCTTT---TGATATGAATATTTAATA-----------

pAtHMA4 ------ATTTTCGTGA--------CATTCCACTTTTGGGGGTTTCCACTAAATTTTTTAT

pNcHMA4-1-LC ------TCTCTC-T------------TTTAATATCTCAC-CTTTATAATAAAAAGCTGAT

pNcHMA4-2-LC ------TCTCTC-T------------TTTAATATCTCAC-CTTTATAATAAAAAGCTGAT

pNcHMA4-3-LC ------TCTCTC-T------------TTTAATATCTCAC-CTTTATAATAAAAAGCTGAT

pNcHMA4-1-Ga CAACAATGTTTCAT------------TTTCACTTCTCGT-TATCATC----ATATATTAC

pNcHMA4-2-Ga CAACAATGTTTCAT------------TTTCACTTCTCGT-TATCATC----ATATATTAC

pNcHMA4-3-Ga CTTCCCTCTCTC-T------------TTTAATATCTCAC-CTTTATATATAATATTTTAT

pNcHMA4-1-CMA CTTCCCTCTCTC-T------------TTTAATATCTCAC-CTTTATATATAATATTTTAT

pNcHMA4-2-CMA CTTCCCTCTCTC-T------------TTTAATATCTCAC-CTTTATATATAATATTTTAT

pNcHMA4-1-SLM CTTCCCTCTCTC-T------------TTTAATATCTCAC-CTTTATATATAATATTTTAT

pNcHMA4-2-SLM ------TCTCTC-T------------TT--------------------------------

pNcHMA4-3-SLM ------TCTCTC-T------------TTTAATATCACAC-CTTTATATATAATATTTTAT

pNcHMA4-4-SLM CTTCCCTCTCTC-T------------TTTAATATCTCAC-CTTTATATATAATATTTTAT

pAhHMA4-1 ------AATCCGGAAA--------CAGTACAATTTTTAGG---TTTAATAAAGGTTTCAA

pAhHMA4-2 ----GGTGGTGCACAAAAGAAGAGTATTTAATAGGTAGTGCATCATATTCGTTATGCAAC

pAhHMA4-3 ----AGTGGTGCACAAAAGAAGAGTATTTAATAGGTAGTGCATCATATTCGTTATGCAAC

pAtHMA4 AACTT--TGATTTCGTGTCTCTTCC-TCACGAGATAAAATCCCCCTTTGACCATTTGTTT

pNcHMA4-1-LC AATT---CGAA--AATGTT------AAA---GAACAAAA-ACCAATC-----ATCTAA--

pNcHMA4-2-LC AATTT--CGAA--AATGTT------AAA---GAACAAAA-ACCAATC-----ATCTAAGA

pNcHMA4-3-LC AATTT--CGAA--AATGTT------AAA---GAACAAAA-ACCAATC-----ATCTAAGA

pNcHMA4-1-Ga AATTTTCTACC--CATGTT-------AACCCTATTAAAACACCAATT-----GGATAC--

pNcHMA4-2-Ga AATTTTCTACC--CATGTT-------AACCCTATTAAAACACCAATT-----GGATAC--

pNcHMA4-3-Ga AACAT--TAAT--AATTTTTTTAATAAATCCTAATAGGG-GT--ATG-----ATATAAA-

pNcHMA4-1-CMA AACAT--TAAT--AATTTTTTTAATAAATCCTAATAGGG-GT--ATG-----ATATAAA-

pNcHMA4-2-CMA AACAT--TAAT--AATTTTTTTAATAAATCCTAATAGGG-GT--ATG-----ATATAAA-

pNcHMA4-1-SLM AACAT--TAAT--AATTTTTTTAATAAATCCTAATAGGG-GT--ATG-----ATATATAA

pNcHMA4-2-SLM -------CGAA--AATGTT------AAA---GAACAAAA-ACCAATC-----ATCTAA--

pNcHMA4-3-SLM AACAT--AAAT--AA----------AAATTTTAATAAAA-AG--CTG-----ATAATT--

pNcHMA4-4-SLM AACAT--TAAT--AATTTTTT-AATAAATCCTAATAGGG-GT--ATG-----ATATATAA

pAhHMA4-1 AAATT--TAGT--AATGATTCTTAAATCATTTGGTA----CCCCCTT-----ATTAAACT

pAhHMA4-2 TATATT-TTAT--AATTTTT-------ATT-TGACAGAAAACATATT-----CCATAT--

pAhHMA4-3 TATATT-TTAT--AATTTTT-------ATT-TGACAGAAAACATATT-----CCATAT--

pAtHMA4 C----------------------------------GTCCAATCA----------------

pNcHMA4-1-LC ------------------------------------------------------------

pNcHMA4-2-LC GCATCCGCATCGCTCATCCCCACTGAGGTTCTGTTATTTAAAAGAAAAGAAAAAAATTAA

pNcHMA4-3-LC GCATCCGCATCGCTCATCCCCACTGAGGTTCTGTTATTTAAAAGAAAAGAAAAAAATTAA

pNcHMA4-1-Ga ------------------------------------------------------------

pNcHMA4-2-Ga ------------------------------------------------------------

pNcHMA4-3-Ga ---------------------------------TGGTCAAAGAAGGTGGAAGAAGCGTGA

pNcHMA4-1-CMA ---------------------------------TGGTCAAAGAAGGTGGAAGAAGCGTGA

pNcHMA4-2-CMA ---------------------------------TGGTCAAAGAAGGTGGAAGAAGCGTGA

pNcHMA4-1-SLM TTATA--------------------------TATGGTCAAAGAAGGTGGAAGAAGCGTGA

pNcHMA4-2-SLM ------------------------------------------------------------

pNcHMA4-3-SLM ------------------------------------TCGAAAA-----------------

pNcHMA4-4-SLM TTATA--------------------------TATGGTCAAAGAAGGTGGAAGAAGCGTGA

pAhHMA4-1 ------------------------------------TTCAACAA----------------

pAhHMA4-2 -------------------------------------TAGACATTA--------------

pAhHMA4-3 -------------------------------------TAGATA-----------------

pAtHMA4 ----------------ACTTAAA-ACCTAGCAAGAAC---TATTAATCATAAT-------

pNcHMA4-1-LC ------------------------------------------------------------

pNcHMA4-2-LC ATAATAATGAGCTGGCATAGAAAAATTAAACAATCGATTCTCTAGGAAGCATTTTTGGAG

pNcHMA4-3-LC ATAATAATGAGCTGGCATAGAAAAATTAAACAATCGATTCTCTAGGAAGCATTTTTGGAG

pNcHMA4-1-Ga ------------------ATGAAAACAATTCAATCCC--------ACACCAA--------

pNcHMA4-2-Ga ------------------ATGAAAACAATTCAATCCC--------ACACCAA--------

pNcHMA4-3-Ga GGATTACTG------TGGAGGAAAAAGAGACATTGGA-------GAAAGCAA--------

pNcHMA4-1-CMA GGATTACTG------TGGAGGAAAAAGAGACATTGGA-------GAAAGCAA--------

pNcHMA4-2-CMA GGATTACTG------TGGAGGAAAAAGAGACATTGGA-------GAAAGCAA--------

pNcHMA4-1-SLM GGATTACTG------TGGAGGAAAAAGAGACATTGGA-------GAAAGCAA--------

pNcHMA4-2-SLM ------------------------------------------------------------

pNcHMA4-3-SLM -------TG------TTAAAGAACAACAACCAATC------------ATCAT--------

pNcHMA4-4-SLM GGATTACTG------TGGAGGAAAAAGAGACATTGGA-------GAAAGCAA-------- pAhHMA4-1 ----------------CCTTATACACCTAACAA--------ATTTGTGGTATT-------

pAhHMA4-2 ---------------TAGTTACAAAATAATCAAATAAATATCT--AAAATATT-------

pAhHMA4-3 --------------------GCAAACCCATCACGT--------------TATC-------

pAtHMA4 ---------------------AT-A-----------------------------------

pNcHMA4-1-L ------------------------------------------------------------

pNcHMA4-2-LC TCGAGTCTGCTATAGGGTTCTGTCACGTGTCACGCCACAATTGAATCCTTATTTTTTT-A

pNcHMA4-3-LC TCGAGTCTGCTATAGG-TTCTGTCACGTGTCACGCCACAATTGAATCCTTATTTTTTTTA

pNcHMA4-1-Ga ---------------------ACCA-----------------------------------

pNcHMA4-2-Ga ---------------------ACCA-----------------------------------

pNcHMA4-3-Ga -------------------CGGTCA-----------------------------------

pNcHMA4-1-CMA -------------------CGGTCA-----------------------------------

pNcHMA4-2-CMA -------------------CGGTCA-----------------------------------

pNcHMA4-1-SLM -------------------CGGTCA-----------------------------------

pNcHMA4-2-SL ------------------------------------------------------------

pNcHMA4-3-SL -------------------CTATCA-----------------------------------

pNcHMA4-4-SL -------------------CGGTCA-----------------------------------

pAhHMA4-1 ---------------------TTTA-----------------------------------

pAhHMA4-2 ----------------------CCAT----------------------------------

pAhHMA4-3 ----------------------CCG-----------------------------------

pAtHMA4 ------------------------------------------------------------

pNcHMA4-1-LC ------------------------------------------------------------

pNcHMA4-2-LC TTTTTATTTTTTACCTCGAAAATCATTTTTATTAATAAAAAAAA-TTAAAGAGCTCGCGT

pNcHMA4-3-LC TTTTTATTTTTTACCTCGAAAATCATTTTTATTAATAAAAAAAAATTAAAGAGCTCGCGT

pNcHMA4-1-Ga ------------------------------------------------------------

pNcHMA4-2-Ga ------------------------------------------------------------

pNcHMA4-3-Ga ------------------------------------------------------------

pNcHMA4-1-CMA ------------------------------------------------------------

pNcHMA4-2-CMA ------------------------------------------------------------

pNcHMA4-1-SLM ------------------------------------------------------------

pNcHMA4-2-SLM ------------------------------------------------------------

pNcHMA4-3-SLM ------------------------------------------------------------

pNcHMA4-4-SLM ------------------------------------------------------------

pAhHMA4-1 ------------------------------------------------------------

pAhHMA4-2 ------------------------------------------------------------

pAhHMA4-3 ------------------------------------------------------------

pAtHMA4 --------------------------ATTATTCAGATTCTTA----GATATGCTATATAC

pNcHMA4-1-LC ----------------------------CATTCTGA----------CTTAT--------C

pNcHMA4-2-LC GTGGGGATGAGCGATGCGGATGCTCTAACATTCTGA----------CTTAT--------C

pNcHMA4-3-LC GTGGGGATGAGCGATGCGGATGCTCTAACATTCTGA----------CTTAT--------C

pNcHMA4-1-Ga -----------------------GAAATCGTTTTAA----------TTTCTAGCTTCAGT

pNcHMA4-2-Ga -----------------------GAAATCGTTTTAA----------TTTCTAGCTTCAGT

pNcHMA4-3-Ga -----------------------AC-ATTACTGTCA----------GATGT--------C

pNcHMA4-1-CMA -----------------------AC-ATTACTGTCA----------GATGT--------C

pNcHMA4-2-CMA -----------------------AC-ATTACTGTCA----------GATGT--------C

pNcHMA4-1-SLM -----------------------AC-ATTACTGTCA----------GATGT--------C

pNcHMA4-2-SLM ----------------------------CATTCTGA----------CTTAT--------C

pNcHMA4-3-SLM -----------------------TCTAACATTCTCA----------CTTAT--------C

pNcHMA4-4-SLM -----------------------AC-ATTACTGTCA----------GATGT--------C

pAhHMA4-1 --------------------------ATAATTTTGAATTTT-------TGTGGTA-GTTT

pAhHMA4-2 ---------------------------CCGTTTCAAAATATAAGACGTTTTAG-----CT

pAhHMA4-3 ---------------------------CAATTTT------------GTTTT-------TT

pAtHMA4 TAATGTGCCTAAACTC---------GTGACGTATCGGACCAGTTTTAGTCCCGCCCTACT

pNcHMA4-1-LC TACAGGG-GCGGCTTATTAGA--GTGGGGT-CAATAGAT--GCTCT-GCACTAGGTGACG

pNcHMA4-2-LC TACAGGG-GCGGCTTATTAGA--GTGGGGT-CAATAGAT--GCTCT-GCACTAGGTGACG

pNcHMA4-3-LC TACAGGG-GCGGCTTATTAGA--GTGGGGT-CAATAGAT--GCTCT-GCACTAGGTGACG

pNcHMA4-1-Ga TTCAGAAACCAATTTAC-----------GC-CAA-AAAC--GTTGT-TTACTGTGAAACA

pNcHMA4-2-Ga TTCAGAAACCAATTTAC-----------GC-CAA-AAAC--GTTGT-TTACTGTGAAACA

pNcHMA4-3-Ga GAAGGAGAGAAAGTGA---------GAGAG-TGTGAGAC--TCTGA-GAGAGAGA-GAAG

pNcHMA4-1-CMA GAAGGAGAGAAAGTGA---------GAGAG-TGTGAGAC--TCTGA-GAGAGAGA-GAAG

pNcHMA4-2-CMA GAAGGAGAGAAAGTGA---------GAGAG-TGTGAGAC--TCTGA-GAGAGAGA-GAAG

pNcHMA4-1-SLM GAAGGAGAGAAAGTGA---------GAGAG-TGTGAGAC--TCTGA-GAGAGAGA-GAAG

pNcHMA4-2-SLM TACAGGG-GCGGCTTATTCGA--GTGGGGT-CAATAGAT--GCTCT-GCACTAGGTGACG

pNcHMA4-3-SLM TGCAGAG-GCGGCTTA---------GAGGT-CAATAGGT--GCTCT-GCACTAGG-GACC

pNcHMA4-4-SLM GAAGGAGAGAAAGTGA---------GAGAG-TGTGAGAC--TCTGA-GAGAGAGA-GAAG

pAhHMA4-1 TGAAGAACATGATTTA---------AGAAT---TTGGA---GCTTT---TTCGAATTACC

pAhHMA4-2 AAAAACACGCCGATTAAGAAGATTTACTTT-TTAAAGGT--TCGGT-TAATCATAAAAGA

pAhHMA4-3 GGTCATACGTTATTC-------CTCACTAT-CTA---GT--TC--T-TACTTTT------

pAtHMA4 TTCAAACTAATAATGTTT-------TGTAA--TCTCAATACTTTTCCTTCAAAACAATTT

pNcHMA4-1-LC T--AGAAAAACAAAATTT----TAGTACAG-AAAAAAGAATACAA--ACAAA---AATT-

pNcHMA4-2-LC T--AGAAAAACAAAATTT----TAGTACAG-AAAAAAGAATACAA--ACAAA---AATT-

pNcHMA4-3-LC T--AGAAAAACAAAATTT----TAGTACAG-AAAAAAGAATACAA--ACAAA---AATT-

pNcHMA4-1-Ga CACACCAAACCAAAAATCAC--CATCACAA-AAATAG--CCAAATTCATCCAT--AATTG

pNcHMA4-2-Ga CACACCAAACCAAAAATCAC--CATCACAA-AAATAG--CCAAATTCATCCAT--AATTG

pNcHMA4-3-Ga TCAAGAAGGAGAAGA--------AG-ACAA-AAGCTAATTTAAAGCTACGAAT--AATTA

pNcHMA4-1-CMA TCAAGAAGGAGAAGA--------AG-ACAA-AAGCTAATTTAAAGCTACGAAT--AATTA

pNcHMA4-2-CMA TCAAGAAGGAGAAGA--------AG-ACAA-AAGCTAATTTAAAGCTACGAAT--AATTA

pNcHMA4-1-SLM TCAAGAAGGAGAAGA--------AG-ACAA-AAGCTAATTTAAAGCTACGAAT--AATT-

pNcHMA4-2-SLM T--AGAAAAACAAAATTT----TAGTA---------------------------------

pNcHMA4-3-SLM TAAGGAAAAACAAAATTT----TAGTATAG-AAAAAAATATGGAG--ACAAA---AATT-

pNcHMA4-4-SLM TCAAGAAGGAGAAGA--------AG-ACAA-AAGCTAATTTAAAGCTACGAAT--AATT-

pAhHMA4-1 CT-------ATAATATTT-------TATAG--T-TCATTA----TCCCT--AAATATCTT

pAhHMA4-2 GACTGCATAATAGAAATAATTATAAAATATTAAAATAATATATAATTTTCATATAAATTT

pAhHMA4-3 --CTACATAATA----------TCTAATA-------AATATCTAGTTTT--------TTT

pAtHMA4 ------------TA----AGAAAACTGC-----CGTATTT----------GTTATG----

pNcHMA4-1-LC ---AG-------TAGAAAAAAAA-----------GTACA-----------CTTAA-----

pNcHMA4-2-LC ---AG-------TAGAAAAAAAA-----------GTACA-----------CTTAA----- pNcHMA4-3-LC ---AG-------TAGAAAAAAAA-----------GTACA-----------CTTAA-----

pNcHMA4-1-Ga TATAAGT-----AACCGTACAAACG--------TATATAA-------CAAAGCGT-----

pNcHMA4-2-Ga TATAAGT-----AACCGTACAAACG--------TATATAA-------CAAAGCGT-----

pNcHMA4-3-Ga TATAA-------TATGGCGAAGACGAGACGGGACATATATTCACCCTCGCTTTTC-----

pNcHMA4-1-CMA TATAA-------TATGGCGAAGACGAGACGGGACATATATTCACCCTCGCTTTTC-----

pNcHMA4-2-CMA TATAA-------TATGGCGAAGACGAGACGGGACATATATTCACCCTCGCTTTTC-----

pNcHMA4-1-SLM --TAA-------AGCTACGAAGACGAGACGGGACATATATTCACCCTCGCTTTTC-----

pNcHMA4-2-SLM ---------------GAAAAAAA-----------GTTCA-----------CTTAA-----

pNcHMA4-3-SLM ---AG-------TATAGAAAAAA---------ATATAGA---ATCTTTGATTAAG-----

pNcHMA4-4-SLM --TAA-------AGCTACGAAGACGAGACGGGACATATATTCACCCTCGCTTTTC-----

pAhHMA4-1 ------------TACAAAAGAAATCTG-------ATAAT-----------ATCAAA----

pAhHMA4-2 GAAAACGACTTATATTGTAAAAAAAAAAAAGTTTCTCCAAAACGCTTTATATTTTGAATC

pAhHMA4-3 --------------------------------TTTTCC-----------TATTT------

pAtHMA4 -----------------------------A---GCTAATTATAATCGAGCAT--------

pNcHMA4-1-LC -----------------------------AAATA-TAGTTTTAATTTTGCAC--------

pNcHMA4-2-LC -----------------------------AAATA-TAGTTTTAATTTTGCAC--------

pNcHMA4-3-LC -----------------------------AAATA-TAGTTTTAATTTTGCAC--------

pNcHMA4-1-Ga -----------------------------ACACA-TGTTTTCAATTGTCCA---------

pNcHMA4-2-Ga -----------------------------ACACA-TGTTTTCAATTGTCCA---------

pNcHMA4-3-Ga -----------------------------ACATA-TATTTTCGGTATTGCCA--------

pNcHMA4-1-CMA -----------------------------ACATA-TATTTTCGGTATTGCCA--------

pNcHMA4-2-CM -----------------------------ACATA-TATTTTCGGTATTGCCA--------

pNcHMA4-1-SLM -----------------------------ACATA-TATTTTCGGTATTGCCA--------

pNcHMA4-2-SLM -----------------------------G------------------------------

pNcHMA4-3-SLM -----------------------------AT-TA-TAGTTTTGATTTTGCAA--------

pNcHMA4-4-SLM -----------------------------ACATA-TATTTTCGGTATTGCCA--------

pAhHMA4-1 -----------------------------AAATGTTAAAGAAAACCAACCAA--------

pAhHMA4-2 AGAGGAAGTAATAATTAAGAAAATTCACTATTTC-CCTGTTTATAATAGCAAATCACGTT

pAhHMA4-3 -----------------------------ATCTG-CCTATTTATAT--GCAA--------

pAtHMA4 ----------------CCTACCTTGA-----------------ATTAACTTGGTTTC---

pNcHMA4-1-LC ------------TTACGTATACTTAT-----------------AAAATTTTTGACCC---

pNcHMA4-2-LC ------------TTACGTATACTTAT-----------------AAAATTTTTGACCC---

pNcHMA4-3-LC ------------TTACGTATACTTAT-----------------AAAATTTTTGACCC---

pNcHMA4-1-Ga -----------------AAAACACATTTTACTACAATTAAACGAAGCTATCAGATCC---

pNcHMA4-2-Ga -----------------AAAACACATTTTACTACAATTAAACGAAGCTATCAGATCC---

pNcHMA4-3-Ga ------------CTCTCAAATTTTATTTTTTTCCTTTTTTCTTGTCTTTTTTGACCC---

pNcHMA4-1-CMA ------------CTCTCAAATTTTATTTTTTTCCTTTTTTCTTGTCTTTTTTGACCC---

pNcHMA4-2-CMA ------------CTCTCAAATTTTATTTTTTTCCTTTTTTCTTGTCTTTTTTGACCC---

pNcHMA4-1-SLM ------------CTCTCAAATTTTATTTTTTCCCTTTTTTCTTGTCTTTTTTGACCC---

pNcHMA4-2-SLM ------------------------------------------------------------

pNcHMA4-3-SLM ------------TT---AAGACTTAT-----------------AAAATCTTTGACCC---

pNcHMA4-4-SLM ------------CTCTCAAATTTTATTTTTTCCCTTTTTTCTTGTCTTTTTTGACCC---

pAhHMA4-1 ----------------TC-ATCTTGC-----------------AT-ATCTAACATTC---

pAhHMA4-2 ATCCCACACTATCTTTTTTTTTTTTTTTGTCATACGTTATCCCGCACTATCTAGTTCTTA

pAhHMA4-3 ----------------------------------------------CTACCAAGTTT---

pAtHMA4 ---------AAGACTAATTAAGATCGAC--------TTTTCCA--AAATAAAGTCATGCA

pNcHMA4-1-LC ---------GGCCCTGCTTA---TCCGG--------CTATATA-AAGCAACTACCATTTC

pNcHMA4-2-LC ---------GGCCCTGCTTA---TCCGG--------CTATATA-AAGCAACTACCATTTC

pNcHMA4-3-LC ---------GGCCCTGCTTA---TCCGG--------CTATATA-AAGCAACTACCATTTC

pNcHMA4-1-Ga ---------A--TGTCCTCT---CCCAG--------AAA-AGA-AAATAAACACT-CGTC

pNcHMA4-2-Ga ---------A--TGTCCTCT---CCCAG--------AAA-AGA-AAATAAACACT-CGTC

pNcHMA4-3-Ga ---------GGCCCTGCTTA---TTTGG--------CTATATA-AAGCAACTACCTTATC

pNcHMA4-1-CMA ---------GGCCCTGCTTA---TTTGG--------CTATATA-A-GCAACTACCTTATC

pNcHMA4-2-CMA ---------GGCCCTGCTTA---TTTGG--------CTATATA-AAGCAACTACCTTATC

pNcHMA4-1-SLM ---------GGCCCTGCTTA---TTTGG--------CTATATA-A-GCAACTACCTTATC

pNcHMA4-2-SLM ------------------------CCGG--------CTATATA-AAGCAACTACCATTTC

pNcHMA4-3-SLM ---------GGCCCTGCTTA---TCCGG--------CTATATA-A-GCAACTACCATTTC

pNcHMA4-4-SLM ---------GGCCCTGCTTA---TTTGG--------CTATATA-A-GCAACTACCTTATC

pAhHMA4-1 -------------TTACTTA---TCTGC--------CTATATA--AGCAACTCCCATTTC

pAhHMA4-2 CTTATCTGCATTTTTTTTTT---CCTATTTATCTGCCTATTTATAAGCAACTACCAAGTT

pAhHMA4-3 ---------AGATCTCTTTA---CCTAA------GATTGTCAAGAAAAAAA---------

pAtHMA4 TAAA---AA---TTGATT--TAAATA-TTATACATACGAGTTATGACAATTTGTATATAA

pNcHMA4-1-LC TAGATATCT---TCACCTCACAATCT-TCCTCTCTACGTTCTAAAAC--CTCTCTCACTC

pNcHMA4-2-LC TAGATATCT---TCACCTCACAATCT-TCCTCTCTACGTTCTAAAAC--CTCTCTCACTC

pNcHMA4-3-LC TAGATATCT---TCACCTCACAATCT-TCCTCTCTACGTTCTAAAAC--CTCTCTCACTC

pNcHMA4-1-Ga AAGATAACCATATTATTTATGAATAAGTAAAATTCATGCGATGCAAT--TTCCTCCAGCC

pNcHMA4-2-Ga AAGATAACCATATTATTTATGAATAAGTAAAATTCATGCGATGCAAT--TTCCTCCAGCC

pNcHMA4-3-Ga TAGATATCT---TCACCTCGCAATCT-TCCTCTCTACGTTCCAAAAC--CTCTCTCACTC

pNcHMA4-1-CMA TAGATATCT---TCACCTCGCAATCT-TCCTCTCTACGTTCCAAAAC--CTCTCTCACTC

pNcHMA4-2-CMA TAGATATCT---TCACCTCGCAATCT-TCCTCTCTACGTTCCAAAAC--CTCTCTCACTC

pNcHMA4-1-SLM TAGATATCT---TCACCTCGCAATCT-TCCTCTCTACGTTCCAAAAC--CTCTCTCACTC

pNcHMA4-2-SLM TAGATATCT---TCACCTCACAATCT-TCCTCTCTACGTTCTAAAAC--CTCTCTCACTC

pNcHMA4-3-SLM TAGATATCT---TCACCTCACAATCT-TCCTCTCTTCGTTCCAAAAC--CTCTCTCACTC

pNcHMA4-4-SLM TAGATATCT---TCACCTCGCAATCT-TCCTCTCTACGTTCCAAAAC--CTCTCTCACTC

pAhHMA4-1 TTGACCTAA---TCCCCT--CAATCT-TCTTCTCTACGT-TCCTAACACTTCTCTCACTC

pAhHMA4-2 TAGATCTCT---TTACCT--CAATCT-TCTTCTCAACGTTCTAAAACACTTCTCTCACTG

pAhHMA4-3 -AGATCTCT---TTACCT--CAATCT-TCTTCTCAAAGTTCTAAAACACTTCTCTCACTG

pAtHMA4 AAAAATTTGCT-----------GGACGTTTG-AGTAAAAC----GTT--TCATAAAATAT

pNcHMA4-1-LC ----TCAGTCT-----------TCACCTTTGTGGTAATACTTTAATC--TGATCGAACCG

pNcHMA4-2-LC ----TCAA------------------------------ACTTTAATC--TGATCGAACCG

pNcHMA4-3-LC ----TCAA------------------------------ACTTTAATC--TGATCGAACCG

pNcHMA4-1-Ga GTA-TCAGTCT------------CTACGTGGTGCGGTCGCCAGAGTT--AAATTAAAAGA

pNcHMA4-2-Ga GTA-TCAGTCT------------CTACGTGGTGCGGTCGCCAGAGTT--AAATTAAAAGA

pNcHMA4-3-Ga ----TCTGTCT-----------TCACCTTTGTGGTAATACTT-AATCTCTGATCGAACCG

pNcHMA4-1-CMA ----TCTGTCT-----------TCACCTTTGTGTTAATACTTTAATCTCTGATCGAACCG

pNcHMA4-2-CMA ----TCTGTCTACTCTCTGTCTTCACCTTTGTGGTAATACTTTAATCTCTGATCGAACCG

pNcHMA4-1-SLM ----TCTGTCT-----------TCACCTTTGTGGTAATACTTTAATCTCTGATCGAACCG

pNcHMA4-2-SLM ----TCAGTCT-----------TCACCTTTGTGGTAATACTTTAATC--TGATCGAACCG

pNcHMA4-3-SLM ----TCAGTCT-----------TCACCTTTGTGGTAATACTTTAATC--TGGTCGAACCG

pNcHMA4-4-SLM ----TCTGTCT-----------TCACCTTTGTGGTAATACTTTAATCTCTGATCGAACCG

pAhHMA4-1 ----CCTCTCT-----------CAACCTTTATGGTAACACCT-AATC--TGATCGAACCA

pAhHMA4-2 ----CCTCTCT-----------CAACCTTTATGGTAACACCT-AATC--TGATTGAACCA

pAhHMA4-3 ----CCTCTCT-----------CAACCTTTATGGTAACACCT-AATC--TGATTGAACCA

pAtHMA4 AG--GAACTGAATC---TTGAAGATAGGAACCGGAAACA----AATAACTAAAAAGATAG

pNcHMA4-1-LC CACCAAACCG-----GTCCGGTCTTTCTTCTCGGCCTC---------------------G

pNcHMA4-2-LC CACCAAACCG-----GTCCGGTCTTTCTTCTCGGCCTC---------------------G

pNcHMA4-3-LC CACCAAACCG-----GTCCGGTCTTTCTTCTCGGCCTC---------------------G

pNcHMA4-1-Ga CACCAA-CCA-----AAATAATCTGATTTTTCACCCCT---------------------T

pNcHMA4-2-Ga CACCAA-CCA-----AAATAATCTGATTTTTCACCCCT---------------------T

pNcHMA4-3-Ga CACCAAACCA-----GTCCGGTCTTTCTTCTCGGCCTC---------------------G

pNcHMA4-1-CMA CACCAAACCA-----GTCCGGTCTTTCTTCTCGGCCTC---------------------G

pNcHMA4-2-CMA CACCAAACCA-----GTCCGGTCTTTCTTCTCGGCCTC---------------------G

pNcHMA4-1-SLM CACCAAACAA-----GTCCGGTCTTTCTTCTCGGCCTC---------------------G

pNcHMA4-2-SLM CACCAAACAA-----GTCCGGTCTTTCTTCTCGGCCTC---------------------G

pNcHMA4-3-SLM CACCAAACCG-----GTCCGGTCTTTCTTCTCGGCCTC---------------------G

pNcHMA4-4-SLM CACCAAACCA-----GTCCGGTCTTTCTTCTCGGCCTC---------------------G

pAhHMA4-1 AACCGAACCGAACCAAATCGGTTTTTCGTATCGGCTTC------TTCCTTTTGCTACTAG

pAhHMA4-2 AACCAAACCAAACTGGTCCGGTCTTTCTTCTCGGCTTCTTCCCATTGCTACTAGCTTGCG

pAhHMA4-3 AACCAAACCAAACTGGTCCGGTCTTTCTTCTCGGCTTCTTCCCATTGCTACTAGCTTGCG

pAtHMA4 CCTACGAGATTTACGTGGTAAAAAAATAACTTAAAACCCCATAAA--ACC--------TT

pNcHMA4-1-LC TCT------TTTCTCCGGTAT----TCT----TTCTCTTCTTAATTCACA--------TA

pNcHMA4-2-LC TCT------TTTCTCCGGTAT----TCT----TTCTCTTCTTAATTCACA--------TA

pNcHMA4-3-LC TCT------TTTCTCCGGTAT----TCT----TTCTCTTCTTAATTCACA--------TA

pNcHMA4-1-Ga TTT------CATTAAAGGTAA----AATGGAACTCTCAACACACTATAAG--------CA

pNcHMA4-2-Ga TTT------CATTAAAGGTAA----AATGGAACTCTCAACACACTATAAG--------CA

pNcHMA4-3-Ga TCT------TTTCTCCGGTAT----TCT----TTCTCTTCTTAATTCACA--------TA

pNcHMA4-1-CMA TCT------TTTCTCCGGTAT----TCT----TTCTCTTCTTAATTCACA--------TA

pNcHMA4-2-CMA TCT------TTTCTCCGGTAT----TCT------CTCTTCTTAATTCACA--------TA

pNcHMA4-1-SLM TCT------TTTCTCCGGTAT----TCT----TTCTCTTCTTAATTCACA--------TA

pNcHMA4-2-SLM TCT------TTTCTCCGGTAT----TCT----TTCTCTTCTTAATTCACA--------TA

pNcHMA4-3-SLM TCT------TTTCTCCGGTAT----TCT----TTCTCTTCTTAATTCACA--------TA

pNcHMA4-4-SLM TCT------TTTCTCCGGTAT----TCT----TTCTCTTCTTAATTCACA--------TA

pAhHMA4-1 CTCTCCTCACTTCTCCGGTAT---TTTTTTTTCTCTCTTCTTAATTCACA--------TA

pAhHMA4-2 TCT------CTTCTCCGGTAT----TTG----TTCTCTTCTTAATTCACAATTTCACATA

pAhHMA4-3 TCT------CTTCTCCGGTAT----TTG----TTCTCTTCTTAATTCACAATTTCACATA

pAtHMA4 AATCAAAGAAAAAATG-GTTT----------AATAAAGGTTTCAAAAATTTAGTAATG--

pNcHMA4-1-LC AATTTCATAACAAGTG-ATTTCTT-CGTAAAAATTAAAATCCGATCAAATTCACGAT-AG

pNcHMA4-2-LC AATTTCATAACAAGTG-ATTTCTT-CGTAAAAATTAAAATCCGATCAAATTCACGAT-AG

pNcHMA4-3-LC AATTTCATAACAAGTG-ATTTCTT-CGTAAAAATTAAAATCCGATCAAATTCACGAT-AG

pNcHMA4-1-Ga AAAAAGAAAAAAAGTCCACCACTT---TCACAATTTCCCTCTTATAAAAGTCACTCTTAG

pNcHMA4-2-Ga AAAAAGAAAAAAAGTCCACCACTT---TCACAATTTCCCTCTTATAAAAGTCACTCTTAG

pNcHMA4-3-Ga GATTTCATAACAAGTG-ATTTTTT-CGTAATAATTAAAATCCGATCAAATTCACGAT-AG

pNcHMA4-1-CMA GATTTCATAACAAGTG-ATTTTTTTCGTAATAATTAAAATCCGATCAAATTCACGAT-AG

pNcHMA4-2-CMA GATTTCATAACAAGTG-ATTTCTT-CGTAAAAATTAAAATCCGATCAAATTCACGAC-AG

pNcHMA4-1-SLM GATTTCATAACAAGTG-ATTTTTT-CGTAATAATTAAAATCCGATCAAATTCACGAT-AG

pNcHMA4-2-SLM GATTTCATAACAAGTG-ATTTCTT-CGTAATAATTAATATCCGATCAAATTCACGAT-AG

pNcHMA4-3-SLM GATTTCATAACAAGTG-ATTTCTT-CGTAAAAATTAAAATCCGATCAAATTCACGGT-AG

pNcHMA4-4-SLM GATTTCATAACAAGTG-ATTTTTT-CGTAATAATTAAAATCCGATCAAATTCACGAT-AG

pAhHMA4-1 GATTTCATGATAAGTG-ATCT----------AAAACAAGACACATAGATTTCATGATAAG

pAhHMA4-2 GATTTCATGACAAGTG-ATCT--------AAAACAAAA---CGCTC---TTCCTCGT---

pAhHMA4-3 GATTTCATGACAAGTG-ATCT--------AAAACAAAA---CGCTC---TTCCTCGT---

pAtHMA4 -GTTCTAAAATCATTTGGTACCCCCCTTATTAAACTTTCAACAACCTTATACACCTA---

pNcHMA4-1-LC TG----------ATATC-------------------TCCAACA-CGTTATATGCAT----

pNcHMA4-2-LC TG----------ATATC-------------------TCCTACA-CGTTATATGCAT----

pNcHMA4-3-LC TG----------ATATC-------------------TCCTACA-CGTTATATGCAT---- pNcHMA4-1-Ga TGT---------AAATGA-------------------CAAATG-ACTCCTGTTAGA----

pNcHMA4-2-Ga TGT---------AAATGA-------------------CAAATG-ACTCCTGTTAGA----

pNcHMA4-3-Ga TG----------ATATGATATATGCATA--TATGCATCCAACA-CGTTATATGCAT----

pNcHMA4-1-CMA TG----------ATATGATATATGCATA--TATGCATCCAACA-CGTTATATGCAT----

pNcHMA4-2-CMA TG----------ATATGATATATGCATA--TATGCATCCAACA-CGTTATATGCAT----

pNcHMA4-1-SLM TG----------ATATGATATATGCATA--TATGCATCCAACA-CGTTATATGCAT----

pNcHMA4-2-SLM TG----------ATATC-------------------TCCAACA-CGTTATATGCATGATG

pNcHMA4-3-SLM TG----------ATATC-------------------TCCAACA-CGTTATATGCAT----

pNcHMA4-4-SLM TG----------ATATGATATATGCATA--TATGCATCCAACA-CGTTATATGCAT----

pAhHMA4-1 TGATCTAAAACAAGACGCTATTCTTCTC-TTGCATTTCTTGTG-TTTTGTTTTTCTAGTA

pAhHMA4-2 TGC---------ATTTGT------------------TTTTATT-TTTTGTGTACGC----

pAhHMA4-3 TGC---------ATTTGT------------------TTTTATT-TTTTGTGTACGC----

pAtHMA4 ----CTAGATTTGTGGTAT--TTTTAATAATTTTGAATTTTTGTG-GTAGTTTTGGAAAA

pNcHMA4-1-LC ---CCCAGCATAAAAGTTTTGCTTTCTTAATTTT---TTT-CCC--------TTAAAAGA

pNcHMA4-2-LC ---CCCAGCATAAAAGTTTTGCTTTCTTAATTTT---TTT-CCC--------TTAAAAGA

pNcHMA4-3-LC ---CCCAGCATAAAAGTTT-GCTTTCTTAATTTT---TT--CCC--------TTAAAAGA

pNcHMA4-1-Ga ----ACTACCTAAAAGCA--GGTTATGGAATTCG---TTGGTCAA--ATGAATTAAAAAA

pNcHMA4-2-Ga ----ACTACCTAAAAGCA--GGTTATGGAATTCG---TTGGTCAA--ATGAATTAAAAAA

pNcHMA4-3-Ga ---CCCAGCATAAAAGTTTTGCTTTCTTAATTTT---TTTTCCC--------TTAAAAGA

pNcHMA4-1-CMA ---CCCAGCATAAAAGTTTTGCTTTCTTAATTTT---TTTTCCC--------TTAAAAGA

pNcHMA4-2-CMA ---CCCAGCATAAAAGTTTTGCTTTCTTAATTTT---TTTTCCC--------TTAAAAGA

pNcHMA4-1-SLM ---CCCAGCATAAAAGTTTTGCTTTCTTATTTTT---TTTTCCC--------TTAAAAGA

pNcHMA4-2-SLM CATCCCAGCATAAAAGTTTTGCTTTCTTAATTTT---TTTTCCC--------TTAAAAGA

pNcHMA4-3-SLM ---CCCAGCATAAAAGTTTTTCTTTCTTATTTTT---TTTCCCC--------TTAAAAGA

pNcHMA4-4-SLM ---CCCAGCATAACAGTTTTGCTTTCTTATTTTT---TTT-CCC--------TTAAAAGA

pAhHMA4-1 CG--CCAAATTCAT--CCC--TTCACATCATTTT---TTTATGCGTATAGAATCCAAAAA

pAhHMA4-2 -----CAAATTA--AGC----CCTTCAAAAATCA---TT---------------GAGAGA

pAhHMA4-3 -----CAAATTA--AGC----CCTTCAAAAATCA---TT---------------GAGAGA

pAtHMA4 CATGATTTAA---TAATTTGAAGCTTTTCGAATTACTCTA-TAATT--TTTAATAGAG-A

pNcHMA4-1-LC TT-GGAAA-A--GTAGCC---------------------ATTAATC-CCATAATA-----

pNcHMA4-2-LC TT-GGAAA-A--GTAGCC---------------------ATTAATC-CCATAATA-----

pNcHMA4-3-LC TT-GGAAA-A--GTAGCC---------------------ATTAATC-CCATAATA-----

pNcHMA4-1-Ga AAAAAAAAA---CAAATT---------------------ATAAATTGTTATGAAAGAA--

pNcHMA4-2-Ga AAAAAAAAAA--CAAATT---------------------ATAAATTGTTATGAAAGAA--

pNcHMA4-3-Ga TTTGGAAAA---TTAGCC---------------------ATTAATC-CCATAATA-----

pNcHMA4-1-CMA TTTGGAAAA---TTAGCC---------------------ATTAATC-CCATAATA-----

pNcHMA4-2-CMA TTTGGAAAA---TAAGCC---------------------ATTAATC-CCATAATA-----

pNcHMA4-1-SLM TTTGGAAAA---TTAGCC---------------------ATTAATC-CCATAATA-----

pNcHMA4-2-SLM TT-GGAAATG--GCTGCC---------------------ATTAATC-CCATAATA----- pNcHMA4-3-SLM TTTGGNAAAA--TTAACC---------------------ATTAATC-CCATAATA-----

pNcHMA4-4-SLM TTTGGAAAA---TTAGCC---------------------ATTAATC-CCATAATA-----

pAhHMA4-1 AATAAATAAAAGCTGATTCGTCTTCTTCCTCGTAACAGAAGTAAGC--CATGAGAGGGTG

pAhHMA4-2 TT-GAAGATCTTTTGGCT---------------------A--GATCTCAACTTTA-----

pAhHMA4-3 TT-GAAGATCTTTTGGCT---------------------A--GATCTCAACTTTA-----

pAtHMA4 TCGATCATTATCCCTAAATATATTTATAAAAAATCTGATAATTACTAAAATGTTAAAGAA

pNcHMA4-1-LC ---ATCTCT-TTT-----------------------------------------------

pNcHMA4-2-LC ---ATCTCT-TTT-----------------------------------------------

pNcHMA4-3-LC ---ATCTCT-TTTG-CGATGTG------ATTTATTTTTTTCT-TTTTAGATTTCCGTTTC

pNcHMA4-1-Ga AATATCTCG-TACACAAGTGTC------ACCAAGTATAAGGGACCC---ATCTTTGTTGA

pNcHMA4-2-Ga AATATCTCG-TACACAAGTGTC------ACCAAGTATAAGGGACCC---ATCTTTGTTGA

pNcHMA4-3-Ga ---ATCTCT-TTTTGCGATGTG------ATTTGTTTTTTTCT-TTTTAGATTTCCGTTTC

pNcHMA4-1-CMA ---ATCTCT-TTTTGCGATGTG------ATTTGTTTTTTTCT-TTTTAGATTTCCGTTTC

pNcHMA4-2-CMA ---ATCTCT-TTTTGCGATGTG------ATTTGTTTTTTTCTGTTTTAGATTTCCGTTTC

pNcHMA4-1-SLM ---ATCTCT-TTTTGCGATGTG------ATTTGTTTTTTTCTGTTTTAGATTTCCGTTTC

pNcHMA4-2-SLM ---ATCTCT-TTT-----------------------------------------------

pNcHMA4-3-SLM ---ATCTCT-TTTTGCGATGTG------ATTTGTTTTTTTCT-TTTTAGATTTCCGTTTC

pNcHMA4-4-SLM ---ATCTCT-TTTTGCGATGTG------ATTTGTTTTTTTCTGTTTTAGATTTCCGTTTC

pAhHMA4-1 AAGATTTTTCCCTTTAGAAAAA---AAAAAGAGAGTGTAGATTTTTTTGGC-TTGATCTC

pAhHMA4-2 --TATTTGTATATATATATATA------TATCTATTTTTTTTTTTT----------TTCT

pAhHMA4-3 --TATTTGTATATATATATAT------------------------T----------TTCT

pAtHMA4 AACT----------AACCAATCATCTTGCATCTAA-CATTCTTACTTATCTGCCTA----

pNcHMA4-1-LC ------------------------------------------------------------

pNcHMA4-2-LC ------------------------------------------------------------

pNcHMA4-3-LC ACA-----------GATTCGCCATTAATCCCATAATAATCTTGATTTGTTTTTTT-----

pNcHMA4-1-Ga AAG-----------AAGATGAAGTTAA--CAAAAAAACTTTTGCCTTCTCTCTC------

pNcHMA4-2-Ga AAG-----------AAGATGAAGTTAA--CAAAAAAACTTTTGCCTTCTCTCTC------

pNcHMA4-3-Ga ACA-----------GATTCGCCATTAATCCCCTAATAATCTCGGTTTGTTTTTTT-----

pNcHMA4-1-CMA ACA-----------GATTCGCCATTAATCCCCTAATAATCTCGGTTTGTTTTTTT-----

pNcHMA4-2-CMA ACA-----------GATTCGCTATTAATCCCATAATAATCTCGATTTGTTTTTTTTGTTT

pNcHMA4-1-SLM ACA-----------GATTCGCCATTAATCCCATAATAATCTCGATTTGTTTTTT------

pNcHMA4-2-SLM ------------------------------------------------------------

pNcHMA4-3-SLM ACA-----------GATTCGCCATTAATCCCATAATAATCTCGGTTTGTTTTTTT-----

pNcHMA4-4-SLM ACA-----------GATTCGCCATTAATCCCATAATAATCTCGATTTGTTTTTT------

pAhHMA4-1 AACTTTATTTTTGTAACC--TTATCTAAAAAGTAATCATTTTTA---ATAAGAAAA----

pAhHMA4-2 AAA-----------AAGTTAACATT-------------TCCAAAGTTTTTTCTTAA----

pAhHMA4-3 AAA-----------AAGTTAACATT-------------TCCAAAGTTTTTTCTTAA----

pAtHMA4 ------------------------------------------------------------

pNcHMA4-1-LC ------------------------------------------------------------

pNcHMA4-2-LC ------------------------------------------------------------

pNcHMA4-3-LC ------------------------------------------------------------

pNcHMA4-1-Ga ------------------------------------------------------------

pNcHMA4-2-Ga ------------------------------------------------------------

pNcHMA4-3-Ga ------------------------------------------------------------

pNcHMA4-1-CMA ------------------------------------------------------------

pNcHMA4-2-CMA TTTTTTAGATTTCCGTTTCACAGATTTGCCATTAATCCAATAATAATCTCTTTTTGCGAT

pNcHMA4-1-SLM ------------------------------------------------------------

pNcHMA4-2-SLM ------------------------------------------------------------

pNcHMA4-3-SLM ------------------------------------------------------------

pNcHMA4-4-SLM ------------------------------------------------------------

pAhHMA4-1 ------------------------------------------------------------

pAhHMA4-2 ------------------------------------------------------------

pAhHMA4-3 ------------------------------------------------------------

pAtHMA4 ------------------------------------------------------------

pNcHMA4-1-LC ------------------------------------------------------------

pNcHMA4-2-LC ------------------------------------------------------------

pNcHMA4-3-LC ------------------------------------------------------------

pNcHMA4-1-Ga ------------------------------------------------------------

pNcHMA4-2-Ga ------------------------------------------------------------

pNcHMA4-3-Ga ------------------------------------------------------------

pNcHMA4-1-CMA ------------------------------------------------------------

pNcHMA4-2-CMA GTGATTTTGTTTTTTTCTTTTTAGATTTCCGTTTCACAGATTCGCTATTAATCCCATAAT

pNcHMA4-1-SLM ------------------------------------------------------------

pNcHMA4-2-SLM ------------------------------------------------------------

pNcHMA4-3-SLM ------------------------------------------------------------

pNcHMA4-4-SLM ------------------------------------------------------------

pAhHMA4-1 ------------------------------------------------------------

pAhHMA4-2 ------------------------------------------------------------

pAhHMA4-3 ------------------------------------------------------------

pAtHMA4 --------------TATAAGCAACAACTCCCATTTCTTGACTTCTCTAT------GTTCC

pNcHMA4-1-LC ------------------------------------------------------------

pNcHMA4-2-LC ------------------------------------------------------------

pNcHMA4-3-LC ------------------------ATTTTTAGATTTCCGTTTCACAGAT--------TCG

pNcHMA4-1-Ga ---------------------------------TCTCCATCGAATA-----------CTA

pNcHMA4-2-Ga ---------------------------------TCTCCATCGAATA-----------CTA

pNcHMA4-3-Ga ------------------------ATTTTTAGATCTCCGTTTCACAGAT--------TCG

pNcHMA4-1-CMA ------------------------ATTTTTAGATCTCCGTTTCACAGAT--------TCG

pNcHMA4-2-CMA AATCTCGATTTGTTTTTTATTTTTATTTTTAGATTTCCGTTTCACAGAT--------TCG

pNcHMA4-1-SLM ------------------------ATTTTTAGATTTCCGTTTCACAGAT--------TCG

pNcHMA4-2-SLM ------------------------------------------------------------

pNcHMA4-3-SLM ------------------------ATTTTTAGATTTCCGTTTCACAGAT--------TCG

pNcHMA4-4-SLM ------------------------ATTTTTAGATTTCCGTTTCACAGAT--------TCG

pAhHMA4-1 --------------TACAAGACCCATATCCAAATTTTTTTCTTATATAAAAAAAAGTTTT

pAhHMA4-2 --------------------------------------AAAAAATAAGT--------TCT

pAhHMA4-3 --------------------------------------AAAAAATAAGT--------TCT

pAtHMA4 TAACACTTCTCTCA-ACCTTTATCTGATCGCACCAAACCAGTTTTTTCGCATCG------

pNcHMA4-1-LC ------------------------------TGCGATG----TGATTTA------------

pNcHMA4-2-LC ------------------------------TGCGATG----TGATTTA------------

pNcHMA4-3-LC CCATTAATCCC--ATAATAATCTCT--TTTTATAATG----TGATTTG------------

pNcHMA4-1-Ga CTATTA-TCCC--ATAATAATCTCT--TTTTGCGATG----TGATTTA------------

pNcHMA4-2-Ga CTATTA-TCCC--ATAATAATCTCT--TTTTGCGATG----TGATTTA------------

pNcHMA4-3-Ga CCATTAATCCC--ATAATATTCTCT--TTTTATAATG----TGATTTG------------

pNcHMA4-1-CMA CCATTAATCCC--ATAATATTCTCT--TTTTATAATG----CGATTTG------------

pNcHMA4-2-CMA CTATTAATCCC--ATAATAATCTCT--TTTTGCGATG----TGATTTTCTTTTTTTCTTT

pNcHMA4-1-SLM CCATTAATCCC--ATAATATTCTCT--TTTTATAATG----CGATTTG------------

pNcHMA4-2-SLM ------------------------------TGCGATG----TGATTTA------------

pNcHMA4-3-SLM CCATTAATCCC--ATAATATTCTCT--TTTTATAATG----CGATTTG------------

pNcHMA4-4-SLM CCATTAATCCC--ATAATATTCTCT--TTTTATAATG----CGATTTG------------

pAhHMA4-1 GAACTGTTTTGTCATAATGATATCTCAAAG-ATAAAA--AATGTTTTTGCTTTG------

pAhHMA4-2 AAATTGTTTCATGATAATGATCTCCAACCTAAAAAAA----AGGTTTTGC----------

pAhHMA4-3 AAATTGTTTCATGATAATGATCTCCAACCTAAAAAAA----AGGTTTTGC----------

pAtHMA4 ------------------------------------------------------------

pNcHMA4-1-LC ------------------------------------------------------------

pNcHMA4-2-LC ------------------------------------------------------------

pNcHMA4-3-LC ------------------------------------------------------------

pNcHMA4-1-Ga ------------------------------------------------------------

pNcHMA4-2-Ga ------------------------------------------------------------

pNcHMA4-3-Ga ------------------------------------------------------------

pNcHMA4-1-CMA ------------------------------------------------------------

pNcHMA4-2-CMA TTAGATTTCCGTTTCACAGATTCGCTATTAATCCCATAATAATCTCTTTATACGATGTGA

pNcHMA4-1-SLM ------------------------------------------------------------

pNcHMA4-2-SLM ------------------------------------------------------------

pNcHMA4-3-SLM ------------------------------------------------------------

pNcHMA4-4-SLM ------------------------------------------------------------

pAhHMA4-1 ------------------------------------------------------------

pAhHMA4-2 ------------------------------------------------------------

pAhHMA4-3 ------------------------------------------------------------

pAtHMA4 ----GCTTCTTCCTTTTGCTACTAGCTCTCCTCT--------------CTTCTCCGGTAT

pNcHMA4-1-LC -------TTTTTTTCTTTTT---AGATTTCCGTT----------------TCACAGAT--

pNcHMA4-2-LC -------TTTTTTTCTTTTT---AGATTTCCGTT----------------TCACAGAT--

pNcHMA4-3-LC -------TTTTTTTCTTTTT---AGATTTCCGTT----------------TCACAGAT--

pNcHMA4-1-Ga -------TTTTTTTCTTTTT---AGATTTCCGTT----------------TCACAGAT--

pNcHMA4-2-Ga -------TTTTTTTCTTTTT---AGATTTCCGTT----------------TCACAGAT--

pNcHMA4-3-Ga -------TTTTTTTCTTTTT---AGATTTCCGTT----------------TCACAGAT--

pNcHMA4-1-CMA -------TTTTTTTCTTTTT---AGATTTCCGTT----------------TCACAGAT--

pNcHMA4-2-CMA TTTTGTTTTTTTTTCTTTTT---AGATTTCCGTT----------------TCACAGAT--

pNcHMA4-1-SLM -------TTTTTT-CTTTTT---AGATTTCCGTT----------------TCACAGAT--

pNcHMA4-2-SLM -------TTTTTTTCTTTTT---AGATTTCCGTT----------------TCACAGAT--

pNcHMA4-3-SLM --------TTTTTTCTTTTT---AGATTTCCGTT----------------TCACAGAT--

pNcHMA4-4-SLM -------TTTTTTTCTTTTT---AGATTTCCGTT----------------TCACAGAT--

pAhHMA4-1 ----TTCTTTTTTTTCTCCT-CAAAATCTGTCTTAAAGTAAGCAAAAGCCTATAAATCAA

pAhHMA4-2 -------TTTTTTTTTTCCTCAAAAACAATCATA----------------TATAAAAT--

pAhHMA4-3 -------TTTTTTTTTTCCTCAAAAACAATCATA----------------TATAAAAT--

pAtHMA4 TTTTGTCTCTCTTCTTAATTCACACAGATTTCATAAA

pNcHMA4-1-LC --TCGT---TAATCATATAAAACTTTGA-TACAGAA-

pNcHMA4-2-LC --TCGT---TAATCATATAAAACTTTGA-TACAGAA-

pNcHMA4-3-LC --TCGT---TAATCATAAAAAACTTTGA-TACAGAA-

pNcHMA4-1-Ga --TCGT---TAATCATAAAAAACTTTGA-TACAGAA-

pNcHMA4-2-Ga --TCGT---TAATCATAAAAAACTTTGA-TACAGAA-

pNcHMA4-3-Ga --TCGT---TAATCATAAAAAATTTTGA-TACAGAA-

pNcHMA4-1-CMA --TCGT---TAATCATAAAAAATTTTGA-TACAGAA-

pNcHMA4-2-CMA --TCGT---TAATCATAAAAAACTTTGA-TACAGAA-

pNcHMA4-1-SLM --TCGT---TAATCATAAAAAACTTTGA-TACAGAA-

pNcHMA4-2-SLM --TCGT---TAATCATAAAAAACTTTGA-TACAGAA-

pNcHMA4-3-SLM --TCGT---TAATCATAAAAAACTTTGA-TACAGAA-

pNcHMA4-4-SLM --TCGT---TAATCATAAAAAACTTTGA-TACAGAA-

pAhHMA4-1 TATAATTTGTAGTTTTGATTAACGT--TTTGCAGAAA

pAhHMA4-2 --TTAT----AGTTTTGATTTACGTTT--TGCAGAAA

pAhHMA4-3 --TTAT----AGTTTTGATTTACGTTT--TGCAGAAA

**Supplementary Alignment S3:** Alignment of *HMA4* promoter sequences from *A. thaliana*, *A. halleri* and different accessions of *N. caerulescens*. Comparisons of promoter sequences are based on the first 2,000 bp upstream of the translational start codon of *HMA4*.

**SUPPLEMENTARY SEQUENCES**

**Supplementary sequence S1.** pNcHMA4-1-LC

gcaagaaggacattgtaactatggtgcactttgccttaacaaaaatacattgttgccctttttatttgttttacctttacacaatttaactttatattttaccatttacacaatttatctctctccacgataaatccttctctctcttcttttctttctccactttattcatctccactttattcatctccactttccttctctctttgcttcctacaaattctctgtctttctcattttccaccaggttcttcttccaccaataaagattgggagggacaatggttgcgtaaagacattatgccttgaataagtgtttgtgtatgccctttttcaaaaaaagaaaaaagaataagtgtttgtgaacaaaatatcttggacagaattttagagtatatcacgtagatcgtggacaatatgccatggacaatgggtgtgtgaacatatatattccgacatataacaataaaatatgtacacaagcttcgattttcacctctgatacacaattcatccacatctttattatgtccaaacaaaagtaatccacggcaattaatctacataatttggctctctctttgattcttccactcttactttcatctttttatttccgtaatcacaagcaacaatgtttcattttcacttctcgttatcatcatatattacaattttctacccatgttaaccctatcaaaacaccaattggatacatgaaaacaattcaatcccacaccaaaccagaaatcgttttaatttctagcttcagtttcagaaaccaatttacgccaaaaaaaaaaaaaaaaaaaaaaaaaaaaaaaaaaaaatttacgccaaaaacgttggttactgtgaaacacacaccaaaccagaaatcaccatcaacagaaatagccaaattcatccacaattgtataagtaaccgtacaaacgtatataacaaagcgtacacatattttcaattgtccaaaaacacattgtactccaattaaacgaagctaccagatccatgtcctctcccagaacagaaaataaacactcgtcaagataaccaaatatcatattatttatgaataagtaaaagtgtaaaactcatgcgatgcaatttcctccagccgtatcagtctctaagtggaaattgtgaaagtccaccactttcacaatttccctcttataaaagtcactcttactgtaaatgactcctgttagaactacctaaaagcaggttatggaattcgttggtcaaataaatttgaaaaaaaaacaaattataaattgttatgaaagaaaatatctcgtacacaagtgtcaccaagtgtaagggacccaaatctttgttgaaagaagatgaagttaacaaaaatacttttgccttctctctctctccatcgaatactattatcccactttgcttccctctctcttttaatatctcacctttataataaaaagctgataattcgaaaatgttaaagaacaaaaaccaatcatctaacattctgacttatctacaggggcggcttattagagtggggtcaatagatgctctgcactaggtgacgtagaaaaacaaaattttagtacagaaaaaagaatacaaacaaaaattagtagaaaaaaaagtacacttaaaaatatagttttaattttgcacttacgtatacttataaaatttttgacccggccctgcttatccggctatataaagcaactaccatttctagatatcttcacctcacaatcttcctctctacgttctaaaacctctctcactctcagtcttcacctttgtggtaatactttaatctgatcgaaccgcaccaaaccggtccggtctttcttctcggcctcgtcttttctccggtattctttctcttcttaattcacataaatttcataacaagtgatttcttcgtaaaaattaaaatccgatcaaattcacgatagtgatatctccaacacgttatatgcatcccagcataaaagttttgctttcttaatttttttcccttaaaagattggaaaagtagccattaatcccataataatctctttttgcgatgtgatttatttttttctttttagatttccgtttcacagattcgttaatcatataaaactttgatacagaaATG

**Supplementary sequence S2.** pNcHMA4-2-LC

gcaagaaggacattgtaactatggtgcactttgccttaacaaaaatacattgttgccctttttatttgttttacctttacacaatttaactttatattttaccatttacacaatttatctctctccacgataaatccttctctctcttcttttctttctccactttattcatctccactttattcatctccactttccttctctctctgcttcctacaaattctctgtctttctcattttccaccaggttcttcttccaccaataaagattgggagggacaatggttgcgtaaagacattatgccttgaataagtgtttgtgtatgccctttttcaaaaaaagaaaaaagaataagtgtatgtgaacaaaatatcttggacagaattttagagtatatcacgtagatcgtggacaatatgccatggacaatgggtgtgtgaacatatatattccgacatataacaataaaatatgtacacaagcttcgattttcacctctgatacacaattcatccacatctttattatgtccaaacaaaagtaatccacggcaattaatctacataatttggctctctctttgattcttccactcttactttcatctttttatttccgtaatcacaagcaacaatgtttcattttcacttctcgttatcatcatatattacaattttctacccatgttaaccctatcaaaacaccaattggatacatgaaaacaattcaatcccacaccaaaccagaaatcgttttaatttctagcttcagtttcagaaaccaatttatgccaaaaacattggttactgtgaaacacatgcacaccaaaccagaaatcaccatcaacagaaatagccaaattcatccacaattgtataagtaaccggtaaagtaaccggtacaaacgtatataacaaggcgtacacatattttcaattgtccaaacacacattgtactccaattaaacgaagctaccagatccatgtcctctcccagaacagaaaataaacactcgtcaagataaccaaatatcatattatttatgaataagtaaaagtgtaaaactcatgcgatgcaatttcctccagccgtatcagtctctaagtggaaattgtgaaagtccaccactttcacaatttccctcttataaaagtcactcttactgtaaatgactcctgttagaactacctaaaagcaggttatggaattcgttggtcaaatgaatttgaaaaaaaaacaaattataaattgttatgaaagaaaatatctcgtacacaagtgtcaccaagtgtaagggacccaaatctttgttgaaagaagatgaagttaacaaaaatacttttgccttctctctctctccatcgaatactattatcccactttgcttccctctctcttttaatatctcacctttataataaaaagctgataatttcgaaaatgttaaagaacaaaaaccaatcatctaagagcatccgcatcgctcatccccactgaggttctgttatttaaaagaaaagaaaaaaattaaataataatgagctggcatagaaaaattaaacaatcgattctctaggaagcatttttggagtcgagtctgctatagggttctgtcacgtgtcacgccacaattgaatccttatttttttatttttattttttacctcgaaaatcatttttattaataaaaaaaattaaagagctcgcgtgtggggatgagcgatgcggatgctctaacattctgacttatctacaggggcggcttattagagtggggtcaatagatgctctgcactaggtgacgtagaaaaacaaaattttagtacagaaaaaagaatacaaacaaaaattagtagaaaaaaaagtacacttaaaaatatagttttaattttgcacttacgtatacttataaaatttttgacccggccctgcttatccggctatataaagcaactaccatttctagatatcttcacctcacaatcttcctctctacgttctaaaacctctctcactctcaaactttaatctgatcgaaccgcaccaaaccggtccggtctttcttctcggcctcgtcttttctccggtattctttctcttcttaattcacataaatttcataacaagtgatttcttcgtaaaaattaaaatccgatcaaattcacgatagtgatatctcctacacgttatatgcatcccagcataaaagttttgctttcttaatttttttcccttaaaagattggaaaagtagccattaatcccataataatctctttttgcgatgtgatttatttttttctttttagatttccgtttcacagattcgttaatcatataaaactttgatacagaaATG

**Supplementary sequence S3.** pNcHMA4-3-LC

gcaagaaggacattgtaactatggtgcactttgccttaacaaaaatacattgttgccctttttatttgttttacctttacacaatttaactttatattttaccatttacacaatttatctctctccacgataaatccttctctctcttcttttctttctccactttattcatctccactttattcatctccactttccttctctctctgcttcctacaaattctctgtctttctcattttccaccaggttcttcttccaccaataaagattgggagggacaatggttgcgtaaagacattatgccttgaataagtgtttgtgtatgccctttttcaaaaaaagaaaaaagaataagtgtatgtgaacaaaatatcttggacagaattttagagtatatcacgtagatcgtggacaatatgccatggacaatgggtgtgtgaacatatatattccgacatataacaataaaatatgtacacaagcttcgattttcacctctgatacacaattcatccacatctttattatgtccaaacaaaagtaatccacggcaattaatctacataatttggctctctctttgattcttccactcttactttcatctttttatttccgtaatcacaagcaacaatgtttcattttcacttctcgttatcatcatatattacaattttctacccatgttaaccctatcaaaacaccaattggatacatgaaaacaattcaatcccacaccaaaccagaaatcgttttaatttctagcttcagtttcagaaaccaatttatgccaaaaacattggttactgtgaaacacatgcacaccaaaccagaaatcaccatcaacagaaatagccaaattcatccacaattgtataagtaaccggtacaaacgtatataacaaggcgtacacatattttcaattgtccaaacacacattgtactccaattaaacgaagctaccagatccatgtcctctcccagaacagaaaataaacactcgtcaagataaccaaatatcatattatttatgaataagtaaaagtgtaaaactcatgcgatgcaatttcctccagccgtatcagtctctaagtggaaattgtgaaagtccaccactttcacaatttccctcttataaaagtcactcttactgtaaatgactcctgttagaactacctaaaagcaggttatggaattcgttggtcaaatgaatttgaaaaaaaaacaaattataaattgttatgaaagaaaatatctcgtacacaagtgtcaccaagtgtaaggggacccaaatctttgttgaaagagatgaagttaacaaaaatacttttgccttctctctctctccatcgaatactattatcccactttgcttccctctctcttttaatatctcacctttataataaaaagctgataatttcgaaaatgttaaagaacaaaaaccaatcatctaagagcatccgcatcgctcatccccactgaggttctgttatttaaaagaaaagaaaaaaattaaataataatgagctggcatagaaaaattaaacaatcgattctctaggaagcatttttggagtcgagtctgctataggttctgtcacgtgtcacgccacaattgaatccttattttttttatttttattttttacctcgaaaatcatttttattaataaaaaaaaattaaagagctcgcgtgtggggatgagcgatgcggatgctctaacattctgacttatctacaggggcggcttattagagtggggtcaatagatgctctgcactaggtgacgtagaaaaacaaaattttagtacagaaaaaagaatacaaacaaaaattagtagaaaaaaaagtacacttaaaaatatagttttaattttgcacttacgtatacttataaaatttttgacccggccctgcttatccggctatataaagcaactaccatttctagatatcttcacctcacaatcttcctctctacgttctaaaacctctctcactctcaaactttaatctgatcgaaccgcaccaaaccggtccggtctttcttctcggcctcgtcttttctccggtattctttctcttcttaattcacataaatttcataacaagtgatttcttcgtaaaaattaaaatccgatcaaattcacgatagtgatatctcctacacgttatatgcatcccagcataaaagtttgctttcttaattttttcccttaaaagattggaaaagtagccattaatcccataataatctcttttgcgatgtgatttatttttttctttttagatttccgtttcacagattcgccattaatcccataataatcttgatttgtttttttatttttagatttccgtttcacagattcgccattaatcccataataatctctttttataatgtgatttgtttttttctttttagatttccgtttcacagattcgttaatcataaaaaactttgatacagaaATG

**Supplementary sequence S4.** pNcHMA4-1-Ga

gcgaggatcatgtgtctaaactagcgacgtatcggacaagttttatcctcgccccatattcaaactgataatgttttataatctcacttttcttttgtaaccattttatataaagtgttaatagatatataccatatttttatcccaaaaacttagaatatgtagttgttttgataaaactctaattgatcatctactccataaaaagctaatttcgaaatttataaaacaaagtcacatgcacaaacaacttatcttgtgattaaggatgtttttacttatgactcagctaggttcaaatctcaaaaacatagccaattcaaattttatgaagttccgtactactgtaaaatgagccatcaatcgtttttaaaaaggagttagattagactagtctataatccattatagtgaaaactgctacacaaaatatcatacttttatatagtgctaatgtaatcgattttaaaataaacttacagttttatattcttggaaattactgaaaacaataagaaattacatcttgataggaactaggttgaaaattcggaaggaatataggaattcgaaacaaagattaaaatatcctacgaaattaacatagtaaaaaaaaaaactaaaccaaataaaaaaagtttaacgtaaaaagaaaagttttaattcaaagaatccaaactaaccacaacttttgaaacataatccataaatgttaggtttagaacggcttttacagatttagtaactattctcaaatcatttagtacgtctttcttaaacttctaagaaccaaatttgtggtatatatttgatatatttgagttatctttttcgttcgactttgatgtaattgaattttttgggggatttcgggaaagcaaaatatacatttcggtactgttagaacgagaaaattacatcaataaggactttttgacttatcttattacagcaagaaggacagtgtaactatggtgcactttgccttaacaaaaatacattgttgctctttttatttgttttacctttacacaatttaactttatattttaccagttacacaatttatctctctccacgataaatccttctctctcttcttttctttctccactttattcatctccactttccttatctctttgcttcctacaaattctctgtctttctcattttccactaggttcttcttccaccaataaagattgggacaagggttgcgtaaagacattattatgccttgaataagtgtttgtgtatgcatgccctttttcaaaaaaaaaaaaaaagaataagtgtttgtgaacaaaatatcttggatagaattttagaatatgattgacaaaaaaaaaaaagaattttagaatatatcacgtagatcgtggacaatatgccatggacaatatgggtgtgtgaacatatatatattcagacatataacaataaaatatgtacacaagcttcgattttcacctctgatacacaattcatccacatctttattatgtccaaacaaaagtaatccacggcaattaatctacataatttggctttctctttgattcttccactcttactttcatctttttatttccgtaatcacaagcaacaatgtttcattttcacttctcgttatcatcatatattacaattttctacccatgttaaccctattaaaacaccaattggatacatgaaaacaattcaatcccacaccaaaccagaaatcgttttaatttctagcttcagtttcagaaaccaatttacgccaaaaacgttgtttactgtgaaacacacaccaaaccaaaaatcaccatcacaaaaatagccaaattcatccataattgtataagtaaccgtacaaacgtatataacaaagcgtacacatgttttcaattgtccaaaaacacattttactacaattaaacgaagctatcagatccatgtcctctcccagaaaagaaaataaacactcgtcaagataaccatattatttatgaataagtaaaattcatgcgatgcaatttcctccagccgtatcagtctctacgtggtgcggtcgccagagttaaattaaaagacaccaaccaaaataatctgatttttcacccctttttcattaaaggtaaaatggaactctcaacacactataagcaaaaaagaaaaaaagtccaccactttcacaatttccctcttataaaagtcactcttagtgtaaatgacaaatgactcctgttagaactacctaaaagcaggttatggaattcgttggtcaaatgaattaaaaaaaaaaaaaaacaaattataaattgttatgaaagaaaatatctcgtacacaagtgtcaccaagtataagggacccatctttgttgaaagaagatgaagttaacaaaaaaacttttgccttctctctctctccatcgaatactactattatcccataataatctctttttgcgatgtgatttatttttttctttttagatttccgtttcacagattcgttaatcataaaaaactttgatacagaaATG

**Supplementary sequence S5.** pNcHMA4-2-Ga

gcgaggatcatgtgtctaaactagcgacgtatcggacaagttttatcctcgccccatattcaaattgataatgttttataatctcacttttcttttgtaaccattttatataaagtgttaatagatatataccatatttttatcccaaaaacttaagagtatgtagttgttttgataaaactctaattgatcatctactccataaaaagctaatttcgaaatttataaaacaaagtcacatgcacaaacaacttatcttgtgattaaggatgtttttacttatgactcagctaggttcaaatctcaaaaacatagccaattcaaattttatgaagttcggtactactgtaaattgagccaccaatcgtttttaaaaaggagttcgattagactagtctataatccattatagtgaaaattgctacacaaaatatcatacttttatatagtgctaatgtaatcgattttaaaataaacttatagttttatattcttggaaattactgaaaacaaaaagaaactacattttgataggaactagcctgaaaattcggaaggaaaataggaattcgaaacaaagattaaaatatcctacgaaattaacatggtaaaaaaaactaaaccaaataaaaaaagtttaacgtaaaaagaaaagttttaattcaaaagaatccagactaaccacaacttttgaagcataatccataaatgttaggtttagaacgacttttaaagatttagtaactattttcaaatcatttagtacgtctttcttttttttttttggctcaaacataacagatttcattagaaatgatttcattagtacgtctttcttaaacttctaagaaccaaatttgtggtatatatttgatatatttgagttatctttttcgttcgactttgatgtaattgaattttttgggggatttcgggaaagcaaaatatacatttcggtactgttagaacgagaaaattacatcaataaggactttttgacttatcttattacagcaagaaggacagtgtaactatggtgcactttgccttaacaaaaatacattgttgctctttttatttgttttacctttacacaatttaactttatattttaccagttacacaatttatctctctccacgataaatccttctctctcttcttttctttctccactttattcatctccactttccttatctctttgcttcctacaaattctctgtctttctcattttccactaggttcttcttccaccaataaagattgggacaagggttgcgtaaagacattattatgccttgaataagtgtttgtgtatgcatgccctttttcaaaaaaaaaaaaaaagaataagtgtttgtgaacaaaatatcttggatagaattttagaatatgattgacaaaaaaaaaaaagaattttagaatatatcacgtagatcgtggacaatatgccatggacaatatgggtgtgtgaacatatatatattcagacatataacaataaaatatgtacacaagcttcgattttcacctctgatacacaattcatccacatctttattatgtccaaacaaaagtaatccacggcaattaatctacataatttggctttctctttgattcttccactcttactttcatctttttatttccgtaatcacaagcaacaatgtttcattttcacttctcgttatcatcatatattacaattttctacccatgttaaccctattaaaacaccaattggatacatgaaaacaattcaatcccacaccaaaccagaaatcgttttaatttctagcttcagtttcagaaaccaatttacgccaaaaacgttgtttactgtgaaacacacaccaaaccaaaaatcaccatcacaaaaatagccaaattcatccataattgtataagtaaccgtacaaacgtatataacaaagcgtacacatgttttcaattgtccaaaaacacattttactacaattaaacgaagctatcagatccatgtcctctcccagaaaagaaaataaacactcgtcaagataaccatattatttatgaataagtaaaattcatgcgatgcaatttcctccagccgtatcagtctctacgtggtgcggtcgccagagttaaattaaaagacaccaaccaaaataatctgatttttcacccctttttcattaaaggtaaaatggaactctcaacacactataagcaaaaaagaaaaaaagtccaccactttcacaatttccctcttataaaagtcactcttagtgtaaatgacaaatgactcctgttagaactacctaaaagcaggttatggaattcgttggtcaaatgaattaaaaaaaaaaaaaaaacaaattataaattgttatgaaagaaaatatctcgtacacaagtgtcaccaagtataagggacccatctttgttgaaagaagatgaagttaacaaaaaaacttttgccttctctctctctccatcgaatactactattatcccataataatctctttttgcgatgtgatttatttttttctttttagatttccgtttcacagattcgttaatcataaaaaactttgatacagaaATG

**Supplementary sequence S6.** pNcHMA4-3-Ga

tgccttgaataagtgtttgtgtatgcatgccctttttcaaaaaaaaaaaaaaaagaataagtgtttgtgaacaaaatatcttggatagaattttagaatatatcacgtagatcgtggacaatatgccatggacaataggtgtgtgaacatatatatatatatatatatattcagacatataacaataaaatatgtacacaagcttcgattttcacctctgatacaacaattcatccacatctttattatgtccaaacaaaagtaatccacggcaataaatctacataatttggctctctttgattcttccactcttagtttcatctttttatttccgtaatcacaagcaacaatgtttcattttcacttctcgttatcatcatatattacaattttttacccatgttaaccctatcaaaacaccaattagatacatgaaaacaattcaatcccacaccaaaccagaatcgttttaatttatagcttcagtttcagaaattaatatacgccaaaaacgttggtttggttactgtgaaacacacaccaaaccaaaaatcaccatcacagaaatagccaaatttatccacaattgtataagtaaccgtacaaacgtatataacaaagcctacacatattttgaattgtccaaaaacacattttactccaattaaacgaagctatcagatccatgtcctctaccaaaacagaaaataaacactagtcaagataaccaaatatcatattatttatgaataagtaaaactcatgcgatgcaatttcctccagccgtatcagtctttatgtggtgcggtcgagttaaattaaaagacaccaaccaaaataatctgatttttcacctctttttcattaaaggtaaaatggaactctcaacacactataagcaaaaaaaaaaaaaaaaaaagtctttgcgtaaagagagttataaaagtaaatatctcgtacacaagtgtcaccaagtgaacatacattagtcaatgttagtattttaatattctcttttagctatatataagggacccatctttgttgaaagaagatgaagttcacaaaaaaacttttgccttctctctctatcgaatactgttatctcactttccttcccttttccttccctctctcttttaatatctcacctttatatataatattttataacattaataatttttttaataaatcctaataggggtatgatataaatggtcaaagaaggtggaagaagcgtgaggattactgtggaggaaaaagagacattggagaaagcaacggtcaacattactgtcagatgtcgaaggagagaaagtgagagagtgtgagactctgagagagagagaagtcaagaaggagaagaagacaaaagctaatttaaagctacgaataattatataatatggcgaagacgagacgggacatatattcaccctcgcttttcacatatattttcggtattgccactctcaaattttatttttttccttttttcttgtcttttttgacccggccctgcttatttggctatataaagcaactaccttatctagatatcttcacctcgcaatcttcctctctacgttccaaaacctctctcactctctgtcttcacctttgtggtaatacttaatctctgatcgaaccgcaccaaaccagtccggtctttcttctcggcctcgtcttttctccggtattctttctcttcttaattcacatagatttcataacaagtgattttttcgtaataattaaaatccgatcaaattcacgatagtgatatgatatatgcatatatgcatccaacacgttatatgcatcccagcataaaagttttgctttcttaattttttttcccttaaaagatttggaaaattagccattaatcccataataatctctttttgcgatgtgatttgtttttttctttttagatttccgtttcacagattcgccattaatcccctaataatctcggtttgtttttttatttttagatctccgtttcacagattcgccattaatcccataatattctctttttataatgtgatttgtttttttctttttagatttccgtttcacagattcgttaatcataaaaaattttgatacagaaATG

**Supplementary sequence S7.** pNcHMA4-1-CMA

ctccctcaacaaagtgatatcttctctccctcacgataaatccttctctctcttcttttctttctccactttattcatctccactttccttctctctttgcttcctacaaattctgtctctcattttccactaggttcttcttccaccaataaagattgggacaagggttgcgtaaagacattattatgccttgaataagtgtttgtgtatgcatgccctttttcaaaaaaaaaaaaaaagaataagtgtttgtgaacaaaatatcttggatagaattttagaatatatcacgtagatcgtggacaatatgccatggacaataggtgtgtgaacatatatatatatatatatatatatatattcagacatataacaataaaatatgtacacaagcttcgattttcacctctgatacaacaattcatccacatctttattatgtccaaacaaaagtaatccacggcaataaatctacataatttggctctctttgattcttccactcttactttcatctttttatttccgtaatcacaagcaacaatgtttcattttcacttctcgttatcatcatatattacaattttttacccatgttaaccctatcaaaacaccaattagatacatgaaaacaattcaatcccacaccaaaccagaaatcgttttaatttatagcttcagtttcagaaattaatatacgccaaaaacgttggttactgtgaaacacacaccaaaccaaaaatcaccatcacagaaatagccaaatttatccacaattgtataagtaaccgtacaaacgtatataacaaagcctacacatattttgaattgtccaaaaacacattttactccaattaaacgaagctatcagatccatgtcctctaccaaaacagaaaataaacactagtcaagataaccaaatatcatattatttatgaataagtaaaactcatgcgatgcaatttcctccagccgtatcagtctctatgtggtgcggtcgagttaaattaaattcaatgacaccaaccaaaataatctgatttttcacccctttttcattaaaggtaaaatggaactctcaacacactataagcaaaaaaaaaaaaaaaaaatctttgcgtaaagagagttataaaagtaaatatctcgtacacaagtgtcaccaagtgaacatacattagtcaatgttagtattttaatattctcttttagctatatataagggacccatctttgttgaaagaagatgaagttcacaaaaaaactttgccttctctctctatcgaatactgttatctcactttccttcccttttccttccctctctcttttaatatctcacctttatatataatattttataacattaataatttttttaataaatcctaataggggtatgatataaatggtcaaagaaggtggaagaagcgtgaggattactgtggaggaaaaagagacattggagaaagcaacggtcaacattactgtcagatgtcgaaggagagaaagtgagagagtgtgagactctgagagagagagaagtcaagaaggagaagaagacaaaagctaatttaaagctacgaataattatataatatggcgaagacgagacgggacatatattcaccctcgcttttcacatatattttcggtattgccactctcaaattttatttttttccttttttcttgtcttttttgacccggccctgcttatttggctatataagcaactaccttatctagatatcttcacctcgcaatcttcctctctacgttccaaaacctctctcactctctgtcttcacctttgtgttaatactttaatctctgatcgaaccgcaccaaaccagtccggtctttcttctcggcctcgtcttttctccggtattctttctcttcttaattcacatagatttcataacaagtgatttttttcgtaataattaaaatccgatcaaattcacgatagtgatatgatatatgcatatatgcatccaacacgttatatgcatcccagcataaaagttttgctttcttaattttttttcccttaaaagatttggaaaattagccattaatcccataataatctctttttgcgatgtgatttgtttttttctttttagatttccgtttcacagattcgccattaatcccctaataatctcggtttgtttttttatttttagatctccgtttcacagattcgccattaatcccataatattctctttttataatgcgatttgtttttttctttttagatttccgtttcacagattcgttaatcataaaaaattttgatacagaaATG

**Supplementary sequence S8.** pNcHMA4-2-CMA

ctcctcaacaaagtgatatcttctctccctcacgataaatccttctctctcttcttttctttctccactttattcatctccactttccttctctctttgcttcctacaaattctgtctctcattttccactaggttcttcttccaccaataaagattgggacaagggttgcgtaaagacattattatgccttgaataagtgtttgtgtatgcatgccctttttcaaaaaaaaaaaaaaagaataagtgtttgtgaacaaaatatcttggatagaattttagaatatatcacgtagatcgtggacaatatgccatggacaataggtgtgtgaacatatatatatatatatatatatatattcagacatataacaataaaatatgtacacaagcttcgattttcacctctgatacaacaattcatccacatctttattatgtccaaacaaaagtaatccacggcaataaatctacataatttggctctctttgattcttccactcttactttcatctttttatttccgtaatcacaagcaacaatgtttcattttcacttctcgttatcatcatatattacaattttttacccatgttaaccctatcaaaacaccaattagatacatgaaaacaattcaatcccacaccaaaccagaaatcgttttaatttatagcttcagtttcagaaattaatatacgccaaaaacgttggttactgtgaaacacacaccaaaccaaaaatcaccatcacagaaatagccaaatttatccacaattgtataagtaaccgtacaaacgtatataacaaagcctacacatattttgaattgtccaaaaacacattttactccaattaaacgaagctatcagatccatgtcctctaccaaaacagaaaataaacactagtcaagataaccaaatatcatattatttatgaataagtaaaactcatgcgatgcaatttcctccagccgtatcagtctctatgtggtgcggtcgagttaaattaaattcaatgacaccaaccaaaataatctgatttttcacccctttttcattaaaggtaaaatggaactctcaacacactataagcaaaaaaaaaaaaaaaaaatctttgcgtaaagagagttataaaagtaaatatctcgtacacaagtgtcaccaagtgaacatacattagtcaatgttagtattttaatattctcttttagctatatataagggacccatctttgttgaaagaagatgaagttcacaaaaaaacttttgccttctctctctatcgaatactgttatctcactttccttcccttttccttccctctctcttttaatatctcacctttatatataatattttataacattaataatttttttaataaatcctaataggggtatgatataaatggtcaaagaaggtggaagaagcgtgaggattactgtggaggaaaaagagacattggagaaagcaacggtcaacattactgtcagatgtcgaaggagagaaagtgagagagtgtgagactctgagagagagagaagtcaagaaggagaagaagacaaaagctaatttaaagctacgaataattatataatatggcgaagacgagacgggacatatattcaccctcgcttttcacatatattttcggtattgccactctcaaattttatttttttccttttttcttgtcttttttgacccggccctgcttatttggctatataaagcaactaccttatctagatatcttcacctcgcaatcttcctctctacgttccaaaacctctctcactctctgtctactctctgtcttcacctttgtggtaatactttaatctctgatcgaaccgcaccaaaccagtccggtctttcttctcggcctcgtcttttctccggtattctctcttcttaattcacatagatttcataacaagtgatttcttcgtaaaaattaaaatccgatcaaattcacgacagtgatatgatatatgcatatatgcatccaacacgttatatgcatcccagcataaaagttttgctttcttaattttttttcccttaaaagatttggaaaataagccattaatcccataataatctctttttgcgatgtgatttgtttttttctgttttagatttccgtttcacagattcgctattaatcccataataatctcgatttgttttttttgtttttttttagatttccgtttcacagatttgccattaatccaataataatctctttttgcgatgtgattttgtttttttctttttagatttccgtttcacagattcgctattaatcccataataatctcgatttgttttttatttttatttttagatttccgtttcacagattcgctattaatcccataataatctctttttgcgatgtgattttctttttttctttttagatttccgtttcacagattcgctattaatcccataataatctctttatacgatgtgattttgtttttttttctttttagatttccgtttcacagattcgttaatcataaaaaactttgatacagaaATG
